# Aplospojaveedins A–C, unusual sulfur-containing alkaloids produced by the endophytic fungus *Aplosporella javeedii* using OSMAC strategy

**DOI:** 10.3389/fmicb.2024.1458622

**Published:** 2024-09-27

**Authors:** Ying Gao, Marian Frank, Nicole Teusch, Dennis Woschko, Christoph Janiak, Attila Mándi, Tibor Kurtán, Rudolf Hartmann, Katja Schiedlauske, Lasse van Geelen, Rainer Kalscheuer, Jesko Kaiser, Christoph G. W. Gertzen, Holger Gohlke, Bin-Gui Wang, Peter Proksch, Zhen Liu

**Affiliations:** ^1^Key Laboratory of Study and Discovery of Small Targeted Molecules of Hunan Province, School of Medicine, Hunan Normal University, Changsha, China; ^2^Institute of Pharmaceutical Biology and Biotechnology, Heinrich Heine University, Düsseldorf, Germany; ^3^Institute of Inorganic and Structural Chemistry, Heinrich Heine University, Düsseldorf, Germany; ^4^Department of Organic Chemistry, University of Debrecen, Debrecen, Hungary; ^5^Institute of Biological Information Processing: Structural Biochemistry (IBI-7), Forschungszentrum Jülich GmbH, Jülich, Germany; ^6^Institute for Pharmaceutical and Medicinal Chemistry, Heinrich Heine University, Düsseldorf, Germany; ^7^Institute of Bio- and Geosciences (IBG-4: Bioinformatics), Forschungszentrum Jülich GmbH, Jülich, Germany; ^8^CAS and Shandong Province Key Laboratory of Experimental Marine Biology, Institute of Oceanology, Chinese Academy of Sciences, Qingdao, China

**Keywords:** *Aplosporella javeedii*, alkaloids, OSMAC approach, X-ray diffraction, DFT-NMR calculations, TDDFT-ECD calculations, target prediction

## Abstract

Three sulfur-containing alkaloids aplospojaveedins A–C (1–3) with a hitherto undescribed carbon skeleton comprising octahy-dronaphthalene, *α*, *β*-unsaturated lactam and glycine-cysteine moieties were isolated from *Aplosporella javeedii*. Their structures were elucidated by 1D and 2D NMR spectroscopy, HR-MS, X-ray diffraction analysis, DFT-NMR and TDDFT-ECD calculations. A plausible biosynthetic pathway and putative targets are described. The blind docking suggested that 1–3 may have functional effects on several putative targets such as the GPCR cannabinoid receptor 2 or the integrin *α*5*β*1 complex.

## Introduction

During the discovery of new pharmaceutical agents from nature, microorganisms have repeatedly proved to be prolific producers of structurally novel and bioactive compounds that inspire drug discovery (Newman and Cragg, 2020; Zhao et al., 2023). In particular, sulfur-containing natural products, such as the well-known penicillin, gliotoxin, and calicheamicins, have often attracted the attention of researchers because of their novel molecular architectures and interesting bioactivities (Hai et al., 2021; Vasilchenko et al., 2023). Recent discoveries further expanded the chemical diversities of sulfur-containing natural products. For example, the first case of noremestrin featuring a sulfur-bearing 15-membered macrocyclic lactone was isolated from *Emericella* sp. (Chen et al., 2024) Neogrisemycin, a trisulfide-bridged angucycline, was obtained from *Streptomyces albus* (Cao et al., 2023). Heterologous expression of a biosynthetic gene cluster in *Streptomyces* sp. SCSIO 40020 led to the production of eight new thiazole-containing compounds, in which grisechelin F is distinguished by the presence of a salicylic acid moiety (Liu et al., 2024).

**Figure 1:**
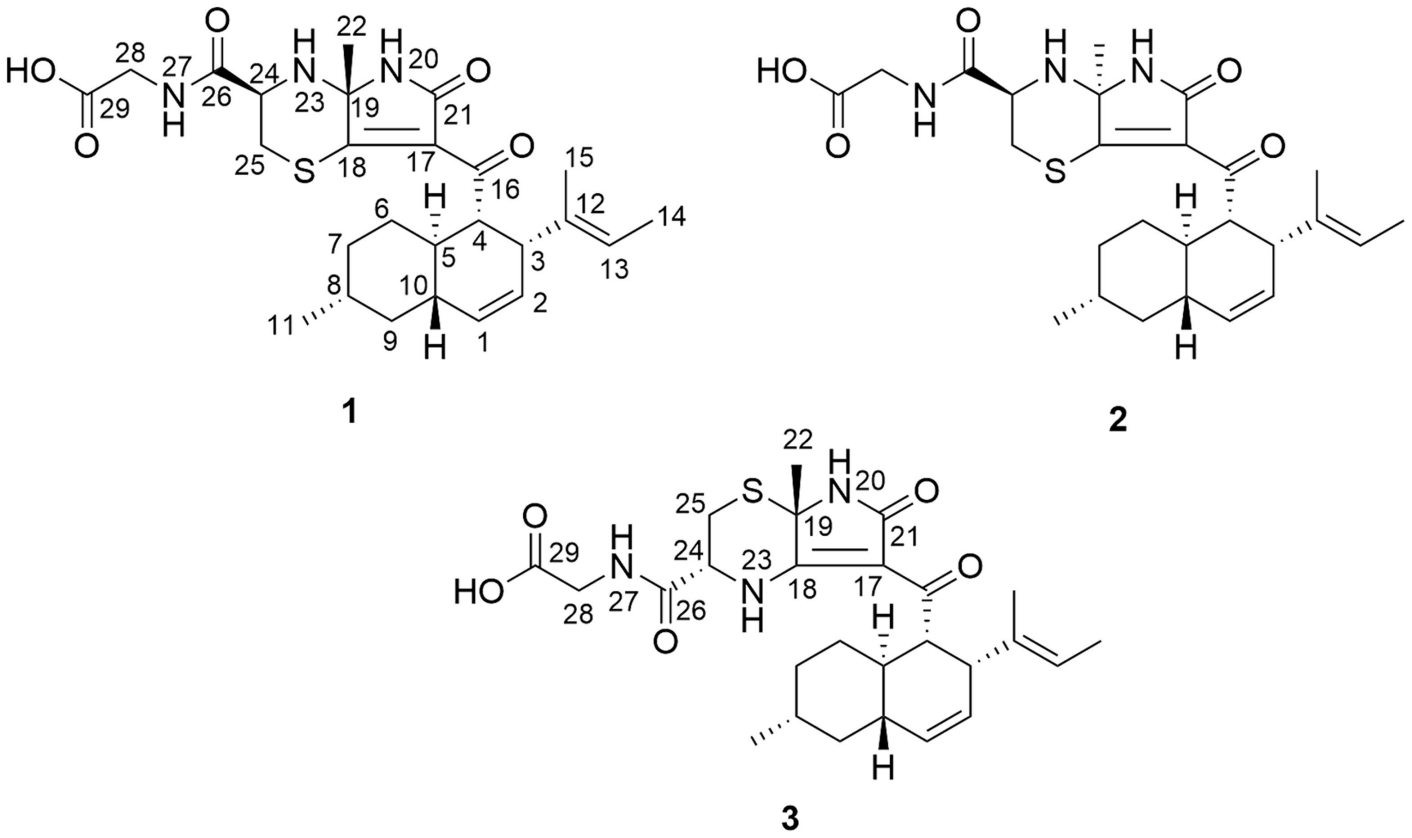
Structures of new alkaloids isolated from *A. javeedii*.

In our ongoing investigation of bioactive new natural products, an underexplored endophytic fungus *Aplosporella javeedii*, which was derived from the Chinese medicinal plant *Orychophragmus violaceus*, was investigated (Medicinal Plant Images Database, 2024). In a previous study, a culture of *A. javeedii* on solid rice medium yielded polyketides, sesterterpenes and macrolides (Gao et al., 2020a; Gao et al., 2020b), whereas addition of NaNO_3_ or monosodium glutamate to rice medium caused the accumulation of new cytotoxic lactam derivatives that were hitherto undetected (Gao et al., 2020c). We now report that addition of NaI to the culture medium elicited the accumulation of biogenetically very different compounds which feature a hitherto undescribed carbon skeleton and contain the biogenetic building blocks octaketide, alanine, L-cysteine, and glycine (Figure 1). These compounds were not detected when NaI was absent or when adding other halogen salts to the medium thereby indicating the power of this OSMAC approach. Herein we report the structural elucidation of these new sulfur-containing alkaloids and propose a plausible biogenetic pathway. Furthermore, putative targets of these compounds are predicted by molecular chemoinformatic approaches.

## Materials and methods

### General experimental procedures

A Perkin-Elmer-241 MC polarimeter was used for measuring optical rotations. HPLC experiments were performed using a Dionex UltiMate-3400SD system equipped with a LPG-3400SD pump and a photodiode array DAD 3000RS detector as well as a Eurosphere-10 C_18_ (125 × 4 mm, Knauer) column. Detection wavelengths were set at 235, 254, 280, and 340 nm. The following program and gradient was applied for analysis: (MeOH, 0.1% HCOOH in H_2_O): 0 min, (10% MeOH); 5 min (10% MeOH); 35 min (100% MeOH); 45 min (100% MeOH). A Merck Hitachi Chromaster HPLC system with UV detector L7400, pump L7100 and column Eurosphere-100 C_18_ (300 × 8 mm, Knauer) column were used for semi-preparative HPLC analysis. A Finnigan LCQ Deca mass spectrometer was used for electrospray ionization mass spectrometry (ESI-MS). A UHR-QTOF maxis 4G mass spectrometer (Bruker Daltonics) was used for HRESIMS analysis. 1D and 2D NMR spectra were recorded on Bruker Avance III HD 800, 750 MHz, and Bruker ARX 600 NMR spectrometers. A J-810 spectropolarimeter was used for ECD spectra analysis. Sephadex LH-20, silica gel 90C_18_-reversed phase RP-18 and silica gel 60 M (40–63 μm, Macherey-Nagel) were employed for column chromatography. TLC was done using TLC plates precoated with silica gel 60 F_254_ (Merck) and was detected under UV detection at 254 and 366 nm or was sprayed with anisaldehyde reagent. Distilled or spectral-grade solvents were used for column chromatography and spectroscopic measurements, respectively.

### Fungal material and fermentation

The endophytic fungus *Aplosporella javeedii* (GenBank accession number: MN720704) was isolated from fresh and healthy stem tissue of the Chinese medicinal herb *Orychophragmus violaceus* (L.) O. E. Schul (Brassicaceae), which was collected close to Beijing, China. Fungal isolation, purification, and identification were carried out according to a standard protocol that was described previously (Kjer et al., 2010). The fungal strain (ID code ZGB-B) is kept in the Institute of Pharmaceutical Biology and Biotechnology, Heinrich Heine University, Duesseldorf, Germany. Fungal control cultivations were done in two 1 L Erlenmeyer flasks on solid rice medium (100 g rice and 110 mL demineralized water). OSMAC experiments were performed in 16 1 L Erlenmeyer flasks on solid rice medium containing 3.5% NaI (100 g rice, 110 mL demineralized water and 3.5 g NaI) each. Cultivation media were autoclaved at 121°C for 20 min. After cooling to room temperature, the fungal strain was transferred from agar plates onto the solid media under sterile conditions. Fermentation was conducted at room temperature under static conditions for around 20 days for control cultivation and for 30 days for NaI OSMAC experiments. The experiments were terminated once the fungus had completely overgrown the media.

### Extraction and isolation

Solid media containing the fungal cultures were cut into small pieces and extracted with 800 mL EtOAc per flask under continuous shaking for 12 h. Following filtration, the extracts were evaporated to dryness. The total amount of the obtained brown crude extract from 3.5% NaI OSMAC cultures was 16.5 g. The crude extract was then subjected to silica gel vacuum liquid column chromatography (VLC), and eluted with solvents of increasing polarity (*n*-hexane, EtOAc, CH_2_Cl_2_, and MeOH). In total, 12 fractions (V1 to V12) were obtained. Fraction V9 (1.75 g) was subjected to a Sephadex LH-20 column and eluted with 100% MeOH to give eight subfractions (V9-S1 to V9-S8). Subfraction V9-S3 was then submitted to a RP-18 vacuum liquid chromatography column and eluted with 10% aqueous MeOH - 100% MeOH to yield 6 subfractions (V9-S3-RP1 to V9-S3-RP6). Subfraction V9-S3-RP4 was purified by semi-preparative HPLC using a gradient of MeCN and H_2_O (15:85 to 50:50) containing 0.1% HCOOH to give **3** (2.1 mg). Subfraction V9-S3-RP5 was purified by semi-preparative HPLC using a gradient of MeCN and H_2_O (30:70 to 50:50) containing 0.1% HCOOH to give **1** (6.1 mg). Fraction V10 (0.83 g) was subjected to a Sephadex LH-20 column and eluted with 100% MeOH to give six subfractions (V10-S1 to V10-S6). Subfraction V10-S3 was then purified by semi-preparative HPLC using a gradient of MeCN and H_2_O (40:60) containing 0.1% HCOOH to give **2** (6.7 mg).

Aplospojaveedin A (**1**): Colorless crystal; [*α*]^24^_D_ -102 (*c* 0.25, MeOH); UV (MeOH) *λ*_max_ 208, 255 and 317 nm; ^1^H and ^13^C NMR data, see Table 1; Supplementary Table S1; HRESIMS [M + H]^+^ *m/z* 502.2366 (calcd for C_26_H_36_N_3_O_5_S 502.2370).

**Table 1:** ^1^H and ^13^C NMR data of compounds 1–3 in DMSO-*d*_6_.

No.	1^a^		2^b^		3^c^	
	*δ*_C_, type	*δ*_H_ (*J* in Hz)	*δ*_C_, type	*δ*_H_ (*J* in Hz)	*δ*_C_, type	*δ*_H_ (*J* in Hz)
1	132.3, CH	5.54, br d (9.8)	132.0, CH	5.52, br d (9.8)	132.0, CH	5.52, br d (9.8)
2	128.1, CH	5.32, ddd (9.8, 4.2, 2.8)	128.1, CH	5.31, ddd (9.8, 4.1, 2.8)	128.3, CH	5.32, ddd (9.8, 4.2, 2.8)
3	46.6, CH	3.14, ddt (6.8, 4.2, 2.4)	46.0, CH	3.41, ddt (7.0, 4.1, 2.5)	46.4, CH	3.15, ddt (6.8, 4.2, 2.4)
4	51.7, CH	3.82, dd (11.8, 6.8)	51.8, CH	3.64, dd (12.0, 7.0)	51.0, CH	3.73, dd (12.0, 6.8)
5	36.2, CH	1.50, qd (11.7, 2.5)	36.5, CH	1.46, m	36.1, CH	1.51, qd (11.5, 2.9)
6	29.8, CH_2_	1.75, m 0.68, qd (12.2, 3.1)	29.5, CH_2_	1.60, m 0.66, qd (12.3, 3,3)	29.9, CH_2_	1.82, dq (12.5, 3.1) 0.69, qd (12.5, 3.0)
7	35.1, CH_2_	1.64, br d (12.9) 0.92, qd (12.9, 3.5)	35.0, CH_2_	1.62, m 0.90, qd (12.7, 3.4)	35.1, CH_2_	1.65, br d (12.8) 0.93, qd (12.8, 3.7)
8	32.7, CH	1.45, m	32.5, CH	1.44, m	32.6, CH	1.46, m
9	41.5, CH_2_	1.74, m 0.76, q (12.1)	41.5, CH_2_	1.74, br d (12.2) 0.75, q (12.2)	41.6, CH_2_	1.75, br d (12.2) 0.77, q (12.2)
10	40.7, CH	1.69, br t (11.9)	40.4, CH	1.68, br t (11.9)	40.7, CH	1.69, br t (11.8)
11	22.5, CH_3_	0.87, d (6.5)	22.4, CH_3_	0.87, d (6.5)	22.4, CH_3_	0.88, d (6.5)
12	135.3, C		136.1, C		136.0, C	
13	122.5, CH	5.03, q (6.6)	121.6, CH	4.99, q (6.7)	121.4, CH	4.93, q (7.0)
14	14.1, CH_3_	1.43, d (6.6)	13.3, CH_3_	1.40, d (6.7)	13.4, CH_3_	1.36, d (7.0)
15	15.4, CH_3_	1.36, s	15.4, CH_3_	1.34, s	15.3, CH_3_	1.37, s
16	196.6, C		196.7, C		197.8, C	
17	123.0, C		124.7, C		99.8, C	
18	174.9, C		172.5, C		171.3, C	
19	71.2, C		70.7, C		60.4, C	
20	NH	8.66, s	NH	8.61, s	NH	8.14, s
21	167.0, C		166.4, C		168.8, C	
22	29.3, CH_3_	1.52, s	29.6, CH_3_	1.58, s	29.4, CH_3_	1.78, s
23	NH		NH		NH	9.71, d (5.3)
24	53.2, CH	3.44, dd (11.5, 5.6)	51.7,CH	3.99, dd (4.9, 3.8)	51.1,CH	4.53, ddd (12.6, 5.3, 4.4)
25	26.1, CH_2_	3.08, dd (14.4, 11.5) 3.05, dd (14.4, 5.6)	27.0, CH_2_	3.34, dd (13.7, 4.9) 3.08, dd (13.7, 3.8)	29.2, CH_2_	3.35, dd (11.0, 4.4) 2.52, dd (12.6, 11.0)
26	171.9, C		171.2, C		167.4, C	
27	NH	8.27, t (5.7)	NH	8.05, dd (5.8, 5.2)	NH	8.51, t (6.0)
28	41.2, CH_2_	3.80, d (5.7)	41.1, CH_2_	3.76, dd (17.8, 5.8) 3.62, dd (17.8, 5.2)	42.4, CH_2_	3.72, d (6.0)
29	171.1, C		170.6, C		170.5, C	

^a^Recorded at 800 MHz (^1^H) and 200 MHz (^13^C). ^b^Recorded at 600 MHz (^1^H) and 150 MHz (^13^C). ^c^Recorded at 750 MHz (^1^H) and 188 MHz (^13^C).

Aplospojaveedin B (**2**): Colorless oil; [*α*]^24^_D_ -62 (*c* 0.25, MeOH); UV (MeOH) *λ*_max_ 206, 265 and 321 nm; ^1^H and ^13^C NMR data, see Table 1; Supplementary Table S1; HRESIMS [M + H]^+^ *m/z* 502.2363 (calcd for C_26_H_36_N_3_O_5_S 502.2370).

Aplospojaveedin C (**3**): Colorless oil; [*α*]^24^_D_ -83 (*c* 0.25, MeOH); UV (MeOH) *λ*_max_ 203, 240 and 301 nm; ^1^H and ^13^C NMR data, see Table 1; Supplementary Table S1; HRESIMS [M + H]^+^ *m/z* 502.2370 (calcd for C_26_H_36_N_3_O_5_S 502.2370).

### X-ray crystallographic analysis of aplospojaveedin A (1)

Suitable single-crystals of **1** were carefully selected under a polarizing microscope and mounted on a loop. Data collection: Kappa APEX2 Duo CCD diffractometer with a microfocus source and multi-layer mirror monochromator for Cu–Kα radiation (*λ* = 1.54178 Å) at 140(2) K; *ω*-scans. Data collection and cell refinement with APEX2 (2006), data reduction with SAINT (Bruker) (SAINT, 2006). The structures were solved by direct methods (SHELXT-2015) (Sheldrick, 2008), refinement was done by full-matrix least squares on *F*^2^ using the SHELXL-2017 program suite (Sheldrick, 2008), empirical (multi-scan) absorption correction with SADABS (Bruker) (Sheldrick, 1996). All non-hydrogen atom positions were refined with anisotropic temperature factors. Hydrogen atoms for aromatic CH, aliphatic CH, CH_2_ and CH_3_ groups were positioned geometrically (C–H = 0.95 Å for aromatic and olefinic CH, 1.00 Å for aliphatic CH, 0.99 for CH_2_ and 0.98 Å for CH_3_,) and refined using a riding model (AFIX 43 for aromatic CH, AFIX 13 for aliphatic CH, AFIX 23 for CH_2_ and AFIX 137 for CH_3_), with U_iso_(H) = 1.2U_eq_(CH, CH_2_) and U_iso_(H) = 1.5U_eq_(CH_3_). The protic hydrogen atoms for the OH and NH group were found and refined freely with U_iso_(H) = 1.5U_eq_(O,N). Graphics were drawn with DIAMOND (Version 4) (Brandenburg, 2023). The structural data for this paper has been deposited with the Cambridge Crystallographic Data Center (CCDC-number 2295373 for **1**). These data can be obtained free of charge via www.ccdc.cam.ac.uk/data_request/cif.

### ECD and NMR calculations

Mixed torsional/low-mode conformational searches were carried out by means of the Macromodel 10.8.011 software using the Merck Molecular Force Field (MMFF) with an implicit solvent model for CHCl_3_ applying a 21 kJ/mol energy window (MacroModel, 2015). Geometry re-optimizations of the resultant conformers [B3LYP/6–31 + G(d,p) level *in vacuo* and ωB97X/TZVP PCM/MeOH], DFT-NMR [mPW1PW91/6–311 + G(2d,p)] and TDDFT-ECD calculations [B3LYP/TZVP, BH&HLYP/TZVP, CAM-B3LYP/TZVP and PBE0/TZVP all with PCM/MeOH] were performed with the Gaussian 09 package (Frisch et al., 2013). Computed NMR shift data were corrected with I = 185.6277 and S = −1.0175 for MeOH and I = 185.2853 and S = −1.0267 for DMSO (CHESHIRE CCAT, 2019; Pierens, 2014). ECD spectra were generated as sums of Gaussians with 3,000 cm^−1^ widths at half height, using dipole-velocity-computed rotational strength values (Stephens and Harada, 2010). Boltzmann distributions were estimated from the B3LYP and the ωB97X energies. Visualization of the results was performed by the MOLEKEL software package (Varetto, 2009).

### Ligand-based target prediction

Potential new targets were predicted based on the similarity to ligands already known to bind to those targets using the SwissTargetPrediction^1^ and the SuperPred webserver^2^ using default parameters. Targets, monomers and complexes, predicted by both webservers were subsequently extracted and used for further investigation.

### Blind docking to predicted targets

For the molecular docking, the three compounds **1**–**3** were drawn and converted into a 3D structure with Maestro in a protonated and deprotonated form (Maestro, 2020). The protonated and deprotonated compounds were subsequently docked over the whole structure of all predicted targets utilizing a combination of AutoDock (Goodsell et al., 1996) as a docking engine and the DrugScore2018 distance-dependent pair-potentials (Dittrich et al., 2019) as an objective function. Experimentally resolved structures of the potential targets were preferred unless domains were missing or the resolution of a complete experimental structure was >3.1 Å, more than double that of a C-C bond length (Lide, 2004). The experimental structures with PDB IDs 1PIN (Ranganathan et al., 1997), 4PXZ (Zhang et al., 2014), 5F1A (Lucido et al., 2016), 7CX2 (Qu et al., 2021), 7F5N (Singh et al., 2021), 7NWL (Schumacher et al., 2021), 7VDP (Daniels et al., 2022), 7VFX (Chen et al., 2022), 7VL9 (Shao et al., 2022), 8GUR (Li et al., 2023), and 8HDO (Cai et al., 2022) were used for the predicted monomers, and the structures 1UNL (Mapelli et al., 2005), 1 W98 (Honda et al., 2005), 6UJA (Campbell et al., 2020), and 7NWL (Schumacher et al., 2021), were used for the predicted complexes. All co-crystallized proteins that were not the target and all other molecules except divalent ions in integrins were deleted from the structures prior to docking. For all other targets, the 4^th^ version of their AlphaFold2 (Jumper et al., 2021) model was used. During docking, default parameters were used, except for the RMSD cutoff used in clustering, which was set to 2.0 Å (Ranganathan et al., 1997; Sotriffer et al., 2002; Lide, 2004). Binding modes were considered valid if they were located in the binding pocket and contained in the largest cluster, which comprised at least 20% of all docking poses. To gain a finer overview of potential binding poses, all binding poses of each ligand were also visually evaluated in Pymol (Schrödinger, 2010).

## Results and discussion

**Figure 2:**
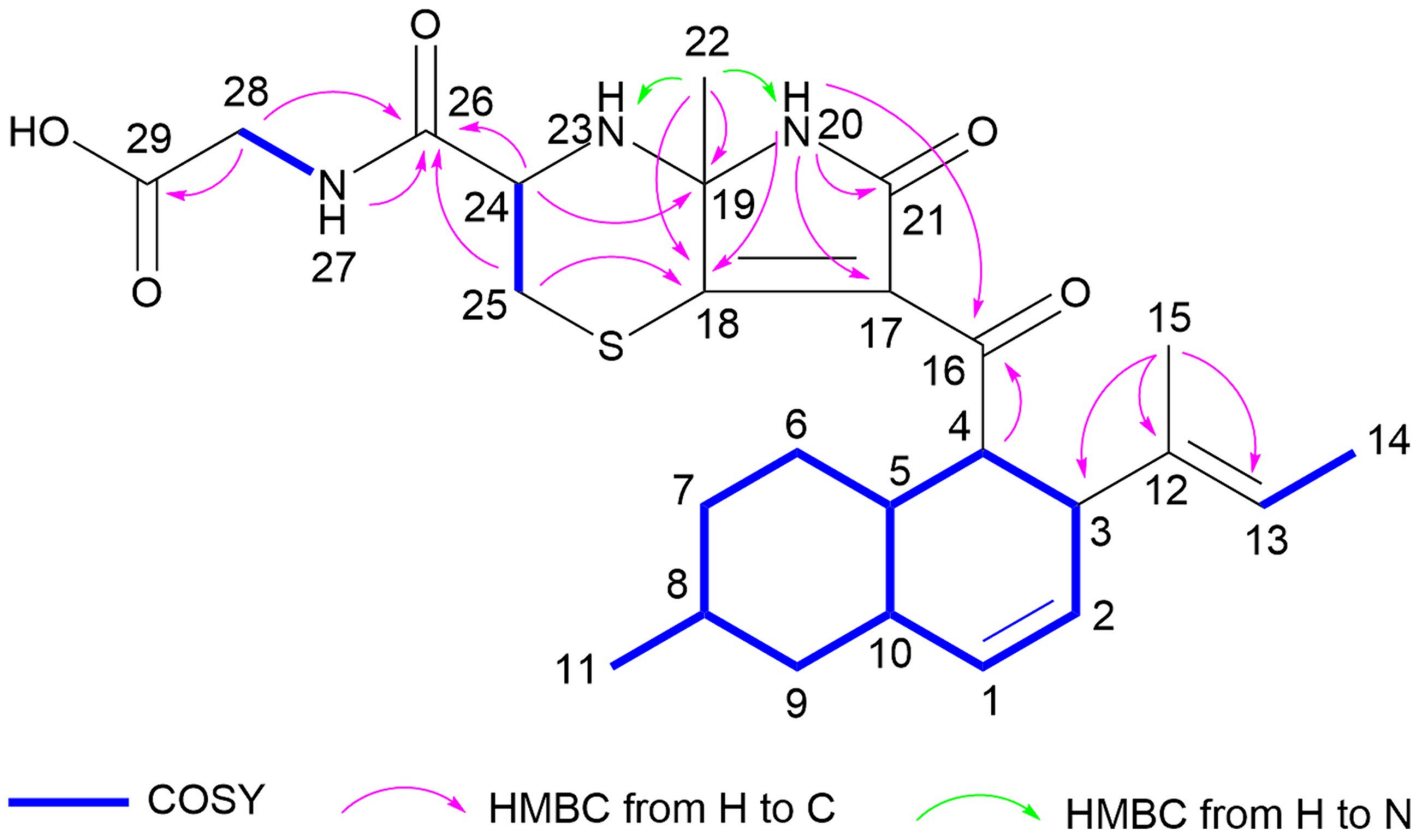
Key COSY and HMBC correlations of compound 1.

Aplospojaveedins A (**1**) was obtained as colorless crystals, with UV absorptions at λ_max_ 208, 255 and 317 nm. Its molecular formula was established as C_26_H_35_O_5_N_3_S on the basis of a prominent pseudomolecular ion peak at *m/z* 502.2366 [M + H]^+^ in the HRESIMS spectrum, indicating 11 degrees of unsaturation. The NMR spectra of **1** were recorded in DMSO-*d*_6_ (Table 1) and in CD_3_OD (Supplementary Table S1). The former solvent revealed two nitrogen-bearing protons at *δ*_H_ 8.66 (s, NH-20) and 8.27 (t, NH-27), three olefinic protons at *δ*_H_ 5.54 (br d, H-1), 5.32 (ddd, H-2), and 5.03 (q, H-13), as well as four methyl signals at *δ*_H_ 1.52 (Me-22), 1.43 (d, Me-14), 1.36 (s, Me-15) and 0.87 (d, Me-11). The ^13^C NMR spectrum of **1** displayed four carbonyl carbons at *δ*_C_ 196.6 (C-16), 171.9 (C-26), 171.1 (C-29), 167.0 (C-21) and six olefinic carbons at *δ*_C_ 174.9 (C-18), 135.3 (C-12), 132.3 (C-1), 128.1 (C-2), 123.0 (C-17), and 122.5 (C-13), accounting for seven degrees of unsaturation. Thus, compound **1** was suggested to bear a tetracyclic ring skeleton. In COSY spectrum (Figure 2), the correlations of H-1/H-2/H-3/H-4/H-5/H_2_-6/H_2_-7/H-8/H_2_-9/H-10, and between H-5/H-10, H-8/Me-11, indicated the existence of an octahydronaphthalene ring with a double bond at C-1/C-2 and a methyl group at C-8. The COSY correlation between H-13 and Me-14, together with the HMBC correlations from Me-15 to C-12, C-13 and C-3, confirmed the attachment of a but-2-en-2-yl group at C-3. Furthermore, the HMBC correlations from NH-20 to C-16, C-17, C-18, C-19, C-21 and from H-4 to C-16 indicated the presence of an *α*, *β*-unsaturated lactam and its linkage with the above mentioned octahydronaphthalene ring via carbonyl group C-16. According to the molecular formula, two additional nitrogen atoms and a sulfur atom must reside in the remaining substructure. The presence of a glycine-cysteine moiety was supported by the COSY correlations between NH-27/H_2_-28 (*δ*_H_ 3.80), H-24/H_2_-25, together with the HMBC correlations from H_2_-28 to C-29, and from H-24, H_2_-25, NH-27 and H_2_-28 to C-26. Moreover, the HMBC correlations from H-24 to C-19, from H_2_-25 to C-18, and from Me-22 to C-18 and C-19, along with the HMBC correlations from Me-22 to N-20 and N-23 in ^1^H-^15^N-HMBC confirmed the linkage between C-19/N-23 and C-18/S-25 to form the fourth ring in **1**. Thus, the planar structure of **1** was elucidated as shown.

Compound **1** contains two isolated blocks of chirality: an octahydronaphthalene and a tetrahydropyrrolo [3,2-b][1,4] thiazin-6(2*H*)-one moiety with five and two chirality centers, respectively. The relative configurations in the isolated blocks were established by ROESY but the two blocks could not be correlated. An *E* configuration was assigned for the double bond between C-12 and C-13 due to the ROESY correlation between H-3 and H-13. The ROESY relationships between H-3/H-4, H-4/H-6b (*δ*_H_ 0.68), H-6b/H-10, H-10/H-4, H-10/H-8 suggested that these protons are present on the same face of the octahydronaphthalene moiety whereas the ROESY correlations between H-5 and H-6a (*δ*_H_ 1.75) established that these latter protons are on the opposite face of the ring. In the heterocyclic moiety, ROESY correlations between Me-22/H-25a (*δ*_H_ 3.08), H-24/H-25b (*δ*_H_ 3.05) as well as the coupling constants between H-24/H-25ab (^2^*J*_H-24/H-25a_ = 11.5 Hz, ^2^*J*_H-24/H-25b_ = 5.6 Hz) suggested that H-24 adopted axial orientation and Me-22 and H-24 are on opposite faces of the ring (Supplementary Figure S60).

**Figure 3:**
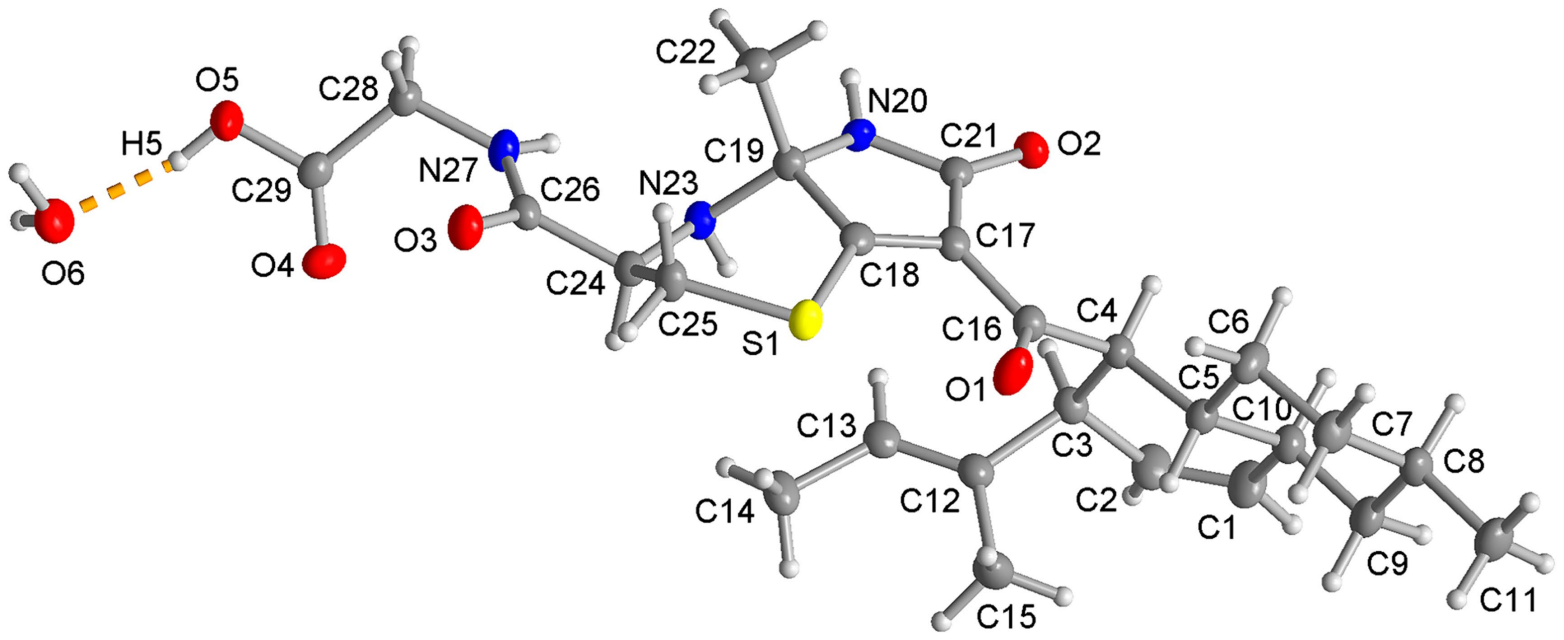
Molecular structure of compound 1 from the single crystal X-ray structure. (50% thermal ellipsoids, H atoms with arbitrary radii. The atom numbering is the same as in Figure 1. Compound 1 crystallizes with a water molecule, shown here with the hydrogen bond from the carboxyl group as a dashed orange line).

To determine the absolute configuration, compound **1** was crystallized in methanol, and a single-crystal X-ray diffraction analysis was performed. Compound **1** crystallizes in the non-centrosymmetric orthorhombic space group (necessary for a chiral molecule) *P*2_1_2_1_2_1_.The absolute configuration of compound 1 is (3*R*, 4*S*, 5*R*, 8*R*, 10*S*, 19*R*, 24*R*) (Figure 3). The asymmetric unit contains a molecule of **1** together with a water molecule of crystallization. In the solid state the O-H and N-H groups are engaged in inter-molecular hydrogen bonding (Supplementary Figure S1; Supplementary Table S5).

To elucidate the absolute configuration of 1 independently and test the combination of TDDFT-ECD (Superchi et al., 2018; Mándi and Kurtán, 2019) and DFT-NMR methods to assign isolated blocks of chirality (Li et al., 2020; Kovács et al., 2023), MMFF conformational searches were applied on stereoisomers (3*R*,4*S*,5*R*,8*R*,10*S*,19*R*,24*R*)-**1**, (3*R*,4*S*,5*R*,8*R*,10*S*,19*R*,24*S*)-**1**, (3*R*,4*S*,5*R*,8*R*,10*S*,19*S*,24*R*-**1**) and (3*R*,4*S*,5*R*,8*R*,10*S*,19*S*,24*S*)-**1**. The computed 199, 126, 99 and 154 initial conformers were re-optimized at the B3LYP/6–31 + G(d,p) and the ωB97X/TZVP PCM/MeOH levels. ^13^C NMR chemical shift data computed for the low-energy (≥ 1% Boltzmann populated) 7, 9, 10 and 7 B3LYP conformers were corrected for DMSO and MeOH and then compared with the experimental ^13^C NMR chemical shifts (Supplementary Tables S6, S7). The DMSO results showed better agreement for the (3*R*,4*S*,5*R*,8*R*,10*S*,19*S*,24*S*) and (3*R*,4*S*,5*R*,8*R*,10*S*,19*R*,24*R*) stereoisomers than for the other two, which confirmed *cis-*(19R*,24R*) relative configuration of the C-19 and C-24 substituents but the two isolated blocks could not be correlated. It is noteworthy that the presence of the sulfur heavy atom in the vicinity of chirality centers and the coordinating DMSO or MeOH solvent molecules can have a relatively large impact on the chemical shifts (Sikandar et al., 2023).

**Figure 4:**
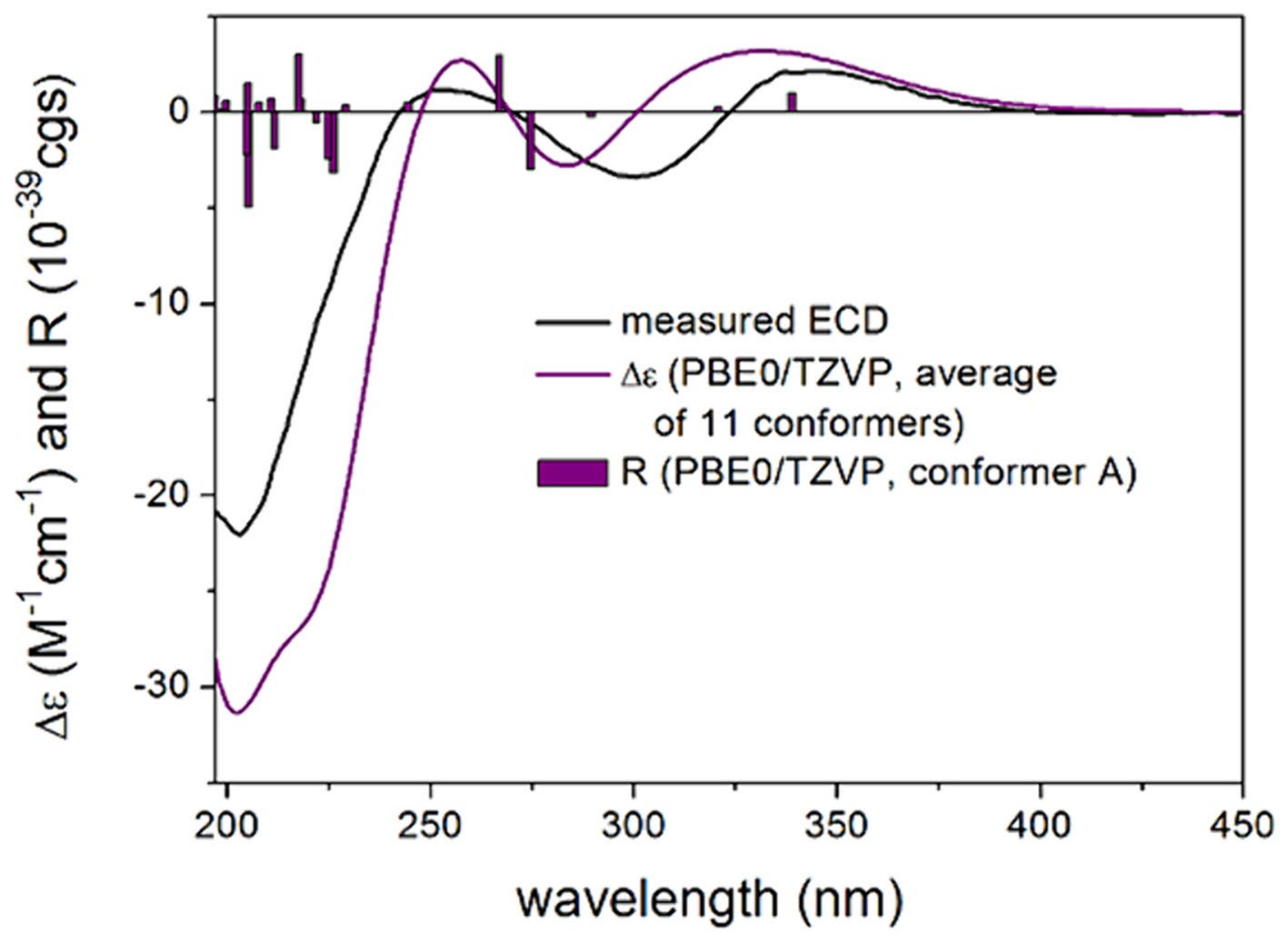
Experimental ECD spectrum of compound 1 in MeOH (black line) compared with the calculated PBE0/TZVP PCM/MeOH spectrum of (3*R*,4*S*,5*R*,8*R*,10*S*,19*R*,24*R*)-1. Level of DFT optimization: ωB97X/TZVP PCM/MeOH. Bars represent the rotational strength values of conformer A.

TDDFT-ECD calculations were performed at various levels of theory on the low-energy (≥ 1% Boltzmann population) 11, 9, 5 and 11 ωB97X conformers of the four stereoisomers, which indicated that the ECD spectrum was governed by the C-19 chirality center above 240 nm. The (3*R*,4*S*,5*R*,8*R*,10*S*,19*R*,24*R*) and (3*R*,4*S*,5*R*,8*R*,10*S*,19*R*,24*S*) stereoisomers reproduced equally well the experimental ECD spectrum of **1**, while the (3*R*,4*S*,5*R*,8*R*,10*S*,19*S*,24*R*) and (3*R*,4*S*,5*R*,8*R*,10*S*,19*S*,24*S*) stereoisomers gave a mismatch in this region (Figure 4; Supplementary Figures S2–S4). Below 240 nm, all four computed stereoisomers reproduced the intense negative Cotton effect (CE), which suggested that this region was determined by the (4*S*) chirality center of the octahydronaphthalene moiety. Thus, ECD transitions above 240 nm reported the AC of C-19 chirality center of the heterocyclic scaffold, while the C-4 chirality center of the octahydronaphthalene moiety was reflected in the intense CE below 240 nm. Thus, combined with the known relative configuration of the isolated blocks, TDDFT-ECD calculations allowed determining the absolute configuration of **1** as (3*R*,4*S*,5*R*,8*R*, 10*S*,19*R*,24*R*), which corroborates the result of X-ray analysis.

Compound **2** shared the same molecular formula as **1** as deduced from HRESIMS. The planar structure of **2** was confirmed to be identical to that of **1** after detailed analysis of the 2D NMR spectra of **2**. The obvious differences of NMR data between **1** and **2** were the chemical shifts for H-24 and H-25a which differed by +0.55 and + 0.26 ppm in **2** when compared to **1**. Further differences were observed with regard to the coupling constants between H-24 and H-25ab, suggesting a different configuration of **2** compared to **1** in the nitrogen containing moiety. In the ROESY spectrum of **2**, the correlations from Me-22 (*δ*_H_ 1.58) to H-24 (*δ*_H_ 3.99) and H-25*α* (*δ*_H_ 3.34) indicated that they were present on the same face of the ring, whereas Me-22 and H-24 were on the opposite side of the ring in **1** (Supplementary Figure S61).

**Figure 5:**
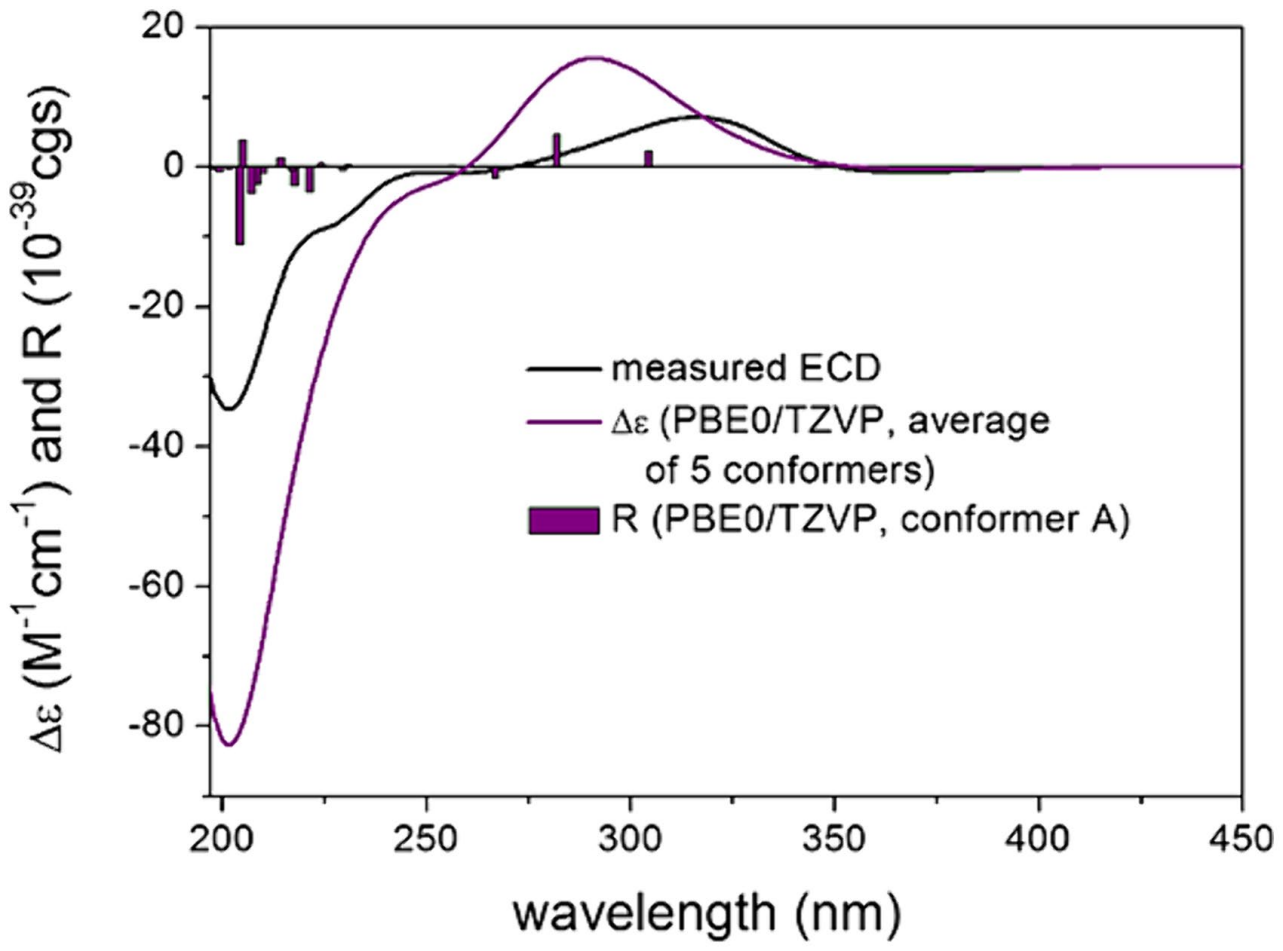
Experimental ECD spectrum of compound 2 in MeOH (black line) compared with the calculated PBE0/TZVP PCM/MeOH spectrum of (3*R*,4*S*,5*R*,8*R*,10*S*,19*S*,24*R*)-2. Level of DFT optimization: ωB97X/TZVP PCM/MeOH. Bars represent the rotational strength values of conformer A.

For the configurational assignment of **2**, the computed ^13^C DFT-NMR and TDDFT-ECD data of four stereoisomers of **2** were compared with the experimental data. The ^13^C DFT-NMR results compared to the experimental data of **2** showed a preference for the *cis-*(19R*,24R*) relative configuration over the *trans-*(19S*,24R*) one, which contradicted the experimental ROE results (Supplementary Tables S8, S9). Above 260 nm, a broad positive ECD band was measured at about 310 nm, which was reproduced well by the computed TDDFT-ECD of the stereoisomers (3*R*,4*S*,5*R*,8*R*,10*S*,19*S*,24*R*)-**2** and (3*R*,4*S*,5*R*,8*R*,10*S*,19*S*,24*S*)-**2**, while the stereoisomers (3*R*,4*S*,5*R*,8*R*,10*S*,19*R*,24*R*)-**2** and (3*R*,4*S*,5*R*,8*R*,10*S*,19*R*,24*S*)-**2** gave a mismatch, allowing elucidation of the (19*S*) absolute configuration. All four stereoisomers reproduced the negative ECD transitions below 260 nm, which confirmed that the C-4 chirality center and hence the octahydronaphthalene moiety had the same absolute configuration as that of **1** (Figure 5; Supplementary Figures S5–S7). The negative highest wavelength n-π* transition above 350 nm could be well estimated by the (3*R*,4*S*,5*R*,8*R*,10*S*,19*S*,24*R*)-**2**, while it could not be reproduced by the stereoisomer (3*R*,4*S*,5*R*,8*R*,10*S*,19*S*,24*S*)-**2** in accordance with the experimental ROE results. The combination of TDDFT-ECD calculations and experimental ROE data determined the absolute configuration of **2** as (3*R*,4*S*,5*R*,8*R*,10*S*,19*S*,24*R*).

The molecular formula of **3** was the same as for compounds **1** and **2** based on the HRESIMS data. The ^1^H and ^13^C NMR data of the octahydronaphthalene ring in **1**, **2** and **3** were likewise very similar, suggesting that they shared the same substructure. However, significant differences were found for chemical shifts for C-17 and C-19 (−24.9 and − 10.3 ppm, respectively) in **3** when compared to **2**. In addition, H-25ab showed HMBC correlations to C-19 in **3** instead of to C-18 as in compounds **1** and **2**, indicating exchange of the positions of S and NH in **3**. Thus, the planar structure of **3** was elucidated as shown. The ROESY relationship between Me-22 (*δ*_H_ 1.78) and H-24 (*δ*_H_ 4.53) suggested they were on the same side of the ring (Supplementary Figure S62).

**Figure 6:**
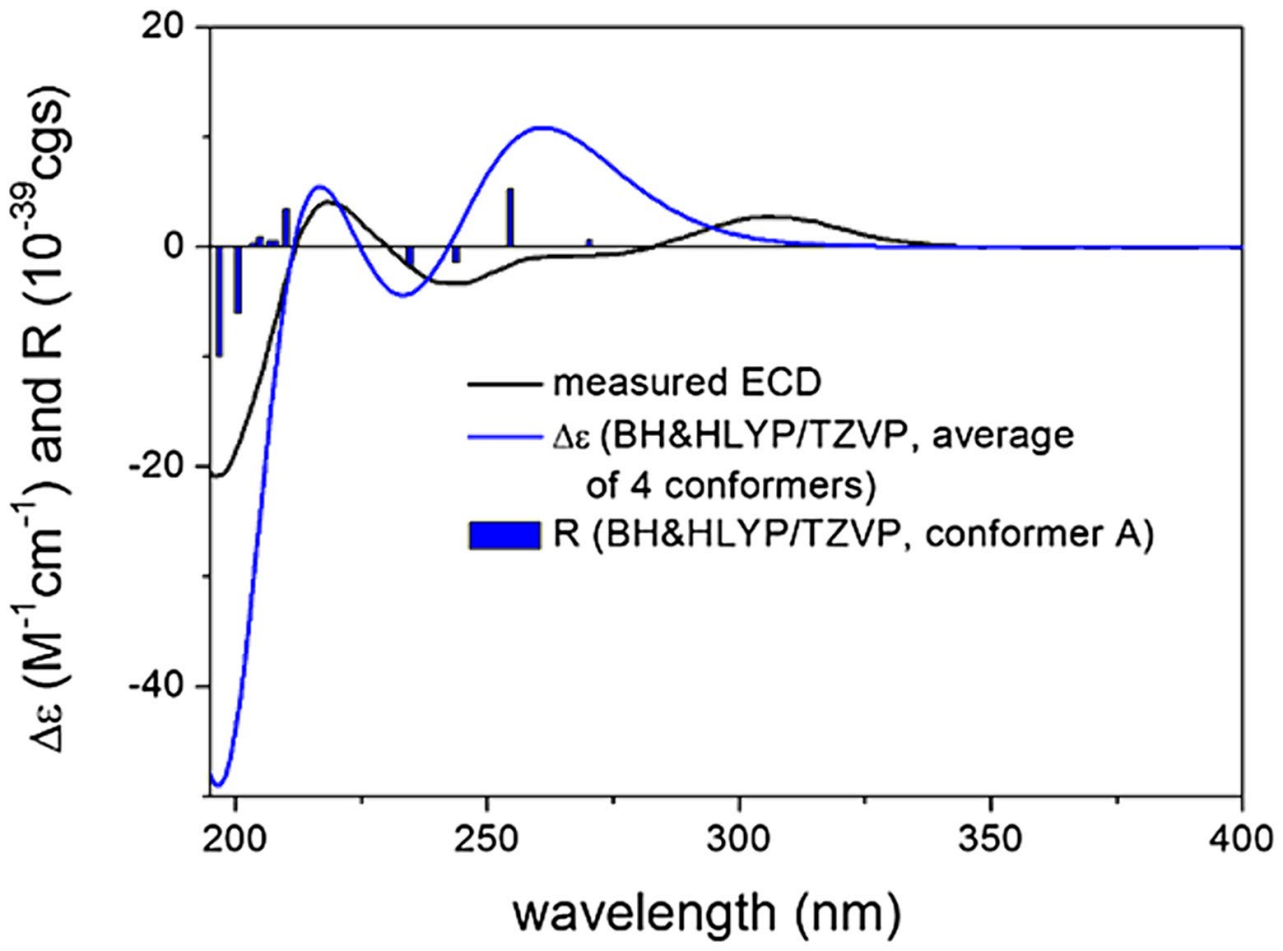
Experimental ECD spectrum of compound 3 in MeOH (black line) compared with the calculated BH&HLYP/TZVP PCM/MeOH spectrum of (3*R*,4*S*,5*R*,8*R*,10*S*,19*S*,24*R*)-3. Level of DFT optimization: ωB97X/TZVP PCM/MeOH. Bars represent the rotational strength values of conformer A.

The above DFT-NMR and TDDFT-ECD protocols were performed on four stereoisomers of **3** by changing the configuration of the C-19 and C-24 chirality centers; (3*R*,4*S*,5*R*,8*R*,10*S*,19*R*,24*R*)-**3**, (3*R*,4*S*,5*R*,8*R*,10*S*,19*R*,24*S*)-**3**, (3*R*,4*S*,5*R*,8*R*,10*S*,19*S*,24*R*) -**3** and (3*R*,4*S*,5*R*,8*R*,10*S*,19*S*,24*S*)-**3**. The comparison of the computed and experimental ^13^C NMR chemical shifts showed a clear preference for the *trans-*(19S*,24R*) relative configuration of the heterocyclic moiety in line with the experimental ROE results (Supplementary Tables S10, S11). TDDFT-ECD calculations of the stereoisomers (3*R*,4*S*,5*R*,8*R*,10*S*,19*S*,24*R*)-**3** and (3*R*,4*S*,5*R*,8*R*,10*S*,19*S*,24*S*)-**3** gave acceptable agreement with the experimental ECD spectrum of **3**, while stereoisomers (3*R*,4*S*,5*R*,8*R*,10*S*,19*R*,24*R*)-**3** and (3*R*,4*S*,5*R*,8*R*,10*S*,19*R*,24*S*)-**3** gave a mismatch (Figure 6; Supplementary Figures S8–S10). The combination of experimental ROE data, ^13^C DFT-NMR and TDDFT-ECD calculations allowed determining the absolute configuration of **3** as (3*R*,4*S*,5*R*,8*R*,10*S*,19*S*,24*R*).

Compounds **1**–**3** show limited structural similarities to known fungal constituents including equisetin (Phillips et al., 1989), phomasetin (Singh et al., 1998), and Ascosalipyrrolidinone A (Osterhage et al., 2000). However, in compounds **1**–**3**, the substituents on the octahydronaphthalene moiety are different from those present in known compounds. Furthermore, **1**–**3** show significant differences in the alkaloid part, implying that the former compounds feature a distinctive novel skeleton. A plausible biogenetic pathway for the formation of **1**–**3** involves iterative hybrid polyketide synthases–non ribosomal peptide synthetases (PKS–NRPS) with the following key steps (Supplementary Figure S59), which can be partially referenced from the known biogenetic pathway of equisetin (Fisch, 2013). Intermediate B was built from an octaketide and an alanine moiety. The beta carbon of the lactam ring in intermediate B was attacked either by *S* atom of the cysteine moiety to form **1** and **2** or by *N* atom of the cysteine moiety to give **3**.

To identify potential targets for the three compounds **1**–**3**, we used the target prediction software packages SwissTargetPrediction (Daina et al., 2019) and SuperPred (Nickel et al., 2014). Targets identically predicted by both approaches (Supplementary Tables S2–S4) were subjected to blind docking experiments individually (“inverse docking”). These probe ligand binding across the whole receptor to unravel whether potential binding sites of compounds **1**–**3** are similar to known binding sites.

**Figure 7:**
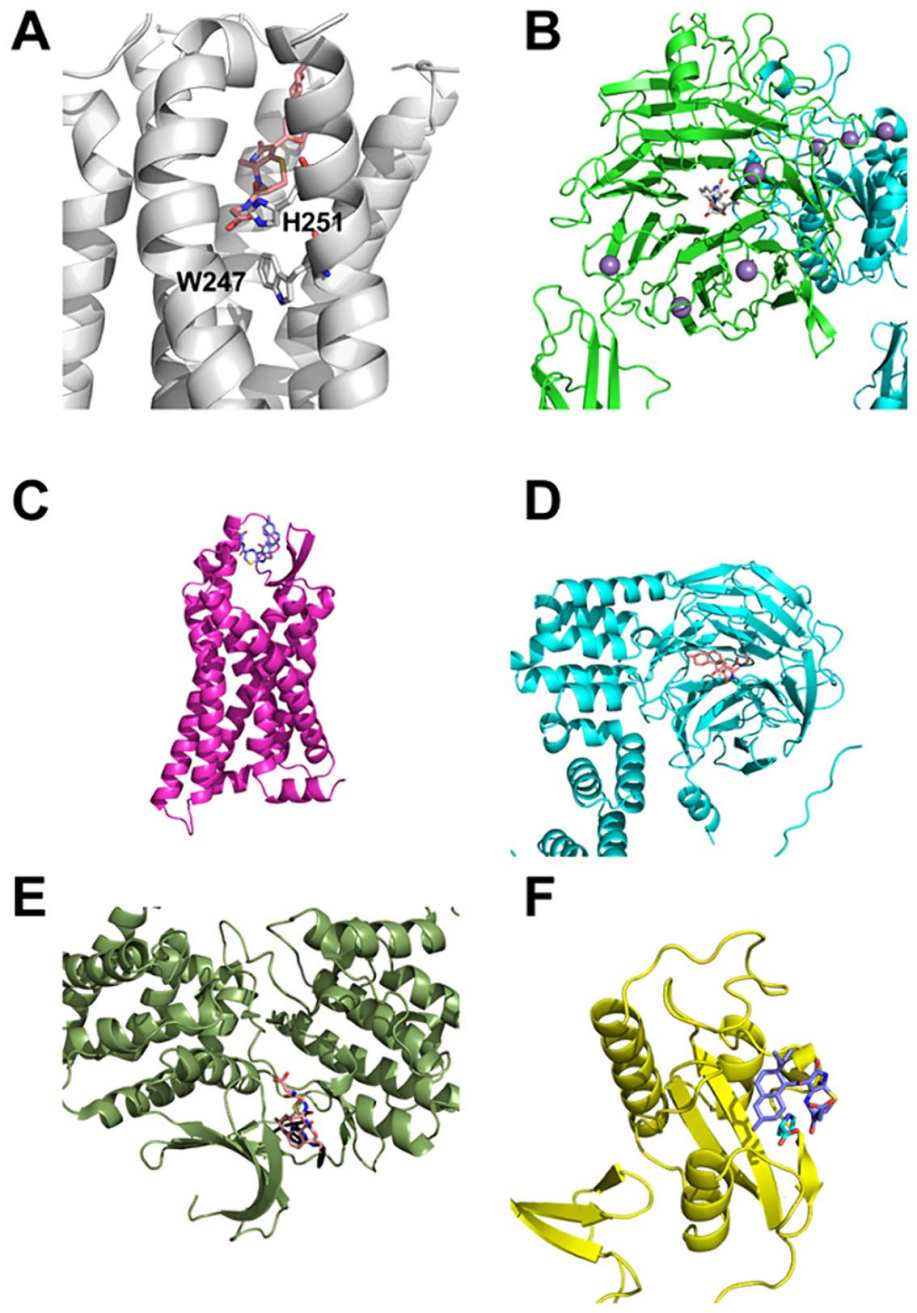
Selected, biologically putatively relevant, predicted binding modes of compound 1 (salmon), 2 (gray), and 3 (navy) in predicted targets. **(A)** Binding mode of compound 1 in the adenosine receptor A_2B_ (white). TMs 3 and 6 are addressed by the compound, which forms a salt bridge to H251, located in a position important for the activation of GPCRs above the functionally relevant W247. **(B)** Binding mode of compound 2 in the integrin *α*_5_*β*_1_ complex (green and blue, respectively). The compound binds to the *β*-propeller domain of integrin *α*_5_. **(C)** Binding mode of compound 3 at the EL2 + 3 of the formyl peptide receptor 1 (pink). This binding mode may convey an allosteric effect. **(D)** Binding mode of compound 1 in the *β*-propeller-like domain of the Kelch-like ECH-associated protein 1 (blue). **(E)** Binding mode of compound 1 at the binding site of *R*-roscovitine (shown in addition in black) in the cyclin-dependent kinase 5 (forest green). **(F)** Binding mode of compound 3 in the proline (shown in addition in cyan) binding site of the mitotic rotamase Pin1 (yellow).

The blind docking revealed a high propensity of the compounds to bind to two different sites: The orthosteric binding site of G-protein coupled receptors (GPCRs) and domains similar to the *β*-propeller domain found in integrins (Figures 7A,B) (Campbell and Humphries, 2011). A few binding poses in the orthosteric binding pocket in the GPCR adenosine receptor A_2B_ and the cannabinoid CB_2_ (Figure 7A) receptor address transmembrane helices (TMs) 3 and 6 and in the chemokine receptor CCR1 TM 6. Such poses, which might be suitable for an agonist (A_2B_, CB_2_) or antagonist (all), were identified for all compounds. However, the number of poses identified in the orthosteric binding pockets was too small to count as valid binding poses (see Methods for criteria). In the GPCRs A_2B_ and formyl peptide receptor 1 (FPR1) (Figure 7C), binding modes at the extracellular loop 2 + 3 (EL2 + 3), a potential allosteric binding site (Peeters et al., 2012), were additionally identified. For the integrin *α*_5_*β*_1_ complex, binding modes of the compounds inside the *β*-propeller (Figure 7B) or the *β*-propeller-like domain of Kelch-like ECH-associated protein 1 were identified (Figure 7D). In the X-ray crystal structure of cyclin-dependent kinase 5 (CDK5), a binding mode of deprotonated compound **1** at the position of the co-crystallized *R*-roscovitine (Figure 7E) was identified. Despite this target being found for compound **3** in the ligand similarity-based screening only, the high similarity to compound **1** still renders CDK5 a viable higher-priority target for compound **1**. In the X-ray crystal structure of mitotic rotamase Pin1 (Peptidyl-prolyl cis-trans isomerase NIMA-interacting 1), a binding mode of deprotonated compound **3** at the position of the co-crystallized proline (Figure 7F) was identified.

We predicted a diverse set of potential targets for compounds **1**–**3** based on a search for chemically and structurally similar ligands. Accordingly, proteins known to bind ligands similar to compounds **1**–**3** were suggested as potential targets for the respective compounds. The set of potential targets is structurally diverse, including GPCRs, integrins, kinases, and histone deacetylases. Despite the high structural similarity of, e.g., the epimeric compounds **1** and **2**, varying targets were identified for all three compounds. To counteract potential biases of the screening methods and to identify targets that are more likely to be influenced by the compounds, a blind docking of all compounds to all potential targets identically identified by both screening methods was conducted (“inverse docking”). The blind docking suggested several putative targets for all three compounds, e.g., the GPCR cannabinoid receptor 2 or the integrin *α*_5_*β*_1_ complex; the predicted compound binding might functionally impact these targets.

Note, however, that computational target prediction (Katsila et al., 2016) and inverse docking (Vasseur et al., 2015) are considered challenging. Further investigations could thus consider compute-intensive molecular simulations of free ligand binding (Gohlke et al., 2013a; Kaiser et al., 2023) to confirm the predicted binding modes, long-time molecular simulations to probe functional effects (Gohlke et al., 2013b), or experimental validation of the putative agonistic or antagonistic activity of compounds **1**–**3** with a higher priority toward those targets.

## Conclusion

In summary, compounds **1**–**3** represent a new family of alkaloids comprising octahydronaphthalene, *α*, *β*-unsaturated lactam and glycine-cysteine moieties. The combination of X-ray diffraction analysis, DFT-NMR, and TDDFT-ECD calculations aided by ROE correlations could efficiently handle the configurational assignment of two isolated blocks of chirality in compounds **1**–**3**. The unusual skeleton of the new compounds that were induced by addition of NaI to solid rice medium during fermentation of the endophytic fungus *Aplosporella javeedii* implies an interesting biosynthetic mechanism that is of value for further investigation.

## Data Availability

The datasets presented in this study can be found in online repositories. The names of the repository/repositories and accession number(s) can be found in the article/Supplementary material.

## Footnotes

^1^http://www.swisstargetprediction.ch/

^2^https://prediction.charite.de/subpages/target_prediction.php

## References

[ref1] APEX2. (2006). *Data collection program for the CCD area-detector system, version 2.1–0, Bruker analytical X-ray systems: Madison, Wisconsin, USA*.

[ref2] Brandenburg K. (2023). Diamond (version 4), crystal and molecular structure visualization, crystal impact – K. Bonn, Germany: Brandenburg and H. Putz Gbr, 2009–2023.

[ref3] Cai H. Xu Y. Guo S. He X. Sun J. Li X.. (2022). Structures of adenosine receptor A_2B_R bound to endogenous and synthetic agonists. Cell Discov. 8:140. doi: 10.1038/s41421-022-00503-1, PMID: 36575181 PMC9794776

[ref4] Campbell M. G. Cormier A. Ito S. Seed R. I. Bondesson A. J. Lou J.. (2020). Cryo-EM reveals integrin-mediated TGF-β activation without release from latent TGF-β. Cell 180:e416, 490–501.e16. doi: 10.1016/j.cell.2019.12.030, PMID: 31955848 PMC7238552

[ref5] Campbell I. D. Humphries M. J. (2011). Integrin structure, activation, and interactions. Cold spring Harb. Perspect. Biol. 3:a004994. doi: 10.1101/cshperspect.a004994, PMID: 21421922 PMC3039929

[ref6] Cao M. Yang D. Adhikari A. Ye F. Zheng C. Yan W.. (2023). Neogrisemycin, a trisulfide-bridged angucycline, produced upon expressing the thioangucycline biosynthetic gene cluster in *Streptomyces albus* J1074. Org. Lett. 25, 961–965. doi: 10.1021/acs.orglett.2c04303, PMID: 36735280 PMC10115141

[ref7] Chen G. Wang X. Liao Q. Ge Y. Jiao H. Chen Q.. (2022). Structural basis for recognition of N-formyl peptides as pathogen-associated molecular patterns. Nat. Commun. 13:5232. doi: 10.1038/s41467-022-32822-y, PMID: 36064945 PMC9445081

[ref8] Chen Y. Xiao T. Guo S. Chang S. Xi X. Su B.. (2024). Unexpected noremestrin with a sulfur-bearing 15-membered macrocyclic lactone from *Emericella* sp. 1454. Org. Lett. 26, 1–5. doi: 10.1021/acs.orglett.3c02958, PMID: 37988124

[ref9] CHESHIRE CCAT. (2019). *The chemical shift repository for computed NMR scaling factors*. Available at: http://cheshirenmr.info/index.htm.

[ref10] Daina A. Michielin O. Zoete V. (2019). SwissTargetPrediction: updated data and new features for efficient prediction of protein targets of small molecules. Nucleic Acids Res. 47, W357–W364. doi: 10.1093/nar/gkz382, PMID: 31106366 PMC6602486

[ref11] Daniels M. H. Malojcic G. Clugston S. L. Williams B. Coeffet-Le Gal M. Pan-Zhou X. R.. (2022). Discovery and optimization of highly selective inhibitors of CDK5. J. Med. Chem. 65, 3575–3596. doi: 10.1021/acs.jmedchem.1c02069, PMID: 35143203

[ref12] Dittrich J. Schmidt D. Pfleger C. Gohlke H. (2019). Converging a knowledge-based scoring function: DrugScore^2018^. J. Chem. Inf. Model. 59, 509–521. doi: 10.1021/acs.jcim.8b00582, PMID: 30513206

[ref13] Fisch K. M. (2013). Biosynthesis of natural products by microbial iterative hybrid PKS–NRPS. RSC Adv. 3, 18228–18247. doi: 10.1039/c3ra42661k

[ref14] Frisch M. J. Trucks G. W. Schlegel H. B. Scuseria G. E. Robb M. A. Cheeseman J. R.. (2013). Gaussian 09, Revision E 01. Wallingford, CT: Gaussian.

[ref15] Gao Y. Stuhldreier F. Schmitt L. Wesselborg S. Guo Z. Zou K.. (2020c). Induction of new lactam derivatives from the endophytic fungus *Aplosporella javeedii* through an OSMAC approach. Front. Microbiol. 11:600983. doi: 10.3389/fmicb.2020.600983, PMID: 33250887 PMC7672018

[ref16] Gao Y. Stuhldreier F. Schmitt L. Wesselborg S. Wang L. Müller W. E. G.. (2020b). Sesterterpenes and macrolide derivatives from the endophytic fungus *Aplosporella javeedii*. Fitoterapia 146:104652. doi: 10.1016/j.fitote.2020.104652, PMID: 32512149

[ref17] Gao Y. Wang L. Kalscheuer R. Liu Z. Proksch P. (2020a). Antifungal polyketide derivatives from the endophytic fungus *Aplosporella javeedii*. Bioorg. Med. Chem. 28:115456. doi: 10.1016/j.bmc.2020.115456, PMID: 32238320

[ref18] Gohlke H. Hergert U. Meyer T. Mulnaes D. Grieshaber M. K. Smits S. H.. (2013a). Binding region of alanopine dehydrogenase predicted by unbiased molecular dynamics simulations of ligand diffusion. J. Chem. Inf. Model. 53, 2493–2498. doi: 10.1021/ci400370y, PMID: 24066861

[ref19] Gohlke H. Schmitz B. Sommerfeld A. Reinehr R. Häussinger D. (2013b). α_5_β_1_-integrins are sensors for tauroursodeoxycholic acid in hepatocytes. Hepatology 57, 1117–1129. doi: 10.1002/hep.25992, PMID: 22865233

[ref20] Goodsell D. S. Morris G. M. Olson A. J. (1996). Automated docking of flexible ligands: applications of AutoDock. J. Mol. Recognit. 9, 1–5. doi: 10.1002/(sici)1099-1352(199601)9:1<1::aid-jmr241>3.0.co;2-6, PMID: 8723313

[ref21] Hai Y. Wei M. Y. Wang C. Y. Gu Y. C. Shao C. L. (2021). The intriguing chemistry and biology of sulfur-containing natural products from marine microorganisms (1987–2020). Mar. Life Sci. Technol. 3, 488–518. doi: 10.1007/s42995-021-00101-2, PMID: 37073258 PMC10077240

[ref22] Honda R. Lowe E. D. Dubinina E. Skamnaki V. Cook A. Brown N. R.. (2005). The structure of cyclin E1/CDK2: implications for CDK2 activation and CDK2-independent roles. EMBO J. 24, 452–463. doi: 10.1038/sj.emboj.7600554, PMID: 15660127 PMC548659

[ref23] Jumper J. Evans R. Pritzel A. Green T. Figurnov M. Ronneberger O.. (2021). Highly accurate protein structure prediction with AlphaFold. Nature 596, 583–589. doi: 10.1038/s41586-021-03819-2, PMID: 34265844 PMC8371605

[ref24] Kaiser J. Gertzen C. G. Bernauer T. Höfner G. Niessen K. V. Seeger T.. (2023). A novel binding site in the nicotinic acetylcholine receptor for MB327 can explain its allosteric modulation relevant for organophosphorus-poisoning treatment. Toxicol. Lett. 373, 160–171. doi: 10.1016/j.toxlet.2022.11.018, PMID: 36503818

[ref25] Katsila T. Spyroulias G. A. Patrinos G. P. Matsoukas M. T. (2016). Computational approaches in target identification and drug discovery. Comput. Struct. Biotechnol. J. 14, 177–184. doi: 10.1016/j.csbj.2016.04.004, PMID: 27293534 PMC4887558

[ref26] Kjer J. Debbab A. Aly A. H. Proksch P. (2010). Methods for isolation of marine-derived endophytic fungi and their bioactive secondary products. Nat. Protoc. 5, 479–490. doi: 10.1038/nprot.2009.233, PMID: 20203665

[ref27] Kovács T. Lajter I. Kúsz N. Schelz Z. Bózsity-Faragó N. Borbás A.. (2023). Isolation and NMR scaling factors for the structure determination of Lobatolide H, a flexible sesquiterpene from *Neurolaena lobata*. Int. J. Mol. Sci. 24:5841. doi: 10.3390/ijms24065841, PMID: 36982924 PMC10052924

[ref28] Li X. Chang H. Bouma J. de Paus L. V. Mukhopadhyay P. Paloczi J.. (2023). Structural basis of selective cannabinoid CB_2_ receptor activation. Nat. Commun. 14:1447. doi: 10.1038/s41467-023-37112-9, PMID: 36922494 PMC10017709

[ref29] Li W. S. Yan R. J. Yu Y. Shi Z. Mándi A. Shen L.. (2020). Determination of the absolute configuration of super-carbon-chain compounds by a combined chemical, spectroscopic, and computational approach: Gibbosols a and B. Angew. Chem. Int. Ed. 59, 13028–13036. doi: 10.1002/anie.202004358, PMID: 32343023

[ref30] Lide D. R. (2004). CRC handbook of chemistry and physics. Boca Raton, FL: CRC Press.

[ref31] Liu W. Zhai S. Zhang L. Chen Y. Liu Z. Ma W.. (2024). Expanding the chemical diversity of Grisechelins via heterologous expression. J. Nat. Prod. 87, 371–380. doi: 10.1021/acs.jnatprod.3c01132, PMID: 38301035

[ref32] Lucido M. J. Orlando B. J. Vecchio A. J. Malkowski M. G. (2016). Crystal structure of aspirin-acetylated human cyclooxygenase-2: insight into the formation of products with reversed stereochemistry. Biochemistry 55, 1226–1238. doi: 10.1021/acs.biochem.5b01378, PMID: 26859324 PMC4775376

[ref33] MacroModel. (2015). *Schrödinger, L.L.C*. Available at: http://www.schrodinger.com/MacroModel.

[ref34] Maestro (2020). Schrödinger Release 2020–4. New York: Maestro.

[ref35] Mándi A. Kurtán T. (2019). Applications of OR/ECD/VCD to the structure elucidation of natural products. Nat. Prod. Rep. 36, 889–918. doi: 10.1039/c9np00002j 31139804

[ref36] Mapelli M. Massimiliano L. Crovace C. Seeliger M. A. Tsai L. H. Meijer L.. (2005). Mechanism of CDK5/p25 binding by CDK inhibitors. J. Med. Chem. 48, 671–679. doi: 10.1021/jm049323m, PMID: 15689152

[ref37] Medicinal Plant Images Database. (2024). *School of Chinese Medicine, Hong Kong Baptist University*. Available at: https://sys01.lib.hkbu.edu.hk/cmed/mpid/detail.php?herb_id=D00879.

[ref38] Newman D. J. Cragg G. M. (2020). Natural products as sources of new drugs over the nearly four decades from 01/1981 to 09/2019. J. Nat. Prod. 83, 770–803. doi: 10.1021/acs.jnatprod.9b01285, PMID: 32162523

[ref39] Nickel J. Gohlke B. O. Erehman J. Banerjee P. Rong W. W. Goede A.. (2014). SuperPred: update on drug classification and target prediction. Nucleic Acids Res. 42 (Web Server issue), W26-31 42, W26–W31. doi: 10.1093/nar/gku477, PMID: 24878925 PMC4086135

[ref40] Osterhage C. Kaminsky R. König G. M. Wright A. D. (2000). Ascosalipyrrolidinone a, an antimicrobial alkaloid, from the obligate marine fungus *Ascochyta salicorniae*. J. Org. Chem. 65, 6412–6417. doi: 10.1021/jo000307g, PMID: 11052082

[ref41] Peeters M. C. Wisse L. E. Dinaj A. Vroling B. Vriend G. Ijzerman A. P. (2012). The role of the second and third extracellular loops of the adenosine A1 receptor in activation and allosteric modulation. Biochem. Pharmacol. 84, 76–87. doi: 10.1016/j.bcp.2012.03.008, PMID: 22449615

[ref42] Phillips N. J. Goodwin J. T. Fraiman A. Cole R. J. Lynn D. G. (1989). Characterization of the *Fusarium* toxin equisetin the use of phenylboronates in structure assignment. J. Am. Chem. Soc. 111, 8223–8231. doi: 10.1021/ja00203a025

[ref43] Pierens G. K. (2014). ^1^H and ^13^C NMR scaling factors for the calculation of chemical shifts in commonly used solvents using density functional theory. J. Comput. Chem. 35, 1388–1394. doi: 10.1002/jcc.23638, PMID: 24854878

[ref44] Qu C. Mao C. Xiao P. Shen Q. Zhong Y. N. Yang F.. (2021). Ligand recognition, unconventional activation, and G protein coupling of the prostaglandin E_2_ receptor EP_2_ subtype. Sci. Adv. 7:eabf1268. doi: 10.1126/sciadv.abf1268, PMID: 33811074 PMC11057787

[ref45] Ranganathan R. Lu K. P. Hunter T. Noel J. P. (1997). Structural and functional analysis of the mitotic rotamase Pin1 suggests substrate recognition is phosphorylation dependent. Cell 89, 875–886. doi: 10.1016/s0092-8674(00)80273-1, PMID: 9200606

[ref46] SAINT (2006). Data reduction and frame integration program for the CCD Area-Detector system. Madison, WI: Bruker Analytical X-ray Systems.

[ref47] Schrödinger L. (2010). *The PyMOL molecular graphics system, version 1.3 r1. Py-MOL, the PyMOL molecular graphics system*, Version 2010.

[ref48] Schumacher S. Dedden D. Nunez R. V. Matoba K. Takagi J. Biertümpfel C.. (2021). Structural insights into integrin α_5_β_1_ opening by fibronectin ligand. Sci. Adv. 7:eabe9716. doi: 10.1126/sciadv.abe9716, PMID: 33962943 PMC8104898

[ref49] Shao Z. Shen Q. Yao B. Mao C. Chen L. N. Zhang H.. (2022). Identification and mechanism of G protein-biased ligands for chemokine receptor CCR1. Nat. Chem. Biol. 18, 264–271. doi: 10.1038/s41589-021-00918-z, PMID: 34949837 PMC8885419

[ref50] Sheldrick G. M. (1996). Program SADABS. Göttingen, Germany: University of Göttingen.

[ref51] Sheldrick G. M. (2008). A short history of SHELX. Acta Cryst. A 64, 112–122. doi: 10.1107/S0108767307043930 18156677

[ref52] Sikandar A. Popoff A. Jumde R. P. Mándi A. Kaur A. Elgaher W. A. M.. (2023). Revision of the absolute configurations of Chelocardin and Amidochelocardin. Angew. Chem. Int. Ed. Engl. 62:e202306437. doi: 10.1002/anie.202306437, PMID: 37466921

[ref53] Singh J. P. Lin M. J. Hsu S. F. Peti W. Lee C. C. Meng T. C. (2021). Crystal structure of TCPTP unravels an allosteric regulatory role of helix α7 in phosphatase activity. Biochemistry 60, 3856–3867. doi: 10.1021/acs.biochem.1c00519, PMID: 34910875

[ref54] Singh S. B. Zink D. L. Goetz M. A. Dombrowski A. W. Polishook J. D. Hazuda D. J. (1998). Equisetin and a novel opposite stereochemical homolog phomasetin, two fungal metabolites as inhibitors of HIV-1 integrase. Tetrahedron Lett. 39, 2243–2246. doi: 10.1016/S0040-4039(98)00269-X

[ref55] Sotriffer C. A. Gohlke H. Klebe G. (2002). Docking into knowledge-based potential fields: a comparative evaluation of DrugScore. J. Med. Chem. 45, 1967–1970. doi: 10.1021/jm025507u, PMID: 11985464

[ref56] Stephens P. J. Harada N. (2010). ECD cotton effect approximated by the Gaussian curve and other methods. Chirality 22, 229–233. doi: 10.1002/chir.20733, PMID: 19408332

[ref57] Superchi S. Scafato P. Górecki M. Pescitelli G. (2018). Absolute configuration determination by quantum mechanical calculation of chiroptical spectra: basics and applications to fungal metabolites. Curr. Med. Chem. 25, 287–320. doi: 10.2174/0929867324666170310112009, PMID: 28294053

[ref58] Varetto U. (2009). *MOLEKEL 5.4; Swiss National Supercomputing Centre: Manno, Switzerland*.

[ref59] Vasilchenko A. S. Gurina E. V. Drozdov K. A. Vershinin N. A. Kravchenko S. V. Vasilchenko A. V. (2023). Exploring the antibacterial action of gliotoxin: does it induce oxidative stress or protein damage? Biochimie 214, 86–95. doi: 10.1016/j.biochi.2023.06.009 37356563

[ref60] Vasseur R. Baud S. Steffenel L. A. Vigouroux X. Martiny L. Krajecki M.. (2015). Inverse docking method for new proteins targets identification: a parallel approach. Parallel Comput. 42, 48–59. doi: 10.1016/j.parco.2014.09.008

[ref61] Zhang J. Zhang K. Gao Z. G. Paoletta S. Zhang D. Han G. W.. (2014). Agonist-bound structure of the human P2Y_12_ receptor. Nature 509, 119–122. doi: 10.1038/nature13288, PMID: 24784220 PMC4128917

[ref62] Zhao J. H. Wang Y. W. Yang J. Tong Z. J. Wu J. Z. Wang Y. B.. (2023). Natural products as potential lead compounds to develop new antiviral drugs over the past decade. Eur. J. Med. Chem. 260:115726. doi: 10.1016/j.ejmech.2023.115726, PMID: 37597436

