# Supplementary material for: Aplospojaveedins A–C, unusual sulfur-containing alkaloids produced by the endophytic fungus Aplosporella javeedii using OSMAC strategy
Source: Front Microbiol. 2024 Sep 27;15:1458622. doi: 10.3389/fmicb.2024.1458622 (PMC11466890; doi:10.3389/fmicb.2024.1458622)
Supplement: Supplementary file 1 [file Data_Sheet_1.PDF]

Supplementary material

Aplospojaveedins A–C, unusual sulfur-containing alkaloids produced by the endophytic fungus Aplosporella javeedii using OSMAC strategy

Ying Gao[1,2], Marian Frank[2], Nicole Teusch[2], Dennis Woschko[3], Christoph Janiak[3], Attila Mándi[4], Tibor Kurtán[4], Rudolf Hartmann[5], Katja Schiedlauske[2], Lasse van Geelen[2], Rainer Kalscheuer[2], Jesko Kaiser[6], Christoph G.W. Gertzen[6], Holger Gohlke[6,7], Bin-Gui Wang[8], Peter Proksch[2*], and Zhen Liu[1*]

[1]Key Laboratory of Study and Discovery of Small Targeted Molecules of Hunan Province, School of Medicine, Hunan Normal University, Changsha, China

[2]Institute of Pharmaceutical Biology and Biotechnology, Heinrich Heine University, Düsseldorf, Germany

[3]Institute of Inorganic and Structural Chemistry, Heinrich Heine University, Düsseldorf, Germany

[4]Department of Organic Chemistry, University of Debrecen, Debrecen, Hungary

[5]Institute of Biological Information Processing: Structural Biochemistry (IBI-7), Forschungszentrum Jülich GmbH, Jülich, Germany

[6]Institute for Pharmaceutical and Medicinal Chemistry, Heinrich Heine University, Düsseldorf, Germany

[7]Institute of Bio- and Geosciences (IBG-4: Bioinformatics), Forschungszentrum Jülich GmbH, Jülich, Germany

[8]CAS and Shandong Province Key Laboratory of Experimental Marine Biology, Institute of Oceanology, Chinese Academy of Sciences, Qingdao, China

*** Correspondence:**

Peter Proksch

proksch@uni-duesseldorf.de

Zhen Liu

liuzhen2020@hunnu.edu.cn; zhenfeizi0@sina.com

Content

Table S1. ^{1}H (600 MHz) and ^{13}C (150 MHz) NMR Data of Compounds **1-3** in CD_3OD. 5
Targets Prediction by Molecular Informatics Approaches 6
Table S2 Targets being predicted by both SwissTargetPrediction and SuperPred for compound **1**.[a] 6
Table S3 Targets being predicted by both SwissTargetPrediction and SuperPred for compound **2**.[a] 6
Table S4 Targets being predicted by both SwissTargetPrediction and SuperPred for compound **3**.[a] 7
X-ray Crystallographic Analysis of Aplospojaveedins A (1) 8
Table S5. Single crystal X-ray diffraction data of Compound **1**: (CCDC number 2295373) 8
Figure S1. Unit cell packing diagram for compound **1** along two different viewing directions 16
ECD and NMR Calculations 17
Table S6. Comparison of the experimental ^{13}C NMR data of **1** measured in DMSO-d_6 with the mPW1PW91/6-311+G(2d,p) // B3LYP/6-31+G(d,p) ones of (3*R*,4*S*,5*R*,8*R*,10*S*,19*R*,24*R*)-**1** [*RR*], (3*R*,4*S*,5*R*,8*R*,10*S*,19*R*,24*S*)-**1** [*RS*], (3*R*,4*S*,5*R*,8*R*,10*S*,19*S*,24*R*)-**1** [*SR*] and (3*R*,4*S*,5*R*,8*R*,10*S*,19*S*,24*S*)-**1** [*SS*] corrected for DMSO. 17
Table S7. Comparison of the experimental ^{13}C NMR data of **1** measured in MeOH-d_4 with the mPW1PW91/6-311+G(2d,p) // B3LYP/6-31+G(d,p) ones of (3*R*,4*S*,5*R*,8*R*,10*S*,19*R*,24*R*)-**1** [*RR*], (3*R*,4*S*,5*R*,8*R*,10*S*,19*R*,24*S*)-**1** [*RS*], (3*R*,4*S*,5*R*,8*R*,10*S*,19*S*,24*R*)-**1** [*SR*] and (3*R*,4*S*,5*R*,8*R*,10*S*,19*S*,24*S*)-**1** [*SS*] corrected for MeOH. 18
Table S8. Comparison of the experimental ^{13}C NMR data of **2** measured in DMSO-d_6 with the mPW1PW91/6-311+G(2d,p) // B3LYP/6-31+G(d,p) ones of (3*R*,4*S*,5*R*,8*R*,10*S*,19*R*,24*R*)-**2** [*RR*], (3*R*,4*S*,5*R*,8*R*,10*S*,19*R*,24*S*)-**2** [*RS*], (3*R*,4*S*,5*R*,8*R*,10*S*,19*S*,24*R*)-**2** [*SR*] , and (3*R*,4*S*,5*R*,8*R*,10*S*,19*S*,24*S*)-**2** [*SS*] corrected for DMSO. 19
Table S9. Comparison of the experimental ^{13}C NMR data of **2** measured in MeOH-d_4 with the mPW1PW91/6-311+G(2d,p) // B3LYP/6-31+G(d,p) ones of (3*R*,4*S*,5*R*,8*R*,10*S*,19*R*,24*R*)-**2** [*RR*], (3*R*,4*S*,5*R*,8*R*,10*S*,19*R*,24*S*)-**2** [*RS*], (3*R*,4*S*,5*R*,8*R*,10*S*,19*S*,24*R*)-**2** [*SR*] and (3*R*,4*S*,5*R*,8*R*,10*S*,19*S*,24*S*)-**2** [*SS*] corrected for MeOH. 20
Table S10. Comparison of the experimental ^{13}C NMR data of **3** measured in DMSO-d_6 with the mPW1PW91/6-311+G(2d,p) // B3LYP/6-31+G(d,p) ones of (3*R*,4*S*,5*R*,8*R*,10*S*,19*R*,24*R*)-**3** [*RR*], (3*R*,4*S*,5*R*,8*R*,10*S*,19*R*,24*S*)-**3** [*RS*], (3*R*,4*S*,5*R*,8*R*,10*S*,19*S*,24*R*)-**3** [*SR*] and (3*R*,4*S*,5*R*,8*R*,10*S*,19*S*,24*S*)-**3** [*SS*] corrected for DMSO. 21
Table S11. Comparison of the experimental ^{13}C NMR data of **3** measured in MeOH-d_4 with the mPW1PW91/6-311+G(2d,p) // B3LYP/6-31+G(d,p) ones of (3*R*,4*S*,5*R*,8*R*,10*S*,19*R*,24*R*)-**3** [*RR*], (3*R*,4*S*,5*R*,8*R*,10*S*,19*R*,24*S*)-**3** [*RS*], (3*R*,4*S*,5*R*,8*R*,10*S*,19*S*,24*R*)-**3** [*SR*] and (3*R*,4*S*,5*R*,8*R*,10*S*,19*S*,24*S*)-**3** [*SS*] corrected for MeOH. 22

Figure S2. Experimental ECD spectrum of compound **1** in MeOH (black line) compared with the calculated PBE0/TZVP PCM/MeOH spectrum of (3*R*,4*S*,5*R*,8*R*,10*S*,19*R*,24*S*)-**1**. Level of DFT optimization: ωB97X/TZVP PCM/MeOH. 23
Figure S3. Experimental ECD spectrum of compound **1** in MeOH (black line) compared with the calculated PBE0/TZVP PCM/MeOH spectrum of (3*R*,4*S*,5*R*,8*R*,10*S*,19*S*,24*R*)-**1**. Level of DFT optimization: ωB97X/TZVP PCM/MeOH. 23
Figure S4. Experimental ECD spectrum of compound **1** in MeOH (black line) compared with the calculated PBE0/TZVP PCM/MeOH spectrum of (3*R*,4*S*,5*R*,8*R*,10*S*,19*S*,24*S*)-**1**. Level of DFT optimization: ωB97X/TZVP PCM/MeOH 24
Figure S5. Experimental ECD spectrum of compound **2** in MeOH (black line) compared with the calculated PBE0/TZVP PCM/MeOH spectrum of (3*R*,4*S*,5*R*,8*R*,10*S*,19*R*,24*R*)-**2**. Level of DFT optimization: ωB97X/TZVP PCM/MeOH 24
Figure S6. Experimental ECD spectrum of compound **2** in MeOH (black line) compared with the calculated PBE0/TZVP PCM/MeOH spectrum of (3*R*,4*S*,5*R*,8*R*,10*S*,19*R*,24*S*)-**2**. Level of DFT optimization: ωB97X/TZVP PCM/MeOH 25
Figure S7. Experimental ECD spectrum of compound **2** in MeOH (black line) compared with the calculated PBE0/TZVP PCM/MeOH spectrum of (3*R*,4*S*,5*R*,8*R*,10*S*,19*S*,24*S*)-**2**. Level of DFT optimization: ωB97X/TZVP PCM/MeOH 25
Figure S8. Experimental ECD spectrum of compound **3** in MeOH (black line) compared with the calculated BH&HLYP/TZVP PCM/MeOH spectrum of (3*R*,4*S*,5*R*,8*R*,10*S*,19*R*,24*R*)-**3**. Level of DFT optimization: ωB97X/TZVP PCM/MeOH 26
Figure S9. Experimental ECD spectrum of compound **3** in MeOH (black line) compared with the calculated BH&HLYP/TZVP PCM/MeOH spectrum of (3*R*,4*S*,5*R*,8*R*,10*S*,19*R*,24*S*)-**3**. Level of DFT optimization: ωB97X/TZVP PCM/MeOH 26
Figure S10. Experimental ECD spectrum of compound **3** in MeOH (black line) compared with the calculated BH&HLYP/TZVP PCM/MeOH spectrum of (3*R*,4*S*,5*R*,8*R*,10*S*,19*S*,24*S*)-**3**. Level of DFT optimization: ωB97X/TZVP PCM/MeOH. 27
Figure S11. Structure and population of the low-energy ωB97X/TZVP PCM/MeOH conformers (≥ 1%) of (3*R*,4*S*,5*R*,8*R*,10*S*,19*R*,24*R*)-**1** 27
Figure S12. Structure and population of the low-energy ωB97X/TZVP PCM/MeOH conformers (≥ 1%) of (3*R*,4*S*,5*R*,8*R*,10*S*,19*S*,24*R*)-**2**. 28
Figure S13. Structure and population of the low-energy ωB97X/TZVP PCM/MeOH conformers (≥ 1%) of (3*R*,4*S*,5*R*,8*R*,10*S*,19*S*,24*R*)-**3**. 28
Table S12. Cartesian coordinates and energies of the low-energy conformers calculated at the B3LYP/6-31+G(d,p) level. 29
Table S13. Cartesian coordinates and energies of the low-energy conformers calculated at the ωB97X/TZVP PCM/MeOH level. 69
HPLC Chromatograms; HRESIMS; NMR Spectra 122
Figure S14. HPLC chromatogram of Aplospojaveedins A (1) 122
Figure S15. HRESIMS of compound **1** 122
Figure S16. ^{1}H NMR (800M Hz, DMSO-d_4) spectrum of compound **1** 123
Figure S17. ^{13}C NMR (200M Hz, DMSO-d_6) spectrum of compound **1** 123
Figure S18. HSQC (DMSO-d_6) spectrum of compound **1** 124

Figure S19. COSY (DMSO-d_6) spectrum of compound **1** 124
Figure S20. HMBC (DMSO-d_6) spectrum of compound **1** 125
Figure S21. ROESY (DMSO-d_6) spectrum of compound **1** 125
Figure S22. ^{1}H-^{15}N-HMBC (DMSO-d_6) spectrum of compound **1** 126
Figure S23. ^{1}H NMR (600M Hz, CD_3OD) spectrum of compound **1** 126
Figure S24. ^{13}C NMR (150M Hz, CD_3OD) spectrum of compound **1** 127
Figure S25. HSQC (CD_3OD) spectrum of compound **1** 127
Figure S26. COSY (CD_3OD) spectrum of compound **1** 128
Figure S27. HMBC (CD_3OD) spectrum of compound **1** 128
Figure S28. ROESY (CD_3OD) spectrum of compound **1** 129
Figure S29. HPLC chromatogram of Aplospojaveedins B (2) 129
Figure S30. HRESIMS of compound **2** 130
Figure S31. ^{1}H NMR (600M Hz, DMSO-d_4) spectrum of compound **2** 130
Figure S32. ^{13}C NMR (150M Hz, DMSO-d_6) spectrum of compound **2** 131
Figure S33. HSQC (DMSO-d_6) spectrum of compound **2** 131
Figure S34. COSY (DMSO-d_6) spectrum of compound **2** 132
Figure S35. HMBC (DMSO-d_6) spectrum of compound **2** 132
Figure S36. ROESY (DMSO-d_6) spectrum of compound **2** 133
Figure S37. ^{1}H-^{15}N-HMBC (DMSO-d_6) spectrum of compound **2** 133
Figure S38. ^{1}H NMR (600M Hz, CD_3OD) spectrum of compound **2** 134
Figure S39. ^{13}C NMR (150M Hz, CD_3OD) spectrum of compound **2** 134
Figure S40. HSQC (CD_3OD) spectrum of compound **2** 135
Figure S41. COSY (CD_3OD) spectrum of compound **2** 135
Figure S42. HMBC (CD_3OD) spectrum of compound **2** 136
Figure S43. ROESY (CD_3OD) spectrum of compound **2** 136
Figure S44. HPLC chromatogram of Aplospojaveedins C (3) 137
Figure S45. HRESIMS of compound **3** 137
Figure S46. ^{1}H NMR (750M Hz, DMSO-d_4) spectrum of compound **3** 138
Figure S47. ^{13}C NMR (188M Hz, DMSO-d_4) spectrum of compound **3** 138
Figure S48. HSQC (DMSO-d_4) spectrum of compound **3** 139
Figure S49.COSY (DMSO-d_4) spectrum of compound **3** 139
Figure S50.HMBC (DMSO-d_4) spectrum of compound **3** 140
Figure S51. ROESY (DMSO-d_4) spectrum of compound **3** 140
Figure S52. ^{1}H-^{15}N-HMBC (DMSO-d_6) spectrum of compound **3** 141
Figure S53. ^{1}H NMR (600M Hz, CD_3OD) spectrum of compound **3** 141
Figure S54. ^{13}C NMR (150M Hz, CD_3OD) spectrum of compound **3** 142
Figure S55. HSQC (CD_3OD) spectrum of compound **3** 142
Figure S56. COSY (CD_3OD) spectrum of compound **3** 143
Figure S57. HMBC (CD_3OD) spectrum of compound **3** 143
Figure S58. ROESY (CD_3OD) spectrum of compound **3** 144
Figure S59. Proposed biosynthetic pathway 144
Figure S60. Key ROESY correlations for compound **1** 145
Figure S61. Key ROESY correlations for compound **2** 145
Figure S62. Key ROESY correlations for compound **3** 146

Table S1. ^{1}H (600 MHz) and ^{13}C (150 MHz) NMR Data of Compounds **1-3** in CD_3OD.

NO.	**1**		**2**		**3**	
	δ_C, type	δ_H (J in Hz)	δ_C, type	δ_H (J in Hz)	δ_C, type	δ_H (J in Hz)
1	133.5, CH	5.55, br d (9.9)	133.3, CH	5.53, br d (9.9)	133.3, CH	5.53, br d (9.9)
2	129.2, CH	5.35, ddd (9.9, 4.2, 2.7)	129.4, CH	5.34, ddd (9.9, 4.0, 2.7)	129.6, CH	5.35, ddd (9.9, 4.0, 2.7)
3	48.5, CH	3.29, m	48.1, CH	3.50, m	48.4, CH	3.28, m
4	53.9, CH	3.91, dd (11.8,7.0)	54.1, CH	3.76, dd (11.9, 7.1)	53.3, CH	3.86, dd (11.8, 7.1)
5	38.1, CH	1.63, m	38.5, CH	1.57, m	37.9, CH	1.62, m
6	31.4, CH_2	1.85, m	31.3, CH_2	1.65, m	31.6, CH_2	1.88, m
		0.78, m		0.75, m		0.80, m
7	36.6, CH_2	1.71, m	36.5, CH_2	1.68, m	36.5, CH_2	1.69, m
		1.03, m		0.98, m		1.06, m
8	34.4, CH	1.50, m	34.4, CH	1.49, m	34.5, CH	1.49, m
9	43.2, CH_2	1.79, m	43.2, CH_2	1.77, m	43.3, CH_2	1.77, m
		0.83, m		0.80, m		0.84, m
10	42.4, CH	1.77, m	42.1, CH	1.75, m	42.5, CH	1.76, m
11	23.0, CH_3	0.93, d (6.5)	23.0, CH_3	0.92, d (6.5)	23.0, CH_3	0.93, d (6.5)
12	137.2, C		137.8, C		137.6, C	
13	123.9, CH	5.10, q (6.7)	123.5, CH	5.10, q (6.7)	123.3, CH	5.06, q (6.6)
14	14.2, CH_3	1.49, d (6.7)	13.6, CH_3	1.45, d (6.7)	13.9, CH_3	1.44, d (6.6)
15	15.6, CH_3	1.45, s	15.9, CH_3	1.42, s	15.8, CH_3	1.47, s
16	199.4, C		199.7, C		201.1, C	
17	124.3, C		127.2, C		101.7, C	
18	177.8, C		174.7, C		173.6, C	
19	72.9, C		72.3, C		61.7, C	
21	169.6, C		168.9, C		171.9, C	
22	29.1, CH_3	1.66, s	31.5, CH_3	1.72, s	30.2, CH_3	1.88, s
24	54.5, CH	3.66, dd (12.3, 5.3)	54.2, CH	4.08, dd (4.1, 3.6)	53.6, CH	4.55, dd (11.9, 4.3)
25	27.2, CH_2	3.25, dd (14.6, 12.3)	29.0, CH_2	3.43, dd (13.7, 4.1)	30.5, CH_2	3.38, dd (11.1, 4.3)
		3.15, dd (14.6, 5.3)		3.29, dd (13.7, 3.6)		2.72, dd (11.9, 11.1)
26	174.9, C		173.9, C		169.5, C	
28	41.9, CH_2	4.01, d (17.9)	42.2, CH_2	3.86, d (18.1)	43.1, CH_2	3.95, s
		3.95, d (17.9)		3.78, d (18.1)		
29	172.9, C		172.3, C		174.0, C	

Targets Prediction by Molecular Informatics Approaches

Table S2 Targets being predicted by both SwissTargetPrediction and SuperPred for compound **1**.[a]

UniProt ID	Protein name
P34972	Cannabinoid receptor 2
P16234	Platelet-derived growth factor receptor alpha
Q9H244	P2Y purinoceptor 12
P32246	C-C chemokine receptor type 1
P29275	Adenosine receptor A2b
P43116	Prostaglandin E2 receptor EP2 subtype
P05556 P08648	Integrin alpha-5 / beta-1
P42338	Phosphatidylinositol 4,5-bisphosphate 3-kinase catalytic subunit beta isoform
P06756 P05106	Integrin alpha-V / beta-3
Q14145	Kelch-like ECH-associated protein 1
O15054	Lysine-specific demethylase 6B
P35354	Prostaglandin G/H synthase 2

[a] Compounds are sorted by the probability that they bind to the respective target according to the prediction tools. The colors green, orange, and purple indicate that the target was proposed for all three compounds, compounds **1** and **2**, or compounds **1** and **3**, respectively.

Table S3 Targets being predicted by both SwissTargetPrediction and SuperPred for compound **2**.[a]

UniProt ID	Protein name
P34972	Cannabinoid receptor 2
P17706	Tyrosine-protein phosphatase non-receptor type 2
P43116	Prostaglandin E2 receptor EP2 subtype
P40763	Signal transducer and activator of transcription 3
P21462	fMet-Leu-Phe receptor
Q14145	Kelch-like ECH-associated protein 1
P28074	Proteasome subunit beta type-5
P09237	Matrilysin
P35414	Apelin receptor

[a] Compounds are sorted by the probability that they bind to the respective target according to the prediction tools. The colors green, orange, and blue indicate that the target was proposed for all three compounds, compounds **1** and **2**, or compounds **2** and **3**, respectively.

Table S4 Targets being predicted by both SwissTargetPrediction and SuperPred for compound **3**.[a]

UniProt ID	Protein name
P34972	Cannabinoid receptor 2
P24864 P24941	Cyclin-dependent kinase 2/cyclin E1
Q15078 Q00535	Cyclin-dependent kinase 5/CDK5 activator 1
Q969S8	Polyamine deacetylase HDAC10
Q9H244	P2Y purinoceptor 12
P43116	Prostaglandin E2 receptor EP2 subtype
P40763	Signal transducer and activator of transcription 3
O00206	Toll-like receptor 4
P21462	fMet-Leu-Phe receptor
P42338	Phosphatidylinositol 4,5-bisphosphate 3-kinase catalytic subunit beta isoform12
Q13526	Peptidyl-prolyl cis-trans isomerase NIMA-interacting 1
Q96DB2	Histone deacetylase 11
P06756 P05106	Integrin alpha-V / beta-3
P35354	Prostaglandin G/H synthase 2

[a] Compounds are sorted by the probability that they bind to the respective target according to the prediction tools. The colors green, purple, and blue indicate that the target was proposed for all three compounds, compounds **1** and **3**, or compounds **2** and **3**, respectively.

X-ray Crystallographic Analysis of Aplospojaveedins A (1)

Table S5. Single crystal X-ray diffraction data of Compound **1**: (CCDC number 2295373)

a) Crystal data

$C_{26}H_{35}N_3O_5S{\cdot}H_2O$	D_x = 1.306 Mg m^{-3}
M_r = 519.64	Cu $K\alpha$ radiation, λ = 1.54178 Å
Orthorhombic, $P2_12_12_1$	Cell parameters from 9168 reflections
a = 7.7908 (4) Å	θ = 5.7–69.6°
b = 7.9359 (4) Å	μ = 1.46 mm^{-1}
c = 42.745 (2) Å	T = 140 K
V = 2642.8 (2) Å^3	Plate, clear colourless
Z = 4	0.29 × 0.23 × 0.13 mm
F(000) = 1112	

b) Data collection

Bruker Kappa APEX-II CCD area detector diffractometer	4788 independent reflections
Radiation source: microfocus sealed tube	4680 reflections with $I > 2\sigma(I)$
Multilayer mirror monochromator	R_{int} = 0.027
ω scans, ϕ scans	θ_{max} = 68.2°, θ_{min} = 2.1°
Absorption correction: multi-scan (*SADABS*; Sheldrick, 1996)	h = -8→9
T_{min} = 0.867, T_{max} = 1.000	k = -9→9
23112 measured reflections	l = -50→51

c) Refinement

Refinement on F^2	Secondary atom site location: difference Fourier map
Least-squares matrix: full	Hydrogen site location: mixed
$R[F^2 > 2\sigma(F^2)]$ = 0.0235<br>$R[F^2$, all data] = 0.0242	H atoms treated by a mixture of independent and constrained refinement
$wR[F^2 > 2\sigma(F^2)]$ = 0.0605<br>$wR(F^2$, all data) = 0.0609	$w = 1/[\sigma^2(F_o^2) + (0.033P)^2 + 0.3847P]$<br>where $P = (F_o^2 + 2F_c^2)/3$
S = 1.03	$(\Delta/\sigma)_{max}$ = 0.001
4788 reflections	$\Delta\rangle_{max}$ = 0.18 e Å^{-3}

347 parameters	$\Delta\rangle_{min}$ = -0.16 e Å^{-3}
0 restraints	Absolute structure: Flack x determined using 1889 quotients [(I+)-(I-)]/[(I+)+(I-)] (Parsons, Flack and Wagner, Acta Cryst. B69 (2013) 249-259).
Primary atom site location: structure-invariant direct methods	Absolute structure parameter: 0.033 (4)

d) Fractional atomic coordinates and isotropic or equivalent isotropic displacement parameters (Å2) for compound **1**

	x	*y*	*z*	U_{iso}*/U_{eq}
S1	0.29819 (6)	0.60922 (5)	0.61648 (2)	0.02381 (11)
O1	0.45566 (19)	0.36902 (18)	0.57922 (3)	0.0326 (3)
C1	1.0136 (3)	0.0929 (3)	0.57047 (5)	0.0340 (5)
H1	1.123852	0.058372	0.563450	0.041*
O2	0.59877 (17)	0.09695 (15)	0.66382 (3)	0.0254 (3)
C2	1.0050 (3)	0.2000 (3)	0.59416 (5)	0.0318 (4)
H2	1.110060	0.237216	0.603095	0.038*
O3	0.13826 (18)	0.97969 (16)	0.69322 (3)	0.0297 (3)
C3	0.8410 (2)	0.2674 (2)	0.60799 (4)	0.0239 (4)
H3	0.843286	0.239591	0.630805	0.029*
O4	0.38666 (18)	1.15699 (17)	0.74431 (3)	0.0294 (3)
C4	0.6791 (2)	0.1770 (2)	0.59385 (4)	0.0216 (4)
H4	0.662081	0.069290	0.605586	0.026*
O5	0.1807 (2)	1.17221 (18)	0.78070 (3)	0.0335 (3)
C5	0.6990 (2)	0.1327 (2)	0.55908 (4)	0.0232 (4)
H5A	0.714870	0.239956	0.547144	0.028*
H5	0.203 (3)	1.273 (3)	0.7810 (5)	0.035*
O6	0.2005 (2)	1.49427 (17)	0.78697 (3)	0.0333 (3)
H6A	0.264 (4)	1.527 (3)	0.8023 (7)	0.050*
H6B	0.093 (4)	1.508 (3)	0.7948 (6)	0.050*
C6	0.5431 (3)	0.0403 (3)	0.54548 (4)	0.0299 (4)
H6C	0.439499	0.111371	0.547965	0.036*
H6D	0.524259	-0.065777	0.557161	0.036*
C7	0.5698 (3)	0.0003 (3)	0.51071 (4)	0.0309 (4)
H7A	0.470623	-0.065672	0.502992	0.037*
H7B	0.573802	0.107145	0.498771	0.037*
C8	0.7342 (3)	-0.0986 (2)	0.50459 (4)	0.0288 (4)

H8	0.722642	-0.210985	0.514968	0.035*
C9	0.8876 (3)	-0.0085 (2)	0.51918 (4)	0.0308 (4)
H9A	0.905949	0.100462	0.508414	0.037*
H9B	0.992069	-0.077855	0.516298	0.037*
C10	0.8590 (3)	0.0230 (2)	0.55414 (4)	0.0260 (4)
H10	0.834238	-0.088927	0.563884	0.031*
C11	0.7617 (3)	-0.1287 (3)	0.46967 (4)	0.0367 (5)
H11A	0.667905	-0.198157	0.461467	0.055*
H11B	0.871176	-0.186911	0.466440	0.055*
H11C	0.763533	-0.020314	0.458686	0.055*
C12	0.8325 (3)	0.4591 (2)	0.60548 (4)	0.0257 (4)
C13	0.8051 (3)	0.5519 (2)	0.63092 (4)	0.0255 (4)
H13	0.801185	0.494989	0.650463	0.031*
C14	0.7797 (3)	0.7407 (2)	0.63116 (5)	0.0322 (4)
H14A	0.863836	0.793623	0.617252	0.048*
H14B	0.795184	0.783398	0.652487	0.048*
H14C	0.663474	0.767381	0.623912	0.048*
C15	0.8499 (3)	0.5343 (3)	0.57339 (5)	0.0403 (5)
H15A	0.920577	0.460369	0.560314	0.060*
H15B	0.904446	0.645264	0.574966	0.060*
H15C	0.735895	0.546459	0.563946	0.060*
C16	0.5242 (2)	0.2880 (2)	0.60002 (4)	0.0224 (4)
C17	0.4626 (2)	0.3139 (2)	0.63218 (4)	0.0200 (3)
C18	0.3657 (2)	0.4492 (2)	0.64037 (4)	0.0198 (4)
C19	0.3506 (2)	0.4621 (2)	0.67584 (4)	0.0203 (4)
N20	0.4634 (2)	0.32575 (18)	0.68563 (4)	0.0223 (3)
H20	0.450 (3)	0.281 (3)	0.7032 (5)	0.034*
C21	0.5168 (2)	0.2291 (2)	0.66115 (4)	0.0202 (4)
C22	0.1718 (3)	0.4340 (2)	0.68953 (4)	0.0290 (4)
H22A	0.095704	0.525701	0.682946	0.043*
H22B	0.178943	0.431949	0.712417	0.043*
H22C	0.126089	0.326326	0.682015	0.043*
N23	0.4257 (2)	0.62337 (19)	0.68608 (3)	0.0219 (3)
H23	0.534 (3)	0.619 (3)	0.6803 (5)	0.033*
C24	0.3423 (2)	0.7771 (2)	0.67407 (4)	0.0221 (4)
H24	0.436390	0.854069	0.667077	0.026*
C25	0.2207 (3)	0.7551 (2)	0.64596 (4)	0.0262 (4)

H25A	0.202313	0.866459	0.636073	0.031*
H25B	0.108114	0.714854	0.653701	0.031*
C26	0.2444 (2)	0.8699 (2)	0.69995 (4)	0.0222 (4)
N27	0.2851 (2)	0.82820 (19)	0.72910 (3)	0.0256 (3)
H27	0.363 (3)	0.749 (3)	0.7323 (5)	0.038*
C28	0.2097 (3)	0.9142 (2)	0.75544 (4)	0.0253 (4)
H28A	0.237839	0.851354	0.774778	0.030*
H28B	0.083250	0.913656	0.753060	0.030*
C29	0.2701 (2)	1.0944 (2)	0.75896 (4)	0.0224 (4)

e) Atomic displacement parameters (Å2) for compound **1**

	U^{11}	U^{22}	U^{33}	U^{12}	U^{13}	U^{23}
S1	0.0286 (2)	0.0246 (2)	0.01826 (19)	0.00631 (19)	-0.00312 (17)	-0.00067 (16)
O1	0.0379 (8)	0.0392 (7)	0.0206 (6)	0.0147 (7)	-0.0014 (6)	-0.0009 (6)
C1	0.0240 (10)	0.0367 (11)	0.0411 (11)	0.0056 (9)	0.0023 (8)	-0.0075 (9)
O2	0.0286 (7)	0.0199 (6)	0.0277 (6)	0.0037 (5)	0.0022 (5)	0.0022 (5)
C2	0.0227 (10)	0.0339 (10)	0.0388 (11)	-0.0008 (9)	-0.0023 (9)	-0.0068 (8)
O3	0.0316 (8)	0.0273 (7)	0.0302 (7)	0.0083 (6)	-0.0008 (6)	-0.0031 (5)
C3	0.0261 (10)	0.0213 (8)	0.0242 (9)	-0.0003 (7)	-0.0012 (7)	-0.0011 (7)
O4	0.0312 (8)	0.0363 (7)	0.0208 (6)	-0.0057 (6)	0.0016 (6)	0.0012 (5)
C4	0.0236 (10)	0.0193 (8)	0.0218 (8)	0.0001 (7)	0.0012 (7)	-0.0011 (6)
O5	0.0379 (8)	0.0254 (6)	0.0371 (7)	-0.0043 (6)	0.0113 (6)	-0.0096 (6)
C5	0.0263 (9)	0.0223 (8)	0.0209 (8)	0.0012 (8)	0.0024 (7)	-0.0016 (7)
O6	0.0334 (8)	0.0325 (7)	0.0339 (7)	-0.0045 (7)	-0.0015 (7)	-0.0068 (6)
C6	0.0273 (11)	0.0368 (10)	0.0256 (9)	-0.0022 (9)	0.0027 (8)	-0.0087 (8)
C7	0.0345 (12)	0.0349 (10)	0.0234 (9)	-0.0004 (9)	-0.0004 (8)	-0.0054 (8)
C8	0.0416 (12)	0.0236 (8)	0.0213 (9)	0.0017 (9)	0.0042 (8)	-0.0015 (7)
C9	0.0328 (11)	0.0294 (9)	0.0303 (10)	0.0047 (8)	0.0079 (8)	-0.0044 (8)
C10	0.0285 (10)	0.0234 (9)	0.0259 (9)	0.0031 (8)	0.0026 (8)	-0.0023 (7)
C11	0.0507 (14)	0.0360 (10)	0.0234 (9)	0.0051 (10)	0.0067 (9)	-0.0024 (8)
C12	0.0261 (10)	0.0222 (9)	0.0287 (9)	-0.0026 (8)	0.0023 (8)	-0.0004 (7)
C13	0.0210 (9)	0.0250 (9)	0.0304 (9)	-0.0020 (7)	-0.0001 (8)	-0.0013 (7)
C14	0.0295 (11)	0.0255 (9)	0.0416 (11)	-0.0024 (9)	0.0002 (9)	-0.0075 (8)
C15	0.0618 (16)	0.0268 (10)	0.0324 (10)	-0.0046 (10)	0.0116 (10)	0.0020 (8)
C16	0.0242 (10)	0.0216 (9)	0.0214 (8)	0.0008 (7)	-0.0013 (7)	-0.0019 (7)
C17	0.0186 (9)	0.0199 (8)	0.0215 (8)	-0.0008 (7)	-0.0015 (7)	-0.0021 (6)

C18	0.0173 (9)	0.0213 (8)	0.0208 (8)	-0.0040 (7)	-0.0028 (7)	-0.0010 (7)
C19	0.0216 (9)	0.0200 (8)	0.0194 (8)	0.0021 (7)	-0.0009 (7)	0.0003 (6)
N20	0.0275 (8)	0.0221 (7)	0.0174 (7)	0.0028 (6)	0.0028 (6)	0.0027 (6)
C21	0.0190 (9)	0.0195 (8)	0.0222 (8)	-0.0031 (7)	0.0015 (7)	-0.0002 (7)
C22	0.0276 (10)	0.0302 (10)	0.0291 (9)	-0.0007 (8)	0.0055 (8)	0.0018 (7)
N23	0.0222 (8)	0.0214 (7)	0.0223 (7)	0.0021 (7)	-0.0017 (6)	-0.0038 (6)
C24	0.0242 (10)	0.0191 (8)	0.0229 (8)	0.0017 (7)	0.0007 (7)	-0.0009 (7)
C25	0.0323 (11)	0.0229 (8)	0.0235 (8)	0.0068 (8)	-0.0026 (8)	-0.0030 (7)
C26	0.0231 (9)	0.0178 (8)	0.0255 (9)	-0.0005 (7)	0.0001 (7)	-0.0021 (7)
N27	0.0301 (9)	0.0238 (7)	0.0228 (7)	0.0067 (7)	0.0001 (7)	-0.0044 (6)
C28	0.0298 (10)	0.0239 (9)	0.0220 (8)	0.0007 (8)	0.0045 (8)	-0.0023 (7)
C29	0.0244 (9)	0.0262 (9)	0.0167 (8)	0.0024 (8)	-0.0018 (7)	0.0001 (7)

f) Geometric parameters (Å, º) for compound **1**

S1—C18	1.7124 (17)	C11—H11B	0.9800
S1—C25	1.8147 (18)	C11—H11C	0.9800
O1—C16	1.220 (2)	C12—C13	1.331 (3)
C1—C2	1.324 (3)	C12—C15	1.502 (3)
C1—C10	1.499 (3)	C13—C14	1.511 (3)
C1—H1	0.9500	C13—H13	0.9500
O2—C21	1.233 (2)	C14—H14A	0.9800
C2—C3	1.506 (3)	C14—H14B	0.9800
C2—H2	0.9500	C14—H14C	0.9800
O3—C26	1.235 (2)	C15—H15A	0.9800
C3—C12	1.527 (2)	C15—H15B	0.9800
C3—C4	1.572 (2)	C15—H15C	0.9800
C3—H3	1.0000	C16—C17	1.471 (2)
O4—C29	1.210 (2)	C17—C18	1.358 (2)
C4—C16	1.517 (2)	C17—C21	1.472 (2)
C4—C5	1.535 (2)	C18—C19	1.524 (2)
C4—H4	1.0000	C19—N20	1.456 (2)
O5—C29	1.315 (2)	C19—N23	1.474 (2)
O5—H5	0.82 (3)	C19—C22	1.527 (3)
C5—C6	1.533 (3)	N20—C21	1.362 (2)
C5—C10	1.535 (3)	N20—H20	0.84 (2)
C5—H5A	1.0000	C22—H22A	0.9800

O6—H6A	0.86 (3)	C22—H22B	0.9800
O6—H6B	0.91 (3)	C22—H22C	0.9800
C6—C7	1.534 (3)	N23—C24	1.474 (2)
C6—H6C	0.9900	N23—H23	0.88 (3)
C6—H6D	0.9900	C24—C26	1.532 (2)
C7—C8	1.525 (3)	C24—C25	1.540 (3)
C7—H7A	0.9900	C24—H24	1.0000
C7—H7B	0.9900	C25—H25A	0.9900
C8—C9	1.526 (3)	C25—H25B	0.9900
C8—C11	1.527 (2)	C26—N27	1.327 (2)
C8—H8	1.0000	N27—C28	1.442 (2)
C9—C10	1.531 (2)	N27—H27	0.88 (3)
C9—H9A	0.9900	C28—C29	1.514 (2)
C9—H9B	0.9900	C28—H28A	0.9900
C10—H10	1.0000	C28—H28B	0.9900
C11—H11A	0.9800		
C18—S1—C25	99.28 (8)	C13—C14—H14A	109.5
C2—C1—C10	123.59 (19)	C13—C14—H14B	109.5
C2—C1—H1	118.2	H14A—C14—H14B	109.5
C10—C1—H1	118.2	C13—C14—H14C	109.5
C1—C2—C3	124.82 (19)	H14A—C14—H14C	109.5
C1—C2—H2	117.6	H14B—C14—H14C	109.5
C3—C2—H2	117.6	C12—C15—H15A	109.5
C2—C3—C12	111.27 (17)	C12—C15—H15B	109.5
C2—C3—C4	111.60 (14)	H15A—C15—H15B	109.5
C12—C3—C4	113.14 (15)	C12—C15—H15C	109.5
C2—C3—H3	106.8	H15A—C15—H15C	109.5
C12—C3—H3	106.8	H15B—C15—H15C	109.5
C4—C3—H3	106.8	O1—C16—C17	117.67 (16)
C16—C4—C5	112.42 (14)	O1—C16—C4	121.83 (16)
C16—C4—C3	107.85 (14)	C17—C16—C4	120.23 (15)
C5—C4—C3	113.33 (15)	C18—C17—C16	122.23 (16)
C16—C4—H4	107.7	C18—C17—C21	107.69 (15)
C5—C4—H4	107.7	C16—C17—C21	128.97 (16)
C3—C4—H4	107.7	C17—C18—C19	110.66 (15)
C29—O5—H5	111.0 (17)	C17—C18—S1	127.09 (14)

C6—C5—C4	113.38 (15)	C19—C18—S1	121.30 (12)
C6—C5—C10	108.66 (14)	N20—C19—N23	108.69 (14)
C4—C5—C10	110.18 (15)	N20—C19—C18	100.90 (13)
C6—C5—H5A	108.2	N23—C19—C18	108.86 (14)
C4—C5—H5A	108.2	N20—C19—C22	109.40 (14)
C10—C5—H5A	108.2	N23—C19—C22	112.03 (14)
H6A—O6—H6B	103 (2)	C18—C19—C22	116.23 (15)
C5—C6—C7	111.03 (16)	C21—N20—C19	112.47 (14)
C5—C6—H6C	109.4	C21—N20—H20	119.3 (16)
C7—C6—H6C	109.4	C19—N20—H20	119.8 (16)
C5—C6—H6D	109.4	O2—C21—N20	124.46 (16)
C7—C6—H6D	109.4	O2—C21—C17	127.99 (16)
H6C—C6—H6D	108.0	N20—C21—C17	107.53 (15)
C8—C7—C6	112.76 (17)	C19—C22—H22A	109.5
C8—C7—H7A	109.0	C19—C22—H22B	109.5
C6—C7—H7A	109.0	H22A—C22—H22B	109.5
C8—C7—H7B	109.0	C19—C22—H22C	109.5
C6—C7—H7B	109.0	H22A—C22—H22C	109.5
H7A—C7—H7B	107.8	H22B—C22—H22C	109.5
C7—C8—C9	110.26 (15)	C19—N23—C24	116.12 (13)
C7—C8—C11	111.46 (16)	C19—N23—H23	105.2 (16)
C9—C8—C11	111.30 (17)	C24—N23—H23	110.9 (16)
C7—C8—H8	107.9	N23—C24—C26	111.45 (14)
C9—C8—H8	107.9	N23—C24—C25	116.68 (14)
C11—C8—H8	107.9	C26—C24—C25	108.14 (15)
C8—C9—C10	111.19 (16)	N23—C24—H24	106.7
C8—C9—H9A	109.4	C26—C24—H24	106.7
C10—C9—H9A	109.4	C25—C24—H24	106.7
C8—C9—H9B	109.4	C24—C25—S1	114.15 (13)
C10—C9—H9B	109.4	C24—C25—H25A	108.7
H9A—C9—H9B	108.0	S1—C25—H25A	108.7
C1—C10—C9	113.46 (17)	C24—C25—H25B	108.7
C1—C10—C5	112.26 (15)	S1—C25—H25B	108.7
C9—C10—C5	110.19 (15)	H25A—C25—H25B	107.6
C1—C10—H10	106.8	O3—C26—N27	123.66 (16)
C9—C10—H10	106.8	O3—C26—C24	120.28 (15)
C5—C10—H10	106.8	N27—C26—C24	116.03 (15)

C8—C11—H11A	109.5	C26—N27—C28	121.17 (16)
C8—C11—H11B	109.5	C26—N27—H27	119.1 (15)
H11A—C11—H11B	109.5	C28—N27—H27	119.7 (15)
C8—C11—H11C	109.5	N27—C28—C29	113.45 (15)
H11A—C11—H11C	109.5	N27—C28—H28A	108.9
H11B—C11—H11C	109.5	C29—C28—H28A	108.9
C13—C12—C15	122.74 (17)	N27—C28—H28B	108.9
C13—C12—C3	120.07 (17)	C29—C28—H28B	108.9
C15—C12—C3	117.16 (16)	H28A—C28—H28B	107.7
C12—C13—C14	125.12 (18)	O4—C29—O5	124.80 (17)
C12—C13—H13	117.4	O4—C29—C28	124.75 (16)
C14—C13—H13	117.4	O5—C29—C28	110.43 (16)

g) Hydrogen-bond geometry (Å, º) for compound **1**

D—H···*A*	*D*—H	H···*A*	*D*···*A*	*D*—H···*A*
C4—H4···O2	1.00	2.55	3.121 (2)	116
O5—H5···O6	0.82 (3)	1.77 (2)	2.5745 (19)	165 (3)
O6—H6*A*···O2^{i}	0.86 (3)	1.88 (3)	2.745 (2)	179 (3)
O6—H6*B*···O3^{ii}	0.91 (3)	1.88 (3)	2.774 (2)	165 (3)
N20—H20···O4^{iii}	0.84 (2)	2.08 (2)	2.906 (2)	172 (2)
C24—H24···O2^{iv}	1.00	2.31	3.260 (2)	158
N27—H27···O4^{v}	0.88 (3)	2.31 (3)	3.111 (2)	151 (2)

Symmetry codes: (i) -*x*+1, *y*+3/2, -*z*+3/2; (ii) -*x*, *y*+1/2, -*z*+3/2; (iii) *x*, *y*-1, *z*; (iv) *x*, *y*+1, *z*; (v) -*x*+1, *y*-1/2, -*z*+3/2.

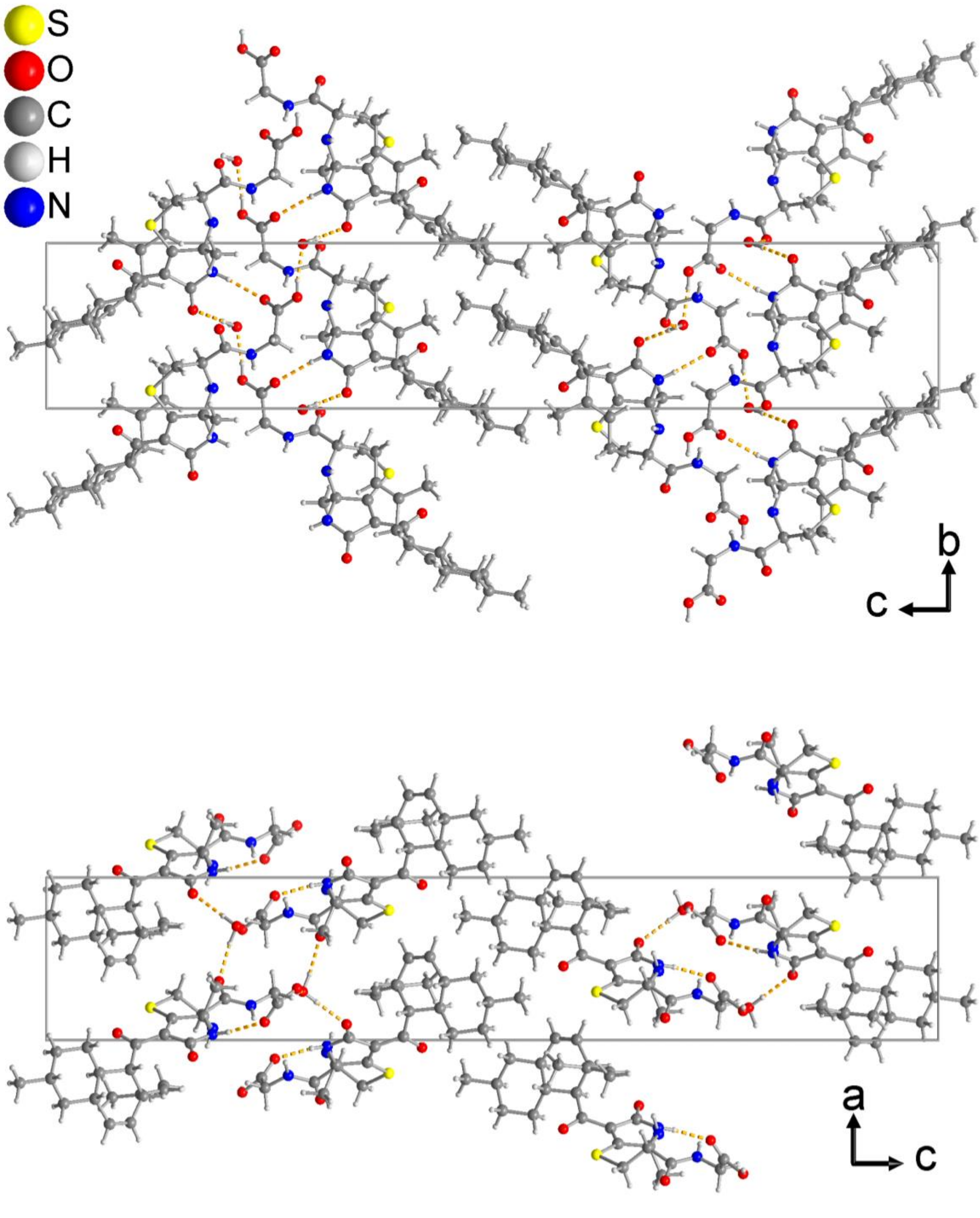

Figure S1. Unit cell packing diagram for compound **1** along two different viewing directions, showing the intermolecular hydrogen bonds as dashed orange lines.

Note that the polar and non-polar part of the molecule arrange with its kind in two-dimensional slabs parallel to the *ab*-plane.

ECD and NMR Calculations

Table S6. Comparison of the experimental ^{13}C NMR data of **1** measured in DMSO-d_6 with the mPW1PW91/6-311+G(2d,p) // B3LYP/6-31+G(d,p) ones of (3*R*,4*S*,5*R*,8*R*,10*S*,19*R*,24*R*)-**1** [*RR*], (3*R*,4*S*,5*R*,8*R*,10*S*,19*R*,24*S*)-**1** [*RS*], (3*R*,4*S*,5*R*,8*R*,10*S*,19*S*,24*R*)-**1** [*SR*] and (3*R*,4*S*,5*R*,8*R*,10*S*,19*S*,24*S*)-**1** [*SS*] corrected for DMSO.

For the MAE all carbons were considered while for the corrected MAE the carbons next to the heavy atom S (C18 and C-25) were neglected.

No.	Experimental	$Calc_{RR}$	$Calc_{RS}$	$Calc_{SR}$	$Calc_{SS}$	$\Delta\delta_{RR}$	$\Delta\delta_{RS}$	$\Delta\delta_{SR}$	$\Delta\delta_{SS}$
C-1	132.30	134.67	134.99	134.40	135.08	2.37	2.69	2.10	2.78
C-2	128.10	132.34	132.09	132.85	131.76	4.24	3.99	4.75	3.66
C-3	46.60	51.08	52.24	51.33	51.40	4.48	5.64	4.73	4.80
C-4	51.70	53.30	53.26	54.52	53.54	1.60	1.56	2.82	1.84
C-5	36.20	37.76	37.75	37.64	37.54	1.56	1.55	1.44	1.34
C-6	29.80	30.11	29.82	30.10	30.03	0.31	0.02	0.30	0.23
C-7	35.10	35.00	35.31	34.84	35.74	0.10	0.21	0.26	0.64
C-8	32.70	34.85	34.95	35.36	35.07	2.15	2.25	2.66	2.37
C-9	41.50	41.35	41.45	41.68	41.73	0.15	0.05	0.18	0.23
C-10	40.70	42.05	42.09	41.91	41.70	1.35	1.39	1.21	1.00
C-11	22.50	21.84	21.48	21.64	21.62	0.66	1.02	0.86	0.88
C-12	135.30	142.94	142.49	142.77	142.65	7.64	7.19	7.47	7.35
C-13	122.50	124.27	123.81	124.41	123.57	1.77	1.31	1.91	1.07
C-14	14.10	13.17	13.35	13.16	13.06	0.93	0.75	0.94	1.04
C-15	15.40	12.88	12.51	12.84	12.99	2.52	2.89	2.56	2.41
C-16	196.60	200.67	199.46	199.81	200.46	4.07	2.86	3.21	3.86
C-17	123.00	123.67	130.09	130.34	123.83	0.67	7.09	7.34	0.83
C-18	174.90	180.11	176.67	176.18	180.01	5.21	1.77	1.28	5.11
C-19	71.20	71.44	72.38	72.49	71.70	0.24	1.18	1.29	0.50
C-21	167.00	167.04	166.18	166.34	166.99	0.04	0.82	0.66	0.01
C-22	29.30	28.16	27.12	28.05	27.80	1.14	2.18	1.25	1.50
C-24	53.20	54.81	54.96	54.91	54.37	1.61	1.76	1.71	1.17
C-25	26.10	31.05	34.93	34.45	30.85	4.95	8.83	8.35	4.75
C-26	171.90	170.09	169.15	169.12	170.14	1.81	2.75	2.78	1.76
C-28	41.20	41.59	40.59	40.65	41.68	0.39	0.61	0.55	0.48
C-29	171.10	171.74	171.94	171.83	171.80	0.64	0.84	0.73	0.70
MAE						2.02	2.43	2.44	2.01
Corr. MAE						1.77	2.19	2.24	1.77

Table S7. Comparison of the experimental ^{13}C NMR data of **1** measured in MeOH-d_4 with the mPW1PW91/6-311+G(2d,p) // B3LYP/6-31+G(d,p) ones of (3*R*,4*S*,5*R*,8*R*,10*S*,19*R*,24*R*)-**1** [*RR*], (3*R*,4*S*,5*R*,8*R*,10*S*,19*R*,24*S*)-**1** [*RS*], (3*R*,4*S*,5*R*,8*R*,10*S*,19*S*,24*R*)-**1** [*SR*] and (3*R*,4*S*,5*R*,8*R*,10*S*,19*S*,24*S*)-**1** [*SS*] corrected for MeOH.

For the MAE all carbons were considered while for the corrected MAE the carbons next to the heavy atom S (C18 and C-25) were neglected.

No.	Experimental	$Calc_{RR}$	$Calc_{RS}$	$Calc_{SR}$	$Calc_{SS}$	$\Delta\delta_{RR}$	$\Delta\delta_{RS}$	$\Delta\delta_{SR}$	$\Delta\delta_{SS}$
C-1	133.50	136.23	136.55	135.95	136.64	2.73	3.05	2.45	3.14
C-2	129.20	133.87	133.62	134.39	133.28	4.67	4.42	5.19	4.08
C-3	48.50	51.88	53.05	52.13	52.20	3.38	4.55	3.63	3.70
C-4	53.90	54.12	54.08	55.35	54.36	0.22	0.18	1.45	0.46
C-5	38.10	38.44	38.43	38.32	38.22	0.34	0.33	0.22	0.12
C-6	31.40	30.72	30.43	30.71	30.64	0.68	0.97	0.69	0.76
C-7	36.60	35.65	35.97	35.49	36.40	0.95	0.63	1.11	0.20
C-8	34.40	35.50	35.60	36.01	35.72	1.10	1.20	1.61	1.32
C-9	43.20	42.06	42.16	42.40	42.44	1.14	1.04	0.80	0.76
C-10	42.40	42.77	42.80	42.63	42.41	0.37	0.40	0.23	0.01
C-11	23.00	22.38	22.01	22.18	22.16	0.62	0.99	0.82	0.84
C-12	137.20	144.57	144.12	144.40	144.28	7.37	6.92	7.20	7.08
C-13	123.90	125.73	125.27	125.88	125.02	1.83	1.37	1.98	1.12
C-14	14.20	13.63	13.81	13.61	13.51	0.57	0.39	0.59	0.69
C-15	15.60	13.34	12.96	13.29	13.45	2.26	2.64	2.31	2.15
C-16	199.40	202.82	201.60	201.95	202.61	3.42	2.20	2.55	3.21
C-17	124.30	125.12	131.60	131.86	125.28	0.82	7.30	7.56	0.98
C-18	177.80	182.07	178.60	178.11	181.98	4.27	0.80	0.31	4.18
C-19	72.90	72.42	73.37	73.48	72.68	0.48	0.47	0.58	0.22
C-21	169.60	168.89	168.02	168.18	168.84	0.71	1.58	1.42	0.76
C-22	29.10	28.75	27.70	28.64	28.39	0.35	1.40	0.46	0.71
C-24	54.50	55.65	55.79	55.75	55.20	1.15	1.29	1.25	0.70
C-25	27.20	31.67	35.59	35.10	31.46	4.47	8.39	7.90	4.26
C-26	174.90	171.97	171.02	170.99	172.02	2.93	3.88	3.91	2.88
C-28	41.90	42.30	41.30	41.35	42.39	0.40	0.60	0.55	0.49
C-29	172.90	173.63	173.83	173.72	173.69	0.73	0.93	0.82	0.79
MAE						1.84	2.23	2.21	1.75
Corr. MAE						1.63	2.03	2.06	1.55

Table S8. Comparison of the experimental ^{13}C NMR data of **2** measured in DMSO-d_6 with the mPW1PW91/6-311+G(2d,p) // B3LYP/6-31+G(d,p) ones of (3*R*,4*S*,5*R*,8*R*,10*S*,19*R*,24*R*)-**2** [*RR*], (3*R*,4*S*,5*R*,8*R*,10*S*,19*R*,24*S*)-**2** [*RS*], (3*R*,4*S*,5*R*,8*R*,10*S*,19*S*,24*R*)-**2** [*SR*] , and (3*R*,4*S*,5*R*,8*R*,10*S*,19*S*,24*S*)-**2** [*SS*] corrected for DMSO.

For the MAE all carbons were considered while for the corrected MAE the carbons next to the heavy atom S (C18 and C-25) were neglected.

No.	Experimental	$Calc_{RR}$	$Calc_{RS}$	$Calc_{SR}$	$Calc_{SS}$	$\Delta\delta_{RR}$	$\Delta\delta_{RS}$	$\Delta\delta_{SR}$	$\Delta\delta_{SS}$
C-1	132.00	134.67	134.99	134.40	135.08	2.67	2.99	2.40	3.08
C-2	128.10	132.34	132.09	132.85	131.76	4.24	3.99	4.75	3.66
C-3	46.00	51.08	52.24	51.33	51.40	5.08	6.24	5.33	5.40
C-4	51.80	53.30	53.26	54.52	53.54	1.50	1.46	2.72	1.74
C-5	36.50	37.76	37.75	37.64	37.54	1.26	1.25	1.14	1.04
C-6	29.50	30.11	29.82	30.10	30.03	0.61	0.32	0.60	0.53
C-7	35.00	35.00	35.31	34.84	35.74	0.00	0.31	0.16	0.74
C-8	32.50	34.85	34.95	35.36	35.07	2.35	2.45	2.86	2.57
C-9	41.50	41.35	41.45	41.68	41.73	0.15	0.05	0.18	0.23
C-10	40.40	42.05	42.09	41.91	41.70	1.65	1.69	1.51	1.30
C-11	22.40	21.84	21.48	21.64	21.62	0.56	0.92	0.76	0.78
C-12	136.10	142.94	142.49	142.77	142.65	6.84	6.39	6.67	6.55
C-13	121.60	124.27	123.81	124.41	123.57	2.67	2.21	2.81	1.97
C-14	13.30	13.17	13.35	13.16	13.06	0.13	0.05	0.14	0.24
C-15	15.40	12.88	12.51	12.84	12.99	2.52	2.89	2.56	2.41
C-16	196.70	200.67	199.46	199.81	200.46	3.97	2.76	3.11	3.76
C-17	124.70	123.67	130.09	130.34	123.83	1.03	5.39	5.64	0.87
C-18	172.50	180.11	176.67	176.18	180.01	7.61	4.17	3.68	7.51
C-19	70.70	71.44	72.38	72.49	71.70	0.74	1.68	1.79	1.00
C-21	166.40	167.04	166.18	166.34	166.99	0.64	0.22	0.06	0.59
C-22	29.60	28.16	27.12	28.05	27.80	1.44	2.48	1.55	1.80
C-24	51.70	54.81	54.96	54.91	54.37	3.11	3.26	3.21	2.67
C-25	27.00	31.05	34.93	34.45	30.85	4.05	7.93	7.45	3.85
C-26	171.20	170.09	169.15	169.12	170.14	1.11	2.05	2.08	1.06
C-28	41.10	41.59	40.59	40.65	41.68	0.49	0.51	0.45	0.58
C-29	170.60	171.74	171.94	171.83	171.80	1.14	1.34	1.23	1.20
MAE						2.21	2.50	2.49	2.20
Corr. MAE						1.91	2.20	2.24	1.91

Table S9. Comparison of the experimental ^{13}C NMR data of **2** measured in MeOH-d_4 with the mPW1PW91/6-311+G(2d,p) // B3LYP/6-31+G(d,p) ones of (3*R*,4*S*,5*R*,8*R*,10*S*,19*R*,24*R*)-**2** [*RR*], (3*R*,4*S*,5*R*,8*R*,10*S*,19*R*,24*S*)-**2** [*RS*], (3*R*,4*S*,5*R*,8*R*,10*S*,19*S*,24*R*)-**2** [*SR*] and (3*R*,4*S*,5*R*,8*R*,10*S*,19*S*,24*S*)-**2** [*SS*] corrected for MeOH.

For the MAE all carbons were considered while for the corrected MAE the carbons next to the heavy atom S (C18 and C-25) were neglected.

No.	Experimental	$Calc_{RR}$	$Calc_{RS}$	$Calc_{SR}$	$Calc_{SS}$	$\Delta\delta_{RR}$	$\Delta\delta_{RS}$	$\Delta\delta_{SR}$	$\Delta\delta_{SS}$
C-1	133.30	136.23	136.55	135.95	136.64	2.93	3.25	2.65	3.34
C-2	129.40	133.87	133.62	134.39	133.28	4.47	4.22	4.99	3.88
C-3	48.10	51.88	53.05	52.13	52.20	3.78	4.95	4.03	4.10
C-4	54.10	54.12	54.08	55.35	54.36	0.02	0.02	1.25	0.26
C-5	38.50	38.44	38.43	38.32	38.22	0.06	0.07	0.18	0.28
C-6	31.30	30.72	30.43	30.71	30.64	0.58	0.87	0.59	0.66
C-7	36.50	35.65	35.97	35.49	36.40	0.85	0.53	1.01	0.10
C-8	34.40	35.50	35.60	36.01	35.72	1.10	1.20	1.61	1.32
C-9	43.20	42.06	42.16	42.40	42.44	1.14	1.04	0.80	0.76
C-10	42.10	42.77	42.80	42.63	42.41	0.67	0.70	0.53	0.31
C-11	23.00	22.38	22.01	22.18	22.16	0.62	0.99	0.82	0.84
C-12	137.80	144.57	144.12	144.40	144.28	6.77	6.32	6.60	6.48
C-13	123.50	125.73	125.27	125.88	125.02	2.23	1.77	2.38	1.52
C-14	13.60	13.63	13.81	13.61	13.51	0.03	0.21	0.01	0.09
C-15	15.90	13.34	12.96	13.29	13.45	2.56	2.94	2.61	2.45
C-16	199.70	202.82	201.60	201.95	202.61	3.12	1.90	2.25	2.91
C-17	127.20	125.12	131.60	131.86	125.28	2.08	4.40	4.66	1.92
C-18	174.70	182.07	178.60	178.11	181.98	7.37	3.90	3.41	7.28
C-19	72.30	72.42	73.37	73.48	72.68	0.12	1.07	1.18	0.38
C-21	168.90	168.89	168.02	168.18	168.84	0.01	0.88	0.72	0.06
C-22	31.50	28.75	27.70	28.64	28.39	2.75	3.80	2.86	3.11
C-24	54.20	55.65	55.79	55.75	55.20	1.45	1.59	1.55	1.00
C-25	29.00	31.67	35.59	35.10	31.46	2.67	6.59	6.10	2.46
C-26	173.90	171.97	171.02	170.99	172.02	1.93	2.88	2.91	1.88
C-28	42.20	42.30	41.30	41.35	42.39	0.10	0.90	0.85	0.19
C-29	172.30	173.63	173.83	173.72	173.69	1.33	1.53	1.42	1.39
MAE						1.95	2.25	2.23	1.88
Corr. MAE						1.70	2.00	2.02	1.64

Table S10. Comparison of the experimental ^{13}C NMR data of **3** measured in DMSO-d_6 with the mPW1PW91/6-311+G(2d,p) // B3LYP/6-31+G(d,p) ones of (3*R*,4*S*,5*R*,8*R*,10*S*,19*R*,24*R*)-**3** [*RR*], (3*R*,4*S*,5*R*,8*R*,10*S*,19*R*,24*S*)-**3** [*RS*], (3*R*,4*S*,5*R*,8*R*,10*S*,19*S*,24*R*)-**3** [*SR*] and (3*R*,4*S*,5*R*,8*R*,10*S*,19*S*,24*S*)-**3** [*SS*] corrected for DMSO.

For the MAE all carbons were considered while for the corrected MAE the carbons next to the heavy atom S (C19 and C-25) were neglected.

No.	Experimental	$Calc_{RR}$	$Calc_{RS}$	$Calc_{SR}$	$Calc_{SS}$	$\Delta\delta_{RR}$	$\Delta\delta_{RS}$	$\Delta\delta_{SR}$	$\Delta\delta_{SS}$
C-1	132.00	134.96	134.84	134.60	134.92	2.96	2.84	2.60	2.92
C-2	128.30	131.88	132.60	132.58	131.93	3.58	4.30	4.28	3.63
C-3	46.40	51.22	51.18	50.88	51.60	4.82	4.78	4.48	5.20
C-4	51.00	52.94	52.76	52.69	52.98	1.94	1.76	1.69	1.98
C-5	36.10	37.98	38.38	37.67	38.36	1.88	2.28	1.57	2.26
C-6	29.90	29.83	30.01	29.95	30.02	0.07	0.11	0.05	0.12
C-7	35.10	35.58	35.28	35.66	35.37	0.48	0.18	0.56	0.27
C-8	32.60	34.71	35.42	34.90	34.42	2.11	2.82	2.30	1.82
C-9	41.60	41.56	41.33	41.47	41.60	0.04	0.27	0.13	0.00
C-10	40.70	41.92	42.12	41.94	42.10	1.22	1.42	1.24	1.40
C-11	22.40	21.48	21.48	21.43	21.62	0.92	0.92	0.97	0.78
C-12	136.00	142.24	143.11	142.00	144.00	6.24	7.11	6.00	8.00
C-13	121.40	123.88	124.71	125.31	125.98	2.48	3.31	3.91	4.58
C-14	13.40	13.63	13.50	13.51	13.40	0.23	0.10	0.11	0.00
C-15	15.30	12.74	13.15	12.95	12.54	2.56	2.15	2.35	2.76
C-16	197.80	205.60	205.01	204.78	205.45	7.80	7.21	6.98	7.65
C-17	99.80	104.86	105.28	104.64	105.07	5.06	5.48	4.84	5.27
C-18	171.30	174.97	172.04	172.20	174.86	3.67	0.74	0.90	3.56
C-19	60.40	62.69	64.94	64.48	62.24	2.29	4.54	4.08	1.84
C-21	168.80	167.04	165.67	165.80	167.07	1.76	3.13	3.00	1.73
C-22	29.40	25.78	31.07	31.23	26.00	3.62	1.67	1.83	3.40
C-24	51.10	61.58	54.79	54.72	61.72	10.48	3.69	3.62	10.62
C-25	29.20	34.14	33.47	33.27	33.85	4.94	4.27	4.07	4.65
C-26	167.40	168.91	166.47	166.46	168.62	1.51	0.93	0.94	1.22
C-28	42.40	40.31	42.97	43.18	40.13	2.09	0.57	0.78	2.27
C-29	170.50	172.17	170.12	170.15	172.04	1.67	0.38	0.35	1.54
MAE						2.94	2.58	2.45	3.06
Corr. MAE						2.88	2.42	2.31	3.04

Table S11. Comparison of the experimental ^{13}C NMR data of **3** measured in MeOH-d_4 with the mPW1PW91/6-311+G(2d,p) // B3LYP/6-31+G(d,p) ones of (3*R*,4*S*,5*R*,8*R*,10*S*,19*R*,24*R*)-**3** [*RR*], (3*R*,4*S*,5*R*,8*R*,10*S*,19*R*,24*S*)-**3** [*RS*], (3*R*,4*S*,5*R*,8*R*,10*S*,19*S*,24*R*)-**3** [*SR*] and (3*R*,4*S*,5*R*,8*R*,10*S*,19*S*,24*S*)-**3** [*SS*] corrected for MeOH.

For the MAE all carbons were considered while for the corrected MAE the carbons next to the heavy atom S (C19 and C-25) were neglected.

No.	Experimental	$Calc_{RR}$	$Calc_{RS}$	$Calc_{SR}$	$Calc_{SS}$	$\Delta\delta_{RR}$	$\Delta\delta_{RS}$	$\Delta\delta_{SR}$	$\Delta\delta_{SS}$
C-1	133.30	134.96	134.84	134.60	134.92	1.66	1.54	1.30	1.62
C-2	129.60	131.88	132.60	132.58	131.93	2.28	3.00	2.98	2.33
C-3	48.40	51.22	51.18	50.88	51.60	2.82	2.78	2.48	3.20
C-4	53.30	52.94	52.76	52.69	52.98	0.36	0.54	0.61	0.32
C-5	37.90	37.98	38.38	37.67	38.36	0.08	0.48	0.23	0.46
C-6	31.60	29.83	30.01	29.95	30.02	1.77	1.59	1.65	1.58
C-7	36.50	35.58	35.28	35.66	35.37	0.92	1.22	0.84	1.13
C-8	34.50	34.71	35.42	34.90	34.42	0.21	0.92	0.40	0.08
C-9	43.30	41.56	41.33	41.47	41.60	1.74	1.97	1.83	1.70
C-10	42.50	41.92	42.12	41.94	42.10	0.58	0.38	0.56	0.40
C-11	23.00	21.48	21.48	21.43	21.62	1.52	1.52	1.57	1.38
C-12	137.60	142.24	143.11	142.00	144.00	4.64	5.51	4.40	6.40
C-13	123.30	123.88	124.71	125.31	125.98	0.58	1.41	2.01	2.68
C-14	13.90	13.63	13.50	13.51	13.40	0.27	0.40	0.39	0.50
C-15	15.80	12.74	13.15	12.95	12.54	3.06	2.65	2.85	3.26
C-16	201.10	205.60	205.01	204.78	205.45	4.50	3.91	3.68	4.35
C-17	101.70	104.86	105.28	104.64	105.07	3.16	3.58	2.94	3.37
C-18	173.60	174.97	172.04	172.20	174.86	1.37	1.56	1.40	1.26
C-19	61.70	62.69	64.94	64.48	62.24	0.99	3.24	2.78	0.54
C-21	171.90	167.04	165.67	165.80	167.07	4.86	6.23	6.10	4.83
C-22	30.20	25.78	31.07	31.23	26.00	4.42	0.87	1.03	4.20
C-24	53.60	61.58	54.79	54.72	61.72	7.98	1.19	1.12	8.12
C-25	30.50	34.14	33.47	33.27	33.85	3.64	2.97	2.77	3.35
C-26	169.50	168.91	166.47	166.46	168.62	0.59	3.03	3.04	0.88
C-28	43.10	40.31	42.97	43.18	40.13	2.79	0.13	0.08	2.97
C-29	174.00	172.17	170.12	170.15	172.04	1.83	3.88	3.85	1.96
MAE						2.25	2.17	2.03	2.42
Corr. MAE						2.25	2.10	1.97	2.46

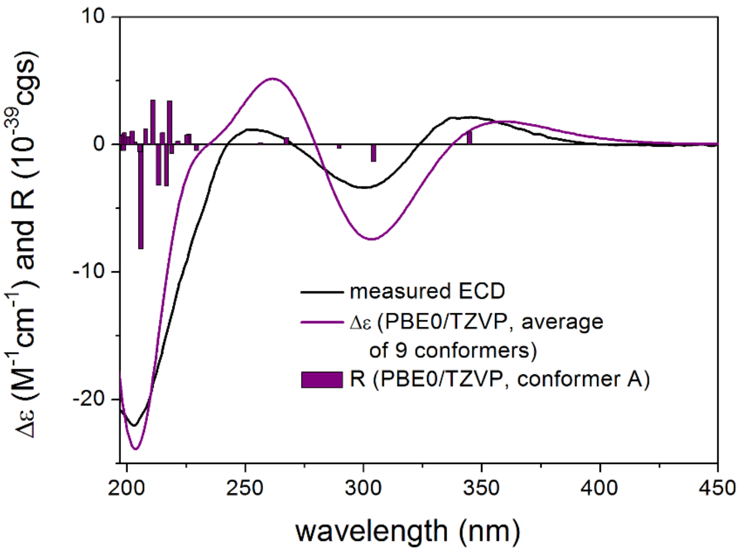

Figure S2. Experimental ECD spectrum of compound **1** in MeOH (black line) compared with the calculated PBE0/TZVP PCM/MeOH spectrum of (3*R*,4*S*,5*R*,8*R*,10*S*,19*R*,24*S*)-**1**. Level of DFT optimization: ωB97X/TZVP PCM/MeOH. Bars represent the rotational strength values of conformer A.

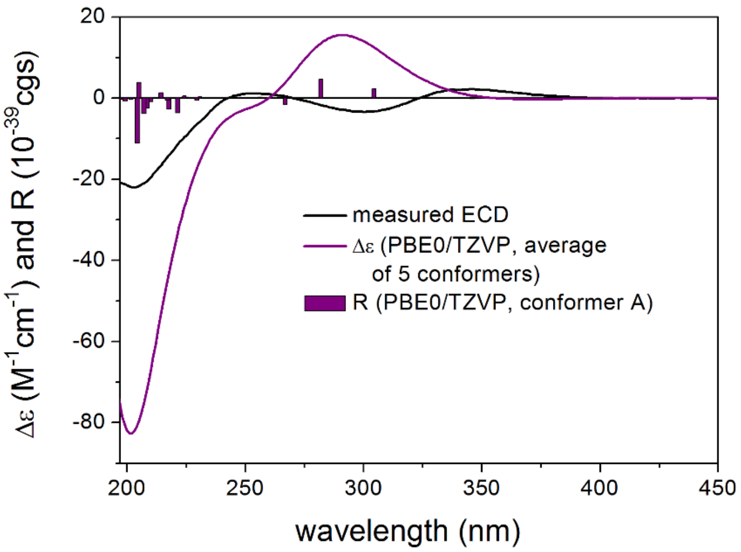

Figure S3. Experimental ECD spectrum of compound **1** in MeOH (black line) compared with the calculated PBE0/TZVP PCM/MeOH spectrum of (3*R*,4*S*,5*R*,8*R*,10*S*,19*S*,24*R*)-**1**. Level of DFT optimization: ωB97X/TZVP PCM/MeOH. Bars represent the rotational strength values of conformer A.

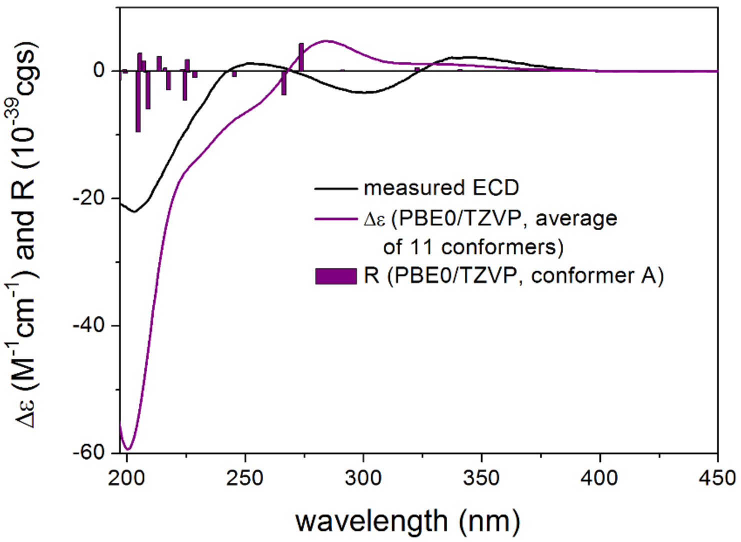

Figure S4. Experimental ECD spectrum of compound **1** in MeOH (black line) compared with the calculated PBE0/TZVP PCM/MeOH spectrum of (3*R*,4*S*,5*R*,8*R*,10*S*,19*S*,24*S*)-**1**. Level of DFT optimization: ωB97X/TZVP PCM/MeOH. Bars represent the rotational strength values of conformer A.

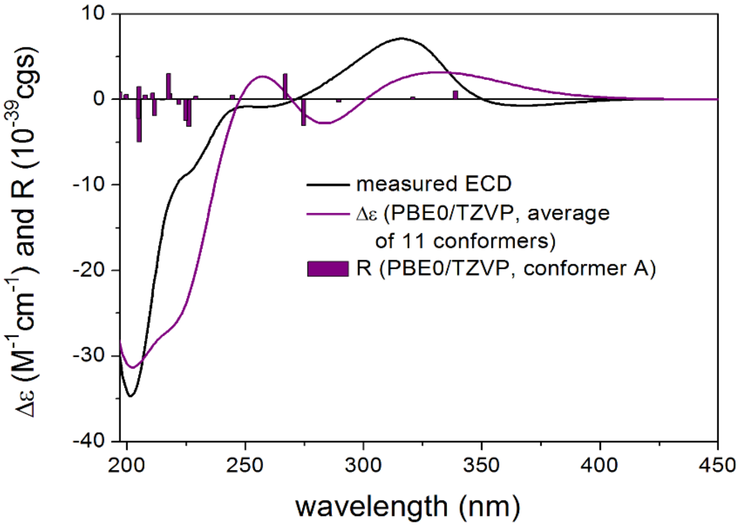

Figure S5. Experimental ECD spectrum of compound **2** in MeOH (black line) compared with the calculated PBE0/TZVP PCM/MeOH spectrum of (3*R*,4*S*,5*R*,8*R*,10*S*,19*R*,24*R*)-**2**. Level of DFT optimization: ωB97X/TZVP PCM/MeOH. Bars represent the rotational strength values of conformer A.

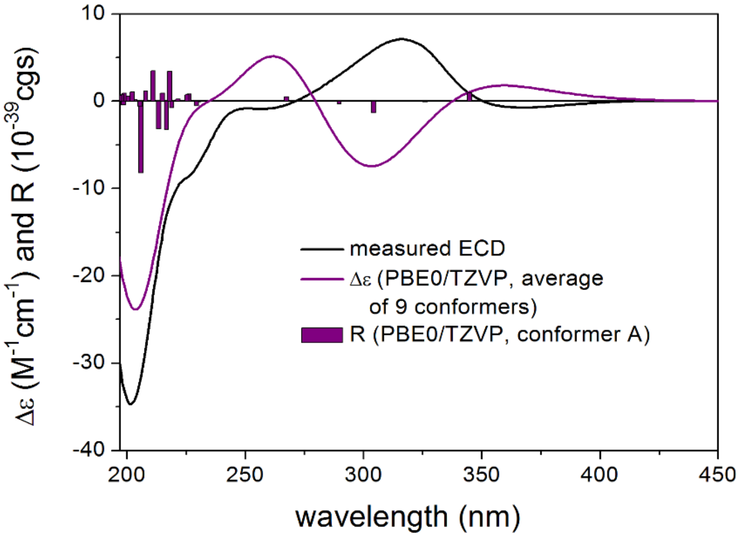

Figure S6. Experimental ECD spectrum of compound **2** in MeOH (black line) compared with the calculated PBE0/TZVP PCM/MeOH spectrum of (3*R*,4*S*,5*R*,8*R*,10*S*,19*R*,24*S*)-**2**. Level of DFT optimization: ωB97X/TZVP PCM/MeOH. Bars represent the rotational strength values of conformer A.

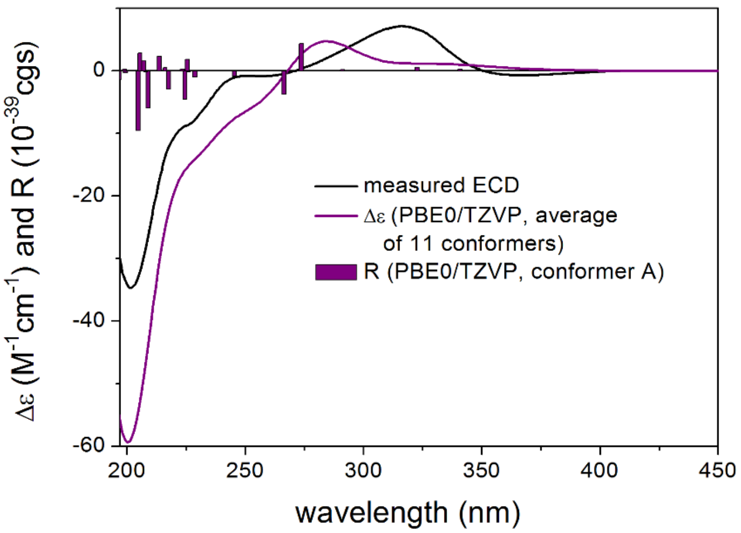

Figure S7. Experimental ECD spectrum of compound **2** in MeOH (black line) compared with the calculated PBE0/TZVP PCM/MeOH spectrum of (3*R*,4*S*,5*R*,8*R*,10*S*,19*S*,24*S*)-**2**. Level of DFT optimization: ωB97X/TZVP PCM/MeOH. Bars represent the rotational strength values of conformer A.

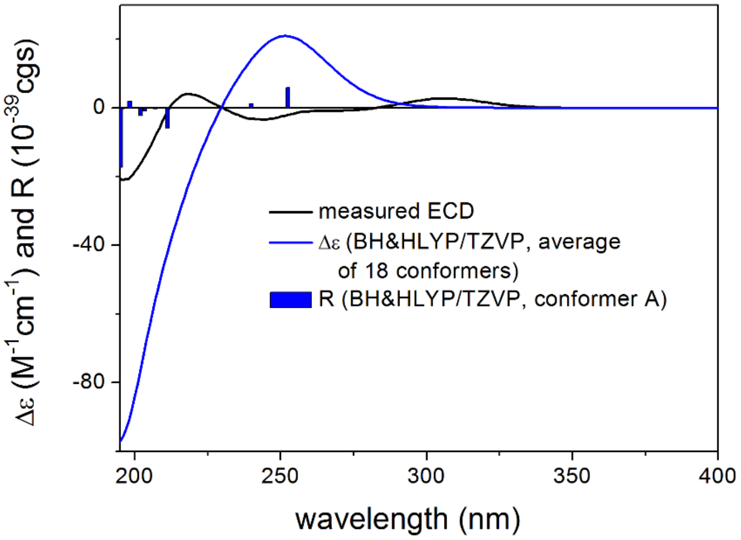

Figure S8. Experimental ECD spectrum of compound **3** in MeOH (black line) compared with the calculated BH&HLYP/TZVP PCM/MeOH spectrum of (3*R*,4*S*,5*R*,8*R*,10*S*,19*R*,24*R*)-**3**. Level of DFT optimization: ωB97X/TZVP PCM/MeOH. Bars represent the rotational strength values of conformer A.

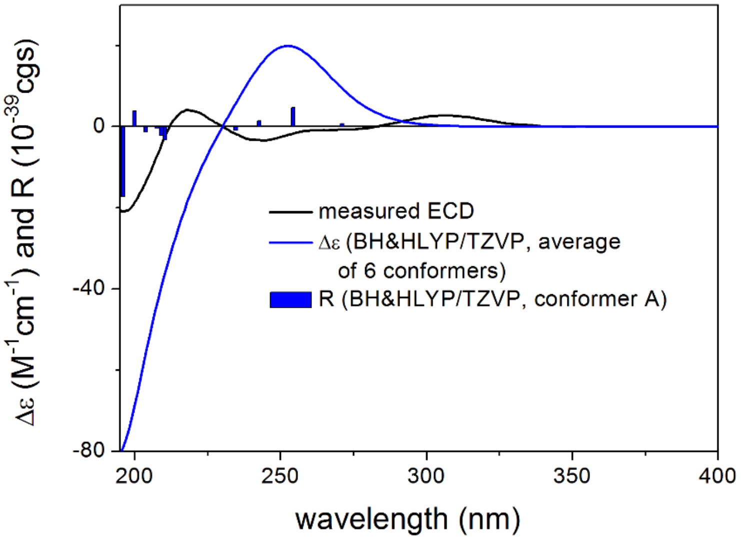

Figure S9. Experimental ECD spectrum of compound **3** in MeOH (black line) compared with the calculated BH&HLYP/TZVP PCM/MeOH spectrum of (3*R*,4*S*,5*R*,8*R*,10*S*,19*R*,24*S*)-**3**. Level of DFT optimization: ωB97X/TZVP PCM/MeOH. Bars represent the rotational strength values of conformer A.

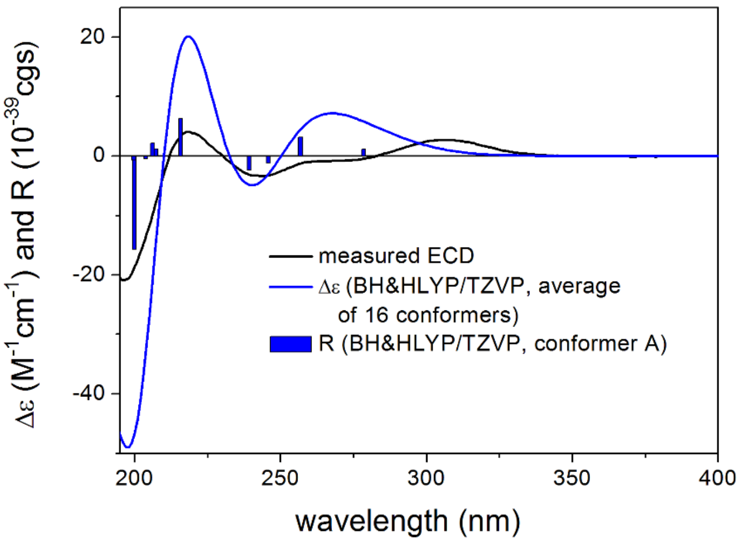

Figure S10. Experimental ECD spectrum of compound **3** in MeOH (black line) compared with the calculated BH&HLYP/TZVP PCM/MeOH spectrum of (3*R*,4*S*,5*R*,8*R*,10*S*,19*S*,24*S*)-**3**. Level of DFT optimization: ωB97X/TZVP PCM/MeOH. Bars represent the rotational strength values of conformer A.

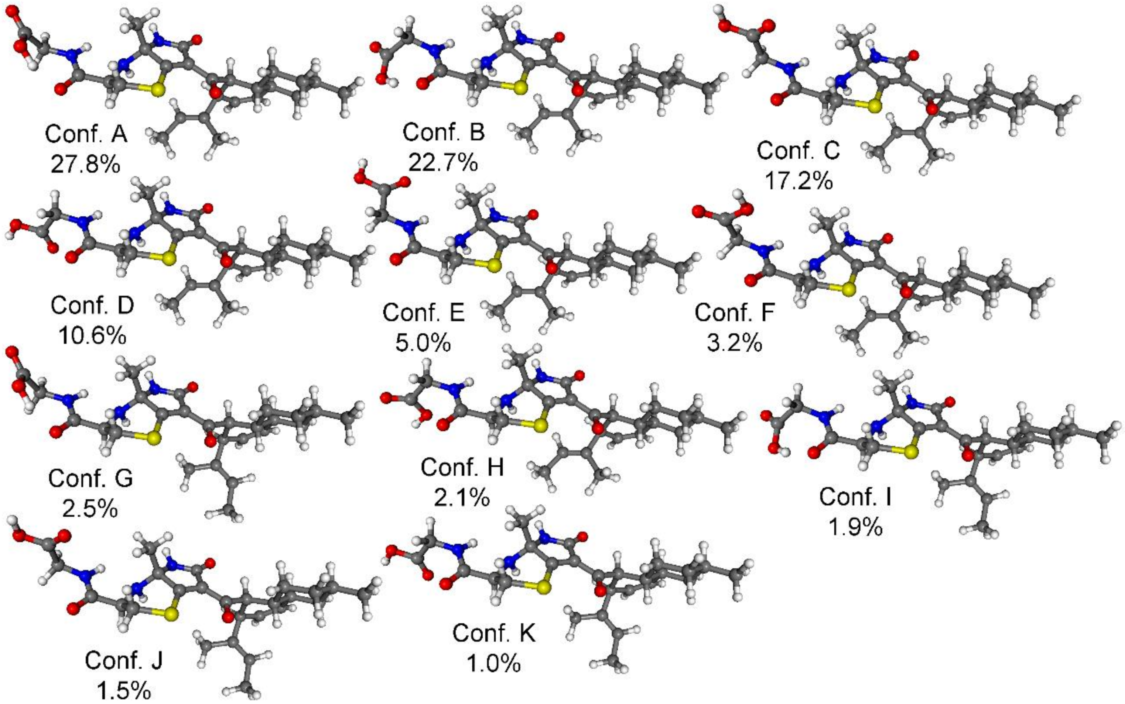

Figure S11. Structure and population of the low-energy ωB97X/TZVP PCM/MeOH conformers (≥ 1%) of (3*R*,4*S*,5*R*,8*R*,10*S*,19*R*,24*R*)-**1**.

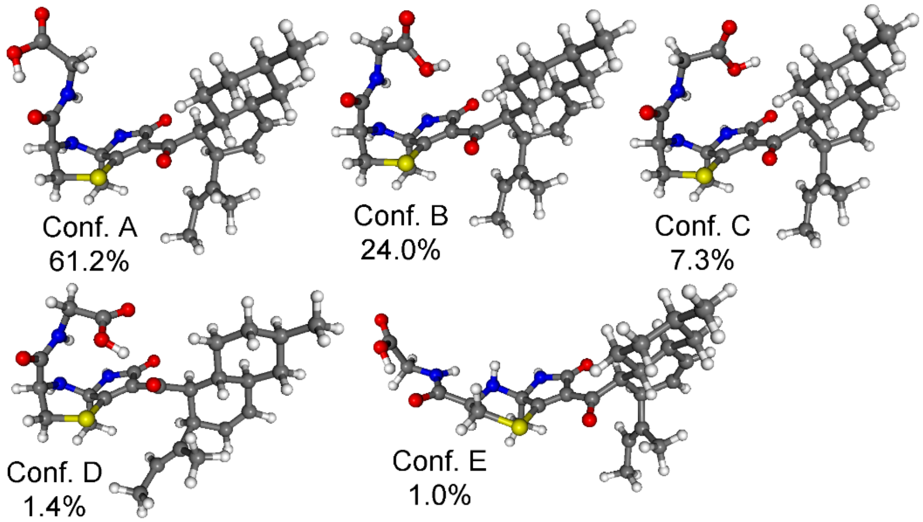

Figure S12. Structure and population of the low-energy ωB97X/TZVP PCM/MeOH conformers (≥ 1%) of (3*R*,4*S*,5*R*,8*R*,10*S*,19*S*,24*R*)-**2**.

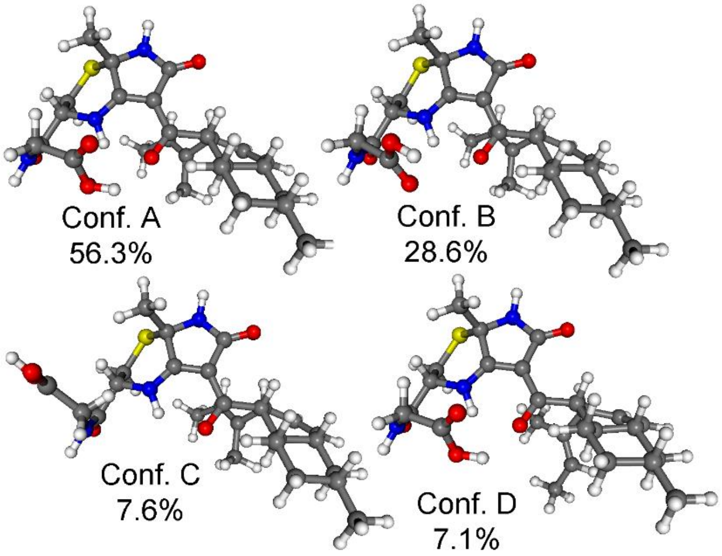

Figure S13. Structure and population of the low-energy ωB97X/TZVP PCM/MeOH conformers (≥ 1%) of (3*R*,4*S*,5*R*,8*R*,10*S*,19*S*,24*R*)-**3**.

Table S12. Cartesian coordinates and energies of the low-energy conformers calculated at the B3LYP/6-31+G(d,p) level.

(3*R*,4*S*,5*R*,8*R*,10*S*,19*R*,24*R*)-**1**, Conf A

C	-5.479025	-2.215988	1.231353
C	-6.677335	-2.007299	0.287321
C	-6.652672	-0.585669	-0.302572
C	-5.302558	-0.264659	-0.970325
C	-4.146478	-0.407681	0.043629
C	-4.131860	-1.849795	0.585982
C	-5.289393	1.080483	-1.649260
C	-4.206477	1.861237	-1.721256
C	-2.846434	1.517370	-1.151172
C	-2.822621	0.029314	-0.620513
C	-2.378210	2.603743	-0.172670
C	-1.287325	3.325542	-0.490191
C	-0.654250	4.455010	0.275687
C	-3.204105	2.822306	1.072799
C	-1.602748	-0.170151	0.266559
C	-8.009578	-2.311681	0.983520
C	-0.281249	-0.422624	-0.339704
O	-1.665628	-0.117786	1.497290
C	0.074608	-0.551194	-1.779336
N	1.465522	-0.612408	-1.834336
C	2.065834	-0.820244	-0.524225
C	0.869762	-0.531189	0.388012
O	-0.653498	-0.583632	-2.762071
N	3.164668	0.153365	-0.344022
C	3.556787	0.547517	1.026736
C	2.915461	-0.290135	2.145459
S	1.074848	-0.291379	2.086436
C	5.087906	0.515301	1.234909
N	5.809341	0.043156	0.194941
C	7.248426	-0.050403	0.248024
C	7.763561	-0.731425	-1.000008
O	9.107279	-0.842418	-0.979172
O	5.582872	0.898662	2.294984
O	7.073157	-1.129644	-1.914864
C	2.570743	-2.272092	-0.404041
H	-4.355178	0.261787	0.887377
H	-5.149834	-1.029337	-1.753240
H	-5.456930	-3.257990	1.576696
H	-5.622895	-1.595989	2.129172
H	-6.564940	-2.711939	-0.552406
H	-6.847746	0.147415	0.494776
H	-7.463896	-0.472978	-1.035131
H	-3.918014	-2.543705	-0.241550
H	-3.332634	-1.971233	1.322373
H	-6.215997	1.408512	-2.120830
H	-4.265150	2.823244	-2.229036
H	-2.139360	1.539697	-1.989295
H	-2.670471	-0.583788	-1.517154
H	-0.781659	3.078226	-1.425421
H	-1.187703	4.707200	1.194669
H	0.378925	4.206597	0.553535
H	-0.600939	5.361098	-0.342124
H	-3.073036	1.990569	1.774610
H	-2.936084	3.743375	1.594206
H	-4.269760	2.875081	0.823781
H	-8.033056	-3.338730	1.366207
H	-8.170092	-1.636291	1.833311
H	-8.855824	-2.190541	0.297110
H	1.891597	-1.012773	-2.661186
H	2.908864	0.978876	-0.877446
H	3.264784	1.590418	1.205503
H	3.296834	-1.313087	2.156911
H	3.174349	0.162462	3.103942
H	5.314127	-0.247341	-0.642776
H	7.576777	-0.614248	1.129736
H	7.717910	0.938592	0.331715
H	9.381231	-1.280305	-1.803278
H	1.745924	-2.967486	-0.581396
H	3.349914	-2.436083	-1.155050
H	2.997599	-2.491051	0.574979

B3LYP Energy = -1950.53951731 a.u.

(3*R*,4*S*,5*R*,8*R*,10*S*,19*R*,24*R*)-**1**, Conf B

C	5.370339	-2.467652	-1.065692
C	6.649096	-2.101705	-0.290405
C	6.673272	-0.594288	0.019531
C	5.389301	-0.134899	0.735304
C	4.145370	-0.449594	-0.124051
C	4.086283	-1.967490	-0.382645
C	5.434723	1.313745	1.146690
C	4.360535	2.109264	1.171324
C	2.954236	1.683124	0.805738
C	2.887476	0.122127	0.565528
C	2.390305	2.574982	-0.309797
C	1.335276	3.362550	-0.028846
C	0.630771	4.342916	-0.926628
C	3.090537	2.544426	-1.647366
C	1.591241	-0.220905	-0.151027
C	7.913229	-2.551009	-1.033465
C	0.331145	-0.329894	0.612168
O	1.534199	-0.403600	-1.369073
C	0.113451	-0.189335	2.077337
N	-1.268981	-0.204678	2.271316
C	-1.990196	-0.629815	1.080725
C	-0.883067	-0.542317	0.025592
O	0.929125	-0.065978	2.979126
N	-3.090039	0.332318	0.825474
C	-3.592420	0.489834	-0.557693

C	-3.078277	-0.561124	-1.556393
S	-1.241381	-0.613535	-1.663860
C	-5.130788	0.485170	-0.610985
N	-5.770252	0.263404	0.549546
C	-7.222210	0.339489	0.693255
C	-7.984532	-0.842061	0.062636
O	-7.784797	-1.030251	-1.249275
O	-5.719101	0.670102	-1.689531
O	-8.737061	-1.534785	0.708646
C	-2.525033	-2.062316	1.272824
H	4.272891	0.048068	-1.093516
H	5.311991	-0.735936	1.659386
H	5.319825	-3.555559	-1.204141
H	5.430211	-2.030667	-2.074012
H	6.616850	-2.633536	0.674164
H	6.790184	-0.027589	-0.916672
H	7.547823	-0.355559	0.640344
H	3.950666	-2.491126	0.576265
H	3.224287	-2.214040	-1.008826
H	6.400131	1.711424	1.460058
H	4.462420	3.148444	1.481621
H	2.329392	1.870448	1.687446
H	2.821341	-0.312064	1.570561
H	0.925137	3.303202	0.981054
H	-0.426924	4.072046	-1.046726
H	0.645836	5.349873	-0.489339
H	1.067139	4.405288	-1.925677
H	4.174344	2.638152	-1.517149
H	2.904317	1.594071	-2.160677
H	2.761812	3.349108	-2.307789
H	7.904602	-3.632025	-1.215144
H	7.994604	-2.050930	-2.006796
H	8.817889	-2.314936	-0.461032
H	-1.609326	-0.458820	3.190811
H	-2.766191	1.230444	1.173234
H	-3.295318	1.471371	-0.949974
H	-3.479989	-1.554083	-1.345461
H	-3.417320	-0.280919	-2.554973
H	-5.171829	0.102643	1.353879
H	-7.578374	1.269639	0.232720
H	-7.463647	0.356587	1.755053
H	-7.142729	-0.367499	-1.606612
H	-1.694919	-2.743713	1.476699
H	-3.212895	-2.071207	2.124468
H	-3.066518	-2.434596	0.403052

B3LYP Energy = -1950.53715613 a.u.

(3*R*,4*S*,5*R*,8*R*,10*S*,19*R*,24*R*)-**1**, Conf C

C	5.263582	1.849901	1.848346
C	6.425099	2.108030	0.871152
C	6.483415	0.998983	-0.194978
C	5.129052	0.818527	-0.905231
C	4.018034	0.478120	0.112908
C	3.916767	1.620207	1.141664
C	5.184915	-0.178786	-2.032898
C	4.188530	-1.019610	-2.329221
C	2.873846	-1.096540	-1.586780
C	2.713588	0.176179	-0.656235
C	2.678624	-2.458258	-0.900280
C	3.709856	-3.100050	-0.324490
C	3.708567	-4.418123	0.398711
C	1.275279	-3.014893	-0.975374
C	1.498745	0.036359	0.242011
C	7.762428	2.264521	1.605177
C	0.168714	0.463096	-0.241919
O	1.567786	-0.435728	1.379017
C	-0.199903	1.058423	-1.556811
N	-1.591563	1.117803	-1.581819
C	-2.185464	0.867161	-0.276572
C	-0.978816	0.306488	0.483038
O	0.519079	1.421330	-2.477460
N	-3.268559	-0.128114	-0.431061
C	-3.654688	-0.955684	0.732668
C	-3.007237	-0.540150	2.064259
S	-1.167310	-0.501233	1.998810
C	-5.185207	-0.997085	0.945073
N	-5.910826	-0.219614	0.112155
C	-7.350164	-0.153955	0.192273
C	-7.868393	0.899485	-0.761014
O	-9.212594	0.991288	-0.706926
O	-5.675569	-1.702710	1.826734
O	-7.179779	1.580876	-1.491573
C	-2.710886	2.187032	0.322751
H	4.312049	-0.430733	0.655461
H	4.875771	1.798774	-1.348345
H	5.178027	2.689291	2.550997
H	5.501844	0.962872	2.454736
H	6.213692	3.054021	0.346802
H	6.777167	0.049141	0.277075
H	7.261450	1.234181	-0.934428
H	3.606101	2.544318	0.630177
H	3.151871	1.392706	1.888972
H	6.088133	-0.186058	-2.643444
H	4.300527	-1.719680	-3.155570
H	2.080702	-1.010381	-2.342408
H	2.511060	1.003218	-1.346176
H	4.684991	-2.618713	-0.377485
H	4.410402	-5.119182	-0.072131
H	4.047792	-4.286007	1.434474
H	2.728230	-4.898420	0.431577
H	1.171143	-3.973886	-0.465180
H	0.543313	-2.329701	-0.532685
H	0.979912	-3.157595	-2.024049
H	7.722174	3.081431	2.335211
H	8.021616	1.346519	2.147679
H	8.579007	2.480104	0.906076
H	-2.029878	1.761016	-2.229530
H	-3.001071	-0.728197	-1.205505
H	-3.362881	-1.998063	0.550778
H	-3.393246	0.415405	2.424435
H	-3.255193	-1.291782	2.815294

H	-5.417561	0.327978	-0.586655
H	-7.679540	0.085766	1.210631
H	-7.816290	-1.116581	-0.055995
H	-9.488624	1.676010	-1.340111
H	-1.895898	2.912312	0.393854
H	-3.491628	2.583833	-0.333764
H	-3.142658	2.058569	1.315655

B3LYP Energy = -1950.53698071 a.u.

(3*R*,4*S*,5*R*,8*R*,10*S*,19*R*,24*R*)-**1**, Conf D

C	5.608023	-2.137000	-1.054394
C	6.837181	-1.676496	-0.249709
C	6.722647	-0.180000	0.091631
C	5.391764	0.147943	0.794272
C	4.194725	-0.256411	-0.093594
C	4.274234	-1.767455	-0.383416
C	5.300840	1.585490	1.235939
C	4.160052	2.282378	1.255117
C	2.803377	1.742316	0.854486
C	2.879409	0.187069	0.583224
C	2.181014	2.602054	-0.254850
C	1.052431	3.283179	0.018873
C	0.274836	4.208742	-0.876437
C	2.904609	2.663293	-1.579306
C	1.632127	-0.254683	-0.165491
C	8.147362	-1.995060	-0.980506
C	0.374075	-0.499829	0.569052
O	1.613970	-0.416228	-1.387821
C	0.112819	-0.408183	2.031112
N	-1.263790	-0.575254	2.193973
C	-1.908189	-1.061333	0.983143
C	-0.798486	-0.829248	-0.047002
O	0.892166	-0.213079	2.951939
N	-3.111988	-0.236659	0.720117
C	-3.594264	-0.102458	-0.672122
C	-2.945166	-1.069897	-1.676406
S	-1.111908	-0.909717	-1.745060
C	-5.120687	-0.275286	-0.768084
N	-5.755837	-0.638082	0.358953
C	-7.183161	-0.945049	0.414491
C	-8.108129	0.282415	0.298656
O	-7.957213	1.025695	-0.805951
O	-5.704481	-0.076254	-1.846415
O	-8.939490	0.534838	1.140381
C	-2.270068	-2.551491	1.141086
H	4.293271	0.271384	-1.050305
H	5.353655	-0.478721	1.703619
H	5.656608	-3.221874	-1.215704
H	5.643921	-1.674752	-2.052489
H	6.836982	-2.230041	0.703144
H	6.803542	0.415215	-0.830455
H	7.562731	0.121162	0.732531
H	4.171373	-2.321469	0.562456
H	3.447117	-2.075610	-1.029057
H	6.221317	2.060370	1.575933
H	4.164126	3.319516	1.588037
H	2.149659	1.855664	1.727850
H	2.835128	-0.272396	1.578251
H	0.630355	3.166033	1.018762
H	0.161809	5.195848	-0.409767
H	0.735877	4.351826	-1.855916
H	-0.740857	3.825923	-1.044629
H	2.806932	1.714115	-2.118733
H	2.520570	3.454182	-2.226436
H	3.974396	2.843954	-1.426807
H	8.236164	-3.067645	-1.188781
H	8.199468	-1.464307	-1.939419
H	9.018866	-1.696009	-0.386502
H	-1.594448	-0.874541	3.103361
H	-2.914316	0.685021	1.099762
H	-3.396208	0.913535	-1.036961
H	-3.232487	-2.106085	-1.486934
H	-3.294890	-0.812569	-2.677593
H	-5.170421	-0.717508	1.184638
H	-7.395493	-1.425018	1.368776
H	-7.429675	-1.642480	-0.395960
H	-7.240843	0.661684	-1.382798
H	-1.368403	-3.133235	1.350025
H	-2.966808	-2.658086	1.978845
H	-2.747058	-2.967435	0.253240

B3LYP Energy = -1950.53661163 a.u.

(3*R*,4*S*,5*R*,8*R*,10*S*,19*R*,24*R*)-**1**, Conf E

C	5.364328	-2.408579	-1.166145
C	6.624473	-2.112930	-0.332657
C	6.647122	-0.635424	0.098199
C	5.346631	-0.224732	0.813870
C	4.125397	-0.462661	-0.101217
C	4.065302	-1.955137	-0.478998
C	5.388451	1.187864	1.336492
C	4.318697	1.987896	1.390091
C	2.920823	1.601954	0.953988
C	2.850893	0.064825	0.592784
C	2.394288	2.581261	-0.104802
C	1.337678	3.354981	0.206654
C	0.663612	4.405033	-0.633687
C	3.129143	2.643551	-1.422794
C	1.574748	-0.209933	-0.188233
C	7.905060	-2.507248	-1.079000
C	0.288961	-0.353193	0.522588
O	1.559467	-0.303633	-1.417794
C	0.026365	-0.325561	1.986437
N	-1.360473	-0.326059	2.135870
C	-2.053072	-0.634276	0.892582
C	-0.910390	-0.489354	-0.117105
O	0.815502	-0.292996	2.920372
N	-3.126672	0.365514	0.685081
C	-3.565473	0.668558	-0.696836
C	-3.054392	-0.316542	-1.759681
S	-1.216640	-0.424696	-1.815478

C	-5.103924	0.737438	-0.826374
N	-5.796870	0.430483	0.298961
C	-7.237337	0.380623	0.326575
C	-7.862422	-1.009088	0.312579
O	-6.975642	-2.007363	0.092213
O	-5.621155	1.059560	-1.893528
O	-9.046417	-1.201430	0.479405
C	-2.619263	-2.066825	0.948171
H	4.284412	0.106713	-1.025584
H	5.241326	-0.894979	1.685989
H	5.312868	-3.481962	-1.391143
H	5.451747	-1.894091	-2.135171
H	6.566377	-2.720517	0.584894
H	6.792316	0.003742	-0.785973
H	7.506083	-0.453805	0.758908
H	3.901099	-2.551991	0.431455
H	3.218947	-2.144846	-1.144888
H	6.346731	1.552984	1.706499
H	4.417845	2.999897	1.780901
H	2.273241	1.726927	1.830365
H	2.751499	-0.443746	1.559386
H	0.898721	3.222299	1.197283
H	0.659473	5.373018	-0.115664
H	1.136257	4.548222	-1.607653
H	-0.388001	4.144391	-0.814412
H	2.940512	1.739717	-2.013397
H	2.830693	3.503477	-2.025595
H	4.210605	2.706987	-1.259092
H	7.895578	-3.568903	-1.352120
H	8.013306	-1.926660	-2.003800
H	8.796120	-2.327065	-0.466272
H	-1.737205	-0.640997	3.021517
H	-2.809417	1.220386	1.133049
H	-3.206764	1.664225	-0.988354
H	-3.496188	-1.307846	-1.641215
H	-3.351779	0.055893	-2.741234
H	-5.236406	0.139564	1.092451
H	-7.608148	0.904132	-0.560864
H	-7.634550	0.898682	1.205112
H	-7.486270	-2.835482	0.079705
H	-1.808887	-2.780091	1.121237
H	-3.336945	-2.129735	1.772402
H	-3.139071	-2.351076	0.033314

B3LYP Energy = -1950.53628409 a.u.

(3*R*,4*S*,5*R*,8*R*,10*S*,19*R*,24*R*)-**1**, Conf F

C	5.654185	-2.198008	-0.705980
C	6.834942	-1.654477	0.118896
C	6.696209	-0.133851	0.312642
C	5.328484	0.245812	0.909682
C	4.181724	-0.251616	0.002287
C	4.284203	-1.781831	-0.144311
C	5.207291	1.717669	1.209563
C	4.062925	2.400972	1.105144
C	2.732681	1.810923	0.686234
C	2.831650	0.238413	0.570248
C	2.169379	2.553891	-0.533348
C	1.026914	3.250870	-0.388206
C	0.297257	4.078151	-1.411042
C	2.961229	2.487352	-1.817647
C	1.622020	-0.286977	-0.187536
C	8.185310	-2.029463	-0.503841
C	0.335242	-0.472218	0.512087
O	1.661695	-0.561237	-1.389237
C	0.012711	-0.242390	1.946232
N	-1.368355	-0.396045	2.066524
C	-1.964858	-0.996872	0.881947
C	-0.811852	-0.863510	-0.118366
O	0.753161	0.042893	2.876987
N	-3.152012	-0.204465	0.491264
C	-3.599432	-0.236413	-0.920573
C	-2.891683	-1.279531	-1.801054
S	-1.059017	-1.101289	-1.811088
C	-5.123093	-0.458742	-1.054313
N	-5.779782	-0.698366	0.108974
C	-7.215160	-0.823474	0.170413
C	-7.971908	0.388153	0.701878
O	-7.192651	1.476337	0.894573
O	-5.658502	-0.423109	-2.159741
O	-9.161518	0.374991	0.928914
C	-2.335851	-2.465453	1.170069
H	4.327875	0.186730	-0.992886
H	5.244675	-0.292947	1.870839
H	5.716697	-3.292477	-0.765810
H	5.742662	-1.826652	-1.738344
H	6.783539	-2.117590	1.117596
H	6.824192	0.373438	-0.655741
H	7.499793	0.233096	0.965995
H	4.131242	-2.249976	0.840388
H	3.494442	-2.153487	-0.803092
H	6.106354	2.231545	1.550314
H	4.043758	3.464678	1.339244
H	2.031563	2.001291	1.508016
H	2.744821	-0.123025	1.602082
H	0.551820	3.228444	0.594235
H	0.811290	4.127249	-2.373322
H	-0.706750	3.672914	-1.595958
H	0.157098	5.105734	-1.050993
H	2.873796	1.494655	-2.274126
H	2.625493	3.222449	-2.551784
H	4.024590	2.665925	-1.624523
H	8.289012	-3.116258	-0.603558
H	8.291364	-1.592287	-1.504777
H	9.020776	-1.668249	0.107261
H	-1.739430	-0.602984	2.985763
H	-2.958484	0.754958	0.763347
H	-3.417273	0.743231	-1.380256
H	-3.172567	-2.299397	-1.531151
H	-3.203658	-1.122041	-2.834700
H	-5.216254	-0.623741	0.949016
H	-7.511775	-1.678122	0.786801

H	-7.580541	-1.007276	-0.845131
H	-7.775538	2.188096	1.211103
H	-1.445927	-3.019637	1.480042
H	-3.073385	-2.489249	1.978750
H	-2.770553	-2.969569	0.306843

B3LYP Energy = -1950.53612337 a.u.

(3*R*,4*S*,5*R*,8*R*,10*S*,19*R*,24*R*)-**1**, Conf G

C	5.514329	-2.179828	-1.203460
C	6.706324	-1.943584	-0.258012
C	6.656761	-0.517478	0.319262
C	5.299480	-0.212448	0.980030
C	4.148995	-0.382750	-0.036012
C	4.159357	-1.829727	-0.565485
C	5.262506	1.138242	1.647058
C	4.167034	1.902198	1.709264
C	2.814411	1.531584	1.138102
C	2.816454	0.038543	0.620725
C	2.331927	2.601159	0.148133
C	1.226128	3.305471	0.453252
C	0.576657	4.416143	-0.326190
C	3.160823	2.824456	-1.094514
C	1.602647	-0.188221	-0.267696
C	8.045295	-2.232093	-0.948029
C	0.282564	-0.453722	0.337244
O	1.667587	-0.146638	-1.498531
C	-0.073853	-0.583882	1.776007
N	-1.465073	-0.661102	1.829253
C	-2.060334	-0.877307	0.518149
C	-0.865711	-0.577115	-0.392480
O	0.651737	-0.607872	2.760534
N	-3.171379	0.083620	0.335329
C	-3.552836	0.483566	-1.038137
C	-2.909627	-0.355889	-2.153352
S	-1.068952	-0.349186	-2.092562
C	-5.082822	0.463385	-1.254946
N	-5.815943	-0.000875	-0.217960
C	-7.261900	-0.052791	-0.288851
C	-7.921957	-0.609930	0.954068
O	-7.042679	-0.953018	1.929753
O	-5.567819	0.850805	-2.317471
O	-9.118637	-0.737815	1.074247
C	-2.548229	-2.334753	0.397325
H	4.349204	0.282561	-0.885045
H	5.156229	-0.972579	1.769152
H	5.509912	-3.225052	-1.539538
H	5.650895	-1.565732	-2.106456
H	6.603149	-2.642698	0.587506
H	6.842417	0.211561	-0.484011
H	7.463678	-0.385036	1.053241
H	3.954230	-2.519545	0.267642
H	3.364658	-1.970965	-1.303258
H	6.182337	1.485360	2.118177
H	4.208792	2.869528	2.208510
H	2.104299	1.550237	1.973787
H	2.672009	-0.568989	1.522419
H	0.719738	3.056799	1.387768
H	-0.446629	4.142221	-0.616491
H	0.494057	5.323457	0.286443
H	1.115972	4.676589	-1.239391
H	3.048341	1.985371	-1.790803
H	2.879658	3.736594	-1.624567
H	4.223967	2.897612	-0.840090
H	8.086478	-3.261777	-1.321973
H	8.197154	-1.561343	-1.803061
H	8.887522	-2.091456	-0.260439
H	-1.883618	-1.072663	2.654357
H	-2.925570	0.909700	0.872975
H	-3.252680	1.524785	-1.213551
H	-3.288282	-1.379944	-2.162528
H	-3.169346	0.093899	-3.112972
H	-5.317235	-0.278713	0.619887
H	-7.589333	-0.664862	-1.137646
H	-7.683076	0.945058	-0.463336
H	-7.562644	-1.290126	2.679612
H	-1.715830	-3.021192	0.574030
H	-3.325052	-2.508446	1.148746
H	-2.973436	-2.557806	-0.581438

B3LYP Energy = -1950.53552289 a.u.

(3*R*,4*S*,5*R*,8*R*,10*S*,19*R*,24*S*)-**1**, Conf A

C	-5.941307	-1.781343	0.849635
C	-7.002687	-1.261165	-0.136381
C	-6.672614	0.179943	-0.564122
C	-5.238421	0.302220	-1.111485
C	-4.209615	-0.155012	-0.053684
C	-4.503083	-1.618579	0.329320
C	-4.928052	1.679974	-1.636577
C	-3.717457	2.242610	-1.564834
C	-2.490829	1.590290	-0.962369
C	-2.781960	0.082547	-0.591752
C	-1.905113	2.470201	0.151521
C	-0.690928	3.019897	-0.036246
C	0.073267	3.939753	0.876386
C	-2.756639	2.701078	1.377521
C	-1.684018	-0.428865	0.329753
C	-8.419550	-1.373610	0.439849
C	-0.363606	-0.793293	-0.238595
O	-1.835249	-0.535366	1.545926
C	-0.001829	-0.979296	-1.678727
N	1.373330	-1.169501	-1.714187
C	1.919414	-1.444134	-0.395254
C	0.770345	-0.956837	0.498779
O	-0.723737	-0.954374	-2.666655
N	3.177320	-0.707732	-0.210454
C	3.681309	-0.739014	1.171497
C	2.755117	-0.082599	2.198629
S	1.004609	-0.706627	2.203428
C	5.091014	-0.120807	1.264921

N	5.793042	-0.102423	0.107179
C	7.154938	0.378301	0.061815
C	7.696933	0.239835	-1.343133
O	8.963131	0.695079	-1.427509
O	5.540863	0.286859	2.334552
O	7.085727	-0.220381	-2.284549
C	2.146482	-2.960247	-0.204624
H	-4.356998	0.458951	0.843602
H	-5.166271	-0.396642	-1.964345
H	-6.136915	-2.836477	1.081743
H	-6.040237	-1.232732	1.798589
H	-6.953679	-1.890299	-1.039786
H	-6.792626	0.855595	0.296463
H	-7.389199	0.515494	-1.326547
H	-4.355239	-2.258830	-0.553926
H	-3.800930	-1.959478	1.094716
H	-5.741072	2.223775	-2.118095
H	-3.559657	3.243113	-1.965810
H	-1.731123	1.560523	-1.753609
H	-2.678030	-0.463179	-1.537531
H	-0.176692	2.784385	-0.969887
H	-0.471167	4.194436	1.788080
H	1.026702	3.486316	1.179848
H	0.325200	4.876167	0.361607
H	-2.806126	1.794431	1.991603
H	-2.371552	3.506637	2.005780
H	-3.782306	2.958814	1.091504
H	-8.658156	-2.409009	0.709708
H	-8.524046	-0.761479	1.344592
H	-9.172129	-1.033505	-0.281206
H	1.783753	-1.587131	-2.540287
H	3.011329	0.256877	-0.499992
H	3.818116	-1.791270	1.446237
H	3.129873	-0.247928	3.209843
H	2.709324	0.998566	2.033993
H	5.374087	-0.475515	-0.738755
H	7.800870	-0.179282	0.751931
H	7.220101	1.429089	0.370109
H	9.258839	0.579717	-2.346877
H	2.391810	-3.217322	0.828058
H	1.236647	-3.501702	-0.473900
H	2.965352	-3.283105	-0.855245

B3LYP Energy = -1950.53674297 a.u.

(3*R*,4*S*,5*R*,8*R*,10*S*,19*R*,24*S*)-**1**, Conf B

C	5.945730	-1.720328	-0.655999
C	7.004019	-0.969394	0.172323
C	6.599555	0.505418	0.345726
C	5.178123	0.646537	0.921721
C	4.148190	-0.050986	0.005975
C	4.516737	-1.541996	-0.115994
C	4.800484	2.077633	1.203258
C	3.557642	2.553364	1.075030
C	2.356005	1.739458	0.641871
C	2.724633	0.206489	0.545539
C	1.697428	2.368933	-0.594436
C	0.465634	2.896831	-0.466889
C	-0.365319	3.601060	-1.504561
C	2.502605	2.403490	-1.871922
C	1.633462	-0.524788	-0.222267
C	8.405319	-1.107789	-0.435516
C	0.355012	-0.851028	0.454539
O	1.754461	-0.846135	-1.403691
C	0.047335	-0.797542	1.917519
N	-1.311159	-1.062247	2.040794
C	-1.872087	-1.604560	0.814320
C	-0.786013	-1.211422	-0.197176
O	0.792796	-0.553682	2.856790
N	-3.186405	-1.001650	0.551362
C	-3.720937	-1.319108	-0.783811
C	-2.874317	-0.791264	-1.944246
S	-1.083259	-1.285545	-1.908006
C	-5.174420	-0.823235	-0.927356
N	-5.857687	-0.720350	0.245752
C	-7.179197	-0.150628	0.305779
C	-7.170387	1.275666	0.839861
O	-8.424655	1.777227	0.887668
O	-5.665088	-0.597759	-2.029614
O	-6.189770	1.892122	1.192879
C	-1.989858	-3.142783	0.903633
H	4.233185	0.394893	-0.992894
H	5.175415	0.110046	1.887653
H	6.198243	-2.787999	-0.698046
H	5.981975	-1.353534	-1.693038
H	7.023567	-1.423040	1.176461
H	6.650694	1.017855	-0.626977
H	7.317484	1.013565	1.004364
H	4.433871	-2.016371	0.874092
H	3.813443	-2.054137	-0.777973
H	5.592151	2.741685	1.550992
H	3.351864	3.599868	1.296538
H	1.616820	1.817306	1.449193
H	2.680079	-0.157454	1.579444
H	-0.010407	2.820701	0.512497
H	-0.626717	4.613651	-1.170301
H	0.132171	3.685659	-2.473072
H	-1.315175	3.074399	-1.667289
H	2.602444	1.397655	-2.295673
H	2.046903	3.040705	-2.632094
H	3.513971	2.779307	-1.680427
H	8.699484	-2.160389	-0.521090
H	8.440716	-0.669839	-1.441034
H	9.158624	-0.598122	0.176631
H	-1.664022	-1.355519	2.943366
H	-3.088807	0.009620	0.655678
H	-3.782701	-2.410599	-0.861129
H	-3.260788	-1.158656	-2.896050
H	-2.906527	0.302175	-1.972199
H	-5.310361	-0.823197	1.092621
H	-7.844248	-0.751834	0.936292
H	-7.599202	-0.145875	-0.704381

H	-8.367568	2.684766	1.232720
H	-2.246897	-3.596868	-0.055755
H	-1.033791	-3.563416	1.223639
H	-2.761213	-3.400908	1.636186

B3LYP Energy = -1950.53602597 a.u.

(3*R*,4*S*,5*R*,8*R*,10*S*,19*R*,24*S*)-**1**, Conf C

C	-5.693750	-1.493284	1.551237
C	-6.736901	-1.640281	0.428110
C	-6.524894	-0.555677	-0.642854
C	-5.076339	-0.548186	-1.165454
C	-4.077778	-0.311448	-0.010529
C	-4.246533	-1.432767	1.032120
C	-4.866349	0.418101	-2.302107
C	-3.751108	1.136514	-2.469183
C	-2.551588	1.090192	-1.549336
C	-2.659863	-0.178543	-0.606710
C	-2.316998	2.436797	-0.843806
C	-3.348723	3.193681	-0.431342
C	-3.315970	4.516616	0.282174
C	-0.867765	2.842449	-0.703752
C	-1.550909	-0.178640	0.430025
C	-8.169237	-1.628401	0.976040
C	-0.220679	-0.740528	0.078510
O	-1.696641	0.284521	1.560105
C	0.148224	-1.562516	-1.118312
N	1.525498	-1.734191	-1.064385
C	2.068623	-1.372508	0.234243
C	0.914507	-0.537232	0.805247
O	-0.568614	-2.000916	-2.007529
N	3.323584	-0.628533	0.062965
C	3.824558	-0.022018	1.305752
C	2.895608	1.030264	1.916316
S	1.143569	0.474972	2.201090
C	5.233565	0.572273	1.105577
N	5.939505	0.048577	0.075648
C	7.302570	0.451645	-0.184104
C	7.851734	-0.335065	-1.353141
O	9.119464	0.026375	-1.635430
O	5.679282	1.432229	1.863385
O	7.244026	-1.185671	-1.968607
C	2.299124	-2.632405	1.098812
H	-4.332657	0.638151	0.479362
H	-4.881818	-1.561863	-1.560441
H	-5.799103	-2.320980	2.265005
H	-5.907512	-0.571716	2.113780
H	-6.567726	-2.615250	-0.056893
H	-6.766498	0.431769	-0.221005
H	-7.219843	-0.716867	-1.478647
H	-3.977196	-2.397693	0.575605
H	-3.567450	-1.273823	1.873778
H	-5.672203	0.506853	-3.031000
H	-3.670611	1.823069	-3.310342
H	-1.672150	0.915562	-2.185521
H	-2.475444	-1.034667	-1.265601
H	-4.350921	2.817259	-0.629162
H	-2.306904	4.897204	0.454624
H	-3.870058	5.276235	-0.284919
H	-3.809905	4.435661	1.259349
H	-0.383053	2.866849	-1.689338
H	-0.746418	3.826640	-0.248131
H	-0.306307	2.130159	-0.089034
H	-8.322478	-2.430580	1.707376
H	-8.389922	-0.676560	1.475588
H	-8.904452	-1.761622	0.173713
H	1.940749	-2.481178	-1.607495
H	3.157169	0.095054	-0.637417
H	3.963028	-0.831178	2.032130
H	3.265590	1.345921	2.892895
H	2.850951	1.916600	1.275699
H	5.521622	-0.675073	-0.500681
H	7.943169	0.287508	0.691641
H	7.368907	1.523127	-0.410119
H	9.419452	-0.509398	-2.389740
H	2.541234	-2.387375	2.135092
H	1.391588	-3.240567	1.106156
H	3.121049	-3.214271	0.670085

B3LYP Energy = -1950.53389160 a.u.

(3*R*,4*S*,5*R*,8*R*,10*S*,19*R*,24*S*)-**1**, Conf D

C	-5.935087	-1.729129	0.682913
C	-6.996677	-1.001699	-0.161968
C	-6.603185	0.472911	-0.360847
C	-5.181262	0.614587	-0.935565
C	-4.148308	-0.059106	-0.005569
C	-4.506128	-1.550276	0.143074
C	-4.812972	2.043255	-1.241020
C	-3.573698	2.529946	-1.119162
C	-2.367102	1.731598	-0.670622
C	-2.725844	0.198192	-0.548165
C	-1.713897	2.386250	0.555410
C	-0.484212	2.917253	0.420923
C	0.341742	3.642472	1.448183
C	-2.521032	2.438702	1.830916
C	-1.631111	-0.512652	0.233504
C	-8.398664	-1.139972	0.444279
C	-0.353043	-0.851317	-0.438318
O	-1.748258	-0.806921	1.422224
C	-0.048925	-0.833631	-1.902559
N	1.310812	-1.098407	-2.022938
C	1.874617	-1.609666	-0.784079
C	0.790315	-1.192853	0.219655
O	-0.796214	-0.614425	-2.846166
N	3.188306	-0.996438	-0.539748
C	3.726839	-1.276085	0.802699
C	2.880871	-0.720451	1.950232
S	1.091514	-1.221732	1.931307
C	5.180202	-0.774660	0.929487
N	5.859423	-0.697713	-0.246952
C	7.215159	-0.208722	-0.320265

C	7.385637	1.213002	-0.842227
O	6.217879	1.874303	-1.015205
O	5.673186	-0.520916	2.024499
O	8.466949	1.703956	-1.078579
C	1.996203	-3.148950	-0.834937
H	-4.237788	0.404321	0.984825
H	-5.172115	0.061669	-1.892138
H	-6.179683	-2.797674	0.744147
H	-5.976733	-1.343488	1.712868
H	-7.009557	-1.473592	-1.157736
H	-6.661192	1.002194	0.602339
H	-7.323042	0.963572	-1.030504
H	-4.417248	-2.042239	-0.837825
H	-3.800750	-2.044963	0.816113
H	-5.608630	2.695819	-1.601093
H	-3.374886	3.573909	-1.358288
H	-1.627396	1.800377	-1.478419
H	-2.678340	-0.183447	-1.575459
H	-0.006615	2.826490	-0.556577
H	-0.158121	3.740844	2.414092
H	1.293220	3.122277	1.622631
H	0.599102	4.650889	1.098300
H	-2.614573	1.439804	2.272034
H	-2.070900	3.092431	2.580286
H	-3.534560	2.804139	1.631394
H	-8.684479	-2.193111	0.548968
H	-8.440719	-0.683298	1.441159
H	-9.154208	-0.648249	-0.179632
H	1.657031	-1.422961	-2.917498
H	3.083217	0.011290	-0.666800
H	3.791791	-2.364974	0.909034
H	3.271097	-1.061008	2.910437
H	2.909534	0.373520	1.949745
H	5.323835	-0.868873	-1.089646
H	7.834039	-0.855600	-0.950034
H	7.636501	-0.231179	0.689611
H	6.443818	2.766709	-1.330209
H	2.257238	-3.578020	0.134780
H	1.040270	-3.579988	-1.141214
H	2.765865	-3.423928	-1.563142

B3LYP Energy = -1950.53378999 a.u.

(3*R*,4*S*,5*R*,8*R*,10*S*,19*R*,24*S*)-**1**, Conf E

C	5.824769	-1.872019	-1.233290
C	6.994913	-1.423831	-0.338956
C	6.760176	0.010158	0.168396
C	5.390834	0.158035	0.857467
C	4.249587	-0.224588	-0.110845
C	4.450243	-1.681717	-0.569907
C	5.182504	1.523439	1.459324
C	3.992342	2.128842	1.525176
C	2.690752	1.542680	1.019682
C	2.889434	0.040990	0.570416
C	2.037045	2.483224	-0.003475
C	0.867775	3.067608	0.318210
C	0.055515	4.044917	-0.486680
C	2.778087	2.730010	-1.296356
C	1.692023	-0.396132	-0.259332
C	8.344134	-1.561639	-1.054751
C	0.414925	-0.735458	0.416543
O	1.723035	-0.464885	-1.486904
C	0.173853	-0.953693	1.876563
N	-1.201684	-1.100832	2.028660
C	-1.863983	-1.325570	0.754718
C	-0.782591	-0.843788	-0.222796
O	0.977512	-0.982844	2.797353
N	-3.109527	-0.541353	0.692858
C	-3.725080	-0.533622	-0.643698
C	-2.875075	0.130925	-1.730173
S	-1.148895	-0.536449	-1.895531
C	-5.123942	0.097917	-0.600346
N	-5.715398	0.151166	0.608966
C	-7.024540	0.763546	0.831320
C	-8.214152	-0.060969	0.301781
O	-8.195534	-0.335357	-1.010459
O	-5.678441	0.488506	-1.639257
O	-9.120464	-0.409730	1.022598
C	-2.157015	-2.827511	0.547483
H	4.333146	0.417279	-0.996683
H	5.376313	-0.571227	1.687504
H	5.958733	-2.923430	-1.519265
H	5.851015	-1.291801	-2.168139
H	7.011471	-2.084345	0.543035
H	6.820532	0.713544	-0.676063
H	7.558279	0.293243	0.868493
H	4.364396	-2.349449	0.301103
H	3.666005	-1.969789	-1.274959
H	6.057116	2.020103	1.879719
H	3.910370	3.117224	1.975592
H	2.007005	1.507955	1.877209
H	2.855793	-0.537483	1.501867
H	0.436994	2.816813	1.289361
H	-0.114964	4.969139	0.080622
H	0.522156	4.315399	-1.435951
H	-0.937071	3.633179	-0.715584
H	2.730077	1.848166	-1.945554
H	2.372045	3.576028	-1.854231
H	3.836358	2.933573	-1.100523
H	8.517857	-2.593948	-1.380029
H	8.383418	-0.921483	-1.945153
H	9.174661	-1.272781	-0.400051
H	-1.541905	-1.550924	2.869822
H	-2.875034	0.413076	0.970450
H	-3.902919	-1.576557	-0.929944
H	-3.336804	-0.001168	-2.709840
H	-2.789081	1.206158	-1.543026
H	-5.201055	-0.249867	1.385397
H	-7.040589	1.749248	0.350367
H	-7.165907	0.892003	1.903494
H	-7.384963	0.036322	-1.435852
H	-2.490863	-3.052605	-0.467693

H	-1.247014	-3.403487	0.730194
H	-2.931055	-3.143857	1.253918

B3LYP Energy = -1950.53368950 a.u.

(3*R*,4*S*,5*R*,8*R*,10*S*,19*R*,24*S*)-**1**, Conf F

C	5.949617	-1.562784	-1.091050
C	7.043590	-0.964111	-0.188582
C	6.652780	0.457473	0.253223
C	5.256966	0.492900	0.903021
C	4.186585	-0.040960	-0.074580
C	4.545455	-1.487059	-0.467981
C	4.895807	1.852672	1.442367
C	3.649618	2.336690	1.454099
C	2.427874	1.602586	0.941817
C	2.789729	0.111538	0.565536
C	1.714939	2.428776	-0.138631
C	0.483890	2.904739	0.125989
C	-0.395505	3.766308	-0.738350
C	2.468010	2.696812	-1.419920
C	1.662546	-0.482678	-0.264303
C	8.419618	-0.993145	-0.864872
C	0.410858	-0.917451	0.405591
O	1.726009	-0.603460	-1.486459
C	0.168861	-1.113926	1.868745
N	-1.189679	-1.382503	2.007920
C	-1.807935	-1.707382	0.733726
C	-0.760917	-1.156946	-0.244981
O	0.957751	-1.043677	2.799857
N	-3.120375	-1.046412	0.631435
C	-3.711511	-1.130361	-0.714297
C	-2.908022	-0.415234	-1.802914
S	-1.129034	-0.935233	-1.931272
C	-5.164011	-0.630536	-0.699896
N	-5.784351	-0.650342	0.495989
C	-7.212173	-0.378323	0.659244
C	-7.599011	1.106214	0.513263
O	-7.279937	1.680358	-0.655376
O	-5.724580	-0.263700	-1.743934
O	-8.181532	1.702896	1.389238
C	-1.954903	-3.235926	0.570872
H	4.229576	0.570466	-0.984499
H	5.292448	-0.201071	1.762203
H	6.196032	-2.605662	-1.329661
H	5.943303	-1.020132	-2.048623
H	7.100476	-1.584999	0.720082
H	6.666695	1.130849	-0.617300
H	7.399092	0.845921	0.959842
H	4.504343	-2.125311	0.428041
H	3.813260	-1.881271	-1.177696
H	5.703809	2.451625	1.862840
H	3.456367	3.328666	1.860464
H	1.726071	1.536080	1.782923
H	2.793117	-0.425807	1.521934
H	0.050914	2.651102	1.095438
H	-1.342400	3.257457	-0.964365
H	-0.661164	4.695321	-0.217430
H	0.065273	4.035926	-1.690788
H	1.984912	3.458562	-2.034711
H	3.486928	3.035680	-1.203164
H	2.548337	1.784159	-2.021616
H	8.705022	-2.014819	-1.141363
H	8.419942	-0.388624	-1.780690
H	9.198108	-0.595541	-0.203282
H	-1.500473	-1.834157	2.859500
H	-2.980479	-0.066929	0.885374
H	-3.786652	-2.192367	-0.975656
H	-3.341821	-0.605608	-2.785817
H	-2.918962	0.666649	-1.636553
H	-5.227719	-0.945087	1.290692
H	-7.509500	-0.696465	1.657409
H	-7.774315	-0.960641	-0.081642
H	-6.819764	1.037577	-1.248359
H	-2.251523	-3.522130	-0.440444
H	-0.997011	-3.716783	0.781711
H	-2.705063	-3.602760	1.278736

B3LYP Energy = -1950.53363462 a.u.

(3*R*,4*S*,5*R*,8*R*,10*S*,19*R*,24*S*)-**1**, Conf G

C	5.789241	-1.930176	-1.210101
C	6.951485	-1.550121	-0.274726
C	6.748540	-0.128599	0.279339
C	5.368156	0.037588	0.941999
C	4.240019	-0.271962	-0.066874
C	4.405666	-1.717973	-0.572858
C	5.187564	1.386045	1.589213
C	4.015429	2.026318	1.649573
C	2.708872	1.501584	1.092091
C	2.871250	0.013068	0.588324
C	2.107861	2.501790	0.093818
C	0.943028	3.099563	0.406998
C	0.176416	4.128322	-0.378279
C	2.892092	2.785513	-1.165628
C	1.685332	-0.352278	-0.292487
C	8.311565	-1.703380	-0.966606
C	0.380839	-0.682187	0.330969
O	1.753703	-0.370009	-1.520692
C	0.090207	-0.945023	1.774028
N	-1.291313	-1.063518	1.879949
C	-1.921896	-1.225443	0.579725
C	-0.799832	-0.737264	-0.347505
O	0.864785	-1.026552	2.717539
N	-3.148389	-0.416425	0.512252
C	-3.729035	-0.344432	-0.839249
C	-2.830149	0.329174	-1.878579
S	-1.111062	-0.363505	-2.016083
C	-5.112891	0.334828	-0.821636
N	-5.737423	0.335426	0.387747
C	-7.097496	0.793390	0.543500
C	-8.173679	-0.286761	0.561344
O	-7.704784	-1.527922	0.300951

O	-5.600284	0.807473	-1.844061
O	-9.339459	-0.051046	0.789030
C	-2.237767	-2.712908	0.308609
H	4.366710	0.397182	-0.927012
H	5.309993	-0.721219	1.743110
H	5.897248	-2.975225	-1.528822
H	5.856259	-1.320297	-2.123769
H	6.928653	-2.241818	0.582889
H	6.849935	0.602213	-0.537484
H	7.538442	0.104231	1.006825
H	4.277169	-2.412227	0.271694
H	3.630080	-1.955498	-1.305756
H	6.066563	1.838926	2.048323
H	3.953668	2.999632	2.134858
H	2.004559	1.454421	1.932136
H	2.792495	-0.599694	1.494755
H	0.477137	2.816128	1.352542
H	-0.003726	5.025374	0.228240
H	0.684087	4.438754	-1.293730
H	-0.811642	3.743860	-0.666083
H	2.846490	1.931736	-1.851580
H	2.518537	3.661157	-1.700020
H	3.947816	2.959721	-0.930731
H	8.461883	-2.728362	-1.325150
H	8.388373	-1.033719	-1.832557
H	9.135903	-1.462709	-0.285113
H	-1.669482	-1.530026	2.695346
H	-2.910122	0.521724	0.836891
H	-3.933005	-1.371673	-1.162279
H	-3.265836	0.236599	-2.874439
H	-2.728002	1.396577	-1.658637
H	-5.267192	-0.154210	1.139642
H	-7.330236	1.458085	-0.293883
H	-7.210425	1.371435	1.465768
H	-8.470740	-2.127573	0.317300
H	-2.547181	-2.891479	-0.723222
H	-1.345177	-3.314443	0.495286
H	-3.038959	-3.037502	0.980207

B3LYP Energy = -1950.53355434 a.u.

(3*R*,4*S*,5*R*,8*R*,10*S*,19*R*,24*S*)-**1**, Conf H

C	-5.752865	-1.714234	0.938899
C	-6.793174	-1.322140	-0.126375
C	-6.472044	0.068744	-0.702632
C	-5.022651	0.151783	-1.216496
C	-4.021499	-0.163978	-0.082256
C	-4.301096	-1.583641	0.446685
C	-4.713463	1.458926	-1.898281
C	-3.538171	2.088478	-1.798747
C	-2.361430	1.591399	-0.989671
C	-2.589858	0.069867	-0.610431
C	-2.028955	2.536561	0.178088
C	-3.003269	3.155247	0.867696
C	-2.876361	4.090373	2.038057
C	-0.554368	2.738619	0.440463
C	-1.499121	-0.425191	0.321370
C	-8.223733	-1.395539	0.421436
C	-0.211439	-0.902633	-0.247730
O	-1.623309	-0.436233	1.544918
C	0.103808	-1.225513	-1.675882
N	1.465334	-1.498298	-1.722904
C	2.026647	-1.699414	-0.397647
C	0.930327	-1.074232	0.476229
O	-0.637958	-1.241387	-2.648483
N	3.331149	-1.029655	-0.296512
C	3.863779	-0.981038	1.075468
C	3.004087	-0.183653	2.058679
S	1.220356	-0.698814	2.149652
C	5.307824	-0.438236	1.083507
N	5.994425	-0.637176	-0.075384
C	7.300854	-0.069032	-0.289677
C	7.257255	1.143964	-1.209230
O	8.496575	1.649559	-1.396304
O	5.788375	0.081501	2.086210
O	6.262820	1.602712	-1.726214
C	2.167133	-3.206792	-0.088690
H	-4.198118	0.538146	0.743935
H	-4.914446	-0.644323	-1.975256
H	-5.940578	-2.741359	1.278560
H	-5.887630	-1.068321	1.819766
H	-6.709969	-2.045355	-0.953606
H	-6.629032	0.834235	0.072348
H	-7.169236	0.303253	-1.518982
H	-4.115641	-2.313667	-0.356173
H	-3.619355	-1.825409	1.266133
H	-5.497690	1.889437	-2.521368
H	-3.386660	3.035646	-2.313781
H	-1.489266	1.605204	-1.658723
H	-2.470101	-0.474990	-1.553857
H	-4.028958	2.966398	0.555376
H	-1.842940	4.297171	2.324625
H	-3.364570	5.049792	1.821941
H	-3.385291	3.672878	2.916787
H	-0.064943	1.805181	0.739848
H	-0.049630	3.087350	-0.470652
H	-0.361411	3.469376	1.227723
H	-8.456370	-2.400321	0.793142
H	-8.361098	-0.693287	1.253435
H	-8.960640	-1.145277	-0.350832
H	1.826615	-2.007848	-2.519740
H	3.222179	-0.082124	-0.661811
H	3.942860	-2.013544	1.434243
H	3.390789	-0.285551	3.073709
H	3.019549	0.879888	1.802065
H	5.451125	-0.968107	-0.864761
H	7.990992	-0.804208	-0.719453
H	7.707417	0.235921	0.679157
H	8.416619	2.417658	-1.987584
H	2.429469	-3.395380	0.954571
H	1.218449	-3.710524	-0.287967
H	2.944122	-3.632387	-0.731647

B3LYP Energy = -1950.53318051 a.u.

(3*R*,4*S*,5*R*,8*R*,10*S*,19*R*,24*S*)-**1**, Conf I

C	4.679831	-2.020144	-1.185826
C	5.895051	-1.590911	-0.343280
C	5.761288	-0.116042	0.076986
C	4.417054	0.158941	0.776726
C	3.236906	-0.208422	-0.147077
C	3.333710	-1.701583	-0.512409
C	4.300566	1.566496	1.300304
C	3.155867	2.257032	1.326497
C	1.814831	1.744809	0.829527
C	1.909224	0.200382	0.541450
C	1.315171	2.698929	-0.269160
C	0.437515	3.665096	0.081903
C	-0.046058	4.838314	-0.732371
C	1.962851	2.621073	-1.630066
C	0.684697	-0.333094	-0.192200
C	7.216240	-1.856875	-1.075314
C	-0.407638	-0.977174	0.559605
O	0.596581	-0.304875	-1.423095
C	-0.578757	-1.080648	2.035708
N	-1.784518	-1.732851	2.238180
C	-2.307845	-2.334918	1.011815
C	-1.464458	-1.607456	-0.039214
O	0.150052	-0.673856	2.932838
N	-3.751756	-2.095841	0.875057
C	-4.333555	-1.831998	-0.450558
C	-3.525368	-2.384499	-1.631810
S	-1.830778	-1.658221	-1.723922
C	-4.649599	-0.333786	-0.685022
N	-4.490659	0.464944	0.397227
C	-4.619891	1.900455	0.317405
C	-3.309536	2.673207	0.175905
O	-2.245436	1.879601	-0.015325
O	-5.060581	0.045247	-1.779541
O	-3.265768	3.885004	0.233683
C	-1.976935	-3.849777	1.000055
H	3.345171	0.364357	-1.075250
H	4.373442	-0.517303	1.649738
H	4.742827	-3.093927	-1.405820
H	4.721224	-1.503106	-2.156380
H	5.890685	-2.195166	0.578287
H	5.849806	0.529550	-0.809806
H	6.589073	0.157092	0.745845
H	3.224188	-2.306970	0.400591
H	2.519292	-1.984510	-1.186194
H	5.205019	2.022151	1.703819
H	3.143485	3.269908	1.725558
H	1.111051	1.848192	1.665756
H	1.901479	-0.255104	1.537367
H	0.087585	3.664597	1.117369
H	-1.128674	4.965382	-0.627196
H	0.416643	5.765334	-0.366376
H	0.186369	4.751035	-1.795914
H	1.753807	1.659018	-2.106896
H	1.618426	3.413555	-2.296074
H	3.051414	2.715335	-1.535366
H	7.318504	-2.915774	-1.339875
H	7.273277	-1.274758	-2.003656
H	8.078031	-1.581657	-0.456177
H	-1.962133	-2.146459	3.144567
H	-4.263856	-2.832867	1.346279
H	-5.312259	-2.326928	-0.477600
H	-3.477773	-3.475681	-1.597937
H	-4.003380	-2.079251	-2.564519
H	-4.083678	0.025423	1.215223
H	-5.134042	2.294747	1.198823
H	-5.230253	2.139646	-0.558645
H	-1.431918	2.421742	-0.109635
H	-2.271543	-4.317738	0.058154
H	-0.906400	-4.010733	1.150694
H	-2.519670	-4.344760	1.814787

B3LYP Energy = -1950.53298244 a.u.

(3*R*,4*S*,5*R*,8*R*,10*S*,19*S*,24*R*)-**1**, Conf A

C	4.299999	3.242596	0.718767
C	5.450370	3.396131	-0.292599
C	6.000504	2.014736	-0.690185
C	4.888034	1.075574	-1.192200
C	3.801013	0.892753	-0.111330
C	3.207321	2.269200	0.244397
C	5.417809	-0.247216	-1.682019
C	4.753923	-1.400587	-1.554638
C	3.384329	-1.553110	-0.928231
C	2.759165	-0.137841	-0.601718
C	3.418785	-2.573792	0.217875
C	2.699681	-3.704443	0.087391
C	2.587794	-4.859718	1.044532
C	4.294510	-2.262476	1.408571
C	1.587654	-0.309138	0.356207
C	6.554805	4.318495	0.237930
C	0.235997	-0.619136	-0.167775
O	1.717696	-0.198777	1.574809
C	-0.155583	-0.948229	-1.573460
N	-1.543882	-0.998566	-1.591984
C	-2.101826	-1.049856	-0.250759
C	-0.905258	-0.557649	0.576622
O	0.556248	-1.121815	-2.553460
N	-3.289749	-0.190873	-0.168894
C	-3.783117	0.002701	1.203093
C	-2.797636	0.712723	2.134074
S	-1.102226	-0.045235	2.225609
C	-5.139226	0.737364	1.209911
N	-5.848861	0.650015	0.060088
C	-7.170811	1.222204	-0.053723
C	-7.731980	0.937127	-1.428748
O	-8.961330	1.470330	-1.576935
O	-5.545518	1.321254	2.213172

O	-7.163897	0.310015	-2.298004
C	-2.456193	-2.503455	0.136637
H	4.286006	0.497816	0.789823
H	4.414169	1.581303	-2.053004
H	3.858969	4.225589	0.930261
H	4.712039	2.877511	1.671687
H	5.033184	3.856691	-1.202780
H	6.500997	1.554863	0.175494
H	6.767069	2.129007	-1.469152
H	2.703250	2.684976	-0.641833
H	2.449778	2.165708	1.026700
H	6.386427	-0.240458	-2.182254
H	5.194215	-2.323108	-1.931323
H	2.731559	-1.982112	-1.696487
H	2.355386	0.215251	-1.558090
H	2.113550	-3.818732	-0.826044
H	2.877889	-5.799086	0.555719
H	3.204718	-4.741640	1.937811
H	1.549115	-4.989443	1.377034
H	3.857487	-1.457209	2.010255
H	4.432174	-3.127490	2.060400
H	5.285057	-1.926724	1.081912
H	6.159208	5.309926	0.487723
H	7.009475	3.903285	1.146266
H	7.352595	4.453062	-0.501924
H	-1.999914	-1.478365	-2.358266
H	-3.046658	0.709366	-0.584047
H	-4.001810	-0.988921	1.615859
H	-2.665604	1.754761	1.825959
H	-3.173954	0.715363	3.158148
H	-5.465312	0.135694	-0.726502
H	-7.154088	2.306814	0.109905
H	-7.852078	0.813075	0.703175
H	-9.271742	1.254839	-2.473162
H	-1.597429	-3.150424	-0.055924
H	-2.714230	-2.601633	1.193121
H	-3.304419	-2.837654	-0.469049

B3LYP Energy = -1950.53642090 a.u.

(3*R*,4*S*,5*R*,8*R*,10*S*,19*S*,24*R*)-**1**, Conf B

C	3.756034	3.343294	1.100876
C	4.891772	3.744437	0.141815
C	5.621835	2.492359	-0.375769
C	4.646888	1.476770	-1.000314
C	3.578084	1.046978	0.027665
C	2.805738	2.292299	0.502902
C	5.347104	0.288239	-1.606765
C	4.835327	-0.947049	-1.610968
C	3.490265	-1.336541	-1.035085
C	2.683561	-0.052270	-0.588389
C	3.645769	-2.450694	0.009053
C	3.077230	-3.644927	-0.241546
C	3.106842	-4.891262	0.600499
C	4.462886	-2.147163	1.242733
C	1.530917	-0.457901	0.320828
C	5.859897	4.740637	0.791686
C	0.219615	-0.831371	-0.260366
O	1.639844	-0.477156	1.546111
C	-0.118474	-1.063841	-1.698330
N	-1.499046	-1.210075	-1.756941
C	-2.080421	-1.412647	-0.440422
C	-0.935857	-0.921018	0.457839
O	0.624376	-1.104097	-2.669578
N	-3.321929	-0.636690	-0.313170
C	-3.854016	-0.600426	1.059275
C	-2.933938	0.080943	2.074560
S	-1.197349	-0.578603	2.141648
C	-5.249044	0.057677	1.093090
N	-5.940933	-0.017621	-0.076516
C	-7.204663	0.650107	-0.256889
C	-7.088313	1.863286	-1.168591
O	-8.288149	2.466557	-1.320511
O	-5.692427	0.557117	2.123142
O	-6.074166	2.246406	-1.708471
C	-2.351602	-2.913110	-0.189659
H	4.098750	0.626965	0.897094
H	4.124263	2.006018	-1.817744
H	3.188336	4.235311	1.396640
H	4.197934	2.938172	2.023738
H	4.431978	4.238698	-0.729349
H	6.164117	2.013643	0.453692
H	6.378621	2.782286	-1.117764
H	2.263528	2.728423	-0.350485
H	2.058181	2.014505	1.251594
H	6.312416	0.468241	-2.080335
H	5.395349	-1.762633	-2.067081
H	2.907161	-1.768737	-1.856050
H	2.250766	0.342604	-1.515283
H	2.515787	-3.743456	-1.172055
H	3.504911	-5.738341	0.026586
H	3.708907	-4.786400	1.505548
H	2.092241	-5.174659	0.911369
H	4.726716	-3.049169	1.798611
H	5.392911	-1.633882	0.974638
H	3.907965	-1.487577	1.919828
H	5.336829	5.645483	1.122473
H	6.346645	4.298000	1.669914
H	6.647581	5.045536	0.092821
H	-1.903151	-1.651616	-2.573506
H	-3.128127	0.312119	-0.637519
H	-4.019841	-1.636834	1.374362
H	-2.862718	1.152501	1.864277
H	-3.329967	-0.032903	3.084741
H	-5.425527	-0.354088	-0.881910
H	-7.564002	0.974847	0.724201
H	-7.960174	-0.025068	-0.675902
H	-8.162726	3.230263	-1.909560
H	-1.450150	-3.488370	-0.412414
H	-2.628037	-3.118899	0.846673
H	-3.162977	-3.244068	-0.845734

B3LYP Energy = -1950.53588657 a.u.

(3*R*,4*S*,5*R*,8*R*,10*S*,19*S*,24*R*)-**1**, Conf C

C	3.084263	-2.979485	-1.507753
C	4.228912	-3.458102	-0.597146
C	4.951114	-2.252573	0.029873
C	3.971846	-1.309618	0.753348
C	2.885942	-0.797148	-0.217082
C	2.126317	-2.000787	-0.807530
C	4.667831	-0.175771	1.460899
C	4.137817	1.043901	1.598209
C	2.775375	1.463383	1.088695
C	1.984105	0.218664	0.519576
C	2.892542	2.685926	0.167801
C	2.326524	3.841055	0.564837
C	2.327410	5.174508	-0.131559
C	3.676841	2.523170	-1.112502
C	0.808966	0.704768	-0.316993
C	5.200820	-4.381302	-1.342362
C	-0.460366	1.082535	0.334385
O	0.874090	0.802847	-1.544680
C	-0.782091	1.112714	1.790771
N	-2.125946	1.419924	1.876879
C	-2.698985	1.846932	0.602450
C	-1.588505	1.416125	-0.361129
O	-0.047846	0.904619	2.749780
N	-3.989354	1.190506	0.345036
C	-4.299978	0.696462	-1.002568
C	-3.640159	1.479620	-2.144902
S	-1.796038	1.409887	-2.076967
C	-4.041908	-0.821113	-1.166626
N	-3.733490	-1.482182	-0.029614
C	-3.549142	-2.913444	-0.011641
C	-3.732388	-3.446457	1.392146
O	-3.554172	-4.784128	1.433263
O	-4.166651	-1.359725	-2.265996
O	-3.994284	-2.777042	2.368434
C	-2.840941	3.389884	0.587941
H	3.390241	-0.283759	-1.045372
H	3.463097	-1.916967	1.523908
H	2.523924	-3.844975	-1.885169
H	3.517105	-2.483839	-2.389878
H	3.779282	-4.035548	0.226936
H	5.482745	-1.693693	-0.755194
H	5.716444	-2.602428	0.736527
H	1.593805	-2.523282	0.002593
H	1.371575	-1.659228	-1.521414
H	5.645184	-0.385841	1.895856
H	4.693875	1.820156	2.122703
H	2.199643	1.793542	1.960865
H	1.570294	-0.274487	1.407225
H	1.793678	3.831087	1.517332
H	2.888265	5.172886	-1.068729
H	1.302252	5.493754	-0.362836
H	2.756466	5.950394	0.516127
H	3.889839	3.478903	-1.595610
H	3.123743	1.902690	-1.826996
H	4.632733	2.024357	-0.917523
H	4.681972	-5.253477	-1.757026
H	5.682286	-3.853826	-2.175562
H	5.992732	-4.747374	-0.678345
H	-2.518517	1.701271	2.765386
H	-4.743703	1.771507	0.692276
H	-5.384568	0.798819	-1.133510
H	-3.896280	1.013562	-3.098364
H	-3.985539	2.516675	-2.151129
H	-3.716605	-0.962347	0.842493
H	-2.551767	-3.205264	-0.367476
H	-4.265799	-3.395836	-0.686100
H	-3.670646	-5.067690	2.356192
H	-1.885463	3.865618	0.822213
H	-3.182342	3.749711	-0.385828
H	-3.573896	3.693340	1.345546

B3LYP Energy = -1950.53486052 a.u.

(3*R*,4*S*,5*R*,8*R*,10*S*,19*S*,24*R*)-**1**, Conf D

C	3.759336	3.224813	0.931171
C	4.811040	3.664222	-0.104333
C	5.524456	2.436233	-0.698499
C	4.523102	1.408155	-1.258665
C	3.553449	0.938715	-0.152965
C	2.789339	2.159363	0.393718
C	5.192122	0.247585	-1.948007
C	4.743401	-1.010558	-1.883901
C	3.514014	-1.455691	-1.124941
C	2.656438	-0.190428	-0.707067
C	3.856650	-2.433238	0.009263
C	4.965408	-2.277285	0.752900
C	5.449313	-3.119065	1.900723
C	2.885258	-3.579968	0.167701
C	1.546384	-0.572851	0.259027
C	5.806180	4.669984	0.487429
C	0.185603	-0.869401	-0.253032
O	1.726793	-0.623789	1.474303
C	-0.227616	-1.115936	-1.669282
N	-1.616489	-1.153791	-1.670275
C	-2.157637	-1.275943	-0.326067
C	-0.941113	-0.860516	0.513636
O	0.473808	-1.247664	-2.662876
N	-3.322367	-0.392836	-0.171385
C	-3.790768	-0.272134	1.217882
C	-2.772930	0.349111	2.178166
S	-1.105419	-0.470529	2.200975
C	-5.122230	0.502043	1.293887
N	-5.848943	0.519618	0.151487
C	-7.149190	1.147830	0.099490
C	-7.735188	0.992365	-1.285880
O	-8.943569	1.583297	-1.376379
O	-5.496516	1.027942	2.340810
O	-7.201438	0.412607	-2.208165
C	-2.544308	-2.741069	-0.025840

H	4.145828	0.520571	0.672001
H	3.921248	1.939170	-2.018530
H	3.196461	4.100251	1.281012
H	4.277897	2.818750	1.812674
H	4.276622	4.162216	-0.929479
H	6.141295	1.957278	0.076828
H	6.213207	2.754267	-1.493443
H	2.173426	2.592729	-0.409663
H	2.108624	1.857492	1.194793
H	6.077928	0.468522	-2.543868
H	5.281816	-1.801838	-2.403008
H	2.884760	-2.007271	-1.835647
H	2.189464	0.154763	-1.636118
H	5.612189	-1.435597	0.509466
H	4.795599	-3.963754	2.128712
H	6.451943	-3.516949	1.694921
H	5.535833	-2.511123	2.810825
H	1.856282	-3.221772	0.286958
H	2.893566	-4.211226	-0.731574
H	3.110052	-4.215318	1.026088
H	5.291813	5.556100	0.877307
H	6.370431	4.221770	1.314947
H	6.529079	5.005995	-0.265112
H	-2.088673	-1.583452	-2.456283
H	-3.060117	0.524953	-0.533064
H	-4.033398	-1.281224	1.570456
H	-2.610296	1.403172	1.931694
H	-3.138964	0.301425	3.204827
H	-5.495468	0.049056	-0.675543
H	-7.088438	2.214849	0.346618
H	-7.838036	0.707252	0.831490
H	-9.271278	1.450605	-2.282471
H	-1.705009	-3.396754	-0.268974
H	-2.792620	-2.897825	1.025906
H	-3.407479	-3.016732	-0.639763

B3LYP Energy = -1950.53415388 a.u.

(3*R*,4*S*,5*R*,8*R*,10*S*,19*S*,24*R*)-**1**, Conf E

C	-2.822544	3.431795	-1.067230
C	-4.024020	3.864732	-0.208108
C	-4.886679	2.643703	0.156294
C	-4.054596	1.528955	0.817591
C	-2.911794	1.074947	-0.116257
C	-2.013470	2.284685	-0.437202
C	-4.896063	0.367221	1.278072
C	-4.470260	-0.899889	1.274258
C	-3.095942	-1.354983	0.831924
C	-2.164590	-0.112094	0.532038
C	-3.202820	-2.417536	-0.270714
C	-2.756983	-3.660132	-0.006591
C	-2.781765	-4.874425	-0.894423
C	-3.847520	-2.008442	-1.573711
C	-0.952703	-0.573204	-0.262390
C	-4.848467	4.960396	-0.895169
C	0.211204	-1.164184	0.430958
O	-0.895829	-0.483026	-1.490634
C	0.394991	-1.413047	1.888892
N	1.700314	-1.855911	2.041708
C	2.332548	-2.190273	0.766641
C	1.357224	-1.532561	-0.214269
O	-0.400061	-1.263273	2.807275
N	3.693410	-1.631767	0.689623
C	4.184822	-1.072261	-0.575651
C	3.532668	-1.649556	-1.839182
S	1.714054	-1.336742	-1.896191
C	4.123074	0.468717	-0.597923
N	3.753775	1.068042	0.545261
C	3.559728	2.511468	0.665647
C	4.863553	3.332589	0.677874
O	5.660141	3.177997	-0.388966
O	4.454750	1.090490	-1.620895
O	5.132245	4.092073	1.580583
C	2.335328	-3.726719	0.573100
H	-3.361883	0.735714	-1.057414
H	-3.588646	1.974381	1.715325
H	-2.167843	4.293835	-1.250014
H	-3.189597	3.108287	-2.053014
H	-3.628030	4.278946	0.733100
H	-5.367134	2.248978	-0.751818
H	-5.698005	2.948787	0.831402
H	-1.538446	2.636748	0.491673
H	-1.210604	1.991150	-1.119581
H	-5.895179	0.593643	1.650651
H	-5.129541	-1.694286	1.622033
H	-2.637297	-1.855485	1.692571
H	-1.806080	0.208160	1.517567
H	-2.321859	-3.832591	0.979466
H	-3.334424	-5.693674	-0.415971
H	-3.237277	-4.687698	-1.869120
H	-1.764390	-5.247834	-1.072742
H	-3.193420	-1.329155	-2.132426
H	-4.066695	-2.863232	-2.216400
H	-4.787594	-1.477192	-1.387561
H	-4.233560	5.839784	-1.119317
H	-5.269095	4.598560	-1.841935
H	-5.682756	5.287829	-0.263822
H	1.946378	-2.343515	2.893904
H	4.359047	-2.296939	1.066659
H	5.256060	-1.301703	-0.636489
H	3.928728	-1.137727	-2.718477
H	3.746616	-2.717439	-1.927251
H	3.550426	0.452847	1.326640
H	3.036906	2.710882	1.599766
H	2.937920	2.856782	-0.169868
H	5.272430	2.526854	-1.025587
H	1.324323	-4.127806	0.678362
H	2.720710	-4.005872	-0.410152
H	2.972944	-4.188201	1.337058

B3LYP Energy = -1950.53382392 a.u.

(3*R*,4*S*,5*R*,8*R*,10*S*,19*S*,24*R*)-**1**, Conf F

C	-3.723470	-3.355781	1.082320
C	-4.864811	-3.753600	0.128518
C	-5.604748	-2.500269	-0.371783
C	-4.639456	-1.474643	-0.994791
C	-3.564805	-1.048892	0.028731
C	-2.782845	-2.294726	0.486650
C	-5.349979	-0.284217	-1.585356
C	-4.844273	0.953536	-1.582558
C	-3.496666	1.344547	-1.013694
C	-2.680411	0.060238	-0.584636
C	-3.649124	2.448828	0.041198
C	-3.088039	3.647849	-0.203217
C	-3.117573	4.886703	0.649770
C	-4.456046	2.131023	1.278005
C	-1.522616	0.463305	0.318933
C	-5.823035	-4.760205	0.776998
C	-0.216686	0.845890	-0.269229
O	-1.621804	0.472749	1.545022
C	0.109782	1.090409	-1.707437
N	1.490566	1.240301	-1.776069
C	2.080673	1.434324	-0.461764
C	0.943854	0.933851	0.441186
O	-0.639614	1.136098	-2.673138
N	3.323327	0.656509	-0.350041
C	3.867708	0.610061	1.017835
C	2.955324	-0.075754	2.036966
S	1.218141	0.580423	2.120639
C	5.261047	-0.052797	1.034001
N	5.941013	0.023185	-0.142143
C	7.230725	-0.598323	-0.323744
C	7.234104	-1.916956	-1.088498
O	5.996695	-2.395362	-1.355371
O	5.712748	-0.560222	2.056140
O	8.250454	-2.482763	-1.424425
C	2.353809	2.932375	-0.201615
H	-4.080566	-0.639511	0.906109
H	-4.120744	-1.994244	-1.820893
H	-3.149301	-4.247710	1.365619
H	-4.160017	-2.961165	2.012221
H	-4.409634	-4.237693	-0.750770
H	-6.142477	-2.031571	0.466290
H	-6.366107	-2.786926	-1.110307
H	-2.245362	-2.720401	-0.375059
H	-2.030810	-2.020121	1.232079
H	-6.318120	-0.464735	-2.052855
H	-5.411783	1.770401	-2.026899
H	-2.922222	1.786766	-1.835469
H	-2.253313	-0.324591	-1.518397
H	-2.534324	3.757364	-1.137159
H	-3.524713	5.736453	0.086309
H	-3.711809	4.770835	1.558618
H	-2.102069	5.172939	0.954986
H	-3.892838	1.469829	1.946638
H	-4.721724	3.027192	1.842338
H	-5.384694	1.614165	1.012099
H	-5.293232	-5.665662	1.095131
H	-6.304477	-4.327883	1.663214
H	-6.615040	-5.062253	0.081827
H	1.882721	1.699811	-2.588706
H	3.121245	-0.290559	-0.674016
H	4.039289	1.644045	1.337671
H	2.883825	-1.146651	1.823389
H	3.359281	0.034110	3.044429
H	5.435021	0.405648	-0.932126
H	7.650193	-0.801932	0.666387
H	7.921608	0.073302	-0.842789
H	6.116377	-3.238953	-1.825142
H	1.450760	3.509346	-0.413225
H	2.637615	3.129993	0.834258
H	3.160355	3.268978	-0.860813

B3LYP Energy = -1950.53362959 a.u.

(3*R*,4*S*,5*R*,8*R*,10*S*,19*S*,24*R*)-**1**, Conf G

C	-3.632452	3.185856	-1.284648
C	-4.645961	3.753481	-0.274807
C	-5.383985	2.607863	0.440569
C	-4.404272	1.603834	1.076475
C	-3.463322	1.006874	0.008005
C	-2.678955	2.148985	-0.665769
C	-5.101563	0.531318	1.872229
C	-4.692320	-0.740719	1.917538
C	-3.480083	-1.286675	1.198062
C	-2.591179	-0.086772	0.665490
C	-3.855991	-2.353303	0.158218
C	-4.966794	-2.237429	-0.589819
C	-5.484432	-3.170134	-1.649288
C	-2.917296	-3.536613	0.101475
C	-1.482133	-0.591363	-0.242695
C	-5.621889	4.733126	-0.938157
C	-0.154554	-0.929586	0.330421
O	-1.633410	-0.720885	-1.455990
C	0.207576	-1.095676	1.772373
N	1.589562	-1.230665	1.815577
C	2.151723	-1.489721	0.500502
C	0.988379	-1.052163	-0.401660
O	-0.522262	-1.102222	2.754061
N	3.382171	-0.707483	0.316193
C	3.889915	-0.725626	-1.065914
C	2.943524	-0.101837	-2.094215
S	1.220730	-0.799065	-2.106675
C	5.276506	-0.054601	-1.152629
N	5.988608	-0.073350	0.006743
C	7.253086	0.605039	0.136359
C	7.162661	1.812008	1.057958
O	8.360446	2.429482	1.160056
O	5.696420	0.405280	-2.210663
O	6.168975	2.179240	1.645118
C	2.437083	-2.997245	0.317571
H	-4.078818	0.526180	-0.763958
H	-3.776573	2.180679	1.780147

H	-3.054174	4.006063	-1.730014
H	-4.183499	2.711820	-2.110888
H	-4.078509	4.306792	0.490775
H	-6.028225	2.080551	-0.279214
H	-6.048584	3.016956	1.214035
H	-2.037208	2.639989	0.082192
H	-2.021994	1.752427	-1.444773
H	-5.975713	0.832396	2.449881
H	-5.250155	-1.466458	2.507111
H	-2.862490	-1.787232	1.954762
H	-2.120867	0.332302	1.562407
H	-5.590014	-1.360760	-0.420211
H	-6.492398	-3.524251	-1.395195
H	-5.570705	-2.647411	-2.610827
H	-4.852463	-4.046984	-1.805373
H	-1.880187	-3.220171	-0.057885
H	-2.934539	-4.079568	1.056508
H	-3.167058	-4.243614	-0.691519
H	-5.089304	5.564911	-1.413876
H	-6.214308	4.230688	-1.713354
H	-6.320831	5.157147	-0.207621
H	2.009669	-1.631481	2.644996
H	3.183971	0.252409	0.602966
H	4.062961	-1.773052	-1.338148
H	2.854963	0.975953	-1.926403
H	3.327235	-0.249922	-3.104732
H	5.491838	-0.382380	0.834627
H	7.568149	0.935336	-0.858089
H	8.033073	-0.063432	0.520719
H	8.252141	3.187602	1.759622
H	1.545949	-3.572161	0.579801
H	2.702981	-3.248864	-0.711395
H	3.260620	-3.287612	0.977622

B3LYP Energy = -1950.53348241 a.u.

(3*R*,4*S*,5*R*,8*R*,10*S*,19*S*,24*R*)-**1**, Conf H

C	4.361520	-3.217402	-0.612337
C	5.581468	-3.275044	0.325079
C	6.128415	-1.858832	0.578862
C	5.032986	-0.903166	1.087303
C	3.871135	-0.819911	0.074357
C	3.283789	-2.228286	-0.137150
C	5.564425	0.461601	1.441527
C	4.866476	1.590285	1.279046
C	3.454160	1.672239	0.739803
C	2.841422	0.225586	0.559915
C	3.388505	2.605748	-0.476729
C	2.656772	3.731469	-0.378702
C	2.457565	4.812442	-1.405900
C	4.187694	2.220685	-1.699256
C	1.604813	0.300000	-0.325861
C	6.666311	-4.217141	-0.211194
C	0.278248	0.591387	0.268734
O	1.658771	0.118930	-1.541525
C	-0.031606	1.012012	1.669338
N	-1.417529	0.995967	1.786603
C	-2.068424	0.914091	0.489331
C	-0.906256	0.420439	-0.385180
O	0.735142	1.300011	2.577845
N	-3.207242	-0.013211	0.557582
C	-3.783036	-0.332681	-0.759408
C	-2.824971	-1.056962	-1.707249
S	-1.186351	-0.221224	-1.975601
C	-5.094332	-1.131067	-0.621854
N	-5.712913	-1.015336	0.585101
C	-7.024798	-1.567454	0.826125
C	-8.195868	-0.598787	0.700967
O	-7.842662	0.628874	0.257915
O	-5.536947	-1.789976	-1.557878
O	-9.335233	-0.903944	0.975011
C	-2.533524	2.311771	0.023093
H	4.284731	-0.484506	-0.884812
H	4.630782	-1.351644	2.013944
H	3.927896	-4.220469	-0.718382
H	4.699618	-2.919315	-1.616292
H	5.237624	-3.671587	1.294211
H	6.557830	-1.458258	-0.351993
H	6.949106	-1.901617	1.308200
H	2.849201	-2.583805	0.810096
H	2.473326	-2.195838	-0.871183
H	6.565099	0.509107	1.871455
H	5.310152	2.545679	1.556795
H	2.844759	2.145179	1.518026
H	2.511622	-0.064272	1.564650
H	2.131748	3.903377	0.562546
H	2.760868	5.788875	-1.005796
H	3.015412	4.640044	-2.328786
H	1.396556	4.902272	-1.675018
H	3.725155	1.368251	-2.209867
H	4.266318	3.037722	-2.419136
H	5.203307	1.921647	-1.417204
H	6.277670	-5.232494	-0.352005
H	7.043876	-3.868113	-1.180586
H	7.518368	-4.277111	0.476021
H	-1.839061	1.526663	2.539010
H	-2.876546	-0.867626	1.008096
H	-4.087138	0.613476	-1.221418
H	-2.613257	-2.065266	-1.337525
H	-3.271424	-1.154443	-2.697866
H	-5.289211	-0.380482	1.251190
H	-7.082695	-2.010776	1.824928
H	-7.196437	-2.367053	0.099253
H	-8.660397	1.152433	0.196812
H	-1.702247	3.016158	0.100878
H	-2.872782	2.313722	-1.014889
H	-3.354329	2.648398	0.664342

B3LYP Energy = -1950.53334256 a.u.

(3*R*,4*S*,5*R*,8*R*,10*S*,19*S*,24*R*)-**1**, Conf I

C	-4.149651	3.327224	-0.626686

C	-5.398679	3.443437	0.266444
C	-6.027833	2.056412	0.488974
C	-5.003475	1.039149	1.025731
C	-3.814838	0.900810	0.050649
C	-3.145081	2.277105	-0.123317
C	-5.619014	-0.297616	1.349449
C	-4.978509	-1.461314	1.197566
C	-3.557241	-1.617186	0.700070
C	-2.859787	-0.205079	0.553070
C	-3.504414	-2.542728	-0.523362
C	-2.826767	-3.701236	-0.417917
C	-2.650432	-4.781149	-1.450277
C	-4.254523	-2.111687	-1.761401
C	-1.609134	-0.338001	-0.304935
C	-6.412395	4.444495	-0.301001
C	-0.309820	-0.696208	0.315344
O	-1.624787	-0.146949	-1.519818
C	-0.049560	-1.153232	1.714915
N	1.333961	-1.193607	1.863606
C	2.016643	-1.112294	0.583467
C	0.894090	-0.562185	-0.308586
O	-0.848090	-1.426421	2.599005
N	3.181171	-0.217240	0.694002
C	3.793170	0.105419	-0.604681
C	2.882167	0.881622	-1.559425
S	1.231320	0.093441	-1.884443
C	5.123383	0.848487	-0.415139
N	5.711597	0.716411	0.789713
C	6.954462	1.396881	1.151105
C	8.218281	0.816717	0.486395
O	8.222999	0.812739	-0.854357
O	5.632399	1.490175	-1.346943
O	9.157093	0.420159	1.137517
C	2.452223	-2.514125	0.103592
H	-4.214005	0.598546	-0.925470
H	-4.608590	1.457658	1.969437
H	-3.659013	4.306192	-0.706128
H	-4.465796	3.056640	-1.645454
H	-5.070252	3.815394	1.250536
H	-6.445817	1.685506	-0.459122
H	-6.869555	2.138120	1.190350
H	-2.728273	2.599968	0.843410
H	-2.310477	2.208645	-0.827479
H	-6.633595	-0.294396	1.748129
H	-5.481880	-2.393119	1.452727
H	-2.998604	-2.129677	1.491141
H	-2.540297	0.056340	1.568944
H	-2.334699	-3.905025	0.534724
H	-3.012435	-5.745160	-1.069467
H	-3.171865	-4.573243	-2.386991
H	-1.587634	-4.920425	-1.689466
H	-3.742929	-1.275639	-2.252023
H	-4.350222	-2.917881	-2.491343
H	-5.263106	-1.771089	-1.502249
H	-5.966095	5.438612	-0.420532
H	-6.773239	4.121154	-1.285493
H	-7.283812	4.545784	0.356371
H	1.716425	-1.756410	2.613763
H	2.858033	0.639647	1.146094
H	4.071776	-0.841303	-1.081684
H	2.689238	1.886626	-1.170394
H	3.355054	0.987758	-2.536932
H	5.241025	0.122575	1.463514
H	7.086810	1.320133	2.229350
H	6.870960	2.456012	0.878077
H	7.377650	1.179934	-1.210136
H	1.599462	-3.195048	0.150985
H	2.813103	-2.509953	-0.927208
H	3.247920	-2.886811	0.756385

B3LYP Energy = -1950.53333501 a.u.

(3*R*,4*S*,5*R*,8*R*,10*S*,19*S*,24*R*)-**1**, Conf J

C	3.665928	-3.472592	-0.839060
C	4.845957	-3.808250	0.091164
C	5.629919	-2.530539	0.440998
C	4.714016	-1.434534	1.016336
C	3.594380	-1.079809	0.013990
C	2.772466	-2.345510	-0.294672
C	5.471401	-0.209567	1.459785
C	4.981509	1.031650	1.376417
C	3.609047	1.395776	0.850793
C	2.756326	0.093502	0.571499
C	3.713725	2.409913	-0.296784
C	3.171720	3.629853	-0.122529
C	3.163143	4.795783	-1.073140
C	4.455054	1.983703	-1.541712
C	1.563959	0.436643	-0.310321
C	5.754766	-4.887775	-0.509200
C	0.285592	0.878689	0.299730
O	1.606629	0.347095	-1.536083
C	0.022981	1.252093	1.723718
N	-1.354695	1.415108	1.838298
C	-1.998619	1.497006	0.538467
C	-0.903273	0.911117	-0.364510
O	0.813773	1.379651	2.646692
N	-3.244640	0.711598	0.548564
C	-3.840155	0.546264	-0.787551
C	-2.973213	-0.241751	-1.772282
S	-1.248511	0.412088	-1.995621
C	-5.240209	-0.077411	-0.685160
N	-5.862883	0.056253	0.502186
C	-7.261670	-0.314394	0.716656
C	-7.517140	-1.832658	0.786406
O	-7.141466	-2.537442	-0.290098
O	-5.761801	-0.639471	-1.660238
O	-8.051906	-2.346299	1.741852
C	-2.284779	2.966060	0.158602
H	4.070096	-0.755748	-0.919948
H	4.231307	-1.866679	1.911624
H	3.064524	-4.374075	-1.014750
H	4.062803	-3.169514	-1.819695

H	4.426643	-4.202796	1.030757
H	6.132420	-2.148738	-0.460590
H	6.422774	-2.765768	1.164307
H	2.271967	-2.684197	0.625710
H	1.988485	-2.122741	-1.024467
H	6.461714	-0.365028	1.888072
H	5.582446	1.874145	1.716673
H	3.085350	1.911844	1.663380
H	2.368740	-0.203295	1.553329
H	2.668190	3.819413	0.827033
H	3.703895	4.600374	-2.001539
H	2.134358	5.069869	-1.343031
H	3.608670	5.682609	-0.603790
H	5.408282	1.510588	-1.280804
H	3.868701	1.249973	-2.106811
H	4.668525	2.822746	-2.206866
H	5.194512	-5.805327	-0.723299
H	6.202712	-4.544098	-1.450097
H	6.572170	-5.146144	0.174027
H	-1.708790	1.949754	2.622133
H	-3.017345	-0.203788	0.940776
H	-4.012978	1.548379	-1.197377
H	-2.886484	-1.285397	-1.453291
H	-3.421398	-0.234180	-2.767070
H	-5.338015	0.510358	1.241440
H	-7.871111	0.105083	-0.093680
H	-7.588585	0.115386	1.662320
H	-6.736051	-1.947592	-0.971529
H	-1.375250	3.556879	0.287445
H	-2.603014	3.073956	-0.880411
H	-3.067374	3.361792	0.813772

B3LYP Energy = -1950.53329412 a.u.

(3*R*,4*S*,5*R*,8*R*,10*S*,19*S*,24*S*)-**1**, Conf A

C	4.717526	3.022424	0.408718
C	5.935725	2.878802	-0.521533
C	6.309964	1.394889	-0.684982
C	5.108403	0.547116	-1.142122
C	3.943875	0.662828	-0.134697
C	3.529229	2.141411	-0.013051
C	5.472661	-0.890397	-1.410702
C	4.643679	-1.916024	-1.189750
C	3.232017	-1.795371	-0.655436
C	2.798259	-0.278782	-0.566735
C	3.058655	-2.635583	0.617842
C	2.202264	-3.673664	0.587945
C	1.877159	-4.652907	1.682630
C	3.893049	-2.263010	1.820632
C	1.554880	-0.155645	0.301273
C	7.124978	3.716929	-0.036643
C	0.217775	-0.379709	-0.280698
O	1.614727	0.129674	1.499873
C	-0.142801	-0.704324	-1.687801
N	-1.529230	-0.591801	-1.772066
C	-2.160514	-0.497229	-0.463538
C	-0.942426	-0.216888	0.422559
O	0.581409	-1.000272	-2.628402
N	-3.117351	0.629977	-0.478747
C	-3.482596	1.279646	0.798894
C	-2.950264	0.576752	2.059329
S	-1.123996	0.342371	2.046991
C	-5.010662	1.445085	0.962762
N	-5.764808	0.914522	-0.024493
C	-7.205723	0.991103	-0.005957
C	-7.776631	0.180196	-1.147642
O	-9.124448	0.221824	-1.152728
O	-5.475181	2.027341	1.943075
O	-7.122837	-0.431658	-1.966399
C	-2.858119	-1.828698	-0.121372
H	4.315453	0.340271	0.845803
H	4.761677	0.982612	-2.096774
H	4.405818	4.074330	0.451393
H	5.018196	2.747628	1.431101
H	5.640629	3.253163	-1.515151
H	6.689576	1.003342	0.271091
H	7.129593	1.295309	-1.410052
H	3.138877	2.487865	-0.982554
H	2.721688	2.250597	0.716411
H	6.461020	-1.083369	-1.828430
H	4.970069	-2.932246	-1.407621
H	2.569494	-2.241986	-1.406388
H	2.508403	-0.013407	-1.590400
H	1.660727	-3.845226	-0.343978
H	0.815158	-4.591444	1.955984
H	2.053476	-5.683267	1.347048
H	2.458763	-4.492519	2.592989
H	4.934704	-2.088849	1.529349
H	3.521044	-1.337880	2.275855
H	3.885117	-3.038998	2.588687
H	6.857909	4.777119	0.044007
H	7.462700	3.382963	0.952626
H	7.975977	3.637276	-0.723228
H	-1.999584	-1.067758	-2.532143
H	-2.739579	1.320894	-1.120100
H	-3.081121	2.301008	0.812964
H	-3.154501	1.215320	2.920160
H	-3.454497	-0.374883	2.238627
H	-5.292642	0.453671	-0.796511
H	-7.560265	2.026486	-0.095382
H	-7.609269	0.616019	0.942370
H	-9.434475	-0.308837	-1.906550
H	-2.132092	-2.645546	-0.150433
H	-3.324110	-1.820887	0.864081
H	-3.641822	-2.014564	-0.862478

B3LYP Energy = -1950.53952659 a.u.

(3*R*,4*S*,5*R*,8*R*,10*S*,19*S*,24*S*)-**1**, Conf B

C	4.736489	3.011801	0.601545
C	6.049465	2.829598	-0.181454

C	6.408425	1.336473	-0.284139
C	5.247901	0.504403	-0.861304
C	3.982761	0.657994	0.010638
C	3.586067	2.145675	0.061139
C	5.611161	-0.943119	-1.069411
C	4.744430	-1.950762	-0.924320
C	3.286059	-1.798033	-0.546804
C	2.872943	-0.272001	-0.526799
C	2.959975	-2.613961	0.712031
C	2.092959	-3.638102	0.604427
C	1.637326	-4.596724	1.670412
C	3.668169	-2.237391	1.991786
C	1.548486	-0.118682	0.203840
C	7.193334	3.649896	0.427423
C	0.275040	-0.365529	-0.502446
O	1.482356	0.201876	1.392841
C	0.052683	-0.722264	-1.929285
N	-1.324494	-0.636711	-2.144858
C	-2.072582	-0.533986	-0.900304
C	-0.946886	-0.207392	0.086115
O	0.863341	-1.022243	-2.793238
N	-3.051556	0.572106	-1.027804
C	-3.526315	1.249114	0.199612
C	-3.105646	0.576328	1.518100
S	-1.282606	0.376647	1.677498
C	-5.057753	1.405755	0.210782
N	-5.722440	0.865880	-0.824445
C	-7.160001	1.027965	-1.029067
C	-8.035281	0.210281	-0.059353
O	-7.846350	0.456613	1.244457
O	-5.618907	1.998938	1.147111
O	-8.859905	-0.581180	-0.455723
C	-2.769680	-1.873491	-0.592381
H	4.238827	0.347126	1.031179
H	5.017424	0.932038	-1.854008
H	4.443659	4.069893	0.592141
H	4.912921	2.749718	1.655719
H	5.878927	3.196578	-1.206510
H	6.670931	0.951288	0.712812
H	7.301164	1.210762	-0.912144
H	3.316177	2.481604	-0.952112
H	2.703430	2.283096	0.692151
H	6.634556	-1.159620	-1.376269
H	5.072912	-2.975763	-1.092010
H	2.700764	-2.245264	-1.358970
H	2.695730	-0.018611	-1.579053
H	1.653623	-3.816373	-0.378696
H	1.843808	-5.633439	1.374030
H	2.112170	-4.423819	2.638475
H	0.551524	-4.526095	1.819570
H	4.738059	-2.086754	1.811010
H	3.268933	-1.298600	2.393000
H	3.563624	-3.000953	2.765120
H	6.944020	4.716891	0.460419
H	7.404765	3.325999	1.454318
H	8.116619	3.539582	-0.153235
H	-1.707330	-1.155279	-2.926185
H	-2.629870	1.254092	-1.651926
H	-3.134352	2.274354	0.228304
H	-3.397413	1.226788	2.344138
H	-3.610525	-0.380009	1.667528
H	-5.146445	0.388307	-1.510867
H	-7.402939	0.706526	-2.040814
H	-7.418988	2.088991	-0.921896
H	-7.138166	1.135209	1.375887
H	-2.025249	-2.670737	-0.522006
H	-3.337795	-1.853692	0.337596
H	-3.465809	-2.104219	-1.405211

B3LYP Energy = -1950.53703214 a.u.

(3*R*,4*S*,5*R*,8*R*,10*S*,19*S*,24*S*)-**1**, Conf C

C	4.442493	3.025863	0.618034
C	5.576399	3.107632	-0.419679
C	6.040086	1.695033	-0.817404
C	4.862689	0.818415	-1.284314
C	3.791264	0.704234	-0.178173
C	3.283797	2.114781	0.177353
C	5.299245	-0.533351	-1.786344
C	4.603362	-1.657670	-1.587459
C	3.290045	-1.741150	-0.843337
C	2.699716	-0.283589	-0.646625
C	3.398719	-2.596969	0.428635
C	4.502453	-2.566610	1.195229
C	4.781666	-3.318358	2.466930
C	2.208427	-3.489050	0.696547
C	1.478579	-0.306860	0.254608
C	6.741888	3.971560	0.077772
C	0.133829	-0.503539	-0.326691
O	1.555889	-0.157417	1.476073
C	-0.241496	-0.725829	-1.750545
N	-1.629653	-0.620082	-1.810203
C	-2.245777	-0.629279	-0.491281
C	-1.019195	-0.410619	0.400608
O	0.473750	-0.948659	-2.717440
N	-3.208565	0.490349	-0.409286
C	-3.545659	1.053713	0.916171
C	-3.011448	0.250045	2.113402
S	-1.186355	0.005077	2.069536
C	-5.068672	1.232373	1.110374
N	-5.843846	0.769460	0.105515
C	-7.282556	0.875723	0.144238
C	-7.878606	0.199004	-1.069712
O	-9.224682	0.278283	-1.063883
O	-5.511045	1.764553	2.128582
O	-7.244114	-0.347600	-1.947675
C	-2.933281	-1.986802	-0.244180
H	4.263613	0.285986	0.720862
H	4.397286	1.349105	-2.134851
H	4.063614	4.033776	0.832806
H	4.854718	2.643050	1.563937
H	5.164795	3.583488	-1.324475

H	6.534999	1.214929	0.040393
H	6.792481	1.760334	-1.615543
H	2.783449	2.553718	-0.699864
H	2.542215	2.061516	0.978885
H	6.226680	-0.573634	-2.357964
H	4.982943	-2.602592	-1.972847
H	2.582384	-2.254238	-1.508427
H	2.361233	0.015148	-1.645128
H	5.314924	-1.919096	0.869240
H	5.682977	-3.936812	2.361945
H	4.978192	-2.618597	3.289752
H	3.963141	-3.971328	2.777561
H	2.083174	-4.210102	-0.123043
H	2.298527	-4.052554	1.626857
H	1.277562	-2.913821	0.752264
H	6.407447	4.985508	0.326626
H	7.193018	3.539636	0.980130
H	7.529390	4.055293	-0.680368
H	-2.105099	-1.042721	-2.598139
H	-2.849548	1.223994	-1.012918
H	-3.126070	2.064460	1.001013
H	-3.205020	0.820660	3.023097
H	-3.521640	-0.709328	2.219639
H	-5.389157	0.351632	-0.700801
H	-7.611281	1.922944	0.165177
H	-7.691424	0.414095	1.051494
H	-9.551280	-0.167371	-1.864313
H	-2.205295	-2.796368	-0.344891
H	-3.387234	-2.056571	0.744451
H	-3.725763	-2.118930	-0.987520

B3LYP Energy = -1950.53696538 a.u.

(3*R*,4*S*,5*R*,8*R*,10*S*,19*S*,24*S*)-**1**, Conf D

C	4.465673	3.230008	0.679834
C	5.741680	3.222862	-0.181666
C	6.233424	1.780108	-0.396231
C	5.125066	0.875840	-0.966862
C	3.901786	0.855073	-0.024381
C	3.371454	2.291798	0.143591
C	5.608710	-0.514498	-1.288830
C	4.849254	-1.608104	-1.167281
C	3.407936	-1.619284	-0.703500
C	2.856207	-0.144221	-0.566128
C	3.239019	-2.533427	0.518556
C	2.478295	-3.636901	0.392444
C	2.186796	-4.697644	1.418660
C	3.977106	-2.155664	1.781016
C	1.563785	-0.167691	0.233495
C	6.836320	4.115079	0.416321
C	0.287439	-0.508024	-0.428300
O	1.527261	0.076247	1.441688
C	0.029967	-0.801512	-1.864290
N	-1.357163	-0.861743	-2.008919
C	-2.045133	-0.916498	-0.728007
C	-0.911417	-0.522914	0.224486
O	0.821496	-0.955080	-2.782589
N	-3.149534	0.072647	-0.739068
C	-3.614625	0.635637	0.547594
C	-3.064332	-0.069079	1.799255
S	-1.224486	-0.078431	1.865418
C	-5.150414	0.647789	0.647046
N	-5.823150	0.070864	-0.362734
C	-7.275278	-0.092633	-0.373005
C	-8.066954	1.210809	-0.599084
O	-7.834229	2.194577	0.280012
O	-5.707467	1.180718	1.621342
O	-8.871422	1.319769	-1.495916
C	-2.569610	-2.343381	-0.471807
H	4.241966	0.509704	0.959809
H	4.801186	1.338348	-1.916815
H	4.076027	4.253483	0.756570
H	4.727703	2.921425	1.703226
H	5.477123	3.629151	-1.171307
H	6.584980	1.365544	0.560835
H	7.098013	1.777735	-1.074189
H	3.013172	2.660331	-0.830082
H	2.517199	2.304318	0.826516
H	6.628398	-0.611611	-1.661896
H	5.260617	-2.584649	-1.419533
H	2.819007	-2.075032	-1.508146
H	2.599750	0.152197	-1.590392
H	2.003735	-3.805890	-0.575914
H	2.704187	-4.535977	2.366691
H	1.111033	-4.744883	1.635274
H	2.472589	-5.689061	1.043640
H	3.508732	-1.286573	2.257397
H	3.993990	-2.966869	2.511424
H	5.014251	-1.885148	1.554956
H	6.488837	5.148480	0.530427
H	7.138063	3.754261	1.407724
H	7.729847	4.130548	-0.218635
H	-1.724100	-1.364661	-2.807753
H	-2.850231	0.827214	-1.350432
H	-3.314183	1.688878	0.619768
H	-3.382182	0.493537	2.678577
H	-3.456652	-1.082699	1.902167
H	-5.251361	-0.285736	-1.122107
H	-7.592092	-0.527992	0.583132
H	-7.538915	-0.778553	-1.176803
H	-7.156428	1.919747	0.946279
H	-1.737831	-3.052497	-0.482485
H	-3.088109	-2.438723	0.482590
H	-3.275647	-2.606836	-1.265993

B3LYP Energy = -1950.53657872 a.u.

(3*R*,4*S*,5*R*,8*R*,10*S*,19*S*,24*S*)-**1**, Conf E

C	-4.810018	-2.927630	0.570763
C	-6.088272	-2.729048	-0.264307
C	-6.407629	-1.230016	-0.405888
C	-5.206834	-0.435503	-0.952387

C	-3.978866	-0.602815	-0.031312
C	-3.619976	-2.097959	0.059649
C	-5.527438	1.016412	-1.197977
C	-4.642761	2.005999	-1.037046
C	-3.203879	1.825589	-0.601241
C	-2.828318	0.291593	-0.543352
C	-2.908305	2.653579	0.657250
C	-2.015787	3.657328	0.568602
C	-1.578559	4.619711	1.639005
C	-3.670212	2.309468	1.915232
C	-1.533624	0.116470	0.235857
C	-7.273209	-3.512089	0.314326
C	-0.231728	0.301926	-0.434242
O	-1.520496	-0.174868	1.434268
C	0.043823	0.620274	-1.860824
N	1.420149	0.474363	-2.036412
C	2.131836	0.366371	-0.770492
C	0.967459	0.109635	0.192162
O	-0.730891	0.936325	-2.752899
N	3.066310	-0.779071	-0.847891
C	3.486876	-1.445555	0.406395
C	3.058124	-0.726749	1.696282
S	1.241242	-0.450163	1.802909
C	5.015219	-1.664713	0.478472
N	5.721201	-1.192828	-0.579649
C	7.159329	-1.271748	-0.642833
C	7.916470	0.025483	-0.386820
O	7.132910	1.043955	0.038094
O	5.512834	-2.244526	1.441067
O	9.112040	0.133790	-0.546529
C	2.873943	1.683578	-0.471330
H	-4.264966	-0.267958	0.973479
H	-4.949679	-0.885446	-1.928484
H	-4.542283	-3.992243	0.589878
H	-5.019654	-2.643473	1.613095
H	-5.887862	-3.117826	-1.275795
H	-6.698426	-0.821120	0.573652
H	-7.272958	-1.095058	-1.069473
H	-3.320362	-2.458291	-0.936700
H	-2.764942	-2.243911	0.725762
H	-6.533267	1.251330	-1.546766
H	-4.940253	3.035279	-1.234101
H	-2.577606	2.246898	-1.396598
H	-2.621782	0.017439	-1.584827
H	-1.534799	3.811458	-0.398945
H	-0.503639	4.516514	1.839690
H	-1.737345	5.657187	1.316924
H	-2.102818	4.478878	2.586633
H	-3.582886	3.084276	2.679580
H	-4.734792	2.171501	1.696940
H	-3.299644	1.371818	2.345369
H	-7.049610	-4.583498	0.376444
H	-7.516530	-3.164153	1.326215
H	-8.170784	-3.392593	-0.303702
H	1.846159	0.953953	-2.820015
H	2.633256	-1.459465	-1.465612
H	3.050939	-2.452119	0.444631
H	3.303211	-1.369664	2.542984
H	3.597580	0.211511	1.838358
H	5.182115	-0.695930	-1.280232
H	7.493076	-1.646933	-1.615543
H	7.489358	-1.987574	0.117278
H	7.721848	1.803677	0.188615
H	2.160713	2.511489	-0.439826
H	3.415186	1.659010	0.474114
H	3.602790	1.865433	-1.267565

B3LYP Energy = -1950.53627901 a.u.

(3*R*,4*S*,5*R*,8*R*,10*S*,19*S*,24*S*)-**1**, Conf F

C	-4.479472	-3.143547	0.878323
C	-5.723618	-3.211053	-0.026391
C	-6.195960	-1.793141	-0.395298
C	-5.059717	-0.949626	-1.003092
C	-3.876172	-0.844071	-0.016957
C	-3.360533	-2.260590	0.301144
C	-5.521894	0.405559	-1.472738
C	-4.763285	1.505189	-1.418308
C	-3.343787	1.558445	-0.893934
C	-2.803684	0.102349	-0.599549
C	-3.226511	2.580621	0.245622
C	-2.459006	3.669597	0.052681
C	-2.211009	4.820899	0.988710
C	-4.021846	2.318201	1.502325
C	-1.548850	0.196259	0.255245
C	-6.846453	-4.037488	0.612636
C	-0.241219	0.469940	-0.372724
O	-1.575100	0.064167	1.481237
C	0.083465	0.631545	-1.815499
N	1.475101	0.676538	-1.901701
C	2.105647	0.846273	-0.600242
C	0.927559	0.542566	0.331199
O	-0.664995	0.702049	-2.780211
N	3.206298	-0.135343	-0.471685
C	3.606364	-0.588533	0.880306
C	3.008196	0.231309	2.033745
S	1.166777	0.256081	2.017501
C	5.140331	-0.621209	1.063749
N	5.861329	-0.112485	0.032262
C	7.302107	-0.169188	-0.000180
C	7.909877	-1.228325	-0.912165
O	7.000152	-2.073987	-1.447265
O	5.629326	-1.075652	2.095290
O	9.097876	-1.303878	-1.135148
C	2.618935	2.292488	-0.449880
H	-4.255605	-0.412141	0.917464
H	-4.699861	-1.499823	-1.891429
H	-4.099579	-4.157326	1.060979
H	-4.776537	-2.743912	1.859784
H	-5.427095	-3.710730	-0.962792
H	-6.582238	-1.288743	0.503345
H	-7.033062	-1.852461	-1.104535

H	-2.967922	-2.717044	-0.620742
H	-2.533024	-2.212404	1.014590
H	-6.523662	0.468715	-1.898203
H	-5.159057	2.454507	-1.777308
H	-2.718457	1.938924	-1.710169
H	-2.501942	-0.284765	-1.580216
H	-1.941197	3.749600	-0.904805
H	-2.766081	4.743771	1.926102
H	-1.144976	4.893436	1.242917
H	-2.483247	5.772528	0.513620
H	-5.051281	2.036708	1.254838
H	-3.583874	1.489632	2.070631
H	-4.061410	3.188842	2.159892
H	-6.512099	-5.057458	0.835421
H	-7.179961	-3.583824	1.554505
H	-7.717123	-4.107793	-0.049832
H	1.880845	1.104925	-2.724692
H	2.940473	-0.939840	-1.032323
H	3.283930	-1.627057	1.029529
H	3.282548	-0.250344	2.973651
H	3.405017	1.248111	2.061085
H	5.322759	0.159297	-0.783074
H	7.652856	-0.382264	1.014788
H	7.730051	0.792394	-0.301291
H	7.494156	-2.713388	-1.989191
H	1.791280	2.995589	-0.575339
H	3.081793	2.477048	0.519762
H	3.371187	2.480617	-1.222702

B3LYP Energy = -1950.53606212 a.u.

(3*R*,4*S*,5*R*,8*R*,10*S*,19*S*,24*S*)-**1**, Conf G

C	4.679929	3.042568	0.426393
C	5.895866	2.919184	-0.509580
C	6.285524	1.440653	-0.685103
C	5.091474	0.583312	-1.144344
C	3.929038	0.679307	-0.132458
C	3.499330	2.152488	0.002033
C	5.469983	-0.848327	-1.424413
C	4.652473	-1.884200	-1.208523
C	3.241013	-1.782218	-0.669662
C	2.791946	-0.270846	-0.568378
C	3.079611	-2.633555	0.597798
C	2.234467	-3.680652	0.561980
C	1.922930	-4.671926	1.649757
C	3.913202	-2.261317	1.801246
C	1.550101	-0.166937	0.304023
C	7.077837	3.766578	-0.022973
C	0.212992	-0.397961	-0.276271
O	1.609817	0.108987	1.504646
C	-0.148235	-0.719910	-1.683199
N	-1.536633	-0.617594	-1.764083
C	-2.164941	-0.532722	-0.453428
C	-0.946601	-0.247051	0.430795
O	0.574495	-1.007924	-2.627082
N	-3.131787	0.587138	-0.462657
C	-3.489275	1.235410	0.819420
C	-2.954873	0.527061	2.075160
S	-1.127776	0.299418	2.059376
C	-5.015577	1.409513	0.988985
N	-5.778176	0.891441	-0.000234
C	-7.221426	1.011927	0.026991
C	-7.923259	0.292744	-1.104941
O	-7.073987	-0.307046	-1.977879
O	-5.472222	1.989217	1.973774
O	-9.125710	0.258207	-1.230372
C	-2.850721	-1.870671	-0.113057
H	4.306862	0.352910	0.844348
H	4.737185	1.022005	-2.094748
H	4.357265	4.090692	0.478818
H	4.987402	2.762611	1.445332
H	5.592849	3.297520	-1.499301
H	6.672726	1.046459	0.266812
H	7.103543	1.355058	-1.413725
H	3.101948	2.502298	-0.963412
H	2.693378	2.247907	0.735203
H	6.459136	-1.027882	-1.846166
H	4.988825	-2.895376	-1.434478
H	2.581039	-2.230080	-1.422119
H	2.496585	-0.000566	-1.589205
H	1.692955	-3.851231	-0.370182
H	2.112786	-5.697439	1.306792
H	2.502736	-4.510329	2.561014
H	0.860353	-4.626597	1.924002
H	4.952376	-2.074914	1.508719
H	3.533374	-1.343129	2.263964
H	3.914728	-3.042748	2.563748
H	6.799810	4.823267	0.065963
H	7.422508	3.429360	0.962774
H	7.927210	3.700769	-0.713001
H	-2.002248	-1.101087	-2.522197
H	-2.758738	1.281603	-1.103263
H	-3.082603	2.254735	0.834676
H	-3.160194	1.161230	2.939014
H	-3.456658	-0.426616	2.250612
H	-5.299244	0.450224	-0.777296
H	-7.532339	2.063677	-0.014699
H	-7.624090	0.620935	0.968375
H	-7.618807	-0.726879	-2.665795
H	-2.118081	-2.681489	-0.143920
H	-3.316920	-1.868453	0.872243
H	-3.633069	-2.062247	-0.854292

B3LYP Energy = -1950.53552809 a.u.

(3*R*,4*S*,5*R*,8*R*,10*S*,19*R*,24*R*)-**3**, Conf A

C	-4.253258	0.348570	-2.848422
C	-5.707662	0.324735	-2.343763
C	-5.816945	-0.525570	-1.065220
C	-4.817326	-0.069843	0.014098
C	-3.369797	-0.147000	-0.517933

C	-3.244633	0.754622	-1.760619
C	-4.976111	-0.814299	1.314734
C	-3.955705	-1.111505	2.126086
C	-2.507976	-0.746003	1.873850
C	-2.387013	0.207426	0.619369
C	-1.621870	-1.999452	1.877151
C	-0.675822	-2.112953	2.828025
C	0.289331	-3.242966	3.061869
C	-1.896502	-3.052927	0.829766
C	-0.935101	0.271581	0.173958
C	-6.679007	-0.152554	-3.430441
C	-0.010467	1.196013	0.828840
O	-0.509634	-0.456145	-0.742523
C	-0.291548	2.206633	1.873575
N	0.859918	2.991335	1.978441
C	1.981984	2.424598	1.269716
C	1.301109	1.363492	0.429185
O	-1.303021	2.391746	2.538449
S	2.716490	3.662482	0.078092
C	3.753078	2.443548	-0.813497
C	2.945341	1.299187	-1.450131
N	1.931301	0.698754	-0.566080
C	3.029587	1.854017	2.242342
H	-3.184168	-1.180068	-0.836999
H	-5.036565	0.994203	0.215851
C	3.941867	0.256556	-1.987651
N	3.989483	-0.930850	-1.337444
C	4.920403	-1.965686	-1.729436
C	4.939851	-3.053932	-0.679075
O	4.664658	0.536237	-2.941710
O	5.793528	-4.042094	-1.011294
O	4.274074	-3.053513	0.335013
H	-4.170779	1.027717	-3.707199
H	-3.990843	-0.653510	-3.220576
H	-5.982104	1.357343	-2.074354
H	-5.632578	-1.582870	-1.309307
H	-6.839976	-0.470952	-0.668063
H	-3.418946	1.800971	-1.466688
H	-2.231172	0.700747	-2.168478
H	-5.988388	-1.096209	1.605341
H	-4.141862	-1.649912	3.054646
H	-2.180842	-0.139966	2.727213
H	-2.652149	1.199997	1.001846
H	-0.578805	-1.285911	3.533386
H	0.137023	-4.088151	2.387069
H	0.206776	-3.615274	4.091377
H	1.326150	-2.904346	2.934175
H	-1.567984	-2.710826	-0.158474
H	-1.385741	-3.994155	1.043207
H	-2.970106	-3.260460	0.762704
H	-6.618919	0.480230	-4.323611
H	-6.450380	-1.181547	-3.735678
H	-7.716016	-0.133296	-3.075316
H	0.993476	3.549280	2.811158
H	4.260120	2.984231	-1.614795
H	4.525314	2.057484	-0.143008
H	2.431046	1.700356	-2.333035
H	1.258453	0.074278	-1.029371
H	3.853717	1.358432	1.725768
H	3.442349	2.664289	2.850234
H	2.550549	1.120860	2.899176
H	3.391400	-1.105126	-0.537363
H	5.929952	-1.556418	-1.851870
H	4.652275	-2.410214	-2.696601
H	5.759252	-4.713171	-0.308004

B3LYP Energy = -1950.55502984 a.u.

(3*R*,4*S*,5*R*,8*R*,10*S*,19*R*,24*R*)-**3**, Conf B

C	-4.571670	0.753817	-2.665609
C	-5.992066	0.649024	-2.080220
C	-6.024560	-0.381465	-0.936934
C	-4.958018	-0.083639	0.132841
C	-3.546154	-0.076109	-0.494235
C	-3.494622	0.999898	-1.595839
C	-5.041790	-1.009363	1.318782
C	-3.976029	-1.422535	2.012337
C	-2.543731	-1.026264	1.721936
C	-2.494017	0.107016	0.620477
C	-1.681336	-2.272075	1.473533
C	-0.695333	-2.553336	2.345745
C	0.252130	-3.722301	2.347282
C	-2.027107	-3.135343	0.283124
C	-1.067924	0.239468	0.112110
C	-7.031933	0.333896	-3.162331
C	-0.096240	1.021695	0.873591
O	-0.698501	-0.329369	-0.932792
C	-0.314048	1.866237	2.069725
N	0.878643	2.563862	2.278900
C	1.966415	2.043475	1.483571
C	1.219921	1.182347	0.484976
O	-1.309496	1.995678	2.771433
S	2.799847	3.401404	0.510387
C	3.763011	2.266331	-0.555165
C	2.888584	1.300091	-1.376205
N	1.803066	0.657512	-0.615637
C	2.959458	1.245762	2.349539
H	-3.388180	-1.049509	-0.975107
H	-5.159548	0.938307	0.501479
C	3.835747	0.274040	-2.022646
N	3.741039	-1.005307	-1.563562
C	4.732599	-1.996599	-1.897941
C	5.788975	-2.142971	-0.809192
O	4.634500	0.637299	-2.879745
O	6.705588	-3.073427	-1.151885
O	5.815582	-1.529237	0.234960
H	-4.539879	1.552296	-3.418458
H	-4.338321	-0.181499	-3.196899
H	-6.244288	1.628926	-1.643765
H	-5.859313	-1.390185	-1.345250
H	-7.020738	-0.391787	-0.473404
H	-3.644164	1.990774	-1.140649

H	-2.508698	1.010291	-2.069444
H	-6.036704	-1.329424	1.629178
H	-4.108431	-2.090163	2.862881
H	-2.150782	-0.560240	2.633517
H	-2.728825	1.028898	1.165250
H	-0.549826	-1.857123	3.173215
H	0.081035	-4.418613	1.523345
H	0.171412	-4.286537	3.285668
H	1.293798	-3.379700	2.281801
H	-1.517348	-4.100782	0.305964
H	-3.105237	-3.326006	0.245020
H	-1.751688	-2.631891	-0.650613
H	-7.025497	1.093410	-3.952765
H	-6.828040	-0.636728	-3.631969
H	-8.044280	0.294857	-2.743472
H	1.047792	2.965695	3.191515
H	4.326269	2.886219	-1.254770
H	4.488930	1.719318	0.051367
H	2.436639	1.864285	-2.201811
H	1.092737	0.169400	-1.175790
H	3.746726	0.769655	1.762425
H	3.429380	1.915910	3.075253
H	2.416368	0.462191	2.888009
H	3.128549	-1.168601	-0.774619
H	5.225543	-1.695519	-2.826690
H	4.269474	-2.974258	-2.069363
H	7.355376	-3.130947	-0.430410

B3LYP Energy = -1950.55481664 a.u.

(3*R*,4*S*,5*R*,8*R*,10*S*,19*R*,24*R*)-**3**, Conf C

C	4.067380	-0.333138	-2.850255
C	5.531335	-0.469065	-2.392919
C	5.743410	0.260380	-1.054166
C	4.734600	-0.200499	0.014681
C	3.286509	0.043885	-0.463080
C	3.054228	-0.742210	-1.767214
C	4.992056	0.409056	1.367932
C	4.022454	0.776816	2.211842
C	2.541735	0.638950	1.940942
C	2.313786	-0.301991	0.685732
C	1.836625	2.004039	1.893883
C	2.425646	3.072606	1.330504
C	1.890420	4.470016	1.185206
C	0.486953	2.042465	2.572885
C	0.855975	-0.293175	0.264663
C	6.510065	0.017422	-3.468832
C	-0.079354	-1.259834	0.844401
O	0.431874	0.524254	-0.572570
C	0.189983	-2.355083	1.804379
N	-0.967547	-3.137236	1.839701
C	-2.082866	-2.505844	1.175838
C	-1.391448	-1.382762	0.430226
O	1.195949	-2.599176	2.457946
S	-2.818239	-3.633537	-0.118517
C	-3.846327	-2.337011	-0.906028
C	-3.030167	-1.146361	-1.439751
N	-2.014937	-0.629470	-0.504517
C	-3.131273	-2.012879	2.189051
H	3.175213	1.111894	-0.694147
H	4.865565	-1.292943	0.118137
C	-4.019373	-0.055891	-1.888114
N	-4.067596	1.069026	-1.135101
C	-4.991587	2.138951	-1.439829
C	-5.004647	3.135683	-0.302167
O	-4.737823	-0.247033	-2.867009
O	-5.855269	4.151032	-0.548855
O	-4.336692	3.048132	0.706731
H	3.906254	-0.930547	-3.757290
H	3.881113	0.714296	-3.132530
H	5.726426	-1.539230	-2.216953
H	5.642380	1.345860	-1.205743
H	6.767743	0.089224	-0.695277
H	3.145611	-1.819997	-1.563455
H	2.040545	-0.570452	-2.139903
H	6.033560	0.529851	1.666605
H	4.281879	1.217162	3.173245
H	2.113038	0.093981	2.792999
H	2.541384	-1.310213	1.048682
H	3.424421	2.930120	0.921086
H	0.883499	4.596925	1.589248
H	1.863287	4.761290	0.126973
H	2.546976	5.191591	1.689590
H	-0.176512	1.256823	2.194684
H	0.601038	1.859120	3.650444
H	-0.029345	2.996156	2.449951
H	6.374358	-0.531699	-4.407944
H	6.359580	1.083353	-3.681941
H	7.550963	-0.114888	-3.151127
H	-1.109097	-3.760128	2.623655
H	-4.352848	-2.804449	-1.752426
H	-4.619003	-2.005611	-0.207327
H	-2.514877	-1.473448	-2.352023
H	-1.336362	0.028492	-0.910351
H	-3.947551	-1.465720	1.713888
H	-3.555375	-2.869091	2.721452
H	-2.650392	-1.345125	2.911217
H	-3.471676	1.172022	-0.321201
H	-6.003819	1.748485	-1.596666
H	-4.719305	2.661060	-2.366238
H	-5.817291	4.761381	0.207585

B3LYP Energy = -1950.55234402 a.u.

(3*R*,4*S*,5*R*,8*R*,10*S*,19*R*,24*R*)-**3**, Conf D

C	-4.203850	0.475325	-2.819659
C	-5.657405	0.648622	-2.342401
C	-5.885656	-0.124814	-1.031224
C	-4.850768	0.254963	0.044564
C	-3.417057	-0.025043	-0.457068
C	-3.166667	0.802958	-1.731819
C	-5.120080	-0.396395	1.375684

C	-4.158187	-0.832406	2.195502
C	-2.675575	-0.739456	1.915902
C	-2.421852	0.237822	0.694167
C	-2.023336	-2.127715	1.812986
C	-2.656314	-3.152254	1.216493
C	-2.176491	-4.562939	1.015857
C	-0.671031	-2.242149	2.478232
C	-0.968818	0.189425	0.258818
C	-6.663815	0.243647	-3.426485
C	0.010848	1.083610	0.877919
O	-0.586225	-0.600275	-0.624553
C	-0.199786	2.124306	1.910932
N	0.987168	2.857181	1.974362
C	2.065562	2.232457	1.246023
C	1.319676	1.184263	0.447076
O	-1.185533	2.361858	2.597278
S	2.803363	3.413807	0.001484
C	3.783285	2.138803	-0.874707
C	2.930266	0.995127	-1.457263
N	1.896235	0.470210	-0.547078
C	3.121420	1.642356	2.198022
H	-3.348676	-1.086585	-0.730833
H	-4.938928	1.346726	0.191769
C	3.899951	-0.100087	-1.934423
N	3.856089	-1.280843	-1.257126
C	4.852625	-2.302615	-1.458108
C	5.905368	-2.303488	-0.357009
O	4.666791	0.129388	-2.864237
O	6.823430	-3.270265	-0.572112
O	5.928510	-1.558681	0.598140
H	-4.027802	1.101720	-3.704055
H	-4.060160	-0.566350	-3.144932
H	-5.809693	1.717547	-2.122300
H	-5.827724	-1.206438	-1.226559
H	-6.899566	0.070733	-0.655637
H	-3.215964	1.874493	-1.484968
H	-2.163429	0.608424	-2.120965
H	-6.162889	-0.488964	1.679777
H	-4.426090	-1.298696	3.142239
H	-2.220401	-0.241365	2.782633
H	-2.606456	1.239481	1.097994
H	-3.651864	-2.957430	0.821082
H	-2.864679	-5.277462	1.486721
H	-1.178190	-4.747806	1.419345
H	-2.154271	-4.811388	-0.053388
H	-0.766048	-2.072343	3.559640
H	-0.204643	-3.218708	2.334574
H	0.025726	-1.482090	2.106770
H	-6.516532	0.825968	-4.343598
H	-6.555740	-0.817326	-3.685192
H	-7.695910	0.401384	-3.092141
H	1.166243	3.425736	2.791163
H	4.286107	2.638645	-1.704369
H	4.560432	1.751789	-0.211263
H	2.427534	1.370365	-2.357745
H	1.185565	-0.131761	-0.983619
H	3.901013	1.087738	1.672488
H	3.595148	2.450361	2.763026
H	2.631337	0.958114	2.898452
H	3.264965	-1.323415	-0.436842
H	5.345532	-2.119910	-2.417338
H	4.395823	-3.297396	-1.504213
H	7.470332	-3.232669	0.153244

B3LYP Energy = -1950.55215621 a.u.

(3*R*,4*S*,5*R*,8*R*,10*S*,19*R*,24*R*)-**3**, Conf E

C	-4.366406	-0.478698	2.849429
C	-5.821075	-0.479326	2.345227
C	-5.954652	0.410836	1.096477
C	-4.943114	0.020608	0.002618
C	-3.498103	0.121310	0.537749
C	-3.347324	-0.818972	1.748966
C	-5.124656	0.803958	-1.271628
C	-4.114203	1.159537	-2.071668
C	-2.656258	0.827620	-1.832443
C	-2.504792	-0.162830	-0.609851
C	-1.808214	2.106647	-1.796332
C	-0.877923	2.285070	-2.752856
C	0.044479	3.455951	-2.958261
C	-2.105330	3.112259	-0.708806
C	-1.051368	-0.195039	-0.166890
C	-6.805550	-0.068208	3.446943
C	-0.093047	-1.050959	-0.865079
O	-0.650709	0.505274	0.781918
C	-0.334413	-2.015039	-1.963188
N	0.850160	-2.739452	-2.114262
C	1.949563	-2.163334	-1.377627
C	1.225150	-1.184213	-0.476280
O	-1.340702	-2.207745	-2.633534
S	2.750695	-3.430637	-0.264510
C	3.734583	-2.216471	0.690567
C	2.877176	-1.153018	1.398585
N	1.829415	-0.549131	0.555391
C	2.962359	-1.486438	-2.319395
H	-3.343331	1.147590	0.893415
H	-5.132661	-1.041519	-0.235594
C	3.832664	-0.099665	1.986652
N	3.781619	1.141306	1.430035
C	4.700239	2.187102	1.815209
C	5.901419	2.394742	0.899701
O	4.596488	-0.412138	2.894445
O	5.991277	1.485697	-0.099483
O	6.699363	3.291888	1.052569
H	-4.263910	-1.184108	3.684451
H	-4.132371	0.517121	3.255686
H	-6.065785	-1.509470	2.040052
H	-5.799925	1.463854	1.376886
H	-6.975881	0.341151	0.697181
H	-3.493043	-1.859068	1.419410
H	-2.335560	-0.751224	2.158944
H	-6.144979	1.065233	-1.553032

H	-4.316710	1.723340	-2.981526
H	-2.312957	0.259721	-2.705249
H	-2.736792	-1.150126	-1.026399
H	-0.765219	1.489674	-3.491476
H	-0.090611	4.248629	-2.219005
H	-0.102442	3.896565	-3.953138
H	1.094862	3.137318	-2.913974
H	-1.616674	4.072704	-0.884783
H	-3.183332	3.292966	-0.635104
H	-1.770673	2.738742	0.265828
H	-6.728398	-0.731043	4.316660
H	-6.605767	0.954949	3.789744
H	-7.841421	-0.103395	3.089842
H	1.003483	-3.248574	-2.974389
H	4.274388	-2.776952	1.455892
H	4.481272	-1.752216	0.041587
H	2.388761	-1.628807	2.258795
H	1.128917	0.011550	1.057916
H	3.762637	-0.978973	-1.777316
H	3.412849	-2.239468	-2.972473
H	2.442386	-0.744613	-2.934329
H	3.163335	1.278460	0.641300
H	5.094535	1.945150	2.806672
H	4.183362	3.148580	1.890049
H	6.795208	1.703010	-0.602731

B3LYP Energy = -1950.55208366 a.u.

(3*R*,4*S*,5*R*,8*R*,10*S*,19*R*,24*R*)-**3**, Conf F

C	3.796266	2.364303	-2.184721
C	5.312144	2.166845	-1.999063
C	5.617233	1.679598	-0.570968
C	4.801469	0.425521	-0.207482
C	3.287622	0.706550	-0.327579
C	2.971828	1.132359	-1.773872
C	5.162865	-0.136759	1.143327
C	4.282901	-0.728789	1.957698
C	2.814048	-0.929454	1.650855
C	2.499677	-0.531129	0.154853
C	1.939555	-0.285786	2.735564
C	1.155466	-1.083620	3.484069
C	0.233990	-0.704281	4.611122
C	2.040597	1.210264	2.919281
C	0.994629	-0.413084	-0.028197
C	6.098419	3.436342	-2.348564
C	0.201108	-1.606170	-0.285735
O	0.420744	0.692240	0.051352
C	0.649471	-2.990236	-0.536737
N	-0.477116	-3.697045	-0.978718
C	-1.701224	-2.959402	-0.773827
C	-1.170482	-1.571085	-0.488882
O	1.760793	-3.493960	-0.424681
S	-2.671278	-2.816129	-2.364493
C	-3.872096	-1.598061	-1.708363
C	-3.243282	-0.309587	-1.142351
N	-1.945952	-0.479675	-0.474804
C	-2.534625	-3.544676	0.380051
H	3.050846	1.554701	0.326867
H	5.053999	-0.342523	-0.960652
C	-4.223993	0.317567	-0.121478
N	-4.395210	1.658405	-0.233638
C	-5.237718	2.390823	0.688192
C	-5.265125	3.848847	0.286153
O	-4.785352	-0.356386	0.737615
O	-6.033151	4.575983	1.117990
O	-4.671669	4.314226	-0.664698
H	3.582999	2.626129	-3.229613
H	3.475243	3.225403	-1.578837
H	5.630343	1.370404	-2.690963
H	5.391281	2.481980	0.147803
H	6.690380	1.464996	-0.472091
H	3.193053	0.295477	-2.453647
H	1.906439	1.357001	-1.878550
H	6.208429	-0.069935	1.445216
H	4.612797	-1.116482	2.920872
H	2.622322	-2.007845	1.707204
H	2.829274	-1.395060	-0.434025
H	1.170942	-2.150870	3.257172
H	0.271444	0.358345	4.861501
H	0.476069	-1.272481	5.518792
H	-0.807236	-0.947448	4.359777
H	1.546541	1.732720	2.091940
H	1.579886	1.547051	3.850351
H	3.088589	1.529254	2.927985
H	5.902857	3.754347	-3.379333
H	5.818955	4.265244	-1.685880
H	7.178369	3.277779	-2.246022
H	-0.465984	-4.705733	-0.904272
H	-4.520460	-1.328895	-2.546270
H	-4.494663	-2.067176	-0.946182
H	-3.075785	0.393726	-1.969905
H	-1.384886	0.362764	-0.300953
H	-3.401559	-2.928951	0.625947
H	-2.878298	-4.548080	0.111230
H	-1.905005	-3.610465	1.273406
H	-3.932453	2.200945	-0.953560
H	-4.871756	2.306415	1.718845
H	-6.260323	1.994979	0.691580
H	-6.009634	5.500306	0.815572

B3LYP Energy = -1950.55191338 a.u.

(3*R*,4*S*,5*R*,8*R*,10*S*,19*R*,24*R*)-**3**, Conf G

C	-4.228918	-1.536879	-2.604324
C	-5.706405	-1.245164	-2.283972
C	-5.911109	-1.130689	-0.762892
C	-4.949706	-0.107877	-0.129521
C	-3.481270	-0.493276	-0.411533
C	-3.260630	-0.546434	-1.935317
C	-5.201357	0.099802	1.341574
C	-4.235674	0.364331	2.227560
C	-2.764978	0.504136	1.895487

C	-2.547003	0.488686	0.329098
C	-1.924951	-0.481471	2.720038
C	-1.041209	0.014849	3.606179
C	-0.138462	-0.730541	4.551440
C	-2.178573	-1.956706	2.516969
C	-1.072934	0.277019	0.033746
C	-6.640875	-2.289073	-2.907721
C	-0.151468	1.415048	0.061397
O	-0.619660	-0.854261	-0.222240
C	-0.454339	2.854418	0.248688
N	0.727179	3.540823	-0.038828
C	1.865489	2.661636	-0.155490
C	1.189559	1.308701	-0.238799
O	-1.500984	3.401489	0.568417
S	2.747723	2.921180	-1.782333
C	3.773717	1.409270	-1.663346
C	2.940746	0.118922	-1.558041
N	1.852767	0.169934	-0.565752
C	2.810028	2.803033	1.051412
H	-3.318444	-1.503555	-0.016181
H	-5.139533	0.856607	-0.634113
C	3.896294	-1.048823	-1.288593
N	3.816325	-1.657550	-0.088790
C	4.603201	-2.843319	0.253661
C	6.091848	-2.556761	0.530363
O	4.722999	-1.373851	-2.154062
O	6.773210	-1.992373	-0.477267
O	6.614641	-2.848709	1.580982
H	-4.077707	-1.530460	-3.691690
H	-3.987513	-2.555568	-2.264566
H	-5.953893	-0.265036	-2.722459
H	-5.755283	-2.114696	-0.295165
H	-6.949582	-0.843868	-0.547567
H	-3.410926	0.458889	-2.357494
H	-2.230894	-0.838697	-2.160781
H	-6.236569	0.052190	1.679748
H	-4.488094	0.507070	3.277547
H	-2.460654	1.507003	2.218216
H	-2.797156	1.506442	0.007513
H	-0.957842	1.101135	3.670388
H	0.914950	-0.485311	4.359650
H	-0.241544	-1.815850	4.485241
H	-0.338419	-0.437927	5.590513
H	-1.805680	-2.282854	1.539275
H	-1.696850	-2.570633	3.280525
H	-3.252671	-2.170716	2.543354
H	-6.515690	-2.335542	-3.995737
H	-6.435460	-3.289386	-2.506055
H	-7.692259	-2.057006	-2.701575
H	0.828972	4.494066	0.283127
H	4.366622	1.355962	-2.578278
H	4.472401	1.499201	-0.827411
H	2.501318	-0.078195	-2.545221
H	1.167797	-0.596308	-0.627946
H	3.641896	2.097217	1.016515
H	3.221949	3.815853	1.076120
H	2.248122	2.623266	1.973597
H	3.140454	-1.302694	0.576228
H	4.529649	-3.565509	-0.568504
H	4.179888	-3.287640	1.153151
H	6.183381	-1.834167	-1.254079

B3LYP Energy = -1950.55191235 a.u.

(3*R*,4*S*,5*R*,8*R*,10*S*,19*R*,24*R*)-**3**, Conf H

C	3.940861	1.432361	-2.636727
C	5.425205	1.148856	-2.341816
C	5.654283	1.021636	-0.825152
C	4.710433	-0.014224	-0.186678
C	3.234827	0.361804	-0.442966
C	2.990543	0.428269	-1.962567
C	4.985773	-0.233349	1.278575
C	4.035323	-0.514877	2.175848
C	2.561424	-0.665339	1.863152
C	2.319230	-0.634846	0.301290
C	1.724545	0.302664	2.711159
C	0.859051	-0.211579	3.604910
C	-0.039481	0.515740	4.568172
C	1.958357	1.782308	2.517446
C	0.838222	-0.431333	0.028005
C	6.342351	2.205333	-2.970114
C	-0.072222	-1.575769	0.056492
O	0.374540	0.699546	-0.209552
C	0.245817	-3.011993	0.227830
N	-0.932140	-3.708875	-0.051157
C	-2.081183	-2.839561	-0.138911
C	-1.420428	-1.478159	-0.225875
O	1.301156	-3.553613	0.531118
S	-2.999744	-3.101514	-1.742798
C	-4.039929	-1.601525	-1.586757
C	-3.225941	-0.300340	-1.489492
N	-2.099688	-0.349469	-0.537864
C	-2.994324	-2.995912	1.090696
H	3.069979	1.367181	-0.035803
H	4.899921	-0.972589	-0.702947
C	-4.200238	0.850567	-1.181275
N	-4.126348	1.388697	0.067609
C	-4.913758	2.540188	0.447291
C	-4.189637	3.882029	0.408296
O	-5.014205	1.198816	-2.030385
O	-2.954481	3.810344	-0.131945
O	-4.681779	4.907796	0.822897
H	3.773069	1.435381	-3.721696
H	3.697291	2.445931	-2.283325
H	5.673023	0.174701	-2.793280
H	5.498564	2.000063	-0.345727
H	6.698211	0.740770	-0.628759
H	3.141374	-0.571666	-2.397164
H	1.955331	0.714916	-2.168744
H	6.025422	-0.179666	1.602201
H	4.304531	-0.665198	3.220665
H	2.271475	-1.674643	2.179066

H	2.572817	-1.646962	-0.034948
H	0.787096	-1.299186	3.658769
H	-1.093119	0.267003	4.381364
H	0.056484	1.602439	4.514788
H	0.171016	0.210950	5.601716
H	1.562363	2.111551	1.549959
H	1.484838	2.384198	3.295707
H	3.030453	2.007652	2.524947
H	6.198169	2.262554	-4.055308
H	6.137406	3.199977	-2.554301
H	7.398651	1.978089	-2.784486
H	-1.019979	-4.663590	0.270480
H	-4.655303	-1.546865	-2.486643
H	-4.717205	-1.709110	-0.735511
H	-2.818044	-0.081858	-2.484631
H	-1.434767	0.432742	-0.603552
H	-3.832658	-2.296927	1.079722
H	-3.396185	-4.012780	1.119878
H	-2.411041	-2.815851	1.999556
H	-3.362808	1.089693	0.660087
H	-5.316969	2.421280	1.457374
H	-5.760404	2.611590	-0.241945
H	-2.582325	4.709167	-0.124912

B3LYP Energy = -1950.55163114 a.u.

(3*R*,4*S*,5*R*,8*R*,10*S*,19*R*,24*S*)-**3**, Conf A

C	4.009002	0.157624	-2.892415
C	5.477930	0.033162	-2.447434
C	5.653146	0.584294	-1.021076
C	4.672324	-0.069485	-0.029969
C	3.212528	0.159406	-0.479008
C	3.019424	-0.446738	-1.881867
C	4.896164	0.367801	1.394704
C	3.911717	0.506891	2.288869
C	2.445370	0.243691	2.015797
C	2.254198	-0.409483	0.589934
C	1.607048	1.491722	2.324711
C	0.710001	1.429768	3.326441
C	-0.194098	2.514980	3.844945
C	1.876699	2.733238	1.507462
C	0.787120	-0.342320	0.198033
C	6.431953	0.708303	-3.440473
C	-0.139170	-1.361996	0.674969
O	0.349551	0.579513	-0.518669
C	0.128452	-2.583368	1.466183
N	-1.072541	-3.287747	1.504414
C	-2.182929	-2.570486	0.932724
C	-1.478267	-1.372481	0.333242
O	1.162438	-2.964350	2.002180
S	-2.999670	-3.548095	-0.467345
C	-3.308650	-2.231132	-1.727278
C	-3.384470	-0.831212	-1.111070
N	-2.123441	-0.486281	-0.445158
C	-3.222810	-2.209158	2.009480
H	3.051709	1.241755	-0.558934
H	4.862060	-1.157313	-0.071635
C	-3.703110	0.226829	-2.189505
N	-3.866432	1.512040	-1.762587
C	-3.905839	2.015763	-0.403496
C	-4.399416	3.450187	-0.419887
O	-3.805823	-0.082707	-3.370155
O	-4.491680	3.943041	0.830459
O	-4.668413	4.081133	-1.418054
H	3.877673	-0.317135	-3.873572
H	3.770496	1.223255	-3.030740
H	5.726057	-1.040011	-2.416188
H	5.494418	1.673456	-1.025643
H	6.685655	0.420327	-0.682830
H	3.166152	-1.536180	-1.826962
H	1.996825	-0.277506	-2.231181
H	5.925868	0.551962	1.701985
H	4.143974	0.823239	3.305119
H	2.122303	-0.523968	2.729359
H	2.498830	-1.467751	0.738573
H	0.612230	0.476541	3.848515
H	-0.067647	3.467695	3.325839
H	-0.017494	2.689597	4.914471
H	-1.248868	2.223361	3.749166
H	2.953754	2.918728	1.432537
H	1.495420	2.612273	0.486949
H	1.412621	3.624049	1.936041
H	6.327531	0.281824	-4.444949
H	6.225697	1.783714	-3.512779
H	7.477657	0.589627	-3.133349
H	-1.190480	-4.057098	2.147905
H	-2.526169	-2.253919	-2.487603
H	-4.258256	-2.476820	-2.205641
H	-4.202163	-0.810151	-0.377229
H	-1.474978	0.190427	-0.863739
H	-4.052814	-1.630246	1.600649
H	-3.641031	-3.126913	2.433496
H	-2.742043	-1.634160	2.808119
H	-4.089819	2.185720	-2.490147
H	-2.922458	2.006046	0.081789
H	-4.582090	1.435562	0.236625
H	-4.793489	4.865595	0.766638

B3LYP Energy = -1950.54438319 a.u.

(3*R*,4*S*,5*R*,8*R*,10*S*,19*R*,24*S*)-**3**, Conf B

C	3.782716	2.773292	0.898121
C	5.209818	2.540570	0.369477
C	5.497920	1.033044	0.253538
C	4.426399	0.307277	-0.581308
C	3.025726	0.504560	0.039239
C	2.711826	2.011521	0.099093
C	4.740912	-1.149976	-0.802944
C	3.807795	-2.104539	-0.881700
C	2.315205	-1.873926	-0.771974
C	1.997353	-0.325884	-0.760560

C	1.714893	-2.715970	0.361711
C	0.790355	-3.644592	0.053951
C	0.081128	-4.606033	0.967563
C	2.235644	-2.478261	1.759524
C	0.557358	-0.101812	-0.329248
C	6.258800	3.257294	1.228455
C	-0.513754	-0.169021	-1.314744
O	0.266833	0.138585	0.860498
C	-0.424836	-0.441337	-2.767730
N	-1.707581	-0.257907	-3.275748
C	-2.704493	-0.041828	-2.258686
C	-1.837837	0.111822	-1.025044
O	0.540341	-0.764039	-3.449965
S	-3.653331	1.567797	-2.552041
C	-3.772293	2.255916	-0.842755
C	-3.658989	1.174480	0.235907
N	-2.358381	0.496057	0.153048
C	-3.679516	-1.232264	-2.182444
H	3.060985	0.130833	1.070218
H	4.416606	0.795563	-1.572424
C	-3.834578	1.795861	1.641121
N	-4.026004	0.942753	2.694682
C	-3.908200	-0.501504	2.727952
C	-2.491723	-1.079270	2.811879
O	-3.842497	3.010284	1.796016
O	-1.560320	-0.136154	3.030788
O	-2.267245	-2.261686	2.683543
H	3.557394	3.847800	0.895269
H	3.738103	2.451205	1.949831
H	5.259521	2.962101	-0.647459
H	5.539896	0.587704	1.259324
H	6.487217	0.876552	-0.198269
H	2.660608	2.410760	-0.925329
H	1.732309	2.178758	0.556549
H	5.790372	-1.417566	-0.927794
H	4.105215	-3.139482	-1.046733
H	1.867604	-2.249738	-1.699692
H	2.067376	-0.026769	-1.812644
H	0.499814	-3.723086	-0.995126
H	-0.987093	-4.363946	1.035881
H	0.475391	-4.605906	1.986028
H	0.153811	-5.629502	0.578021
H	3.331312	-2.493593	1.774733
H	1.927111	-1.491657	2.125114
H	1.876628	-3.225338	2.469888
H	6.064219	4.335027	1.277034
H	6.254134	2.871661	2.255850
H	7.268449	3.117040	0.824996
H	-1.937447	-0.597464	-4.198882
H	-2.997470	3.008359	-0.688464
H	-4.745098	2.746488	-0.774834
H	-4.463048	0.441549	0.089683
H	-1.647105	0.651892	0.871726
H	-4.425716	-1.105126	-1.396535
H	-4.216332	-1.321304	-3.131651
H	-3.120247	-2.155880	-2.000013
H	-4.045492	1.428607	3.583627
H	-4.373790	-0.955260	1.849364
H	-4.467044	-0.875477	3.591349
H	-0.673814	-0.508310	2.862739

B3LYP Energy = -1950.54324021 a.u.

(3*R*,4*S*,5*R*,8*R*,10*S*,19*R*,24*S*)-**3**, Conf C

C	4.166140	-2.597118	-1.212359
C	5.621340	-2.257506	-0.840645
C	5.813968	-0.731965	-0.773051
C	4.786559	-0.064523	0.160067
C	3.347102	-0.368695	-0.308570
C	3.134307	-1.894489	-0.313848
C	5.020657	1.414613	0.327892
C	4.036920	2.305001	0.493129
C	2.560806	1.971161	0.544311
C	2.346333	0.405808	0.576492
C	1.791602	2.753923	-0.529293
C	0.879459	3.660309	-0.131027
C	0.030869	4.572185	-0.974991
C	2.142064	2.488067	-1.974592
C	0.890329	0.090933	0.275029
C	6.621614	-2.915753	-1.798911
C	-0.101711	0.146400	1.343435
O	0.517317	-0.208744	-0.875830
C	0.084046	0.400503	2.789855
N	-1.153719	0.167045	3.382978
C	-2.211891	-0.081262	2.437998
C	-1.431786	-0.167508	1.143500
O	1.086028	0.742085	3.406842
S	-3.054744	-1.749620	2.748339
C	-3.216721	-2.417041	1.034281
C	-3.262596	-1.308538	-0.021569
N	-2.014230	-0.534228	-0.013517
C	-3.255046	1.051180	2.462977
H	3.249934	-0.016421	-1.342913
H	4.914448	-0.531193	1.153565
C	-3.490045	-1.901323	-1.427741
N	-3.589317	-1.018456	-2.471085
C	-3.787445	0.415960	-2.421141
C	-5.227230	0.907405	-2.276190
O	-3.557544	-3.109886	-1.604845
O	-6.086692	-0.057003	-1.878551
O	-5.554759	2.056216	-2.471731
H	4.018040	-3.684266	-1.172765
H	3.989511	-2.298886	-2.256978
H	5.807714	-2.655926	0.169658
H	5.720240	-0.304052	-1.782805
H	6.831496	-0.500197	-0.429038
H	3.220181	-2.274669	0.715502
H	2.126275	-2.136239	-0.662611
H	6.056119	1.755672	0.336941
H	4.277065	3.360854	0.612690
H	2.186784	2.332146	1.509970
H	2.526487	0.125347	1.620981

H	0.722157	3.768595	0.943390
H	-1.036840	4.393770	-0.789318
H	0.202710	4.454286	-2.047175
H	0.217173	5.623574	-0.718947
H	3.228623	2.488596	-2.114604
H	1.773929	1.503829	-2.286471
H	1.718004	3.234264	-2.649679
H	6.504695	-4.005673	-1.809423
H	6.475891	-2.555620	-2.825282
H	7.655795	-2.692590	-1.511489
H	-1.325775	0.459254	4.334226
H	-2.390578	-3.096757	0.818167
H	-4.146170	-2.988548	1.013182
H	-4.110246	-0.646202	0.196980
H	-1.321508	-0.651175	-0.762531
H	-4.043462	0.899455	1.723666
H	-3.731624	1.084963	3.447279
H	-2.762628	2.010724	2.272780
H	-3.779974	-1.482490	-3.351810
H	-3.390389	0.868602	-3.333497
H	-3.213358	0.851021	-1.597439
H	-6.966607	0.351644	-1.803560

B3LYP Energy = -1950.54211187 a.u.

(3*R*,4*S*,5*R*,8*R*,10*S*,19*R*,24*S*)-**3**, Conf D

C	3.766373	0.091358	-2.928518
C	5.237322	-0.210220	-2.589807
C	5.554539	0.227602	-1.148427
C	4.566466	-0.379364	-0.134584
C	3.114441	0.021455	-0.476492
C	2.779641	-0.477761	-1.894807
C	4.918051	-0.054421	1.293796
C	4.009484	0.193883	2.242882
C	2.512708	0.182066	2.029783
C	2.180411	-0.498535	0.638037
C	1.885834	1.564870	2.268959
C	2.519629	2.690856	1.898496
C	2.063362	4.116049	2.046409
C	0.558724	1.542278	2.992557
C	0.704703	-0.368813	0.308730
C	6.197214	0.426454	-3.602383
C	-0.235949	-1.383436	0.776726
O	0.268873	0.590740	-0.356442
C	0.017760	-2.624230	1.543283
N	-1.192978	-3.311949	1.573715
C	-2.295393	-2.568656	1.020138
C	-1.576988	-1.367969	0.442359
O	1.048092	-3.028076	2.068770
S	-3.132571	-3.507062	-0.394478
C	-3.410771	-2.164247	-1.632932
C	-3.474751	-0.774503	-0.992669
N	-2.213899	-0.455191	-0.312861
C	-3.326878	-2.213020	2.106884
H	3.051724	1.118149	-0.487717
H	4.636736	-1.476450	-0.246759
C	-3.775969	0.304134	-2.055854
N	-3.915127	1.586876	-1.613232
C	-3.941902	2.077045	-0.249108
C	-4.452898	3.505646	-0.244431
O	-3.887974	0.011387	-3.239949
O	-4.540904	3.982449	1.012557
O	-4.735455	4.145868	-1.232812
H	3.528949	-0.299727	-3.926538
H	3.632374	1.182478	-2.983806
H	5.371332	-1.302956	-2.637306
H	5.517092	1.325534	-1.080664
H	6.580522	-0.066359	-0.886891
H	2.819637	-1.577641	-1.913132
H	1.762003	-0.187791	-2.169816
H	5.976851	-0.045697	1.553461
H	4.336069	0.428769	3.254684
H	2.090739	-0.489897	2.789568
H	2.380934	-1.563941	0.797738
H	3.495578	2.578865	1.429061
H	1.992413	4.600383	1.063546
H	2.791150	4.697627	2.627645
H	1.091949	4.215573	2.536142
H	-0.164909	0.896312	2.483024
H	0.685185	1.129812	4.003151
H	0.108151	2.531816	3.090344
H	5.984709	0.083812	-4.621806
H	6.107296	1.520115	-3.593601
H	7.240321	0.176466	-3.375528
H	-1.319311	-4.091154	2.203647
H	-2.621803	-2.183762	-2.386683
H	-4.358818	-2.388915	-2.124435
H	-4.297073	-0.757134	-0.263677
H	-1.557643	0.225382	-0.713400
H	-4.148096	-1.612759	1.711215
H	-3.759042	-3.132207	2.513435
H	-2.835614	-1.662188	2.916155
H	-4.131654	2.271412	-2.332695
H	-2.951631	2.076731	0.222393
H	-4.600727	1.481444	0.394419
H	-4.852256	4.902659	0.961958

B3LYP Energy = -1950.54172943 a.u.

(3*R*,4*S*,5*R*,8*R*,10*S*,19*R*,24*S*)-**3**, Conf E

C	3.529784	-1.160906	-2.719917
C	4.946512	-1.554122	-2.264320
C	5.339481	-0.764649	-1.003015
C	4.289292	-0.912828	0.114227
C	2.905849	-0.428040	-0.367820
C	2.484906	-1.259009	-1.595134
C	4.700255	-0.243099	1.399604
C	3.844145	0.380687	2.215581
C	2.355382	0.501981	1.981771
C	1.914589	-0.478785	0.817117
C	1.909880	1.965203	1.834783
C	2.668718	2.864122	1.183864

C	2.394495	4.325552	0.955853
C	0.609370	2.299616	2.528833
C	0.478422	-0.224661	0.399574
C	5.974509	-1.381196	-3.389222
C	-0.610614	-0.966490	1.023642
O	0.203421	0.613830	-0.482720
C	-0.550065	-2.000799	2.081696
N	-1.845143	-2.496307	2.212851
C	-2.822236	-1.764363	1.447781
C	-1.931934	-0.856163	0.624891
O	0.402085	-2.390798	2.746202
S	-3.776029	-2.901407	0.276135
C	-3.897903	-1.864899	-1.248749
C	-3.739032	-0.367400	-0.965701
N	-2.429599	-0.090103	-0.360955
C	-3.793872	-1.009382	2.375069
H	2.999230	0.618883	-0.687662
H	4.202988	-1.994959	0.320666
C	-3.897267	0.440564	-2.275608
N	-4.024204	1.800396	-2.178575
C	-3.828769	2.654681	-1.024341
C	-2.383020	3.018202	-0.668362
O	-3.954890	-0.126522	-3.359195
O	-1.498477	2.570639	-1.574224
O	-2.100591	3.632134	0.336080
H	3.225650	-1.788793	-3.567682
H	3.552912	-0.126094	-3.094569
H	4.922639	-2.620717	-1.988495
H	5.454744	0.300223	-1.256179
H	6.317951	-1.106763	-0.638541
H	2.363219	-2.311246	-1.296100
H	1.516562	-0.919064	-1.973210
H	5.754393	-0.297258	1.672137
H	4.211955	0.849790	3.126717
H	1.862620	0.117564	2.885353
H	1.951256	-1.477754	1.264294
H	3.614096	2.512599	0.773053
H	3.152456	4.944914	1.454413
H	1.414211	4.646224	1.315279
H	2.455524	4.567582	-0.113442
H	-0.177077	1.581899	2.271687
H	0.738838	2.238964	3.618662
H	0.231689	3.294140	2.288043
H	5.704041	-1.974997	-4.270150
H	6.039559	-0.331549	-3.702695
H	6.974537	-1.695105	-3.067865
H	-2.092424	-3.049710	3.020938
H	-3.143642	-2.180140	-1.971189
H	-4.883612	-2.057550	-1.676083
H	-4.532727	-0.048786	-0.277537
H	-1.701378	0.390999	-0.895964
H	-4.523511	-0.419888	1.817499
H	-4.350764	-1.731552	2.979514
H	-3.230834	-0.346236	3.040423
H	-4.042986	2.247471	-3.087800
H	-4.261835	2.206136	-0.126667
H	-4.372794	3.590256	-1.185942
H	-0.596039	2.617946	-1.204035

B3LYP Energy = -1950.54169167 a.u.

(3*R*,4*S*,5*R*,8*R*,10*S*,19*S*,24*R*)-**3**, Conf A

C	-3.855761	-2.880582	-0.704757
C	-5.333130	-2.466068	-0.831256
C	-5.608542	-1.205676	0.008191
C	-4.636345	-0.062133	-0.337352
C	-3.174142	-0.505460	-0.112138
C	-2.879215	-1.729418	-0.999806
C	-4.953081	1.217314	0.392800
C	-4.021598	2.085989	0.799331
C	-2.531079	1.926312	0.587783
C	-2.231090	0.693341	-0.354494
C	-1.785180	1.968747	1.928838
C	-0.925366	2.980484	2.151196
C	-0.108850	3.264943	3.382261
C	-2.100597	0.891021	2.939605
C	-0.755505	0.340078	-0.264953
C	-6.279729	-3.615081	-0.462597
C	0.218909	1.078796	-1.060285
O	-0.350274	-0.562093	0.493854
C	0.013321	2.199078	-2.005269
N	1.285526	2.627509	-2.377020
C	2.345677	1.782787	-1.888173
C	1.580207	0.865501	-0.957924
O	-1.025237	2.704816	-2.413977
S	3.621724	2.756423	-0.883116
C	3.888694	1.643494	0.564660
C	3.610835	0.175533	0.231527
N	2.204973	-0.010015	-0.150356
C	3.043290	1.042377	-3.045174
H	-3.077775	-0.823325	0.933482
H	-4.757297	0.137531	-1.417458
C	3.935181	-0.733505	1.436859
N	3.841166	-2.078605	1.232598
C	3.605245	-2.783500	-0.012126
C	3.675226	-4.276947	0.242755
O	4.257612	-0.260800	2.519814
O	3.442147	-4.971003	-0.887946
O	3.911853	-4.787990	1.314943
H	-3.650601	-3.726837	-1.373788
H	-3.677005	-3.244525	0.318504
H	-5.515110	-2.202964	-1.885769
H	-5.518949	-1.449257	1.077762
H	-6.643400	-0.872176	-0.150338
H	-2.961061	-1.437122	-2.057947
H	-1.853471	-2.074463	-0.840271
H	-6.005429	1.441582	0.568391
H	-4.319873	2.995341	1.319713
H	-2.195945	2.806912	0.026890
H	-2.408748	1.070345	-1.368858
H	-0.787699	3.703434	1.345488

H	-0.269842	2.543707	4.186409
H	-0.336314	4.264604	3.775147
H	0.963641	3.262567	3.146528
H	-3.183749	0.757099	3.036178
H	-1.679535	-0.070811	2.624732
H	-1.702197	1.121220	3.929711
H	-6.100115	-4.495825	-1.090353
H	-6.140636	-3.917831	0.582980
H	-7.329137	-3.323661	-0.587822
H	1.400788	3.246174	-3.167030
H	4.932727	1.767656	0.857845
H	3.258740	1.954122	1.399829
H	4.258408	-0.129470	-0.602265
H	1.544395	-0.474197	0.483741
H	3.527705	1.769395	-3.703931
H	3.816334	0.360008	-2.686958
H	2.302891	0.477246	-3.621437
H	4.050897	-2.660321	2.039181
H	4.356341	-2.545039	-0.777661
H	2.624324	-2.555736	-0.444418
H	3.494394	-5.918369	-0.673424

B3LYP Energy = -1950.54425774 a.u.

(3*R*,4*S*,5*R*,8*R*,10*S*,19*S*,24*R*)-**3**, Conf B

C	4.105600	2.409015	-0.634649
C	5.495209	1.773890	-0.821724
C	5.589640	0.451278	-0.039749
C	4.438648	-0.509107	-0.394005
C	3.071272	0.148948	-0.106701
C	2.954428	1.437354	-0.943717
C	4.566046	-1.852290	0.277133
C	3.518532	-2.582241	0.674181
C	2.067116	-2.185514	0.507578
C	1.944777	-0.874560	-0.368379
C	1.345188	-2.181959	1.862100
C	0.328673	-3.044411	2.048852
C	-0.507822	-3.257127	3.280743
C	1.852786	-1.231561	2.921093
C	0.546904	-0.298151	-0.218851
C	6.619970	2.743789	-0.438999
C	-0.543813	-0.819604	-1.030308
O	0.306026	0.614445	0.598302
C	-0.513602	-1.883133	-2.060115
N	-1.831037	-2.056633	-2.477241
C	-2.737266	-1.073232	-1.940684
C	-1.854011	-0.386676	-0.919156
O	0.431429	-2.527436	-2.499320
S	-4.190562	-1.890267	-1.048010
C	-4.393886	-0.800853	0.430315
C	-3.794352	0.593822	0.225473
N	-2.354776	0.503253	-0.046767
C	-3.245264	-0.131076	-3.049280
H	3.053133	0.434411	0.952889
H	4.496444	-0.680037	-1.484029
C	-4.038758	1.470981	1.475927
N	-3.801931	2.815272	1.367637
C	-3.150723	3.539231	0.293196
C	-1.620340	3.474726	0.245916
O	-4.472473	0.982568	2.511433
O	-1.091985	2.923262	1.351515
O	-0.984175	3.877459	-0.701758
H	4.019008	3.303665	-1.264812
H	4.012628	2.754178	0.406678
H	5.606981	1.530720	-1.890631
H	5.570262	0.660094	1.040795
H	6.553108	-0.035063	-0.245880
H	2.970755	1.174392	-2.012229
H	1.998646	1.935731	-0.757910
H	5.573543	-2.245027	0.415734
H	3.681629	-3.548412	1.150218
H	1.590384	-2.974061	-0.087009
H	2.036663	-1.225498	-1.402774
H	0.059056	-3.685257	1.207993
H	-0.223930	-2.611817	4.114854
H	-0.437740	-4.298604	3.621251
H	-1.568722	-3.072828	3.066050
H	2.944248	-1.286200	2.999016
H	1.596342	-0.196862	2.665698
H	1.435094	-1.444980	3.907023
H	6.564270	3.668703	-1.024808
H	6.557085	3.017662	0.621844
H	7.606921	2.298043	-0.609532
H	-2.024666	-2.565119	-3.328445
H	-5.467368	-0.719075	0.610442
H	-3.934281	-1.268999	1.301868
H	-4.295170	1.075931	-0.623880
H	-1.673235	0.810476	0.650427
H	-3.813294	-0.710478	-3.783107
H	-3.908116	0.644347	-2.661786
H	-2.393572	0.342692	-3.548829
H	-3.905865	3.294777	2.254196
H	-3.434815	4.593678	0.364582
H	-3.503742	3.197803	-0.683100
H	-0.158766	2.690436	1.181452

B3LYP Energy = -1950.54373149 a.u.

(3*R*,4*S*,5*R*,8*R*,10*S*,19*S*,24*R*)-**3**, Conf C

C	3.996137	3.085057	-0.296725
C	5.461618	2.693774	-0.559665
C	5.765600	1.315891	0.055369
C	4.767014	0.243298	-0.418317
C	3.321554	0.646445	-0.055389
C	2.995957	1.996663	-0.722005
C	5.105858	-1.136430	0.084769
C	4.186320	-2.057024	0.392924
C	2.688842	-1.864896	0.275660
C	2.359177	-0.496846	-0.444146
C	2.000052	-2.119711	1.623543
C	1.134737	-3.146641	1.717161
C	0.362734	-3.616376	2.919820

C	2.367907	-1.217536	2.778145
C	0.891258	-0.159946	-0.239972
C	6.436223	3.768235	-0.062081
C	-0.117977	-0.760165	-1.106406
O	0.521492	0.605982	0.670782
C	0.047828	-1.712370	-2.228139
N	-1.238328	-2.077708	-2.614434
C	-2.278365	-1.348995	-1.936269
C	-1.472959	-0.577465	-0.912597
O	1.068317	-2.141428	-2.753344
S	-3.465690	-2.506300	-1.019842
C	-3.689419	-1.623836	0.587129
C	-3.448997	-0.116225	0.463317
N	-2.065959	0.156104	0.048326
C	-3.060355	-0.454990	-2.916240
H	3.276734	0.793202	1.031031
H	4.833460	0.217175	-1.520958
C	-3.733306	0.594490	1.802553
N	-3.602008	1.958452	1.828147
C	-3.492207	2.876579	0.712078
C	-4.802019	3.332325	0.070051
O	-4.043662	-0.035781	2.804044
O	-5.830535	2.484133	0.296559
O	-4.897072	4.332847	-0.604288
H	3.766588	4.026572	-0.813096
H	3.869942	3.283562	0.778582
H	5.589339	2.600076	-1.650230
H	5.730614	1.386605	1.153214
H	6.788256	1.009702	-0.204978
H	3.025021	1.875518	-1.815822
H	1.982165	2.313983	-0.461183
H	6.163084	-1.386919	0.174485
H	4.500327	-3.036943	0.750571
H	2.323276	-2.644485	-0.403501
H	2.492632	-0.707123	-1.512075
H	0.954419	-3.730020	0.812899
H	0.566527	-3.033984	3.820964
H	0.589118	-4.667987	3.139471
H	-0.718074	-3.566145	2.731748
H	1.937759	-0.218953	2.639292
H	2.014159	-1.603015	3.736492
H	3.454852	-1.098095	2.844839
H	6.235729	4.737054	-0.534407
H	6.350214	3.903131	1.023566
H	7.475403	3.497149	-0.282350
H	-1.390395	-2.583280	-3.475215
H	-4.718127	-1.810534	0.899900
H	-3.019282	-2.042721	1.339984
H	-4.145617	0.293645	-0.279140
H	-1.381032	0.524778	0.718152
H	-3.562640	-1.081996	-3.658928
H	-3.828967	0.132450	-2.410762
H	-2.369105	0.221187	-3.430516
H	-3.842055	2.352821	2.730528
H	-2.878831	2.437173	-0.081082
H	-2.967478	3.779071	1.034584
H	-6.610478	2.844339	-0.160286

B3LYP Energy = -1950.54203077 a.u.

(3*R*,4*S*,5*R*,8*R*,10*S*,19*S*,24*R*)-**3**, Conf D

C	3.592841	2.772035	-1.056578
C	5.059686	2.392281	-1.330595
C	5.469636	1.187667	-0.464674
C	4.500220	0.003160	-0.636739
C	3.059679	0.416976	-0.266722
C	2.626599	1.581232	-1.177922
C	4.940000	-1.228649	0.111199
C	4.093843	-2.070799	0.713375
C	2.589781	-1.920322	0.732296
C	2.148620	-0.828974	-0.328453
C	2.047810	-1.729310	2.157811
C	2.708491	-0.984731	3.060898
C	2.327823	-0.683125	4.483954
C	0.771598	-2.482906	2.453825
C	0.672736	-0.500504	-0.194959
C	6.002451	3.585493	-1.132211
C	-0.317016	-1.272939	-0.941382
O	0.278589	0.421886	0.544368
C	-0.131656	-2.442448	-1.829840
N	-1.412026	-2.866133	-2.179254
C	-2.457524	-1.983289	-1.728254
C	-1.675065	-1.035347	-0.845154
O	0.897235	-2.987479	-2.210695
S	-3.736599	-2.891261	-0.667408
C	-4.018487	-1.686127	0.704517
C	-3.697124	-0.245619	0.297346
N	-2.283525	-0.115632	-0.075595
C	-3.150969	-1.292933	-2.918009
H	3.060785	0.786681	0.767632
H	4.501250	-0.249274	-1.712703
C	-4.012650	0.734704	1.448199
N	-3.879389	2.063839	1.173928
C	-3.602046	2.694584	-0.101792
C	-3.632915	4.200931	0.072315
O	-4.362498	0.329027	2.549627
O	-3.361250	4.826408	-1.089320
O	-3.872308	4.775074	1.111404
H	3.285093	3.574594	-1.740211
H	3.519242	3.187062	-0.039777
H	5.129926	2.076991	-2.384198
H	5.494844	1.486572	0.594258
H	6.489614	0.872230	-0.724406
H	2.598333	1.233834	-2.222150
H	1.615690	1.908900	-0.919824
H	6.009501	-1.438706	0.134812
H	4.482261	-2.939049	1.243226
H	2.173653	-2.868280	0.364537
H	2.281183	-1.315771	-1.301384
H	3.646117	-0.532558	2.741322
H	1.386733	-1.144231	4.791372
H	2.231626	0.400264	4.633804

H	3.109402	-1.024793	5.175585
H	0.359771	-2.258382	3.439209
H	-0.004696	-2.265918	1.711794
H	0.950234	-3.565848	2.402477
H	5.724607	4.424612	-1.780826
H	5.970321	3.942064	-0.094873
H	7.040536	3.316048	-1.359384
H	-1.540646	-3.527564	-2.931538
H	-5.072700	-1.768850	0.974882
H	-3.419223	-1.962846	1.573415
H	-4.326946	0.030775	-0.559540
H	-1.615576	0.367146	0.536839
H	-3.642610	-2.047091	-3.539758
H	-3.917750	-0.587215	-2.593310
H	-2.406647	-0.763253	-3.522216
H	-4.083107	2.693406	1.945376
H	-4.346787	2.436772	-0.867223
H	-2.621085	2.416718	-0.503543
H	-3.389104	5.785044	-0.926478

B3LYP Energy = -1950.54166085 a.u.

(3*R*,4*S*,5*R*,8*R*,10*S*,19*S*,24*R*)-**3**, Conf E

C	3.813827	2.236254	-1.299725
C	5.209942	1.622921	-1.512398
C	5.441217	0.460665	-0.529652
C	4.308004	-0.580750	-0.596028
C	2.946201	0.076248	-0.285510
C	2.686776	1.189464	-1.317848
C	4.562110	-1.784609	0.272965
C	3.601407	-2.432057	0.940561
C	2.135769	-2.061567	0.926796
C	1.857799	-1.019229	-0.234217
C	1.628679	-1.668148	2.323282
C	2.398996	-0.964518	3.170505
C	2.074200	-0.494808	4.561369
C	0.250813	-2.187413	2.663257
C	0.453491	-0.454490	-0.135980
C	6.317150	2.679703	-1.414501
C	-0.639178	-1.076789	-0.872756
O	0.207909	0.545322	0.569734
C	-0.611222	-2.261274	-1.762598
N	-1.927692	-2.476584	-2.160382
C	-2.834533	-1.437680	-1.744315
C	-1.949320	-0.630806	-0.816298
O	0.330567	-2.962026	-2.112477
S	-4.276801	-2.144912	-0.749332
C	-4.491610	-0.872487	0.574824
C	-3.887391	0.485295	0.201563
N	-2.448091	0.358448	-0.057008
C	-3.351299	-0.640924	-2.957841
H	3.006769	0.543792	0.707016
H	4.267899	-0.934963	-1.641912
C	-4.132236	1.508672	1.335670
N	-3.875006	2.827388	1.072354
C	-3.199461	3.412256	-0.069742
C	-1.668458	3.337767	-0.077702
O	-4.585937	1.152238	2.415591
O	-1.166124	2.909475	1.092351
O	-1.011873	3.629104	-1.051930
H	3.624639	3.001659	-2.063444
H	3.800582	2.757713	-0.330168
H	5.234705	1.200973	-2.530038
H	5.513871	0.854190	0.495721
H	6.403147	-0.024128	-0.746351
H	2.615869	0.742413	-2.321051
H	1.730978	1.685462	-1.125905
H	5.588776	-2.146589	0.330898
H	3.858190	-3.293220	1.555288
H	1.584070	-2.968130	0.641111
H	1.906723	-1.606418	-1.157153
H	3.395706	-0.693255	2.825561
H	2.788644	-0.907079	5.286364
H	1.069969	-0.768744	4.892813
H	2.160292	0.597773	4.628514
H	-0.131956	-1.803117	3.610486
H	-0.477582	-1.935257	1.884599
H	0.264426	-3.284292	2.726832
H	6.162452	3.486774	-2.140097
H	6.340839	3.131219	-0.414534
H	7.304221	2.242410	-1.605416
H	-2.126777	-3.097215	-2.932060
H	-5.566216	-0.766865	0.734229
H	-4.040467	-1.227798	1.502289
H	-4.386966	0.862490	-0.700049
H	-1.763667	0.750216	0.593936
H	-3.908042	-1.312085	-3.618604
H	-4.027865	0.164668	-2.667868
H	-2.504568	-0.218290	-3.508985
H	-3.983529	3.409259	1.894813
H	-3.480155	4.467941	-0.135679
H	-3.533994	2.951902	-1.002781
H	-0.233016	2.647339	0.967958

B3LYP Energy = -1950.54138283 a.u.

(3*R*,4*S*,5*R*,8*R*,10*S*,19*S*,24*S*)-**3**, Conf A

C	3.635345	3.202889	-0.476539
C	5.132094	2.947454	-0.731078
C	5.579981	1.648731	-0.036495
C	4.693720	0.451973	-0.429387
C	3.216465	0.730167	-0.074325
C	2.750677	1.993487	-0.822847
C	5.174596	-0.851304	0.154343
C	4.356218	-1.838520	0.532964
C	2.845884	-1.805261	0.429772
C	2.372142	-0.528639	-0.372394
C	2.202546	-2.035479	1.804500
C	1.447636	-3.135738	1.982156
C	0.743257	-3.600117	3.227458
C	2.494364	-1.026490	2.890416

C	0.880098	-0.324835	-0.165046
C	5.993319	4.144655	-0.310228
C	-0.078632	-1.075563	-0.975208
O	0.450507	0.463511	0.697498
C	0.180192	-2.097515	-2.014803
N	-1.061189	-2.655319	-2.332950
C	-2.161878	-1.903791	-1.778386
C	-1.444827	-1.013902	-0.782823
O	1.235276	-2.453974	-2.523776
S	-3.305056	-3.008730	-0.797863
C	-4.223836	-1.636882	-0.003955
C	-3.328352	-0.702070	0.825958
N	-2.096645	-0.271813	0.141354
C	-2.906434	-1.113539	-2.869932
H	3.164001	0.943385	1.000610
H	4.755733	0.362524	-1.529035
C	-4.185358	0.488770	1.290482
N	-3.919748	1.689617	0.723066
C	-4.690681	2.865545	1.060935
C	-4.310458	4.001815	0.137922
O	-5.080647	0.312654	2.114194
O	-5.003782	5.120838	0.425088
O	-3.486338	3.930264	-0.749386
H	3.307588	4.081961	-1.046936
H	3.494126	3.453284	0.585982
H	5.263425	2.800253	-1.815287
H	5.544954	1.784235	1.055222
H	6.627016	1.432997	-0.290617
H	2.788616	1.806055	-1.906953
H	1.710845	2.222493	-0.573073
H	6.252570	-0.988351	0.242726
H	4.771474	-2.757479	0.945051
H	2.553354	-2.662003	-0.189010
H	2.515300	-0.794890	-1.426325
H	1.314855	-3.792921	1.121429
H	0.902142	-2.943838	4.086002
H	1.076460	-4.607898	3.508111
H	-0.339384	-3.668973	3.057314
H	1.966156	-0.085925	2.695783
H	2.194192	-1.380466	3.878828
H	3.565007	-0.797239	2.927170
H	5.689808	5.057397	-0.836200
H	5.900575	4.334732	0.766568
H	7.053605	3.969768	-0.527181
H	-1.157060	-3.156667	-3.206033
H	-4.781587	-1.080022	-0.761708
H	-4.953201	-2.090635	0.669548
H	-3.041670	-1.234851	1.741810
H	-1.406894	0.209870	0.732170
H	-3.353088	-1.810246	-3.585204
H	-3.700172	-0.484694	-2.462442
H	-2.196579	-0.466714	-3.395574
H	-3.184014	1.784342	0.031753
H	-5.765673	2.668457	0.973795
H	-4.521276	3.177496	2.099461
H	-4.715811	5.815457	-0.191925

B3LYP Energy = -1950.55498897 a.u.

(3*R*,4*S*,5*R*,8*R*,10*S*,19*S*,24*S*)-**3**, Conf B

C	3.810593	3.320021	-0.360150
C	5.299106	3.058030	-0.653756
C	5.743086	1.726472	-0.021818
C	4.836444	0.557771	-0.450490
C	3.368268	0.836147	-0.058920
C	2.904631	2.137068	-0.741201
C	5.311033	-0.774642	0.068978
C	4.487142	-1.769401	0.414271
C	2.976238	-1.716569	0.331856
C	2.505239	-0.397891	-0.402143
C	2.347215	-2.007664	1.701569
C	1.581944	-3.107086	1.834285
C	0.886431	-3.624489	3.063617
C	2.663242	-1.056186	2.831643
C	1.018997	-0.192874	-0.159975
C	6.182730	4.227043	-0.201495
C	0.042105	-0.895814	-0.989804
O	0.609348	0.550876	0.751239
C	0.276123	-1.836583	-2.109680
N	-0.969736	-2.380115	-2.430256
C	-2.059282	-1.687025	-1.783919
C	-1.317486	-0.864560	-0.749921
O	1.317973	-2.142677	-2.675890
S	-3.150746	-2.878205	-0.848697
C	-4.062809	-1.583728	0.072480
C	-3.154651	-0.688986	0.935063
N	-1.944559	-0.201973	0.249232
C	-2.850182	-0.828390	-2.787882
H	3.335565	1.000425	1.025373
H	4.879562	0.514885	-1.553749
C	-4.022189	0.461961	1.473777
N	-3.737756	1.704961	0.995388
C	-4.602027	2.829176	1.254572
C	-5.481961	3.165432	0.057863
O	-4.921986	0.223224	2.272907
O	-6.282499	4.217975	0.331958
O	-5.472074	2.591273	-1.008811
H	3.483675	4.226570	-0.886435
H	3.692546	3.526468	0.714561
H	5.408243	2.953884	-1.745397
H	5.727552	1.815920	1.075095
H	6.783144	1.510169	-0.302549
H	2.918461	1.997593	-1.833050
H	1.872536	2.365853	-0.459971
H	6.388496	-0.926112	0.137403
H	4.897698	-2.709721	0.780272
H	2.667143	-2.539674	-0.323790
H	2.628840	-0.615342	-1.469490
H	1.431535	-3.719567	0.943943
H	-0.197442	-3.685025	2.898232
H	1.052459	-3.006245	3.948616
H	1.220161	-4.643887	3.297876

H	2.151589	-0.098300	2.683073
H	2.360878	-1.449875	3.804197
H	3.738208	-0.848711	2.873261
H	5.881825	5.163502	-0.685621
H	6.111733	4.375645	0.883402
H	7.236598	4.048065	-0.444639
H	-1.090403	-2.818674	-3.333492
H	-4.660105	-0.986441	-0.621102
H	-4.757647	-2.098249	0.738538
H	-2.839072	-1.268320	1.812172
H	-1.240881	0.242603	0.852343
H	-3.323478	-1.478219	-3.529638
H	-3.627463	-0.229142	-2.310612
H	-2.162138	-0.146719	-3.298596
H	-3.052545	1.771055	0.253605
H	-5.242280	2.581625	2.106208
H	-4.023806	3.720503	1.522730
H	-6.820879	4.395851	-0.458314

B3LYP Energy = -1950.55476860 a.u.

(3*R*,4*S*,5*R*,8*R*,10*S*,19*S*,24*S*)-**3**, Conf C

C	3.853221	2.839937	-1.199367
C	5.348142	2.471751	-1.204006
C	5.639177	1.404983	-0.133437
C	4.722753	0.176466	-0.284238
C	3.237663	0.589693	-0.189544
C	2.930869	1.613606	-1.299326
C	5.058801	-0.928732	0.683490
C	4.145637	-1.748384	1.214382
C	2.661177	-1.704899	0.920302
C	2.349890	-0.672739	-0.236870
C	1.851010	-1.547589	2.214904
C	1.008112	-2.536260	2.567167
C	0.127697	-2.630638	3.783094
C	2.078222	-0.297733	3.032122
C	0.860151	-0.370522	-0.249312
C	6.239505	3.708163	-1.034526
C	-0.067928	-1.294629	-0.901872
O	0.402030	0.642674	0.308623
C	0.225267	-2.564669	-1.604404
N	-1.009750	-3.168444	-1.852006
C	-2.119686	-2.282158	-1.597425
C	-1.440293	-1.160208	-0.836521
O	1.299429	-3.060660	-1.919856
S	-3.347257	-3.065752	-0.428893
C	-4.281828	-1.524292	-0.112075
C	-3.421419	-0.393791	0.479176
N	-2.129268	-0.182758	-0.200126
C	-2.774875	-1.815182	-2.909926
H	3.089982	1.091010	0.775230
H	4.889453	-0.216395	-1.303572
C	-4.285981	0.878831	0.513348
N	-3.953324	1.866780	-0.364216
C	-4.624113	3.143847	-0.338773
C	-3.881204	4.181526	0.494935
O	-5.253483	0.931800	1.265671
O	-4.556310	5.353679	0.505097
O	-2.829146	4.008025	1.064957
H	3.641073	3.535465	-2.022193
H	3.624470	3.382748	-0.269636
H	5.576427	2.022244	-2.184027
H	5.501907	1.841645	0.867508
H	6.690528	1.091717	-0.195829
H	3.062756	1.131511	-2.280265
H	1.888402	1.938757	-1.237702
H	6.110509	-1.066305	0.935537
H	4.456888	-2.529113	1.907456
H	2.393000	-2.687171	0.513381
H	2.574389	-1.216095	-1.162508
H	0.935504	-3.392456	1.894845
H	-0.925726	-2.730346	3.489315
H	0.206943	-1.764837	4.444381
H	0.370369	-3.526223	4.370185
H	1.645582	0.575703	2.530719
H	1.634824	-0.365414	4.027574
H	3.149953	-0.105470	3.153530
H	6.049801	4.446437	-1.822469
H	6.053772	4.196705	-0.069526
H	7.302322	3.442113	-1.073503
H	-1.065591	-3.890632	-2.557704
H	-4.781140	-1.201368	-1.029279
H	-5.060669	-1.767262	0.612876
H	-3.215447	-0.637887	1.529030
H	-1.466096	0.427845	0.295526
H	-3.206623	-2.675106	-3.430097
H	-3.566192	-1.081808	-2.743428
H	-2.013636	-1.356255	-3.549321
H	-3.067149	1.784531	-0.845327
H	-4.748276	3.536724	-1.353492
H	-5.621114	3.004311	0.088079
H	-4.048643	5.981256	1.047682

B3LYP Energy = -1950.55278272 a.u.

(3*R*,4*S*,5*R*,8*R*,10*S*,19*S*,24*S*)-**3**, Conf D

C	3.737619	2.808335	-1.749961
C	5.257912	2.596166	-1.635455
C	5.595699	1.839367	-0.338979
C	4.802212	0.524187	-0.220554
C	3.282992	0.798062	-0.261675
C	2.934754	1.506935	-1.584276
C	5.189651	-0.290325	0.986434
C	4.330062	-1.056716	1.665867
C	2.864617	-1.232420	1.327723
C	2.516735	-0.524853	-0.041628
C	1.981600	-0.877988	2.531961
C	1.219109	-1.845845	3.073891
C	0.296553	-1.761708	4.259278
C	2.052839	0.536629	3.057038
C	1.007951	-0.381349	-0.174592
C	6.027122	3.919065	-1.739504

C	0.215561	-1.488641	-0.690497
O	0.429978	0.670877	0.164924
C	0.648010	-2.843129	-1.089272
N	-0.521518	-3.585078	-1.302917
C	-1.698718	-2.749866	-1.345869
C	-1.168625	-1.456061	-0.765646
O	1.774216	-3.310360	-1.209509
S	-2.999375	-3.360633	-0.153302
C	-4.064493	-1.877456	-0.307217
C	-3.366756	-0.550868	0.043513
N	-1.962204	-0.449500	-0.376554
C	-2.235356	-2.595071	-2.780396
H	3.043306	1.486999	0.558151
H	5.051330	-0.074351	-1.115376
C	-4.146024	0.611789	-0.616417
N	-4.382849	1.678099	0.188148
C	-5.060894	2.858828	-0.303769
C	-5.229171	3.844704	0.831128
O	-4.504974	0.568839	-1.789551
O	-5.852963	4.965156	0.423596
O	-4.851741	3.658375	1.969429
H	3.500326	3.273933	-2.715709
H	3.420886	3.523378	-0.975364
H	5.568019	1.956680	-2.477566
H	5.373601	2.478674	0.529041
H	6.673045	1.626210	-0.302342
H	3.152636	0.830168	-2.424612
H	1.865313	1.733079	-1.623528
H	6.235351	-0.259106	1.293312
H	4.676552	-1.623706	2.529236
H	2.710169	-2.302927	1.145974
H	2.839201	-1.236251	-0.810836
H	1.256029	-2.830346	2.605255
H	0.583007	-2.492525	5.026996
H	-0.732503	-2.009080	3.965874
H	0.280436	-0.774619	4.726945
H	1.583106	1.231799	2.351791
H	1.552408	0.648396	4.021162
H	3.095079	0.851746	3.180307
H	5.806710	4.435329	-2.681265
H	5.756484	4.595017	-0.918394
H	7.110311	3.756145	-1.692797
H	-0.459428	-4.417850	-1.873795
H	-4.480316	-1.820427	-1.313676
H	-4.895921	-2.026330	0.386751
H	-3.376867	-0.427845	1.135626
H	-1.406108	0.323504	0.007424
H	-2.594138	-3.562728	-3.143645
H	-3.048377	-1.870243	-2.847298
H	-1.422659	-2.249278	-3.427507
H	-4.090202	1.694718	1.158110
H	-4.498615	3.335163	-1.116377
H	-6.045173	2.607990	-0.716919
H	-5.928904	5.560447	1.189258

B3LYP Energy = -1950.55218964 a.u.

(3*R*,4*S*,5*R*,8*R*,10*S*,19*S*,24*S*)-**3**, Conf E

C	3.430440	3.109783	-0.765663
C	4.901131	2.880163	-1.158517
C	5.450638	1.623097	-0.460601
C	4.562539	0.391575	-0.719247
C	3.121030	0.646125	-0.226261
C	2.547673	1.866837	-0.970996
C	5.136957	-0.878540	-0.148609
C	4.394191	-1.849484	0.392636
C	2.887670	-1.820869	0.513055
C	2.301515	-0.653716	-0.384460
C	2.429234	-1.836813	1.980495
C	3.095815	-1.151271	2.924922
C	2.796637	-1.046898	4.394700
C	1.234439	-2.719068	2.260432
C	0.817114	-0.464688	-0.131610
C	5.765152	4.114705	-0.872813
C	-0.159307	-1.234897	-0.905795
O	0.404872	0.334804	0.728307
C	0.077209	-2.291499	-1.916707
N	-1.174377	-2.841103	-2.207354
C	-2.260422	-2.060817	-1.664428
C	-1.522921	-1.152036	-0.701734
O	1.123107	-2.679416	-2.421091
S	-3.405594	-3.123496	-0.639635
C	-4.301239	-1.718817	0.122081
C	-3.388270	-0.768207	0.914596
N	-2.155416	-0.376956	0.208807
C	-3.008074	-1.294210	-2.770563
H	3.160360	0.896496	0.842568
H	4.515393	0.264943	-1.816145
C	-4.227902	0.448340	1.343141
N	-3.954141	1.624243	0.729384
C	-4.710724	2.820210	1.026808
C	-4.337806	3.910705	0.047232
O	-5.118329	0.312058	2.179509
O	-5.020242	5.046154	0.292749
O	-3.526961	3.793360	-0.847426
H	3.022789	3.955474	-1.335133
H	3.389909	3.402445	0.294593
H	4.929915	2.690617	-2.243651
H	5.517477	1.803053	0.623189
H	6.473650	1.421798	-0.807468
H	2.477619	1.637002	-2.045294
H	1.534336	2.084036	-0.622409
H	6.218134	-1.004569	-0.209431
H	4.877229	-2.740087	0.791312
H	2.517644	-2.750643	0.059705
H	2.407641	-1.013113	-1.414225
H	3.975310	-0.594365	2.605847
H	1.910848	-1.607380	4.701846
H	2.643277	0.001716	4.681595
H	3.644900	-1.411080	4.989534
H	1.491318	-3.772573	2.082851
H	0.869588	-2.633692	3.285716

H	0.398306	-2.492144	1.590005
H	5.385992	4.996942	-1.401857
H	5.772684	4.347668	0.199598
H	6.803713	3.956882	-1.186475
H	-1.285153	-3.372525	-3.060558
H	-4.861652	-1.179518	-0.646247
H	-5.028024	-2.144828	0.816216
H	-3.102102	-1.273458	1.846006
H	-1.453206	0.112058	0.779246
H	-3.470527	-2.006538	-3.459938
H	-3.790259	-0.644558	-2.373304
H	-2.296846	-0.671550	-3.322822
H	-3.222340	1.685051	0.030133
H	-5.788225	2.627243	0.966428
H	-4.521680	3.177811	2.047129
H	-4.736853	5.710083	-0.359195

B3LYP Energy = -1950.55217393 a.u.

(3*R*,4*S*,5*R*,8*R*,10*S*,19*S*,24*S*)-**3**, Conf F

C	3.790703	3.320587	-0.435644
C	5.283333	3.064248	-0.712559
C	5.732884	1.749193	-0.050647
C	4.837430	0.564728	-0.459522
C	3.365161	0.841080	-0.082358
C	2.896853	2.122550	-0.797771
C	5.319004	-0.752356	0.091488
C	4.500742	-1.744624	0.456617
C	2.989802	-1.704603	0.367951
C	2.512093	-0.407161	-0.398865
C	2.357662	-1.966690	1.742094
C	1.600674	-3.068745	1.898713
C	0.905759	-3.562701	3.137910
C	2.662721	-0.985933	2.849932
C	1.023910	-0.204946	-0.167048
C	6.154354	4.248826	-0.276562
C	0.052235	-0.941854	-0.974644
O	0.606574	0.569175	0.714726
C	0.293936	-1.934021	-2.047609
N	-0.951251	-2.487443	-2.355553
C	-2.043779	-1.758077	-1.756499
C	-1.309333	-0.893334	-0.751709
O	1.340081	-2.270005	-2.587606
S	-3.160838	-2.895339	-0.784784
C	-4.069881	-1.551440	0.066323
C	-3.162267	-0.635056	0.904082
N	-1.944151	-0.180803	0.208636
C	-2.813903	-0.940238	-2.809570
H	3.325454	1.030963	0.997479
H	4.887329	0.498457	-1.561375
C	-4.020485	0.538917	1.407326
N	-3.722612	1.767619	0.903421
C	-4.522531	2.930227	1.210982
C	-5.578787	3.309360	0.178818
O	-4.927207	0.328877	2.206640
O	-5.676547	2.435023	-0.850007
O	-6.263527	4.303102	0.271169
H	3.460765	4.213172	-0.983420
H	3.662663	3.548718	0.633500
H	5.401781	2.939372	-1.801050
H	5.710216	1.861287	1.044028
H	6.776094	1.534685	-0.320714
H	2.921096	1.958646	-1.886097
H	1.860938	2.350048	-0.530162
H	6.397201	-0.894813	0.166908
H	4.916384	-2.673585	0.845123
H	2.689110	-2.545094	-0.268961
H	2.640544	-0.649740	-1.460408
H	1.458887	-3.703839	1.022920
H	1.066895	-2.924394	4.009452
H	1.244945	-4.574780	3.394848
H	-0.177479	-3.633002	2.972308
H	2.143716	-0.035835	2.678370
H	2.361578	-1.359167	3.830861
H	3.735766	-0.768369	2.888815
H	5.849432	5.173448	-0.780458
H	6.074308	4.417313	0.804787
H	7.211326	4.073832	-0.508759
H	-1.063579	-2.969658	-3.237399
H	-4.647874	-0.975739	-0.661171
H	-4.782149	-2.028085	0.742118
H	-2.856205	-1.188423	1.801231
H	-1.241530	0.279069	0.802020
H	-3.277951	-1.618789	-3.531160
H	-3.596984	-0.321509	-2.367522
H	-2.115328	-0.282170	-3.336689
H	-3.001353	1.824884	0.196715
H	-5.050280	2.742439	2.150682
H	-3.886694	3.808407	1.357964
H	-6.384247	2.765084	-1.430511

B3LYP Energy = -1950.55201040 a.u.

(3*R*,4*S*,5*R*,8*R*,10*S*,19*S*,24*S*)-**3**, Conf G

C	3.848141	3.279294	-0.622648
C	5.349345	2.998962	-0.818123
C	5.765513	1.741483	-0.033908
C	4.881003	0.529329	-0.380916
C	3.396719	0.837392	-0.086449
C	2.963609	2.057188	-0.921679
C	5.333632	-0.740630	0.292834
C	4.496352	-1.697659	0.706114
C	2.989795	-1.660622	0.556707
C	2.552632	-0.431858	-0.336203
C	2.302565	-1.800836	1.922527
C	1.533368	-2.883168	2.144498
C	0.790946	-3.266329	3.395395
C	2.572901	-0.730685	2.954340
C	1.057436	-0.210948	-0.191356
C	6.208336	4.213202	-0.444322
C	0.120860	-1.006882	-0.987623
O	0.600365	0.628054	0.607166

C	0.408915	-2.084175	-1.965000
N	-0.824065	-2.652537	-2.291312
C	-1.939146	-1.878090	-1.802872
C	-1.247813	-0.932855	-0.842689
O	1.478258	-2.467171	-2.419590
S	-3.090670	-2.934180	-0.776969
C	-4.037615	-1.529358	-0.084550
C	-3.163730	-0.537560	0.705472
N	-1.922988	-0.133395	0.022376
C	-2.670507	-1.160842	-2.951997
H	3.315256	1.112676	0.972356
H	4.974460	0.373529	-1.470945
C	-4.028316	0.673504	1.073724
N	-3.720157	1.857653	0.508691
C	-4.408630	3.103299	0.850775
C	-5.827827	3.223918	0.262789
O	-4.989524	0.525774	1.843412
O	-6.691457	2.263253	0.622842
O	-6.145780	4.142807	-0.456127
H	3.544284	4.124595	-1.253994
H	3.678867	3.594241	0.418303
H	5.510403	2.786207	-1.887239
H	5.700029	1.942877	1.046119
H	6.817377	1.503483	-0.243506
H	3.031401	1.804868	-1.991042
H	1.918737	2.307662	-0.716383
H	6.407289	-0.879861	0.420170
H	4.891313	-2.593365	1.183855
H	2.708261	-2.551445	-0.017424
H	2.728549	-0.762611	-1.366665
H	1.423022	-3.593379	1.323567
H	0.905493	-2.543852	4.206447
H	1.131331	-4.242763	3.764448
H	-0.282870	-3.371227	3.191733
H	2.062881	0.202837	2.689500
H	2.240014	-1.022090	3.952557
H	3.644547	-0.510411	3.009818
H	5.928004	5.095043	-1.032041
H	6.086863	4.467489	0.616208
H	7.272632	4.018100	-0.619997
H	-0.897896	-3.209851	-3.131953
H	-4.589094	-1.024029	-0.881785
H	-4.774133	-1.951437	0.601607
H	-2.896041	-1.011782	1.659367
H	-1.237519	0.360348	0.610528
H	-3.094956	-1.902986	-3.634042
H	-3.481145	-0.520317	-2.599178
H	-1.957423	-0.537862	-3.501620
H	-2.946644	1.882604	-0.143913
H	-4.470645	3.184124	1.942757
H	-3.821112	3.936213	0.467643
H	-6.256133	1.605045	1.217316

B3LYP Energy = -1950.55200485 a.u.

(3*R*,4*S*,5*R*,8*R*,10*S*,19*S*,24*S*)-**3**, Conf H

C	3.486095	3.284271	-0.549760
C	4.938437	3.115767	-1.031202
C	5.537158	1.811310	-0.475455
C	4.652703	0.592285	-0.799083
C	3.234567	0.777775	-0.218310
C	2.610594	2.049515	-0.824692
C	5.270462	-0.712410	-0.367699
C	4.567456	-1.736991	0.125993
C	3.068029	-1.744558	0.319652
C	2.424745	-0.517360	-0.449626
C	2.678319	-1.888861	1.799398
C	3.378923	-1.274614	2.768002
C	3.145020	-1.299613	4.253066
C	1.506931	-2.809023	2.056267
C	0.951279	-0.373364	-0.116161
C	5.799451	4.334658	-0.677312
C	-0.050116	-1.088044	-0.909409
O	0.569145	0.347048	0.824751
C	0.151189	-2.056453	-2.012051
N	-1.108177	-2.589504	-2.298420
C	-2.176124	-1.859434	-1.656124
C	-1.405691	-1.031212	-0.648361
O	1.178917	-2.392847	-2.586290
S	-3.280220	-3.005058	-0.679108
C	-4.159967	-1.667628	0.210723
C	-3.229629	-0.767735	1.045100
N	-2.002975	-0.341138	0.349712
C	-2.960651	-1.007648	-2.671134
H	3.322521	0.934356	0.865323
H	4.554514	0.560366	-1.899392
C	-4.068366	0.425026	1.537098
N	-3.746279	1.642124	1.018013
C	-4.585103	2.796109	1.224226
C	-5.451250	3.100632	0.008890
O	-4.980802	0.238667	2.335739
O	-6.229094	4.181534	0.232770
O	-5.448837	2.481155	-1.032222
H	3.041046	4.170914	-1.020667
H	3.493193	3.480065	0.533331
H	4.916354	3.024093	-2.129148
H	5.654932	1.895950	0.615568
H	6.544226	1.658526	-0.887455
H	2.494274	1.914456	-1.911085
H	1.611933	2.216974	-0.412010
H	6.348755	-0.814990	-0.491062
H	5.080839	-2.649269	0.425360
H	2.689474	-2.640182	-0.191590
H	2.488165	-0.788663	-1.509385
H	4.236548	-0.679162	2.459201
H	2.282941	-1.901096	4.549873
H	2.988753	-0.282023	4.634603
H	4.024808	-1.696323	4.777043
H	1.770126	-3.841465	1.787531
H	1.181860	-2.808735	3.098341
H	0.642012	-2.544416	1.438077
H	5.385520	5.253388	-1.109298

H	5.853670	4.473406	0.409962
H	6.824227	4.221620	-1.049963
H	-1.249629	-3.046390	-3.189522
H	-4.747174	-1.077591	-0.497336
H	-4.863165	-2.148265	0.893028
H	-2.934014	-1.323010	1.944351
H	-1.281029	0.091795	0.940452
H	-3.459877	-1.664675	-3.389193
H	-3.716163	-0.376447	-2.200136
H	-2.262937	-0.357324	-3.208844
H	-3.050973	1.663538	0.283071
H	-5.235125	2.600566	2.081935
H	-3.987198	3.684297	1.457081
H	-6.759265	4.337432	-0.567632

B3LYP Energy = -1950.55198278 a.u.

(3*R*,4*S*,5*R*,8*R*,10*S*,19*S*,24*S*)-**3**, Conf I

C	3.901969	2.765363	-1.224178
C	5.387999	2.363213	-1.220355
C	5.651877	1.302864	-0.136704
C	4.708217	0.093535	-0.276122
C	3.232454	0.540969	-0.191972
C	2.952496	1.558874	-1.314261
C	5.016119	-1.007261	0.705882
C	4.083024	-1.799939	1.243048
C	2.600893	-1.726399	0.942832
C	2.318173	-0.702856	-0.229278
C	1.790636	-1.533761	2.232544
C	0.927086	-2.499785	2.597378
C	0.044271	-2.559620	3.813790
C	2.042527	-0.277879	3.033436
C	0.834944	-0.372838	-0.256504
C	6.306807	3.580999	-1.062728
C	-0.103439	-1.286838	-0.908289
O	0.390511	0.653755	0.289531
C	0.174295	-2.564584	-1.604748
N	-1.068225	-3.150328	-1.855951
C	-2.166952	-2.248455	-1.607284
C	-1.473847	-1.134239	-0.847994
O	1.242626	-3.077266	-1.912560
S	-3.410206	-3.011464	-0.441384
C	-4.319952	-1.454477	-0.122828
C	-3.439537	-0.343774	0.473833
N	-2.151035	-0.144993	-0.216943
C	-2.811933	-1.775469	-2.922778
H	3.092420	1.055703	0.766834
H	4.869264	-0.315376	-1.290012
C	-4.276689	0.946316	0.532889
N	-3.934298	1.932527	-0.341499
C	-4.581992	3.224799	-0.321230
C	-3.843234	4.328938	0.428438
O	-5.223696	1.018280	1.309299
O	-2.741801	3.894451	1.076660
O	-4.214593	5.481476	0.434921
H	3.707968	3.456996	-2.054670
H	3.683544	3.322750	-0.300457
H	5.608231	1.897387	-2.194458
H	5.521705	1.754117	0.858739
H	6.695998	0.965387	-0.192231
H	3.077681	1.063382	-2.289220
H	1.917003	1.907345	-1.261288
H	6.063609	-1.165431	0.963113
H	4.374050	-2.579336	1.946263
H	2.311807	-2.707610	0.547761
H	2.538177	-1.262542	-1.146099
H	0.837960	-3.363861	1.937330
H	0.142901	-1.687976	4.464576
H	0.266944	-3.453146	4.411675
H	-1.011066	-2.639590	3.520935
H	1.629487	0.597353	2.518673
H	1.595846	-0.322774	4.028756
H	3.117693	-0.106181	3.154936
H	6.136572	4.313937	-1.859998
H	6.129418	4.085147	-0.104200
H	7.363367	3.290454	-1.095001
H	-1.131942	-3.876799	-2.556555
H	-4.811562	-1.120000	-1.040090
H	-5.103977	-1.687004	0.600124
H	-3.227639	-0.604800	1.518514
H	-1.479210	0.458916	0.274456
H	-3.251688	-2.631146	-3.443155
H	-3.595997	-1.033548	-2.759455
H	-2.043782	-1.325920	-3.560560
H	-3.084959	1.808431	-0.876710
H	-4.755458	3.587462	-1.338770
H	-5.557358	3.108010	0.160104
H	-2.350415	4.661493	1.529313

B3LYP Energy = -1950.55173170 a.u.

Table S13. Cartesian coordinates and energies of the low-energy conformers calculated at the ωB97X/TZVP PCM/MeOH level.

(3*R*,4*S*,5*R*,8*R*,10*S*,19*R*,24*R*)-**1**, Conf A

C	5.714247	-1.951501	-1.326664
C	6.888669	-1.561731	-0.428535
C	6.683407	-0.155890	0.135259
C	5.332195	-0.020365	0.836563
C	4.187817	-0.341775	-0.132525
C	4.359739	-1.773798	-0.642467
C	5.151189	1.321643	1.485947
C	3.980873	1.938572	1.576995
C	2.677457	1.384410	1.062031
C	2.851396	-0.089655	0.568349
C	2.036975	2.331661	0.050462
C	0.811658	2.798984	0.293339
C	-0.022980	3.698445	-0.566378
C	2.834212	2.668216	-1.180842
C	1.633971	-0.443306	-0.258001
C	8.217193	-1.679455	-1.164239
C	0.353180	-0.675643	0.431879
O	1.651318	-0.470919	-1.478470
C	0.110699	-0.824540	1.892899
N	-1.246122	-0.765821	2.068094
C	-1.979948	-0.824958	0.822054
C	-0.847640	-0.648154	-0.183750
O	0.919144	-0.960546	2.793863
N	-2.915216	0.299063	0.770956
C	-3.484045	0.676651	-0.524546
C	-2.995462	-0.124113	-1.738113
S	-1.187886	-0.290604	-1.835565
C	-5.013281	0.615382	-0.514463
N	-5.585895	0.256956	0.631982
C	-7.016506	0.326747	0.861515
C	-7.816021	-0.759342	0.144751
O	-7.660507	-0.825036	-1.173510
O	-5.657430	0.886393	-1.535023
O	-8.574845	-1.496832	0.721037
C	-2.683782	-2.175847	0.701030
H	4.275847	0.335036	-0.989972
H	5.309058	-0.779126	1.634075
H	5.834147	-2.987152	-1.659797
H	5.741390	-1.327860	-2.230109
H	6.902619	-2.257396	0.421521
H	6.742262	0.577026	-0.681008
H	7.490072	0.086083	0.835807
H	4.280828	-2.466169	0.205899
H	3.560548	-2.029374	-1.339801
H	6.036634	1.788512	1.912589
H	3.916800	2.912856	2.055820
H	1.991802	1.342080	1.914130
H	2.816758	-0.709420	1.470125
H	0.329953	2.480930	1.217618
H	-0.383102	4.557494	0.005406
H	0.509387	4.070605	-1.440790
H	-0.908456	3.162416	-0.923599
H	3.880392	2.851269	-0.925703
H	2.814144	1.836006	-1.890338
H	2.451255	3.550862	-1.690899
H	8.371742	-2.692822	-1.543584
H	8.245650	-0.994728	-2.017786
H	9.056565	-1.432181	-0.509165
H	-1.655488	-1.113312	2.922051
H	-2.461582	1.096379	1.196409
H	-3.249222	1.728599	-0.711157
H	-3.457943	-1.107123	-1.790296
H	-3.279060	0.407890	-2.643764
H	-4.954308	0.055559	1.396731
H	-7.394357	1.297973	0.527135
H	-7.200752	0.229804	1.927084
H	-7.017230	-0.138921	-1.479880
H	-1.947952	-2.978405	0.759591
H	-3.388459	-2.277205	1.527952
H	-3.235841	-2.286213	-0.228959

ωB97X Energy = -1950.62926283 a.u.

(3*R*,4*S*,5*R*,8*R*,10*S*,19*R*,24*R*)-**1**, Conf B

C	5.870211	-1.821041	-1.116219
C	7.006476	-1.283350	-0.245807
C	6.709782	0.152914	0.184407
C	5.345517	0.266152	0.863924
C	4.232796	-0.207733	-0.078759
C	4.498477	-1.665260	-0.461631
C	5.076243	1.644460	1.395101
C	3.867536	2.188383	1.434835
C	2.604456	1.507319	0.974244
C	2.875071	0.014765	0.590856
C	1.895183	2.330083	-0.098279
C	0.644023	2.733936	0.125802
C	-0.252946	3.519434	-0.781285
C	2.662043	2.624970	-1.359089
C	1.696793	-0.471504	-0.225543
C	8.350671	-1.390413	-0.954046
C	0.417953	-0.737095	0.454617
O	1.744293	-0.588086	-1.440091
C	0.142324	-0.764090	1.916730
N	-1.220077	-0.792969	2.053098
C	-1.907419	-1.036121	0.802776
C	-0.761957	-0.864842	-0.188839
O	0.930245	-0.749678	2.845612
N	-2.927364	-0.006175	0.610458
C	-3.516078	0.143713	-0.721555
C	-2.893336	-0.698632	-1.845667
S	-1.076346	-0.679700	-1.873404

C	-5.020136	-0.138819	-0.716772
N	-5.565484	-0.434037	0.460786
C	-6.936789	-0.880675	0.614022
C	-7.980948	0.217525	0.423207
O	-7.936098	0.871450	-0.732794
O	-5.673196	-0.069660	-1.764940
O	-8.825285	0.463123	1.246992
C	-2.499724	-2.444665	0.804893
H	4.284568	0.398030	-0.990867
H	5.360426	-0.421227	1.724084
H	6.054725	-2.873417	-1.353767
H	5.873455	-1.279416	-2.071341
H	7.047153	-1.896145	0.665015
H	6.732617	0.810313	-0.695458
H	7.492396	0.507044	0.864222
H	4.449310	-2.284344	0.443739
H	3.725601	-2.028537	-1.140685
H	5.927734	2.202997	1.778488
H	3.737527	3.193230	1.829987
H	1.927313	1.482319	1.833501
H	2.863341	-0.540837	1.534054
H	0.192633	2.455988	1.077969
H	-1.144678	2.934285	-1.027745
H	-0.601845	4.430410	-0.287697
H	0.226038	3.801645	-1.717935
H	2.707911	1.736887	-1.995998
H	2.211599	3.429992	-1.937787
H	3.690780	2.909168	-1.126435
H	8.571999	-2.425492	-1.226751
H	8.350666	-0.795057	-1.872602
H	9.163393	-1.027461	-0.319578
H	-1.628018	-1.087168	2.927488
H	-2.540644	0.875372	0.920694
H	-3.426778	1.193269	-1.013114
H	-3.244808	-1.727650	-1.826708
H	-3.194233	-0.280256	-2.803750
H	-4.936951	-0.425738	1.253710
H	-7.058195	-1.288261	1.613072
H	-7.145736	-1.671195	-0.113302
H	-7.195062	0.531615	-1.293204
H	-1.704435	-3.177617	0.942797
H	-3.204897	-2.525144	1.633797
H	-3.029907	-2.683815	-0.113577

ωB97X Energy = -1950.62907139 a.u.

(3*R*,4*S*,5*R*,8*R*,10*S*,19*R*,24*R*)-**1**, Conf C

C	5.656003	-2.039682	-1.290271
C	6.826402	-1.693721	-0.369348
C	6.654237	-0.287024	0.202917
C	5.296362	-0.115705	0.883117
C	4.158534	-0.394349	-0.106927
C	4.296199	-1.826719	-0.626786
C	5.146468	1.227094	1.538682
C	3.993903	1.878195	1.616560
C	2.681863	1.365313	1.081540
C	2.818758	-0.108422	0.574768
C	2.078951	2.340357	0.073505
C	0.866407	2.842299	0.311132
C	0.064284	3.772795	-0.546323
C	2.896842	2.664082	-1.147638
C	1.605293	-0.416536	-0.276649
C	8.163174	-1.845829	-1.083508
C	0.303369	-0.603329	0.385744
O	1.647897	-0.439175	-1.496820
C	0.026568	-0.730323	1.842341
N	-1.327583	-0.600694	1.991409
C	-2.042774	-0.635887	0.732942
C	-0.884046	-0.526242	-0.253249
O	0.810446	-0.900798	2.759778
N	-2.913186	0.534157	0.644220
C	-3.503908	0.855280	-0.658051
C	-2.981141	0.057775	-1.862834
S	-1.175602	-0.147110	-1.908879
C	-5.035143	0.732398	-0.652365
N	-5.589763	0.481370	0.541816
C	-7.002369	0.301314	0.691508
C	-7.468446	-1.076357	0.260566
O	-8.799158	-1.171831	0.347587
O	-5.679604	0.886352	-1.682561
O	-6.760054	-1.976769	-0.105501
C	-2.820902	-1.946089	0.619997
H	4.280221	0.287125	-0.956558
H	5.236562	-0.879053	1.674345
H	5.749967	-3.075561	-1.631030
H	5.717094	-1.409301	-2.187287
H	6.804837	-2.396513	0.474656
H	6.748769	0.450190	-0.606127
H	7.456129	-0.075224	0.918604
H	4.182446	-2.523375	0.214047
H	3.501358	-2.051976	-1.339316
H	6.039271	1.665117	1.980323
H	3.951965	2.851296	2.100272
H	1.986494	1.333688	1.925967
H	2.750242	-0.736130	1.469021
H	0.369132	2.531409	1.229647
H	-0.840277	3.269073	-0.902583
H	-0.263933	4.643664	0.026921
H	0.608866	4.126477	-1.420936
H	2.540090	3.557784	-1.657362
H	3.944099	2.821868	-0.879998
H	2.865655	1.835928	-1.861428
H	8.293922	-2.860394	-1.468589
H	8.226499	-1.155625	-1.930745
H	8.998178	-1.628989	-0.412272
H	-1.769608	-0.913875	2.842225
H	-2.399965	1.329861	0.999264
H	-3.307966	1.912481	-0.855444
H	-3.455658	-0.917477	-1.941040
H	-3.224840	0.598236	-2.774857
H	-4.954192	0.329242	1.311707
H	-7.543458	1.038440	0.095566

H	-7.285372	0.448245	1.734024
H	-9.064287	-2.061670	0.075739
H	-2.130970	-2.788558	0.674576
H	-3.523145	-2.006232	1.453167
H	-3.390311	-2.026594	-0.302657

ωB97X Energy = -1950.62880665 a.u.

(3*R*,4*S*,5*R*,8*R*,10*S*,19*R*,24*R*)-**1**, Conf D

C	-5.944958	-1.778101	0.936337
C	-7.044293	-1.141414	0.085791
C	-6.689964	0.309746	-0.238140
C	-5.308519	0.423235	-0.882446
C	-4.232364	-0.158379	0.042416
C	-4.556733	-1.628371	0.316534
C	-4.979629	1.827717	-1.299723
C	-3.751351	2.327174	-1.278910
C	-2.521524	1.564560	-0.857509
C	-2.854933	0.059589	-0.587354
C	-1.794111	2.277703	0.279637
C	-0.522490	2.638749	0.102402
C	0.396983	3.306251	1.079084
C	-2.568029	2.513911	1.548106
C	-1.710464	-0.533310	0.207086
C	-8.404884	-1.248295	0.762170
C	-0.430853	-0.793103	-0.473052
O	-1.783735	-0.738494	1.408682
C	-0.138448	-0.738252	-1.930940
N	1.223299	-0.793922	-2.054467
C	1.893358	-1.117438	-0.812415
C	0.738539	-0.984818	0.174465
O	-0.915233	-0.647997	-2.865619
N	2.926800	-0.116371	-0.555139
C	3.486519	-0.022591	0.796557
C	2.854141	-0.935712	1.855738
S	1.036667	-0.915885	1.870326
C	5.001287	-0.273087	0.824567
N	5.563884	-0.566144	-0.356477
C	6.977906	-0.754547	-0.485795
C	7.748207	0.551895	-0.498439
O	9.065261	0.322837	-0.525675
O	5.624024	-0.209125	1.877353
O	7.264453	1.652883	-0.493744
C	2.464254	-2.532742	-0.889356
H	-4.280260	0.382634	0.994492
H	-5.328460	-0.196166	-1.792726
H	-6.170826	-2.836494	1.099728
H	-5.946916	-1.301413	1.925443
H	-7.088257	-1.688232	-0.865871
H	-6.709364	0.904523	0.685265
H	-7.446067	0.737297	-0.905693
H	-4.512693	-2.183818	-0.629392
H	-3.809519	-2.065704	0.980395
H	-5.803191	2.447575	-1.648420
H	-3.578537	3.355168	-1.588938
H	-1.835170	1.575128	-1.709888
H	-2.847889	-0.426333	-1.568234
H	-0.068164	2.409221	-0.861160
H	1.213240	2.628773	1.351073
H	0.856948	4.192565	0.634817
H	-0.100838	3.606615	2.000205
H	-3.568198	2.892161	1.324124
H	-2.691798	1.578026	2.100303
H	-2.075758	3.227918	2.206571
H	-8.664449	-2.290633	0.964366
H	-8.402859	-0.712698	1.716778
H	-9.193335	-0.818802	0.138749
H	1.637263	-1.038496	-2.941163
H	2.563811	0.782352	-0.843175
H	3.366106	1.007853	1.141898
H	3.206388	-1.961417	1.772312
H	3.147798	-0.580166	2.841041
H	4.963203	-0.516254	-1.166856
H	7.195907	-1.289143	-1.410578
H	7.355467	-1.358781	0.340839
H	9.526582	1.173063	-0.544875
H	1.660619	-3.245239	-1.077683
H	3.178005	-2.574112	-1.713672
H	2.982633	-2.830799	0.018470

ωB97X Energy = -1950.62835076 a.u.

(3*R*,4*S*,5*R*,8*R*,10*S*,19*R*,24*R*)-**1**, Conf E

C	5.746739	-1.381630	-1.784513
C	6.847705	-1.461996	-0.726413
C	6.620920	-0.404535	0.353672
C	5.219164	-0.502154	0.955040
C	4.153262	-0.338025	-0.135628
C	4.343516	-1.437301	-1.182576
C	5.010075	0.465152	2.084573
C	3.845851	1.044299	2.344174
C	2.576572	0.806751	1.567328
C	2.766203	-0.331437	0.510688
C	2.018633	2.116125	1.014411
C	0.786761	2.487918	1.365070
C	0.023995	3.702362	0.930974
C	2.905912	2.905848	0.090768
C	1.620746	-0.238173	-0.475202
C	8.231397	-1.333765	-1.350304
C	0.277798	-0.664411	-0.047260
O	1.751010	0.246767	-1.588353
C	-0.104894	-1.378746	1.201142
N	-1.469115	-1.309408	1.283274
C	-2.085622	-0.818614	0.068566
C	-0.859640	-0.318385	-0.688087
O	0.611870	-1.919095	2.025499
N	-2.982362	0.285419	0.398686
C	-3.483391	1.118548	-0.697102
C	-2.844193	0.891249	-2.076605
S	-1.034945	0.715786	-2.055598
C	-5.005670	1.002429	-0.874329
N	-5.627401	0.214246	0.007263

C	-7.043784	-0.019230	-0.032320
C	-7.383846	-1.233781	0.793046
O	-8.687688	-1.504353	0.744722
O	-5.578726	1.609936	-1.772397
O	-6.587610	-1.878936	1.426806
C	-2.811455	-1.962995	-0.637866
H	4.323396	0.627789	-0.624973
H	5.115861	-1.518463	1.365820
H	5.875499	-2.187792	-2.513618
H	5.860380	-0.439407	-2.336712
H	6.775505	-2.446040	-0.243763
H	6.759185	0.594786	-0.081221
H	7.372158	-0.511877	1.143753
H	4.184274	-2.413697	-0.706591
H	3.599583	-1.341962	-1.974847
H	5.867221	0.678366	2.720100
H	3.760597	1.742616	3.173575
H	1.833559	0.438524	2.281474
H	2.640395	-1.270393	1.059299
H	0.240333	1.829868	2.040331
H	-0.871515	3.405886	0.375220
H	-0.318217	4.274579	1.797303
H	0.603914	4.366779	0.291722
H	2.969637	2.420343	-0.887081
H	2.544161	3.921325	-0.062712
H	3.921256	2.964120	0.489950
H	8.400606	-2.109255	-2.101808
H	8.342998	-0.361933	-1.841426
H	9.016666	-1.418635	-0.594695
H	-1.967817	-1.939658	1.892812
H	-2.525549	0.859745	1.094257
H	-3.309375	2.162482	-0.424530
H	-3.280254	0.037229	-2.589688
H	-3.034477	1.762213	-2.699782
H	-5.061480	-0.241272	0.711784
H	-7.378136	-0.181845	-1.059247
H	-7.612875	0.830438	0.357279
H	-8.865052	-2.281152	1.293939
H	-2.103365	-2.759180	-0.868862
H	-3.580733	-2.352355	0.031654
H	-3.294450	-1.654129	-1.561389

ωB97X Energy = -1950.62764991 a.u.

(3*R*,4*S*,5*R*,8*R*,10*S*,19*R*,24*R*)-**1**, Conf F

C	-5.623265	-2.085843	1.256775
C	-6.801898	-1.718799	0.354568
C	-6.639057	-0.296433	-0.180599
C	-5.286858	-0.102646	-0.865978
C	-4.141181	-0.402418	0.108633
C	-4.268954	-1.849096	0.589923
C	-5.146560	1.256179	-1.489702
C	-3.997646	1.914773	-1.557536
C	-2.680758	1.396280	-1.040251
C	-2.806986	-0.091334	-0.572979
C	-2.080360	2.348756	-0.009347
C	-0.872050	2.864644	-0.238432
C	-0.072866	3.778850	0.639083
C	-2.895979	2.637195	1.222063
C	-1.587058	-0.414922	0.263251
C	-8.132602	-1.895465	1.074353
C	-0.288373	-0.580023	-0.411206
O	-1.622239	-0.466979	1.482742
C	-0.020171	-0.672456	-1.872064
N	1.332110	-0.530838	-2.026618
C	2.055807	-0.588962	-0.773953
C	0.902765	-0.509761	0.221770
O	-0.808917	-0.827208	-2.788132
N	2.916744	0.585607	-0.661009
C	3.534126	0.855749	0.640022
C	3.007166	0.044250	1.835290
S	1.201599	-0.159293	1.882256
C	5.059977	0.689278	0.605382
N	5.594246	0.481436	-0.605505
C	7.005741	0.283507	-0.771090
C	7.540947	-1.029520	-0.230835
O	6.602401	-1.956759	-0.032631
O	5.726024	0.763575	1.631205
O	8.713975	-1.221252	-0.031632
C	2.847182	-1.893839	-0.696279
H	-4.260652	0.254676	0.977478
H	-5.230266	-0.845861	-1.676440
H	-5.710996	-3.131349	1.568566
H	-5.680058	-1.481310	2.171658
H	-6.784505	-2.397959	-0.508719
H	-6.729936	0.418583	0.648502
H	-7.446994	-0.068468	-0.884402
H	-4.157948	-2.521903	-0.270458
H	-3.468225	-2.089977	1.290725
H	-6.043533	1.699797	-1.917089
H	-3.962789	2.898991	-2.018772
H	-1.988017	1.391522	-1.887432
H	-2.740438	-0.694020	-1.484381
H	-0.376063	2.580289	-1.166236
H	0.246635	4.666303	0.086726
H	-0.616279	4.106388	1.524572
H	0.836752	3.272632	0.978555
H	-2.542777	3.520099	1.752650
H	-3.945034	2.795521	0.961905
H	-2.857664	1.792002	1.915210
H	-8.258587	-2.921805	1.428580
H	-8.190345	-1.232165	1.943190
H	-8.973353	-1.659546	0.416854
H	1.770855	-0.821187	-2.887165
H	2.387821	1.390234	-0.970048
H	3.371063	1.913238	0.862878
H	3.478235	-0.933586	1.899492
H	3.252531	0.569922	2.755481
H	4.950443	0.388868	-1.377683
H	7.569275	1.076076	-0.277385
H	7.248312	0.317668	-1.833811
H	7.034814	-2.757573	0.296386

H	2.164103	-2.741316	-0.758347
H	3.539124	-1.931424	-1.539270
H	3.430735	-1.986778	0.216463

ωB97X Energy = -1950.62723388 a.u.

(3*R*,4*S*,5*R*,8*R*,10*S*,19*R*,24*R*)-**1**, Conf G

C	5.181887	-2.193476	-1.599306
C	6.389216	-2.226902	-0.661227
C	6.468781	-0.933678	0.150203
C	5.155664	-0.650094	0.878972
C	4.000004	-0.531845	-0.121738
C	3.875936	-1.854263	-0.881271
C	5.238357	0.543169	1.786400
C	4.244536	1.403836	1.961121
C	2.905585	1.309384	1.277641
C	2.738723	-0.106737	0.633337
C	2.667320	2.486115	0.328390
C	3.657046	2.986746	-0.409777
C	3.604626	4.119594	-1.389432
C	1.266085	3.037660	0.346034
C	1.482806	-0.139379	-0.206068
C	7.680910	-2.489500	-1.424558
C	0.186168	-0.392720	0.445584
O	1.496265	0.071777	-1.409294
C	-0.086617	-0.661561	1.885105
N	-1.447104	-0.619950	2.034786
C	-2.154364	-0.592543	0.772561
C	-1.004655	-0.311409	-0.189227
O	0.701879	-0.861291	2.791594
N	-3.114788	0.508326	0.786057
C	-3.708706	0.918340	-0.487546
C	-3.130322	0.270836	-1.755290
S	-1.314680	0.208512	-1.801109
C	-5.224399	0.707305	-0.504938
N	-5.776325	0.269816	0.624512
C	-7.210170	0.211550	0.836801
C	-7.908260	-0.919114	0.083901
O	-7.732625	-0.939284	-1.233229
O	-5.878175	0.942043	-1.528185
O	-8.609791	-1.730724	0.632507
C	-2.825025	-1.943968	0.527697
H	4.251306	0.252525	-0.846112
H	4.948476	-1.527330	1.511828
H	5.088394	-3.155639	-2.112570
H	5.364237	-1.440600	-2.377556
H	6.236283	-3.049518	0.050537
H	6.701236	-0.094775	-0.519848
H	7.288990	-0.998013	0.873529
H	3.625472	-2.652563	-0.170526
H	3.063815	-1.805641	-1.608086
H	6.168573	0.682111	2.333696
H	4.374087	2.252818	2.627147
H	2.142303	1.382496	2.060976
H	2.584982	-0.795095	1.469219
H	4.639200	2.532723	-0.298030
H	3.917919	3.778818	-2.380168
H	2.614888	4.563530	-1.486861
H	4.301052	4.910159	-1.096298
H	0.524781	2.264644	0.124630
H	1.030331	3.419921	1.344070
H	1.119506	3.846657	-0.367178
H	7.631812	-3.434300	-1.971985
H	7.868117	-1.691614	-2.150121
H	8.538528	-2.535359	-0.748384
H	-1.871912	-1.031874	2.851727
H	-2.674653	1.299259	1.236691
H	-3.579216	1.999500	-0.584634
H	-3.531579	-0.725773	-1.923757
H	-3.411962	0.872728	-2.616647
H	-5.139583	0.110415	1.394670
H	-7.665484	1.155738	0.522246
H	-7.396724	0.071902	1.897174
H	-7.145360	-0.194467	-1.514342
H	-2.070935	-2.731254	0.518126
H	-3.528155	-2.135688	1.339804
H	-3.373533	-1.984225	-0.410067

ωB97X Energy = -1950.62699941 a.u.

(3*R*,4*S*,5*R*,8*R*,10*S*,19*R*,24*R*)-**1**, Conf H

C	5.934194	-1.756411	-0.989145
C	7.036197	-1.133756	-0.131648
C	6.681937	0.311205	0.218986
C	5.303348	0.411340	0.871461
C	4.223669	-0.154201	-0.059179
C	4.548098	-1.618576	-0.361795
C	4.974517	1.806973	1.317421
C	3.745393	2.304820	1.312590
C	2.514695	1.548714	0.881960
C	2.849150	0.049769	0.581389
C	1.781289	2.282027	-0.238395
C	0.509254	2.635568	-0.049527
C	-0.415824	3.319146	-1.009619
C	2.549677	2.544559	-1.505032
C	1.701471	-0.528806	-0.218704
C	8.394386	-1.227981	-0.814669
C	0.426987	-0.806918	0.463949
O	1.767644	-0.707603	-1.424869
C	0.145726	-0.792733	1.925005
N	-1.214950	-0.856397	2.057573
C	-1.893452	-1.147397	0.812138
C	-0.746790	-0.983955	-0.179576
O	0.929486	-0.725653	2.855750
N	-2.932371	-0.143084	0.591139
C	-3.499754	-0.009196	-0.754063
C	-2.875376	-0.892297	-1.842737
S	-1.058059	-0.870942	-1.870835
C	-5.014446	-0.256808	-0.779182
N	-5.571440	-0.587658	0.393774
C	-6.985053	-0.804298	0.513519
C	-7.845744	0.442844	0.437601

O	-7.170771	1.579325	0.613776
O	-5.648512	-0.151067	-1.822244
O	-9.037495	0.399416	0.262802
C	-2.458383	-2.566589	0.852311
H	4.266376	0.404441	-1.001285
H	5.328110	-0.225252	1.769698
H	6.160130	-2.811718	-1.171350
H	5.932332	-1.262888	-1.969972
H	7.084070	-1.697025	0.810197
H	6.696540	0.922215	-0.693879
H	7.440582	0.727757	0.890594
H	4.507730	-2.191437	0.573863
H	3.798649	-2.044046	-1.030908
H	5.798855	2.421050	1.674412
H	3.572665	3.326019	1.644366
H	1.831876	1.542120	1.737315
H	2.847711	-0.454944	1.552719
H	0.059198	2.385328	0.910875
H	0.077703	3.639543	-1.926297
H	-1.230639	2.644176	-1.292151
H	-0.877247	4.194953	-0.546382
H	2.671708	1.619913	-2.076258
H	2.053887	3.271162	-2.146873
H	3.550496	2.919057	-1.277858
H	8.654577	-2.266669	-1.034148
H	8.388312	-0.677475	-1.760750
H	9.184520	-0.807389	-0.187321
H	-1.620587	-1.129021	2.939892
H	-2.570982	0.747820	0.904472
H	-3.379714	1.030617	-1.070245
H	-3.226928	-1.920000	-1.784752
H	-3.177058	-0.509664	-2.815450
H	-4.967839	-0.573983	1.203139
H	-7.193513	-1.280334	1.472248
H	-7.338268	-1.474877	-0.270487
H	-7.798450	2.314179	0.563071
H	-1.650404	-3.281235	1.011878
H	-3.164550	-2.635945	1.681325
H	-2.983387	-2.838975	-0.059827

ωB97X Energy = -1950.62680612 a.u.

(3*R*,4*S*,5*R*,8*R*,10*S*,19*R*,24*R*)-**1**, Conf I

C	5.558521	-1.668157	-1.639800
C	6.715845	-1.622453	-0.641164
C	6.579302	-0.409827	0.279591
C	5.208206	-0.371280	0.953509
C	4.093113	-0.319425	-0.098514
C	4.187209	-1.571935	-0.971811
C	5.084584	0.735274	1.960630
C	3.975785	1.438558	2.146802
C	2.695762	1.230588	1.380473
C	2.754381	-0.135166	0.618918
C	2.346841	2.444717	0.515776
C	3.296168	3.134077	-0.116299
C	3.138368	4.325630	-1.011674
C	0.883940	2.802531	0.480638
C	1.544999	-0.269030	-0.275686
C	8.063655	-1.636503	-1.350806
C	0.267521	-0.713747	0.308840
O	1.571399	0.015385	-1.463488
C	-0.009121	-1.142771	1.708614
N	-1.369078	-1.258033	1.815505
C	-2.046848	-1.175148	0.539701
C	-0.909714	-0.694196	-0.354380
O	0.774915	-1.346077	2.618166
N	-3.115581	-0.181063	0.635086
C	-3.667285	0.374722	-0.602111
C	-3.047592	-0.142063	-1.906564
S	-1.231207	-0.076630	-1.929814
C	-5.178893	0.158191	-0.700859
N	-5.751424	-0.509571	0.297712
C	-7.134946	-0.944656	0.273806
C	-8.152119	0.179275	0.460080
O	-8.073874	1.187422	-0.402084
O	-5.811425	0.602686	-1.666440
O	-9.005579	0.149788	1.310196
C	-2.570072	-2.556156	0.144978
H	4.265868	0.554384	-0.738915
H	5.095957	-1.322154	1.497731
H	5.622294	-2.585325	-2.233775
H	5.669668	-0.832591	-2.343547
H	6.648374	-2.520037	-0.011463
H	6.723452	0.510784	-0.302093
H	7.367606	-0.430228	1.039907
H	4.017003	-2.458045	-0.346354
H	3.409704	-1.565407	-1.736665
H	5.959976	0.945279	2.571938
H	3.957150	2.233906	2.887157
H	1.894229	1.135262	2.122025
H	2.664417	-0.904474	1.391536
H	4.324805	2.809501	0.023889
H	3.656518	5.192794	-0.592392
H	3.595384	4.129078	-1.985230
H	2.099390	4.603993	-1.182344
H	0.281779	2.010275	0.026532
H	0.509022	2.938009	1.499481
H	0.687496	3.719336	-0.072082
H	8.169334	-2.522119	-1.982665
H	8.170639	-0.754425	-1.989984
H	8.888909	-1.632789	-0.634077
H	-1.768738	-1.785866	2.576584
H	-2.790744	0.565168	1.234941
H	-3.533659	1.459606	-0.586590
H	-3.382678	-1.147950	-2.148886
H	-3.364182	0.504785	-2.722075
H	-5.137219	-0.794036	1.050025
H	-7.284422	-1.666250	1.071242
H	-7.346282	-1.432717	-0.682593
H	-7.329141	1.041707	-1.037188
H	-1.742218	-3.264499	0.102037
H	-3.281706	-2.886250	0.903385

H	-3.075258	-2.561887	-0.817530

ωB97X Energy = -1950.62673375 a.u.

(3*R*,4*S*,5*R*,8*R*,10*S*,19*R*,24*R*)-**1**, Conf J

C	5.125870	2.252523	1.575169
C	6.330719	2.295595	0.634251
C	6.429747	0.995195	-0.163529
C	5.120048	0.683260	-0.886885
C	3.968402	0.556537	0.117420
C	3.824228	1.884475	0.863592
C	5.219688	-0.517428	-1.782843
C	4.239238	-1.395437	-1.946946
C	2.900845	-1.316094	-1.260297
C	2.712679	0.103905	-0.630588
C	2.685900	-2.486418	-0.297573
C	3.686484	-2.962001	0.442539
C	3.656282	-4.084148	1.435391
C	1.294477	-3.062439	-0.304659
C	1.457473	0.125472	0.210559
C	7.619189	2.587286	1.392491
C	0.156808	0.352565	-0.441754
O	1.476772	-0.075510	1.415602
C	-0.118916	0.625570	-1.879760
N	-1.477140	0.554618	-2.032982
C	-2.188011	0.498697	-0.773033
C	-1.033880	0.238621	0.189553
O	0.667019	0.850453	-2.783302
N	-3.122398	-0.622746	-0.799457
C	-3.710052	-1.063295	0.468770
C	-3.149431	-0.411262	1.742122
S	-1.336416	-0.299261	1.796334
C	-5.234819	-0.884892	0.503251
N	-5.792071	-0.421123	-0.624023
C	-7.195185	-0.145644	-0.704301
C	-7.579033	1.140915	0.002324
O	-8.906942	1.295032	0.012397
O	-5.874379	-1.171349	1.507956
O	-6.814005	1.933934	0.484530
C	-2.890887	1.832093	-0.518809
H	4.233567	-0.216303	0.849241
H	4.897549	1.550712	-1.527987
H	5.017848	3.218366	2.078593
H	5.322023	1.510871	2.360780
H	6.163137	3.108027	-0.085876
H	6.676461	0.167326	0.515062
H	7.247774	1.064884	-0.888847
H	3.559081	2.671211	0.145319
H	3.014516	1.829797	1.592682
H	6.150801	-0.647035	-2.330928
H	4.381054	-2.248890	-2.604719
H	2.136164	-1.410834	-2.040174
H	2.546682	0.780640	-1.473501
H	4.660496	-2.492807	0.322477
H	3.972016	-3.727535	2.419754
H	2.673226	-4.539870	1.545123
H	4.361074	-4.868846	1.146487
H	1.162403	-3.861831	0.422125
H	0.539872	-2.298844	-0.096076
H	1.065232	-3.465703	-1.295935
H	7.555135	3.536581	1.930509
H	7.820885	1.799894	2.125560
H	8.474784	2.640717	0.714301
H	-1.909632	0.960503	-2.848836
H	-2.663846	-1.397815	-1.258846
H	-3.547258	-2.140449	0.554717
H	-3.577419	0.573297	1.916878
H	-3.418621	-1.026514	2.597885
H	-5.163666	-0.186902	-1.378694
H	-7.770461	-0.959046	-0.258825
H	-7.495266	-0.062851	-1.749103
H	-9.118625	2.129329	0.454189
H	-2.155427	2.636633	-0.494938
H	-3.592630	2.015898	-1.334116
H	-3.449916	1.847930	0.413396

ωB97X Energy = -1950.62652446 a.u.

(3*R*,4*S*,5*R*,8*R*,10*S*,19*R*,24*R*)-**1**, Conf K

C	-5.604119	-1.836590	1.359862
C	-6.738789	-1.658119	0.350511
C	-6.576290	-0.337348	-0.401936
C	-5.191998	-0.225203	-1.039896
C	-4.095723	-0.322363	0.027936
C	-4.219372	-1.674515	0.733742
C	-5.040567	1.000985	-1.892786
C	-3.920314	1.706452	-1.971503
C	-2.654867	1.383509	-1.220809
C	-2.742028	-0.065861	-0.637218
C	-2.301995	2.475871	-0.207719
C	-3.252348	3.103874	0.484079
C	-3.089064	4.172116	1.522508
C	-0.833168	2.793022	-0.091557
C	-1.552836	-0.323273	0.258073
C	-8.102589	-1.756137	1.021850
C	-0.261832	-0.686735	-0.350502
O	-1.608695	-0.197552	1.472144
C	0.048764	-0.913188	-1.789585
N	1.411714	-0.996748	-1.881828
C	2.063672	-1.090804	-0.592734
C	0.901102	-0.755915	0.335774
O	-0.714089	-0.995489	-2.735983
N	3.112532	-0.077621	-0.512245
C	3.676808	0.233766	0.804040
C	3.000022	-0.435551	2.008875
S	1.184330	-0.354272	1.986029
C	5.178909	-0.073590	0.888802
N	5.731640	-0.579874	-0.223267
C	7.139645	-0.825545	-0.317264
C	7.939292	0.432332	-0.597203
O	9.248341	0.162072	-0.628281
O	5.800545	0.138973	1.922420

O	7.482064	1.530341	-0.774892
C	2.608320	-2.504683	-0.393388
H	-4.269797	0.464835	0.772170
H	-5.077963	-1.097814	-1.702207
H	-5.687904	-2.817423	1.838306
H	-5.723450	-1.089283	2.155531
H	-6.659100	-2.467331	-0.387998
H	-6.725459	0.500045	0.293197
H	-7.349659	-0.250780	-1.172978
H	-4.047469	-2.476141	0.003485
H	-3.455749	-1.774837	1.506021
H	-5.903554	1.299813	-2.484516
H	-3.880460	2.590540	-2.602355
H	-1.844521	1.368306	-1.958099
H	-2.643923	-0.734585	-1.497499
H	-4.283817	2.821998	0.284740
H	-3.539997	3.852738	2.466027
H	-2.048704	4.425705	1.721140
H	-3.609750	5.085199	1.220428
H	-0.404535	2.955666	-1.084625
H	-0.642747	3.684025	0.503973
H	-0.274177	1.970409	0.364079
H	-8.226127	-2.716579	1.528833
H	-8.221060	-0.965499	1.769550
H	-8.911286	-1.652709	0.293735
H	1.833183	-1.406752	-2.701395
H	2.762302	0.765009	-0.947619
H	3.604773	1.314346	0.950204
H	3.314403	-1.469218	2.132797
H	3.293201	0.093876	2.912847
H	5.134868	-0.637143	-1.036164
H	7.339134	-1.543838	-1.112653
H	7.508979	-1.256354	0.614869
H	9.729183	0.980799	-0.813796
H	1.792507	-3.225763	-0.451537
H	3.326139	-2.714227	-1.188060
H	3.114930	-2.634786	0.559497

ωB97X Energy = -1950.62612613 a.u.

(3*R*,4*S*,5*R*,8*R*,10*S*,19*R*,24*S*)-**1**, Conf A

C	-5.611046	-1.139118	0.818082
C	-6.531393	-0.278439	-0.049042
C	-5.891840	1.084638	-0.315453
C	-4.489919	0.939486	-0.906131
C	-3.588924	0.146738	0.048075
C	-4.193121	-1.242823	0.257404
C	-3.879750	2.257429	-1.290065
C	-2.585017	2.525488	-1.184916
C	-1.551385	1.556909	-0.668386
C	-2.164949	0.129541	-0.512135
C	-0.860511	2.108034	0.576589
C	0.463078	2.266716	0.562473
C	1.342780	2.760491	1.671259
C	-1.723666	2.436124	1.765542
C	-1.190568	-0.708405	0.284639
C	-7.911614	-0.133358	0.578924
C	-0.051185	-1.338161	-0.410654
O	-1.285620	-0.849320	1.493174
C	0.116998	-1.569781	-1.875717
N	1.327512	-2.171694	-2.027880
C	1.931958	-2.572068	-0.767429
C	1.039473	-1.827681	0.217202
O	-0.645252	-1.287007	-2.785743
N	3.336128	-2.185632	-0.704661
C	3.872787	-1.653481	0.538017
C	3.198814	-2.151449	1.809457
S	1.429534	-1.718998	1.895155
C	3.972432	-0.124416	0.512902
N	3.813367	0.446289	-0.680670
C	3.894387	1.876639	-0.904994
C	5.305861	2.450294	-0.792340
O	5.926242	2.249486	0.366691
O	4.272082	0.510204	1.530637
O	5.825390	3.083432	-1.675495
C	1.752268	-4.084103	-0.565000
H	-3.589613	0.660661	1.014924
H	-4.592693	0.347124	-1.828703
H	-6.044058	-2.137360	0.935837
H	-5.563867	-0.698008	1.822454
H	-6.644651	-0.782430	-1.018433
H	-5.832365	1.650017	0.624554
H	-6.524547	1.666056	-0.994860
H	-4.211653	-1.772988	-0.703660
H	-3.573063	-1.832224	0.935897
H	-4.551396	3.013066	-1.692554
H	-2.207198	3.502125	-1.477960
H	-0.774202	1.476193	-1.436270
H	-2.204031	-0.291091	-1.520779
H	0.984884	1.993156	-0.354830
H	1.891602	3.656887	1.365514
H	0.789551	3.001664	2.577733
H	2.090100	2.003735	1.931263
H	-1.974436	1.525391	2.316497
H	-1.231398	3.121065	2.454263
H	-2.661954	2.895194	1.447756
H	-8.380563	-1.108349	0.734474
H	-7.840634	0.362988	1.551877
H	-8.574288	0.462963	-0.053546
H	1.572262	-2.620969	-2.896472
H	3.912963	-2.941028	-1.045027
H	4.916691	-1.974613	0.597636
H	3.328126	-3.228291	1.902214
H	3.648924	-1.674179	2.677877
H	3.603395	-0.180808	-1.447088
H	3.268260	2.399053	-0.176471
H	3.521834	2.092100	-1.902029
H	5.358080	1.719068	0.976314
H	2.120968	-4.395034	0.412542
H	0.700987	-4.359241	-0.652032
H	2.317974	-4.616496	-1.332836

ωB97X Energy = -1950.62935489 a.u.

(3*R*,4*S*,5*R*,8*R*,10*S*,19*R*,24*S*)-**1**, Conf B

C	5.247437	-1.712486	-0.524840
C	6.214592	-0.785249	0.212778
C	5.691823	0.651429	0.200311
C	4.264887	0.732876	0.741902
C	3.324471	-0.146939	-0.090567
C	3.809879	-1.595612	-0.019931
C	3.762007	2.144580	0.848874
C	2.499897	2.492956	0.635522
C	1.400183	1.531436	0.262322
C	1.891537	0.057130	0.406219
C	0.797637	1.887433	-1.094608
C	-0.509388	2.137074	-1.172521
C	-1.317373	2.486817	-2.384689
C	1.721212	1.922107	-2.282453
C	0.865501	-0.840400	-0.247352
C	7.620451	-0.873250	-0.367164
C	-0.343632	-1.216732	0.509440
O	0.981120	-1.235225	-1.396779
C	-0.587882	-1.094352	1.977397
N	-1.837811	-1.579707	2.193940
C	-2.428003	-2.206398	1.021775
C	-1.445605	-1.758555	-0.053604
O	0.152243	-0.647692	2.840083
N	-3.797500	-1.759736	0.805735
C	-4.221970	-1.415091	-0.542907
C	-3.571987	-2.233865	-1.650715
S	-1.764353	-1.999992	-1.732139
C	-4.121882	0.093705	-0.825633
N	-3.914517	0.853311	0.260498
C	-3.957938	2.290575	0.215668
C	-3.052577	2.970547	1.215041
O	-2.462854	2.128434	2.067871
O	-4.284310	0.536655	-1.955725
O	-2.898634	4.164345	1.247031
C	-2.354721	-3.734258	1.160772
H	3.395699	0.178472	-1.133744
H	4.287490	0.315064	1.760451
H	5.593271	-2.747314	-0.438533
H	5.270184	-1.463622	-1.594075
H	6.253191	-1.110111	1.261420
H	5.713279	1.040447	-0.826803
H	6.352064	1.292067	0.795045
H	3.751760	-1.942658	1.020004
H	3.160883	-2.244803	-0.610804
H	4.485021	2.905245	1.136468
H	2.202636	3.535080	0.725675
H	0.596518	1.660526	0.996075
H	1.871296	-0.158273	1.478331
H	-1.077437	2.060477	-0.246464
H	-1.812822	3.454056	-2.252325
H	-0.720744	2.538095	-3.294530
H	-2.107418	1.745279	-2.540500
H	1.913046	0.907603	-2.643220
H	1.306005	2.498014	-3.108314
H	2.683893	2.362024	-2.014485
H	8.003715	-1.895986	-0.323817
H	7.624423	-0.558456	-1.415471
H	8.315143	-0.229499	0.178491
H	-2.166320	-1.777902	3.125770
H	-4.440567	-2.418017	1.220633
H	-5.295139	-1.618502	-0.594720
H	-3.809565	-3.289290	-1.527074
H	-3.941445	-1.904483	-2.619952
H	-3.837804	0.365766	1.143646
H	-4.967037	2.673453	0.399242
H	-3.669756	2.627764	-0.780977
H	-1.917652	2.641150	2.681999
H	-2.708624	-4.225082	0.253815
H	-1.331808	-4.052987	1.361732
H	-2.988803	-4.045816	1.993939

ωB97X Energy = -1950.62686763 a.u.

(3*R*,4*S*,5*R*,8*R*,10*S*,19*R*,24*S*)-**1**, Conf C

C	4.522005	-2.085217	-1.103224
C	5.729568	-1.685092	-0.253325
C	5.639842	-0.211220	0.144825
C	4.305992	0.101405	0.822011
C	3.141548	-0.227932	-0.116716
C	3.187162	-1.718864	-0.455040
C	4.225048	1.507366	1.342664
C	3.113230	2.230804	1.341861
C	1.780104	1.760960	0.803959
C	1.828680	0.228933	0.538001
C	1.366474	2.698690	-0.330204
C	0.465336	3.652511	-0.069133
C	0.014454	4.770455	-0.964837
C	2.113957	2.607228	-1.631298
C	0.611565	-0.260770	-0.223332
C	7.038038	-1.990453	-0.971001
C	-0.469529	-0.953808	0.494427
O	0.530594	-0.150982	-1.438129
C	-0.642236	-1.110191	1.966837
N	-1.798130	-1.806544	2.142162
C	-2.327984	-2.355361	0.902140
C	-1.505174	-1.575111	-0.116617
O	0.065100	-0.689322	2.868888
N	-3.765390	-2.142206	0.796044
C	-4.325378	-1.731339	-0.483482
C	-3.580017	-2.263271	-1.702168
S	-1.886464	-1.592505	-1.795887
C	-4.493363	-0.205931	-0.586767
N	-4.314319	0.462995	0.562290
C	-4.380599	1.893666	0.631944
C	-3.083506	2.621851	0.326299
O	-2.106087	1.828343	-0.100119
O	-4.805525	0.324972	-1.644488
O	-2.973963	3.816550	0.460463

C	-1.969163	-3.846546	0.806516
H	3.290474	0.327735	-1.047544
H	4.226083	-0.570545	1.690843
H	4.557741	-3.159563	-1.308636
H	4.595012	-1.578733	-2.074552
H	5.697211	-2.275689	0.672266
H	5.750278	0.417624	-0.748897
H	6.467020	0.043642	0.816122
H	3.046566	-2.300748	0.465266
H	2.371253	-1.987114	-1.130796
H	5.133916	1.929225	1.766949
H	3.123478	3.241647	1.741634
H	1.044360	1.908148	1.602458
H	1.786490	-0.227196	1.529685
H	0.037198	3.673852	0.933686
H	-1.063638	4.916106	-0.869337
H	0.490427	5.710554	-0.669986
H	0.242446	4.595140	-2.015379
H	1.884852	1.669986	-2.140793
H	1.875919	3.432597	-2.299322
H	3.190992	2.627478	-1.444629
H	7.116258	-3.052353	-1.217824
H	7.105288	-1.423757	-1.904950
H	7.900210	-1.723363	-0.354453
H	-2.019911	-2.216139	3.036246
H	-4.257182	-2.946806	1.156311
H	-5.342103	-2.129522	-0.529412
H	-3.570827	-3.351847	-1.703610
H	-4.063789	-1.916268	-2.613140
H	-4.029324	-0.082015	1.363793
H	-4.699117	2.205561	1.626166
H	-5.121116	2.259814	-0.081538
H	-1.296857	2.354183	-0.252046
H	-2.265815	-4.262013	-0.156345
H	-0.896889	-3.991970	0.939499
H	-2.495876	-4.390346	1.594035

ωB97X Energy = -1950.62666446 a.u.

(3*R*,4*S*,5*R*,8*R*,10*S*,19*R*,24*S*)-**1**, Conf D

C	6.021139	-1.379291	-1.360234
C	7.092208	-0.944405	-0.359391
C	6.671462	0.346355	0.343224
C	5.285660	0.218582	0.974922
C	4.240702	-0.128810	-0.093247
C	4.626773	-1.460685	-0.740248
C	4.898000	1.435038	1.765227
C	3.654318	1.886357	1.853057
C	2.463202	1.249022	1.184323
C	2.849510	-0.122674	0.544911
C	1.780815	2.239769	0.243809
C	0.520670	2.598503	0.492121
C	-0.339208	3.550697	-0.281207
C	2.591607	2.762347	-0.911338
C	1.729865	-0.536130	-0.385346
C	8.452980	-0.796614	-1.027840
C	0.450319	-0.989106	0.207625
O	1.815468	-0.465624	-1.598190
C	0.213796	-1.474537	1.602621
N	-1.138989	-1.623314	1.736185
C	-1.826314	-1.552457	0.465607
C	-0.739826	-0.944914	-0.416628
O	1.029257	-1.690142	2.480810
N	-3.012541	-0.713925	0.582275
C	-3.644763	-0.441046	-0.703673
C	-2.790955	0.381972	-1.659283
S	-1.129558	-0.321495	-1.991791
C	-5.004227	0.222510	-0.507521
N	-5.579176	0.044127	0.682873
C	-6.814409	0.699024	1.072131
C	-8.064160	0.131092	0.402598
O	-8.065464	0.128117	-0.926767
O	-5.547269	0.842509	-1.426738
O	-9.016375	-0.260824	1.027841
C	-2.186201	-2.957105	-0.027496
H	4.283258	0.645686	-0.866696
H	5.330713	-0.628328	1.677075
H	6.295211	-2.344575	-1.797188
H	6.000337	-0.656665	-2.186699
H	7.167833	-1.725972	0.408768
H	6.663166	1.170471	-0.383066
H	7.407432	0.608864	1.110875
H	4.606691	-2.247644	0.024854
H	3.901426	-1.739068	-1.505782
H	5.692722	1.949384	2.301832
H	3.439284	2.776875	2.438891
H	1.735622	1.029284	1.972576
H	2.851732	-0.843422	1.369158
H	0.035020	2.149539	1.357916
H	0.175004	4.004633	-1.127058
H	-1.223084	3.036145	-0.670096
H	-0.705118	4.352133	0.366124
H	2.678660	2.003140	-1.693740
H	2.150176	3.652060	-1.357419
H	3.604757	3.013045	-0.588723
H	8.764931	-1.730470	-1.502494
H	8.418961	-0.023091	-1.801460
H	9.221406	-0.512565	-0.304283
H	-1.508733	-2.183277	2.489646
H	-2.738782	0.158181	1.028351
H	-3.878424	-1.399005	-1.175353
H	-3.271370	0.459456	-2.632313
H	-2.650237	1.390682	-1.267419
H	-5.066791	-0.500108	1.362765
H	-6.754902	1.763979	0.828375
H	-6.935688	0.595070	2.145987
H	-7.217455	0.498562	-1.272693
H	-2.530131	-2.957949	-1.060930
H	-1.306472	-3.597685	0.031360
H	-2.972004	-3.364917	0.610171

ωB97X Energy = -1950.62652579 a.u.

(3*R*,4*S*,5*R*,8*R*,10*S*,19*R*,24*S*)-**1**, Conf E

C	6.080724	-1.238494	-1.093210
C	7.078201	-0.602604	-0.124474
C	6.540434	0.732087	0.391424
C	5.143635	0.582666	0.993508
C	4.166412	0.018494	-0.045632
C	4.673649	-1.349153	-0.507757
C	4.637409	1.860454	1.599405
C	3.360963	2.220089	1.591480
C	2.245308	1.404295	0.989542
C	2.758877	-0.005413	0.554986
C	1.517628	2.195437	-0.094891
C	0.221637	2.466212	0.065558
C	-0.686000	3.218893	-0.858675
C	2.323832	2.628038	-1.289907
C	1.705407	-0.634383	-0.330140
C	8.451423	-0.440462	-0.763359
C	0.449086	-1.105590	0.297639
O	1.821610	-0.725083	-1.539087
C	0.210116	-1.404268	1.744106
N	-1.129949	-1.642603	1.873679
C	-1.783057	-1.810802	0.594424
C	-0.721968	-1.249829	-0.347562
O	1.013662	-1.424565	2.658811
N	-3.032701	-1.061286	0.567570
C	-3.649327	-1.032218	-0.754449
C	-2.836214	-0.293937	-1.808240
S	-1.113116	-0.893358	-2.002677
C	-5.061487	-0.459746	-0.675149
N	-5.659262	-0.538742	0.515261
C	-7.071633	-0.264806	0.708992
C	-7.434778	1.217744	0.658650
O	-7.096568	1.865274	-0.451933
O	-5.614376	0.015744	-1.670953
O	-8.025549	1.773908	1.549142
C	-2.019047	-3.295627	0.303705
H	4.175271	0.689933	-0.911233
H	5.223221	-0.157987	1.804377
H	6.440616	-2.227191	-1.394859
H	6.040163	-0.629290	-2.005896
H	7.176006	-1.273276	0.739949
H	6.503677	1.455152	-0.434701
H	7.225189	1.143790	1.140926
H	4.681982	-2.035479	0.349141
H	3.999248	-1.773914	-1.252374
H	5.370883	2.504322	2.080529
H	3.059162	3.161677	2.043961
H	1.511721	1.234843	1.784711
H	2.792268	-0.602849	1.471972
H	-0.258916	2.092700	0.969307
H	-1.470684	2.559226	-1.242101
H	-1.190818	4.030424	-0.328338
H	-0.168166	3.644280	-1.717121
H	1.827022	3.409924	-1.862040
H	3.301210	3.005113	-0.980395
H	2.499150	1.782313	-1.960994
H	8.842601	-1.399863	-1.111683
H	8.397274	0.231673	-1.625574
H	9.170939	-0.019881	-0.056067
H	-1.478182	-2.113954	2.695006
H	-2.836674	-0.113745	0.881044
H	-3.798665	-2.065574	-1.078942
H	-3.291836	-0.404060	-2.790094
H	-2.791013	0.770399	-1.571253
H	-5.118391	-0.929438	1.274224
H	-7.365973	-0.647303	1.681645
H	-7.653574	-0.781077	-0.060589
H	-6.635794	1.255836	-1.078072
H	-2.325699	-3.471819	-0.726290
H	-1.095156	-3.847481	0.475221
H	-2.792440	-3.669972	0.976169

ωB97X Energy = -1950.62652074 a.u.

(3*R*,4*S*,5*R*,8*R*,10*S*,19*R*,24*S*)-**1**, Conf F

C	-6.126742	-1.164258	0.893293
C	-7.060069	-0.297115	0.047978
C	-6.400991	1.047410	-0.257856
C	-5.024251	0.866300	-0.896500
C	-4.102798	0.068017	0.034594
C	-4.733127	-1.305022	0.282457
C	-4.407849	2.168075	-1.318564
C	-3.103589	2.403124	-1.286388
C	-2.063765	1.412048	-0.829314
C	-2.696953	0.007310	-0.568000
C	-1.247154	1.994196	0.321167
C	0.048974	2.241507	0.130923
C	1.019729	2.836167	1.109246
C	-1.972148	2.296241	1.604971
C	-1.708726	-0.810784	0.236846
C	-8.414956	-0.115892	0.720097
C	-0.451555	-1.236761	-0.418922
O	-1.871912	-1.079217	1.413359
C	-0.184500	-1.348859	-1.885573
N	1.155821	-1.577352	-2.016762
C	1.790114	-1.894442	-0.755539
C	0.702514	-1.474595	0.227593
O	-0.971876	-1.248281	-2.809795
N	3.021233	-1.128389	-0.609249
C	3.622192	-1.248912	0.716406
C	2.766732	-0.689376	1.845256
S	1.057040	-1.348648	1.924390
C	5.004356	-0.588467	0.744894
N	5.626028	-0.514645	-0.444543
C	6.902734	0.122906	-0.576857
C	6.803195	1.636838	-0.565200
O	8.019458	2.190402	-0.594145
O	5.505762	-0.202222	1.790979
O	5.782789	2.273068	-0.542073
C	2.053350	-3.398582	-0.649895
H	-4.056776	0.596833	0.993472

H	-5.172073	0.264309	-1.806475
H	-6.574195	-2.152322	1.039884
H	-6.034573	-0.711749	1.889514
H	-7.216057	-0.810792	-0.910369
H	-6.295964	1.622492	0.672226
H	-7.044018	1.635081	-0.922064
H	-4.801242	-1.841660	-0.672863
H	-4.102897	-1.902564	0.941993
H	-5.082151	2.938849	-1.686194
H	-2.716573	3.367744	-1.606074
H	-1.362642	1.285152	-1.660665
H	-2.768059	-0.472550	-1.550175
H	0.466889	2.002449	-0.846059
H	0.724228	2.680959	2.147101
H	2.011445	2.398488	0.972931
H	1.131722	3.912899	0.949019
H	-2.216517	1.374679	2.140830
H	-1.382270	2.927119	2.268278
H	-2.914691	2.810035	1.400531
H	-8.898249	-1.079161	0.902460
H	-8.301182	0.389252	1.684426
H	-9.086137	0.487220	0.103205
H	1.526215	-1.931154	-2.885714
H	2.809999	-0.152927	-0.805738
H	3.813717	-2.308630	0.903362
H	3.211585	-0.920899	2.810670
H	2.691251	0.395567	1.759195
H	5.106646	-0.799705	-1.261064
H	7.371937	-0.182111	-1.512224
H	7.564642	-0.177816	0.236747
H	7.921939	3.152973	-0.597034
H	2.358560	-3.693174	0.353284
H	1.141373	-3.942583	-0.895502
H	2.837820	-3.673682	-1.356555

ωB97X Energy = -1950.62636730 a.u.

(3*R*,4*S*,5*R*,8*R*,10*S*,19*R*,24*S*)-**1**, Conf G

C	6.096447	-1.297888	-0.869651
C	7.060609	-0.506979	0.015618
C	6.472986	0.863656	0.350966
C	5.075013	0.742473	0.956294
C	4.130698	0.028115	-0.018945
C	4.683221	-1.372772	-0.292662
C	4.524346	2.063572	1.409676
C	3.237952	2.379523	1.351726
C	2.155342	1.470677	0.828146
C	2.711760	0.037228	0.554210
C	1.425934	2.129446	-0.340449
C	0.129888	2.415441	-0.210349
C	-0.777282	3.073265	-1.204763
C	2.235379	2.436389	-1.571610
C	1.692309	-0.706068	-0.281575
C	8.434467	-0.381878	-0.630238
C	0.428822	-1.130444	0.363441
O	1.841773	-0.923354	-1.470728
C	0.166540	-1.296735	1.826485
N	-1.171479	-1.542809	1.953493
C	-1.801817	-1.831041	0.683752
C	-0.728145	-1.348525	-0.286743
O	0.953652	-1.219873	2.753128
N	-3.057676	-1.102635	0.570712
C	-3.655135	-1.188518	-0.758888
C	-2.821963	-0.551303	-1.862140
S	-1.093114	-1.155851	-1.974285
C	-5.064131	-0.586385	-0.758774
N	-5.693021	-0.613187	0.428723
C	-7.008818	-0.068112	0.588045
C	-7.009820	1.445547	0.687347
O	-8.260509	1.913532	0.748229
O	-5.578682	-0.164043	-1.784207
O	-6.034317	2.148293	0.716632
C	-2.017590	-3.338865	0.523467
H	4.140445	0.583925	-0.962979
H	5.168081	0.103682	1.848289
H	6.490062	-2.306077	-1.032608
H	6.052004	-0.816646	-1.855580
H	7.172352	-1.054948	0.961038
H	6.420761	1.472443	-0.562031
H	7.134731	1.392022	1.045879
H	4.696433	-1.939498	0.647464
H	4.034327	-1.912898	-0.983385
H	5.232710	2.778651	1.823085
H	2.901668	3.354737	1.695811
H	1.413720	1.366197	1.627161
H	2.737706	-0.460599	1.529104
H	-0.349890	2.143361	0.729263
H	-0.275178	3.340849	-2.133327
H	-1.610152	2.410801	-1.458285
H	-1.219031	3.979608	-0.782005
H	2.438659	1.521279	-2.134929
H	1.727596	3.132508	-2.237030
H	3.200016	2.871197	-1.299789
H	8.870326	-1.364458	-0.828394
H	8.363695	0.151179	-1.583608
H	9.125573	0.170440	0.011718
H	-1.532252	-1.935833	2.809585
H	-2.882201	-0.130331	0.811517
H	-3.806142	-2.245920	-0.990956
H	-3.262147	-0.752423	-2.836507
H	-2.782071	0.530521	-1.726736
H	-5.162626	-0.918922	1.230542
H	-7.467267	-0.470917	1.491270
H	-7.640889	-0.350350	-0.255478
H	-8.227267	2.877712	0.820647
H	-2.319287	-3.606968	-0.488125
H	-1.087763	-3.862506	0.745051
H	-2.789055	-3.663121	1.223635

ωB97X Energy = -1950.62634931 a.u.

(3*R*,4*S*,5*R*,8*R*,10*S*,19*R*,24*S*)-**1**, Conf H

C	6.000769	-1.488184	-1.320235
C	7.080038	-1.058930	-0.325835
C	6.688641	0.255128	0.350070
C	5.298867	0.173403	0.980730
C	4.248006	-0.170328	-0.082798
C	4.603494	-1.523499	-0.702401
C	4.939008	1.415102	1.744468
C	3.706476	1.898314	1.818971
C	2.501490	1.275152	1.162045
C	2.855892	-0.118045	0.551285
C	1.841884	2.260472	0.199918
C	0.588409	2.649095	0.436720
C	-0.251807	3.598426	-0.361310
C	2.664890	2.741319	-0.964801
C	1.728854	-0.522883	-0.374111
C	8.444874	-0.955803	-0.994471
C	0.437286	-0.931756	0.222753
O	1.820805	-0.479256	-1.588003
C	0.182163	-1.375359	1.627919
N	-1.173788	-1.491192	1.756847
C	-1.853382	-1.437011	0.480716
C	-0.748735	-0.875606	-0.409581
O	0.988039	-1.585710	2.516892
N	-3.024432	-0.576082	0.569774
C	-3.647007	-0.322560	-0.725925
C	-2.769255	0.455695	-1.696150
S	-1.116638	-0.281503	-2.000063
C	-4.998009	0.378837	-0.563365
N	-5.569994	0.247629	0.645782
C	-6.890943	0.742140	0.900926
C	-7.974258	-0.161406	0.343540
O	-9.182270	0.379618	0.531220
O	-5.514591	0.985657	-1.490784
O	-7.796475	-1.224233	-0.190253
C	-2.236223	-2.846734	0.019286
H	4.310543	0.587498	-0.871396
H	5.322078	-0.659846	1.700023
H	6.252282	-2.468421	-1.737014
H	5.999057	-0.782221	-2.161281
H	7.136094	-1.826846	0.457572
H	6.701375	1.064601	-0.392434
H	7.429148	0.515355	1.114142
H	4.562769	-2.294680	0.077849
H	3.873536	-1.799405	-1.464376
H	5.744881	1.921552	2.271899
H	3.512058	2.806410	2.384698
H	1.768623	1.088642	1.954041
H	2.838538	-0.822059	1.389788
H	0.092181	2.229117	1.311024
H	-0.596829	4.427092	0.262869
H	0.269860	4.014870	-1.221745
H	-1.148669	3.092894	-0.731950
H	2.736052	1.964514	-1.731433
H	2.242724	3.631144	-1.429049
H	3.682918	2.976806	-0.646081
H	8.734290	-1.904823	-1.453062
H	8.430514	-0.194669	-1.780880
H	9.219202	-0.678480	-0.274602
H	-1.561029	-2.020731	2.523296
H	-2.740707	0.301514	0.997922
H	-3.899191	-1.288151	-1.171827
H	-3.242343	0.516680	-2.673839
H	-2.613177	1.472163	-1.330619
H	-5.094801	-0.326122	1.326227
H	-7.016337	1.730697	0.456492
H	-7.046623	0.842560	1.975128
H	-9.850146	-0.223089	0.175298
H	-2.573542	-2.865290	-1.016109
H	-1.369696	-3.502789	0.100215
H	-3.034520	-3.223416	0.660646

ωB97X Energy = -1950.62631738 a.u.

(3*R*,4*S*,5*R*,8*R*,10*S*,19*R*,24*S*)-**1**, Conf I

C	-3.599869	-2.587359	0.714540
C	-4.690138	-2.554862	-0.357285
C	-4.997517	-1.112278	-0.758920
C	-3.732935	-0.366291	-1.182263
C	-2.711992	-0.352228	-0.037659
C	-2.353440	-1.797730	0.317551
C	-4.015672	1.014602	-1.699271
C	-3.187976	2.038309	-1.542000
C	-1.861307	1.970035	-0.830478
C	-1.506164	0.496152	-0.448571
C	-1.809426	2.965471	0.324448
C	-0.875852	3.916846	0.309275
C	-0.613030	4.970733	1.338775
C	-2.826360	2.797890	1.420795
C	-0.392289	0.526469	0.575448
C	-5.944684	-3.287525	0.099939
C	0.976270	0.891399	0.128888
O	-0.591074	0.297743	1.757045
C	1.863419	1.781167	0.949815
N	3.066010	1.784698	0.321722
C	3.069766	1.080119	-0.948770
C	1.680718	0.445070	-0.923650
O	1.584718	2.385642	1.967726
N	4.148851	0.096808	-1.006520
C	3.853610	-1.247995	-1.475032
C	2.867366	-1.311301	-2.635309
S	1.216507	-0.687859	-2.163138
C	3.414852	-2.189793	-0.338882
N	3.665697	-1.738040	0.902229
C	3.201366	-2.432332	2.082520
C	1.797877	-1.965754	2.434779
O	1.778854	-0.668221	2.729821
O	2.911381	-3.277349	-0.582552
O	0.816099	-2.667024	2.411198
C	3.161021	2.082760	-2.103838
H	-3.189845	0.107551	0.834667
H	-3.287632	-0.940132	-2.009929
H	-3.331277	-3.625068	0.934918

H	-4.007971	-2.166110	1.642785
H	-4.297503	-3.065415	-1.247060
H	-5.462846	-0.588222	0.086916
H	-5.725843	-1.100976	-1.576925
H	-1.880648	-2.268470	-0.554774
H	-1.624302	-1.829675	1.128747
H	-4.944866	1.157672	-2.246758
H	-3.449310	3.015171	-1.941523
H	-1.097465	2.296959	-1.543815
H	-1.098156	0.045883	-1.360145
H	-0.186880	3.926470	-0.535043
H	-0.526850	5.954858	0.871434
H	-1.386231	5.023274	2.104431
H	0.338431	4.762774	1.837167
H	-2.561703	1.956490	2.068052
H	-2.905840	3.684969	2.047171
H	-3.813566	2.587479	1.003060
H	-5.725844	-4.328640	0.350581
H	-6.366576	-2.810495	0.990197
H	-6.712544	-3.282427	-0.677900
H	3.796967	2.427589	0.581676
H	4.938760	0.479732	-1.505112
H	4.795259	-1.666781	-1.839497
H	3.249562	-0.749027	-3.485642
H	2.702639	-2.343660	-2.935811
H	4.043319	-0.803719	0.978122
H	3.875959	-2.224576	2.912923
H	3.177427	-3.502094	1.890751
H	0.859636	-0.320625	2.682357
H	3.073319	1.575333	-3.064960
H	2.371069	2.830395	-2.023319
H	4.127837	2.589234	-2.064532

ωB97X Energy = -1950.62544420 a.u.

(3*R*,4*S*,5*R*,8*R*,10*S*,19*S*,24*R*)-**1**, Conf A

C	-3.192722	3.222616	-1.095125
C	-4.416814	3.524824	-0.230638
C	-5.123973	2.226690	0.158152
C	-4.164927	1.238187	0.820662
C	-2.995030	0.909869	-0.114833
C	-2.252152	2.203817	-0.453781
C	-4.858473	-0.005934	1.297036
C	-4.288818	-1.203525	1.299763
C	-2.876579	-1.484752	0.855184
C	-2.113160	-0.157078	0.539791
C	-2.847265	-2.531337	-0.255024
C	-2.163246	-3.658968	-0.055740
C	-1.973800	-4.806770	-1.000301
C	-3.597345	-2.215024	-1.520685
C	-0.876286	-0.503704	-0.261953
C	-5.366674	4.491167	-0.926461
C	0.306492	-1.050978	0.428146
O	-0.835421	-0.383839	-1.476391
C	0.495076	-1.285318	1.889762
N	1.770641	-1.734592	2.040512
C	2.422014	-2.044191	0.778311
C	1.447191	-1.405756	-0.203079
O	-0.297264	-1.108559	2.800511
N	3.759395	-1.464789	0.720256
C	4.228718	-0.891924	-0.531423
C	3.629575	-1.512253	-1.789028
S	1.828377	-1.240865	-1.879282
C	4.054838	0.630327	-0.564774
N	3.649681	1.203480	0.567473
C	3.309029	2.609762	0.668680
C	4.512099	3.550390	0.639684
O	5.309142	3.446293	-0.418534
O	4.320626	1.273531	-1.586542
O	4.719354	4.369167	1.499090
C	2.438511	-3.566065	0.573616
H	-3.412867	0.506688	-1.044370
H	-3.746153	1.743708	1.704584
H	-2.653416	4.151108	-1.307168
H	-3.535149	2.831646	-2.062416
H	-4.062157	3.996085	0.696042
H	-5.557852	1.764940	-0.739273
H	-5.956833	2.444702	0.835564
H	-1.826401	2.622266	0.467638
H	-1.418736	2.000544	-1.128238
H	-5.874959	0.104913	1.669281
H	-4.846557	-2.068101	1.652396
H	-2.358359	-1.931026	1.708095
H	-1.777309	0.227118	1.508062
H	-1.660670	-3.774470	0.904242
H	-2.338353	-5.737063	-0.556063
H	-2.480369	-4.664601	-1.954026
H	-0.909566	-4.953561	-1.207096
H	-3.043129	-1.489061	-2.122349
H	-3.767011	-3.099464	-2.132875
H	-4.568510	-1.770806	-1.290989
H	-4.863683	5.428344	-1.178284
H	-5.746441	4.054645	-1.855638
H	-6.225456	4.728922	-0.293356
H	2.065909	-2.162618	2.904100
H	4.434451	-2.118731	1.088500
H	5.309133	-1.049624	-0.577594
H	4.031323	-1.020697	-2.673041
H	3.869058	-2.572414	-1.842896
H	3.509142	0.586916	1.357370
H	2.781070	2.770853	1.603649
H	2.647166	2.883980	-0.158476
H	4.982843	2.737454	-1.026676
H	1.429515	-3.972101	0.658328
H	2.845139	-3.831125	-0.401690
H	3.063961	-4.021382	1.344756

ωB97X Energy = -1950.62863172 a.u.

(3*R*,4*S*,5*R*,8*R*,10*S*,19*S*,24*R*)-**1**, Conf B

C	3.601737	-2.510693	-1.058055

C	4.860054	-2.501004	-0.189975
C	5.239815	-1.065913	0.173205
C	4.075386	-0.320550	0.824992
C	2.862955	-0.288829	-0.113267
C	2.447763	-1.725818	-0.436278
C	4.458984	1.059217	1.277110
C	3.627627	2.092088	1.254773
C	2.190358	2.029743	0.806254
C	1.753893	0.555111	0.522182
C	1.922427	3.017924	-0.325237
C	0.995498	3.960175	-0.145868
C	0.545456	5.014115	-1.111228
C	2.727255	2.859132	-1.586651
C	0.472645	0.587614	-0.286433
C	6.011361	-3.227791	-0.872937
C	-0.805500	0.876847	0.385473
O	0.469643	0.417916	-1.496258
C	-1.046717	1.131859	1.834354
N	-2.391551	1.278210	1.975620
C	-3.097293	1.355717	0.705499
C	-1.998122	0.904829	-0.249295
O	-0.236906	1.186612	2.746502
N	-4.276032	0.498578	0.699763
C	-4.543355	-0.318049	-0.474714
C	-4.133549	0.322323	-1.796028
S	-2.326427	0.548415	-1.904732
C	-3.953500	-1.731888	-0.350927
N	-3.497778	-2.062306	0.866982
C	-2.948150	-3.362380	1.127859
C	-1.572950	-3.618494	0.540391
O	-0.916842	-2.510723	0.191487
O	-3.945430	-2.503155	-1.301794
O	-1.107873	-4.725169	0.433166
C	-3.457830	2.817582	0.404117
H	3.177656	0.186371	-1.049032
H	3.786701	-0.895642	1.718620
H	3.292906	-3.542749	-1.250428
H	3.844698	-2.069029	-2.033075
H	4.625619	-3.026197	0.745811
H	5.549968	-0.530191	-0.734202
H	6.101610	-1.068753	0.849384
H	2.125663	-2.218187	0.492226
H	1.604154	-1.720775	-1.131077
H	5.471619	1.194123	1.651715
H	3.969156	3.068941	1.589581
H	1.580394	2.360984	1.650338
H	1.513422	0.124755	1.499699
H	0.477333	3.973374	0.812677
H	1.086877	4.988641	-2.056086
H	-0.518764	4.891737	-1.333783
H	0.663242	6.010756	-0.677260
H	2.682148	3.743165	-2.221052
H	3.776416	2.664755	-1.351828
H	2.362663	2.007935	-2.168762
H	5.747723	-4.264129	-1.099116
H	6.271497	-2.734843	-1.814859
H	6.903995	-3.237121	-0.242149
H	-2.783297	1.660696	2.822026
H	-5.092689	1.036193	0.950993
H	-5.624713	-0.472282	-0.514124
H	-4.380447	-0.338653	-2.624580
H	-4.652554	1.269152	-1.937316
H	-3.531912	-1.352407	1.584061
H	-3.603030	-4.143703	0.740477
H	-2.865018	-3.505428	2.205751
H	-0.048112	-2.769085	-0.151992
H	-2.568404	3.447571	0.451056
H	-3.908562	2.913966	-0.583643
H	-4.175806	3.169004	1.148402

ωB97X Energy = -1950.62774728 a.u.

(3*R*,4*S*,5*R*,8*R*,10*S*,19*S*,24*R*)-**1**, Conf C

C	3.475134	-2.357327	-1.334173
C	4.745656	-2.407776	-0.484434
C	5.135403	-1.002548	-0.025734
C	3.979077	-0.303160	0.688027
C	2.760439	-0.203869	-0.236261
C	2.332079	-1.615668	-0.643016
C	4.365954	1.036608	1.244924
C	3.532902	2.066102	1.315587
C	2.094240	2.041964	0.866284
C	1.657794	0.596666	0.461165
C	1.831664	3.124879	-0.175992
C	0.913641	4.056421	0.086134
C	0.478336	5.199476	-0.779718
C	2.639394	3.070731	-1.444262
C	0.380745	0.692090	-0.348651
C	5.886553	-3.090087	-1.227987
C	-0.911366	0.839468	0.342450
O	0.390466	0.676214	-1.569809
C	-1.163737	0.914592	1.810694
N	-2.512305	0.948656	1.961698
C	-3.236059	1.074085	0.707117
C	-2.105027	0.845610	-0.289267
O	-0.352874	0.931244	2.724066
N	-4.301110	0.081381	0.607547
C	-4.493139	-0.616067	-0.655378
C	-4.183911	0.220595	-1.892286
S	-2.414062	0.655730	-1.977499
C	-3.727629	-1.950387	-0.715455
N	-3.173147	-2.336909	0.444117
C	-2.400935	-3.542171	0.571538
C	-1.022987	-3.345814	1.165080
O	-0.863788	-2.161336	1.761630
O	-3.673174	-2.593929	-1.755372
O	-0.169408	-4.195267	1.138836
C	-3.772979	2.504388	0.562829
H	3.068553	0.330625	-1.142053
H	3.695050	-0.943717	1.537741
H	3.162668	-3.374595	-1.589885
H	3.705736	-1.852765	-2.281566

H	4.520511	-2.995241	0.416105
H	5.439055	-0.405146	-0.896185
H	6.003927	-1.056025	0.639769
H	2.026703	-2.170108	0.255208
H	1.465006	-1.577971	-1.305442
H	5.380890	1.142379	1.622857
H	3.875772	3.011696	1.729404
H	1.482840	2.301426	1.734966
H	1.416025	0.094008	1.402539
H	0.394925	3.988828	1.041991
H	0.612933	6.151586	-0.259211
H	1.017652	5.250721	-1.724651
H	-0.587851	5.114881	-1.009175
H	2.586828	4.000582	-2.008479
H	3.689829	2.868439	-1.222257
H	2.282664	2.262956	-2.089350
H	5.616524	-4.108427	-1.519224
H	6.137332	-2.537030	-2.138725
H	6.786954	-3.143654	-0.610535
H	-2.935658	1.173232	2.848069
H	-5.171627	0.479285	0.928380
H	-5.548578	-0.894326	-0.708224
H	-4.370335	-0.361022	-2.792824
H	-4.807813	1.112397	-1.916285
H	-3.311196	-1.724501	1.237021
H	-2.283039	-3.986757	-0.416027
H	-2.908036	-4.278430	1.202385
H	0.017706	-2.130693	2.160948
H	-2.965648	3.228435	0.679180
H	-4.242816	2.651577	-0.409423
H	-4.519769	2.685584	1.339310

ωB97X Energy = -1950.62662502 a.u.

(3*R*,4*S*,5*R*,8*R*,10*S*,19*S*,24*R*)-**1**, Conf D

C	4.281234	-2.321957	-0.649885
C	5.547038	-1.839956	0.059220
C	5.570574	-0.313049	0.124719
C	4.292328	0.242959	0.751191
C	3.061736	-0.201111	-0.049948
C	3.004613	-1.730343	-0.054173
C	4.335504	1.733413	0.928190
C	3.270082	2.515756	0.821591
C	1.875669	2.030158	0.516287
C	1.818494	0.467258	0.542771
C	1.342377	2.691708	-0.752657
C	0.276021	3.487853	-0.662641
C	-0.410436	4.249797	-1.754305
C	2.083846	2.424125	-2.034956
C	0.511224	0.042645	-0.080825
C	6.802622	-2.389660	-0.605036
C	-0.726917	0.263075	0.706421
O	0.447786	-0.411387	-1.214386
C	-0.838594	0.122317	2.189905
N	-2.144679	0.344359	2.492837
C	-2.902340	0.891905	1.378155
C	-1.933273	0.630544	0.228102
O	0.034230	-0.168573	2.990748
N	-4.179938	0.206078	1.220317
C	-4.669182	-0.087482	-0.119839
C	-4.229548	0.913131	-1.187148
S	-2.416573	0.906955	-1.410028
C	-4.308683	-1.510994	-0.583264
N	-3.664250	-2.261062	0.330461
C	-2.942351	-3.467647	-0.012462
C	-1.508250	-3.057088	-0.308721
O	-1.409312	-2.444643	-1.484549
O	-4.602512	-1.891753	-1.706138
O	-0.590916	-3.186506	0.466364
C	-3.077020	2.404611	1.576296
H	3.195268	0.135544	-1.083749
H	4.207883	-0.204866	1.753471
H	4.236138	-3.415058	-0.619926
H	4.344045	-2.041007	-1.709543
H	5.511536	-2.211836	1.092174
H	5.685440	0.095178	-0.888667
H	6.440771	0.021342	0.699987
H	2.876671	-2.085148	0.976848
H	2.140188	-2.082714	-0.618687
H	5.299092	2.175064	1.173672
H	3.369013	3.589844	0.958389
H	1.231170	2.377702	1.330946
H	1.758020	0.188892	1.599599
H	-0.171564	3.610985	0.323111
H	0.055561	4.115132	-2.729568
H	-1.453023	3.928904	-1.836155
H	-0.423761	5.319400	-1.527614
H	1.812449	3.127322	-2.820437
H	1.875784	1.415400	-2.401964
H	3.162443	2.495589	-1.876330
H	6.792761	-3.482409	-0.627486
H	6.878056	-2.034407	-1.637648
H	7.704114	-2.070978	-0.075402
H	-2.434140	0.485329	3.448110
H	-4.890240	0.673362	1.764642
H	-5.760179	-0.056004	-0.081949
H	-4.629205	0.620715	-2.155990
H	-4.586346	1.912936	-0.947725
H	-3.504127	-1.824235	1.228616
H	-3.402455	-3.919130	-0.890091
H	-2.956717	-4.166806	0.820155
H	-0.603591	-1.886352	-1.510769
H	-2.109711	2.882229	1.737261
H	-3.558005	2.865250	0.713948
H	-3.700932	2.579004	2.455675

ωB97X Energy = -1950.62503289 a.u.

(3*R*,4*S*,5*R*,8*R*,10*S*,19*S*,24*R*)-**1**, Conf E

C	3.850924	3.378840	0.853565
C	5.018226	3.663758	-0.092293
C	5.722246	2.361116	-0.472387

C	4.742387	1.334854	-1.040800
C	3.636219	1.027992	-0.024741
C	2.892072	2.322828	0.306591
C	5.425315	0.081952	-1.509441
C	4.877276	-1.122863	-1.425543
C	3.501735	-1.407891	-0.878879
C	2.734369	-0.080183	-0.577362
C	3.566182	-2.407996	0.271619
C	2.903363	-3.559704	0.155005
C	2.812018	-4.680310	1.145998
C	4.387647	-2.027454	1.473503
C	1.555592	-0.389367	0.323742
C	5.992009	4.669044	0.508805
C	0.276401	-0.839264	-0.267875
O	1.625826	-0.285007	1.535941
C	0.008637	-1.234940	-1.685513
N	-1.342346	-1.419461	-1.788111
C	-1.992139	-1.449687	-0.497492
C	-0.899491	-0.858316	0.389295
O	0.798891	-1.356955	-2.603598
N	-3.210637	-0.652424	-0.533528
C	-3.814547	-0.472180	0.782380
C	-2.959686	0.323257	1.758440
S	-1.265300	-0.337003	2.004688
C	-5.200004	0.153861	0.655175
N	-5.806355	0.001638	-0.523767
C	-7.207163	0.317372	-0.737505
C	-7.509921	1.812717	-0.807818
O	-7.140361	2.534791	0.245054
O	-5.726598	0.736020	1.607683
O	-8.083418	2.317272	-1.739507
C	-2.283705	-2.893333	-0.076791
H	4.114478	0.668548	0.893264
H	4.264973	1.800410	-1.917001
H	3.309864	4.307385	1.060705
H	4.254588	3.030487	1.813402
H	4.602901	4.094054	-1.013715
H	6.213433	1.939688	0.415191
H	6.511524	2.564254	-1.204348
H	2.407846	2.699219	-0.603878
H	2.101636	2.135013	1.035895
H	6.413269	0.191994	-1.952018
H	5.426211	-1.992940	-1.778503
H	2.937219	-1.898478	-1.675389
H	2.336023	0.257676	-1.539445
H	2.345559	-3.721997	-0.766763
H	3.360710	-4.483789	2.066201
H	1.767858	-4.864699	1.413681
H	3.194066	-5.609441	0.713968
H	5.338358	-1.586662	1.164427
H	3.861676	-1.278586	2.072080
H	4.604249	-2.880554	2.114742
H	5.489897	5.608193	0.754947
H	6.433278	4.273559	1.429069
H	6.807078	4.893953	-0.183870
H	-1.721224	-1.934796	-2.568180
H	-2.980327	0.251310	-0.938453
H	-4.007164	-1.462570	1.203647
H	-2.861174	1.356482	1.422945
H	-3.412525	0.327614	2.747692
H	-5.288912	-0.480623	-1.245434
H	-7.806341	-0.110050	0.072236
H	-7.521478	-0.130372	-1.675370
H	-6.699309	1.960945	0.917601
H	-1.379095	-3.490953	-0.189266
H	-2.604224	-2.965899	0.961564
H	-3.065269	-3.298188	-0.721435

ωB97X Energy = -1950.62478648 a.u.

(3*R*,4*S*,5*R*,8*R*,10*S*,19*S*,24*S*)-**1**, Conf A

C	5.359247	2.540734	0.582745
C	6.590077	2.083069	-0.200217
C	6.619832	0.557813	-0.295214
C	5.316719	0.001818	-0.868910
C	4.122382	0.425619	-0.005467
C	4.058849	1.953641	0.036133
C	5.360914	-1.487065	-1.060157
C	4.304313	-2.274680	-0.911274
C	2.919944	-1.796841	-0.555688
C	2.851485	-0.234306	-0.546027
C	2.410283	-2.472887	0.714131
C	1.280339	-3.178978	0.655491
C	0.573754	-3.892398	1.767566
C	3.219621	-2.284536	1.968782
C	1.587132	0.172874	0.180211
C	7.874865	2.629504	0.408654
C	0.288386	0.022622	-0.498030
O	1.596201	0.574288	1.333906
C	0.023303	-0.285327	-1.929805
N	-1.328900	-0.151530	-2.111580
C	-2.051554	-0.053162	-0.860943
C	-0.904309	0.214578	0.106880
O	0.811765	-0.575137	-2.811861
N	-2.976498	1.074310	-0.924080
C	-3.577891	1.534733	0.328855
C	-3.028560	0.917376	1.625060
S	-1.215146	0.816161	1.690429
C	-5.097770	1.355857	0.330928
N	-5.644844	0.913787	-0.799230
C	-7.076814	0.883993	-1.028873
C	-7.807589	-0.221617	-0.269846
O	-7.649187	-0.226428	1.049696
O	-5.759568	1.620961	1.341539
O	-8.518138	-1.026931	-0.816090
C	-2.766184	-1.375193	-0.576721
H	4.303539	0.067194	1.014229
H	5.183552	0.460248	-1.861158
H	5.306993	3.634040	0.581073
H	5.476306	2.235162	1.630871
H	6.498186	2.474413	-1.222538

H	6.785277	0.133117	0.704371
H	7.463702	0.239627	-0.917148
H	3.881706	2.331206	-0.979486
H	3.220745	2.283644	0.651669
H	6.315703	-1.921870	-1.348907
H	4.404194	-3.347669	-1.057906
H	2.255119	-2.121762	-1.361234
H	2.731701	0.061559	-1.593066
H	0.781195	-3.241262	-0.311119
H	0.422759	-4.946175	1.518651
H	1.106159	-3.840306	2.716424
H	-0.419911	-3.459928	1.920104
H	4.286103	-2.394452	1.759126
H	3.070692	-1.279853	2.374775
H	2.952742	-3.001615	2.743636
H	7.858357	3.721453	0.454886
H	8.006855	2.253276	1.428056
H	8.749582	2.329320	-0.174115
H	-1.762262	-0.576251	-2.917589
H	-2.503836	1.839148	-1.386576
H	-3.427871	2.615658	0.393522
H	-3.303760	1.556451	2.461322
H	-3.456714	-0.062143	1.824410
H	-5.001483	0.725039	-1.557321
H	-7.253660	0.734576	-2.089521
H	-7.515259	1.841858	-0.732240
H	-7.050532	0.510340	1.327797
H	-2.033907	-2.182275	-0.527875
H	-3.327313	-1.366707	0.354216
H	-3.463881	-1.577085	-1.391071

ωB97X Energy = -1950.62859651 a.u.

(3*R*,4*S*,5*R*,8*R*,10*S*,19*S*,24*S*)-**1**, Conf B

C	5.279301	-2.717261	-0.630510
C	6.520409	-2.348494	0.182692
C	6.619263	-0.831349	0.339656
C	5.336086	-0.239200	0.922038
C	4.135731	-0.570903	0.027046
C	4.000773	-2.091468	-0.075131
C	5.447809	1.236007	1.178151
C	4.432297	2.079208	1.050705
C	3.033161	1.684228	0.653855
C	2.889416	0.128237	0.574692
C	2.581071	2.439190	-0.593519
C	1.488895	3.201155	-0.521960
C	0.836406	3.993253	-1.613543
C	3.402009	2.261150	-1.842107
C	1.619299	-0.180224	-0.188723
C	7.785447	-2.928991	-0.436116
C	0.319436	0.032118	0.470480
O	1.626413	-0.537938	-1.356573
C	0.041218	0.263406	1.913730
N	-1.322810	0.240888	2.053944
C	-2.012256	0.307072	0.782118
C	-0.866425	-0.001379	-0.174847
O	0.826480	0.417786	2.831984
N	-3.043111	-0.724550	0.730706
C	-3.660132	-0.999771	-0.567571
C	-2.997165	-0.360118	-1.800233
S	-1.182718	-0.461524	-1.804240
C	-5.142619	-0.620470	-0.588984
N	-5.669687	-0.216890	0.564656
C	-7.014304	0.314133	0.685813
C	-8.119099	-0.732700	0.555495
O	-8.112391	-1.455545	-0.559172
O	-5.799347	-0.721010	-1.632399
O	-8.976308	-0.879541	1.389612
C	-2.587914	1.710603	0.585092
H	4.349547	-0.182851	-0.975287
H	5.168737	-0.731972	1.892401
H	5.176444	-3.806108	-0.673454
H	5.423334	-2.375950	-1.664136
H	6.399391	-2.776014	1.187356
H	6.815310	-0.375455	-0.640422
H	7.469544	-0.577676	0.982133
H	3.794001	-2.499029	0.923091
H	3.155880	-2.357867	-0.711854
H	6.417531	1.611432	1.498814
H	4.580147	3.139001	1.244918
H	2.369900	2.006990	1.461823
H	2.734725	-0.205773	1.605625
H	0.978192	3.250383	0.439422
H	0.734393	5.042652	-1.324509
H	1.379504	3.951514	-2.556830
H	-0.175349	3.617636	-1.795951
H	3.217030	1.279468	-2.287396
H	3.179719	3.016747	-2.594042
H	4.468667	2.316208	-1.612321
H	7.719326	-4.016323	-0.525905
H	7.944482	-2.519409	-1.438661
H	8.666904	-2.692360	0.165428
H	-1.738811	0.648034	2.878068
H	-2.655168	-1.573636	1.119369
H	-3.646165	-2.082209	-0.715388
H	-3.309842	-0.903099	-2.689457
H	-3.302743	0.673806	-1.939752
H	-5.039818	-0.197150	1.356156
H	-7.178386	1.072676	-0.085890
H	-7.113141	0.783884	1.659715
H	-7.351088	-1.194712	-1.135176
H	-1.780942	2.444113	0.615619
H	-3.119572	1.828887	-0.355773
H	-3.286389	1.917810	1.397433

ωB97X Energy = -1950.62852052 a.u.

(3*R*,4*S*,5*R*,8*R*,10*S*,19*S*,24*S*)-**1**, Conf C

C	-5.471683	-2.409386	0.534879
C	-6.660289	-1.891005	-0.275216
C	-6.609995	-0.366470	-0.373553
C	-5.267979	0.120467	-0.919246

C	-4.115761	-0.361573	-0.028985
C	-4.131387	-1.890725	0.016087
C	-5.231885	1.609045	-1.116191
C	-4.139688	2.342033	-0.947317
C	-2.790442	1.794922	-0.558604
C	-2.802492	0.231185	-0.545356
C	-2.278840	2.449987	0.721305
C	-1.122147	3.112910	0.684591
C	-0.413263	3.804642	1.808843
C	-3.120262	2.296414	1.959649
C	-1.571282	-0.235427	0.201801
C	-7.984137	-2.370025	0.306131
C	-0.257475	-0.139588	-0.456036
O	-1.617871	-0.636450	1.354848
C	0.039553	0.133412	-1.888429
N	1.387551	-0.055052	-2.047919
C	2.089631	-0.152406	-0.784600
C	0.919555	-0.359936	0.170742
O	-0.725005	0.436978	-2.787553
N	2.971065	-1.313426	-0.801619
C	3.584512	-1.710401	0.468166
C	3.000329	-1.081961	1.745858
S	1.187106	-0.952885	1.764631
C	5.102594	-1.473709	0.481325
N	5.647608	-1.170873	-0.705868
C	7.044410	-0.886556	-0.840461
C	7.401620	0.524234	-0.412342
O	8.721380	0.721977	-0.495920
O	5.749341	-1.598728	1.513869
O	6.624389	1.367290	-0.049287
C	2.848664	1.148452	-0.519072
H	-4.300109	0.008348	0.985996
H	-5.136421	-0.347661	-1.907209
H	-5.476015	-3.503929	0.535611
H	-5.595539	-2.095700	1.579819
H	-6.566377	-2.289289	-1.294680
H	-6.775896	0.069085	0.621264
H	-7.422436	-0.007640	-1.014903
H	-3.951677	-2.279437	-0.994854
H	-3.324605	-2.261214	0.650194
H	-6.156757	2.090328	-1.427712
H	-4.180765	3.418063	-1.100035
H	-2.090890	2.083471	-1.348577
H	-2.678942	-0.071757	-1.589932
H	-0.601585	3.154071	-0.271773
H	-0.959257	3.761902	2.750406
H	0.567717	3.348237	1.972510
H	-0.234079	4.855555	1.566191
H	-2.844433	3.007670	2.736764
H	-4.177679	2.442644	1.727510
H	-3.014580	1.289093	2.372714
H	-8.024690	-3.461278	0.353820
H	-8.119157	-1.985338	1.321996
H	-8.829386	-2.026892	-0.296087
H	1.847250	0.337380	-2.855667
H	2.459147	-2.088741	-1.200666
H	3.469482	-2.793644	0.554483
H	3.242204	-1.716940	2.595262
H	3.433707	-0.106633	1.952847
H	5.005905	-1.029175	-1.472763
H	7.351660	-1.018234	-1.878155
H	7.630202	-1.578180	-0.232537
H	8.916873	1.629207	-0.222622
H	2.144741	1.981553	-0.495332
H	3.402459	1.139076	0.416215
H	3.563628	1.307863	-1.327824

ωB97X Energy = -1950.62806088 a.u.

(3*R*,4*S*,5*R*,8*R*,10*S*,19*S*,24*S*)-**1**, Conf D

C	5.250339	2.716591	0.756809
C	6.487146	2.410770	-0.088486
C	6.602888	0.906689	-0.334227
C	5.320696	0.332874	-0.936530
C	4.126578	0.596120	-0.011194
C	3.973741	2.106562	0.179980
C	5.447618	-1.123097	-1.280557
C	4.442825	-1.984212	-1.195709
C	3.042667	-1.630255	-0.764819
C	2.882070	-0.084515	-0.585896
C	2.611148	-2.472342	0.433103
C	1.533814	-3.249614	0.315544
C	0.910577	-4.138149	1.348392
C	3.440462	-2.365618	1.684102
C	1.619171	0.160553	0.212244
C	7.751160	2.971650	0.550204
C	0.312255	-0.031749	-0.437679
O	1.641519	0.449448	1.399021
C	0.014217	-0.187263	-1.886763
N	-1.351590	-0.176149	-2.005124
C	-2.021415	-0.320612	-0.728609
C	-0.864233	-0.049714	0.226488
O	0.787094	-0.279871	-2.824289
N	-3.064085	0.690624	-0.603848
C	-3.675391	0.872222	0.715403
C	-2.974584	0.188199	1.902118
S	-1.161696	0.317988	1.881759
C	-5.152693	0.454202	0.741999
N	-5.676205	0.093003	-0.438649
C	-7.066299	-0.225523	-0.573373
C	-7.953902	1.004443	-0.585434
O	-9.244262	0.654163	-0.602070
O	-5.788270	0.469460	1.788917
O	-7.574571	2.145534	-0.589888
C	-2.575631	-1.740573	-0.599774
H	4.356021	0.153102	0.964532
H	5.135909	0.879083	-1.874579
H	5.134556	3.799699	0.863471
H	5.409627	2.318125	1.767516
H	6.350634	2.893563	-1.065752
H	6.816055	0.397525	0.615560
H	7.448805	0.701952	-0.999511

H	3.750518	2.567400	-0.791117
H	3.132612	2.324147	0.839878
H	6.418811	-1.466960	-1.630845
H	4.600846	-3.028855	-1.453659
H	2.376711	-1.908058	-1.587009
H	2.709850	0.311873	-1.591737
H	1.016382	-3.240531	-0.643448
H	0.832735	-5.164042	0.978514
H	1.461133	-4.156357	2.288043
H	-0.108878	-3.804982	1.566475
H	4.505512	-2.406615	1.443805
H	3.258462	-1.410519	2.184844
H	3.223322	-3.161996	2.394218
H	7.673843	4.051912	0.697680
H	7.923131	2.511874	1.528584
H	8.629992	2.777167	-0.069943
H	-1.775170	-0.541414	-2.844755
H	-2.689513	1.568002	-0.938800
H	-3.689265	1.945528	0.919521
H	-3.278872	0.681371	2.822613
H	-3.260261	-0.856417	1.997978
H	-5.075252	0.177028	-1.245691
H	-7.390271	-0.865859	0.248769
H	-7.230469	-0.773074	-1.501648
H	-9.782857	1.457555	-0.623881
H	-1.760330	-2.460839	-0.682080
H	-3.092066	-1.914447	0.340848
H	-3.285377	-1.911795	-1.410737

ωB97X Energy = -1950.62773779 a.u.

(3*R*,4*S*,5*R*,8*R*,10*S*,19*S*,24*S*)-**1**, Conf E

C	-5.452297	-2.514710	-0.003557
C	-6.615965	-1.893169	-0.777133
C	-6.580314	-0.369469	-0.658905
C	-5.223605	0.196914	-1.076632
C	-4.107746	-0.393011	-0.206106
C	-4.099400	-1.913222	-0.381139
C	-5.199669	1.698256	-1.073854
C	-4.127785	2.411560	-0.756636
C	-2.791863	1.830168	-0.371687
C	-2.778119	0.277228	-0.558836
C	-2.357894	2.323937	1.005874
C	-1.215316	3.003521	1.112582
C	-0.579153	3.563177	2.348225
C	-3.257891	2.001580	2.167977
C	-1.590134	-0.270784	0.203580
C	-7.953867	-2.457588	-0.316844
C	-0.235849	-0.065208	-0.336053
O	-1.707361	-0.823109	1.286912
C	0.158586	0.385971	-1.698085
N	1.522654	0.270413	-1.764457
C	2.127017	0.051842	-0.466017
C	0.897127	-0.316834	0.355895
O	-0.547700	0.761720	-2.617247
N	3.071760	-1.055271	-0.547296
C	3.573984	-1.616776	0.709773
C	2.878542	-1.145874	1.998273
S	1.065497	-1.078356	1.890731
C	5.082920	-1.392961	0.892528
N	5.703108	-0.795080	-0.128333
C	7.114169	-0.526400	-0.116510
C	7.482312	0.304600	-1.318435
O	8.785510	0.580752	-1.337241
O	5.651131	-1.759847	1.915668
O	6.705329	0.674227	-2.162016
C	2.790450	1.344902	0.012398
H	-4.349561	-0.174837	0.840400
H	-5.043694	-0.134106	-2.111229
H	-5.440386	-3.597414	-0.163559
H	-5.623268	-2.359238	1.069873
H	-6.482320	-2.142455	-1.838512
H	-6.788889	-0.079185	0.379834
H	-7.372222	0.069743	-1.275372
H	-3.869400	-2.148408	-1.428533
H	-3.314569	-2.362964	0.228999
H	-6.115002	2.208629	-1.366690
H	-4.176661	3.497973	-0.770395
H	-2.057811	2.224515	-1.080585
H	-2.583008	0.111363	-1.623202
H	-0.649512	3.171599	0.196571
H	-0.399160	4.635806	2.237157
H	-1.176952	3.409990	3.245841
H	0.396558	3.096230	2.514669
H	-3.021868	2.594038	3.050634
H	-4.303352	2.183094	1.907840
H	-3.170063	0.945637	2.438625
H	-7.989620	-3.543034	-0.440657
H	-8.120675	-2.236146	0.742044
H	-8.783146	-2.026258	-0.883482
H	2.026326	0.769111	-2.482367
H	2.653009	-1.782148	-1.111648
H	3.454978	-2.701452	0.653608
H	3.085221	-1.862806	2.789754
H	3.257107	-0.185069	2.338730
H	5.147773	-0.539988	-0.935193
H	7.704074	-1.447432	-0.137816
H	7.404044	0.015378	0.787364
H	8.980533	1.110945	-2.122925
H	2.042715	2.136604	0.078815
H	3.272027	1.245280	0.981939
H	3.551442	1.634416	-0.714299

ωB97X Energy = -1950.62687325 a.u.

(3*R*,4*S*,5*R*,8*R*,10*S*,19*S*,24*S*)-**1**, Conf F

C	-4.933697	-2.667531	0.869295
C	-6.142323	-2.543808	-0.059568
C	-6.357469	-1.084128	-0.460688
C	-5.086765	-0.475400	-1.052830
C	-3.934789	-0.545193	-0.042782
C	-3.674775	-2.013322	0.300525

C	-5.295570	0.916830	-1.575045
C	-4.392389	1.882588	-1.473192
C	-3.043736	1.723006	-0.822483
C	-2.731645	0.203204	-0.621085
C	-2.913578	2.586541	0.434232
C	-3.939751	2.751419	1.268060
C	-3.986236	3.549476	2.535886
C	-1.573273	3.251647	0.605266
C	-1.463481	0.045004	0.184923
C	-7.393361	-3.141353	0.571303
C	-0.159874	0.129114	-0.496374
O	-1.475686	-0.123238	1.395057
C	0.113199	0.263230	-1.953683
N	1.464304	0.094566	-2.112176
C	2.183578	0.134489	-0.856256
C	1.030234	0.009993	0.133894
O	-0.668868	0.463071	-2.865552
N	3.080636	-1.014893	-0.776158
C	3.667061	-1.334864	0.526608
C	3.136899	-0.545718	1.734270
S	1.327758	-0.380593	1.783420
C	5.191431	-1.200305	0.514823
N	5.752249	-0.903834	-0.655223
C	7.184861	-0.936328	-0.881013
C	7.944873	0.225381	-0.244325
O	7.781557	0.384002	1.064879
O	5.843372	-1.366733	1.552646
O	8.681920	0.942188	-0.872752
C	2.931803	1.462850	-0.736633
H	-4.250442	-0.034605	0.875351
H	-4.804511	-1.105764	-1.910910
H	-4.743113	-3.723473	1.085374
H	-5.178178	-2.191170	1.827828
H	-5.916788	-3.104862	-0.976624
H	-6.662153	-0.503285	0.420518
H	-7.176034	-1.014432	-1.185357
H	-3.359553	-2.543661	-0.607539
H	-2.861527	-2.100140	1.022308
H	-6.240191	1.118116	-2.076329
H	-4.611100	2.872167	-1.865729
H	-2.298824	2.093342	-1.535662
H	-2.528120	-0.187798	-1.622251
H	-4.874369	2.257170	1.012123
H	-3.041757	4.035930	2.775542
H	-4.756813	4.322900	2.472752
H	-4.256407	2.907329	3.378777
H	-1.494769	3.818167	1.531220
H	-0.757978	2.523344	0.593179
H	-1.390217	3.937810	-0.227566
H	-7.244030	-4.194147	0.824453
H	-7.651772	-2.608920	1.492056
H	-8.249128	-3.075429	-0.105557
H	1.904825	0.416732	-2.960733
H	2.590867	-1.818780	-1.145241
H	3.484816	-2.394592	0.722839
H	3.393448	-1.083694	2.644376
H	3.593360	0.438037	1.811806
H	5.114940	-0.785593	-1.432417
H	7.366515	-0.908017	-1.951041
H	7.597804	-1.867246	-0.480231
H	7.161169	-0.299601	1.420691
H	2.221525	2.288601	-0.790103
H	3.495500	1.556344	0.188295
H	3.632833	1.542574	-1.569022

ωB97X Energy = -1950.62663916 a.u.

(3*R*,4*S*,5*R*,8*R*,10*S*,19*S*,24*S*)-**1**, Conf G

C	-5.375751	-2.467366	0.627533
C	-6.590713	-2.005497	-0.177487
C	-6.591156	-0.482468	-0.306897
C	-5.272884	0.036588	-0.880416
C	-4.093832	-0.390033	0.002639
C	-4.059792	-1.917592	0.079410
C	-5.287428	1.521543	-1.104979
C	-4.217110	2.292125	-0.965081
C	-2.845004	1.796052	-0.587648
C	-2.806626	0.233421	-0.542936
C	-2.332983	2.491235	0.670737
C	-1.192100	3.178842	0.604427
C	-0.482754	3.906717	1.705394
C	-3.153672	2.343123	1.923404
C	-1.554249	-0.182970	0.199314
C	-7.890960	-2.514484	0.431004
C	-0.250749	-0.079745	-0.477396
O	-1.577989	-0.557226	1.362126
C	0.024298	0.192815	-1.914364
N	1.369859	0.007168	-2.093748
C	2.091545	-0.096397	-0.842480
C	0.935818	-0.300299	0.131492
O	-0.754042	0.495052	-2.801963
N	2.966973	-1.261801	-0.878713
C	3.587580	-1.680721	0.380577
C	3.036975	-1.042287	1.667887
S	1.226513	-0.890968	1.722244
C	5.109037	-1.476113	0.375500
N	5.643178	-1.118096	-0.799986
C	7.050911	-0.875180	-0.932818
C	7.556873	0.384195	-0.254486
O	6.596759	1.257231	0.055072
O	5.773133	-1.654743	1.389665
O	8.726786	0.584303	-0.045220
C	2.862872	1.199373	-0.586703
H	-4.276346	-0.005076	1.012353
H	-5.139499	-0.445753	-1.861259
H	-5.344217	-3.561168	0.652791
H	-5.496354	-2.134054	1.666797
H	-6.496611	-2.421719	-1.189743
H	-6.757639	-0.032785	0.681543
H	-7.423261	-0.162673	-0.943670
H	-3.881174	-2.321736	-0.925676
H	-3.233508	-2.249217	0.709862

H	-6.231515	1.967547	-1.411342
H	-4.295438	3.363357	-1.135918
H	-2.167624	2.089734	-1.394699
H	-2.686619	-0.087707	-1.582499
H	-0.685540	3.211840	-0.359710
H	0.504298	3.465081	1.873271
H	-0.316894	4.952957	1.434929
H	-1.021417	3.882043	2.651820
H	-4.216845	2.465391	1.703953
H	-3.023464	1.344886	2.351237
H	-2.880426	3.072063	2.684844
H	-7.894943	-3.605171	0.502699
H	-8.025934	-2.112231	1.440054
H	-8.754381	-2.212210	-0.167343
H	1.817674	0.396538	-2.909554
H	2.449798	-2.028956	-1.286460
H	3.448515	-2.761156	0.466060
H	3.288821	-1.678957	2.513167
H	3.486278	-0.071709	1.863823
H	5.000896	-0.948584	-1.560226
H	7.301080	-0.792753	-1.991016
H	7.627216	-1.703867	-0.519754
H	7.012090	2.027416	0.468408
H	2.164241	2.035643	-0.535213
H	3.444362	1.178117	0.331573
H	3.554048	1.363274	-1.415068

ωB97X Energy = -1950.62659233 a.u.

(3*R*,4*S*,5*R*,8*R*,10*S*,19*S*,24*S*)-**1**, Conf H

C	4.746317	-2.884000	-0.873681
C	5.960385	-2.864842	0.056327
C	6.280443	-1.432859	0.486051
C	5.055774	-0.742980	1.087017
C	3.909524	-0.705952	0.068929
C	3.538978	-2.144410	-0.297849
C	5.365806	0.617951	1.639899
C	4.542699	1.653755	1.547571
C	3.195548	1.614468	0.875082
C	2.763623	0.127877	0.645530
C	3.157612	2.502999	-0.370568
C	4.206909	2.591554	-1.187134
C	4.337918	3.395124	-2.445459
C	1.881418	3.281854	-0.551236
C	1.499524	0.086565	-0.181132
C	7.165418	-3.536959	-0.589100
C	0.196579	0.275467	0.479532
O	1.516479	-0.067227	-1.393170
C	-0.093875	0.403915	1.933769
N	-1.458579	0.365450	2.061535
C	-2.141610	0.505577	0.792639
C	-0.985339	0.285854	-0.176527
O	0.683934	0.505669	2.865244
N	-3.150999	-0.541863	0.666565
C	-3.735266	-0.774377	-0.655434
C	-3.099057	-0.018172	-1.833524
S	-1.281854	-0.039294	-1.840134
C	-5.236648	-0.478776	-0.678265
N	-5.792211	-0.130852	0.479988
C	-7.162819	0.328384	0.600888
C	-8.212356	-0.769598	0.439606
O	-8.160788	-1.466391	-0.690477
O	-5.879448	-0.588184	-1.729397
O	-9.067046	-0.977944	1.263054
C	-2.745846	1.906307	0.686362
H	4.272559	-0.209801	-0.839552
H	4.721721	-1.368144	1.930027
H	4.477735	-3.919608	-1.104788
H	5.027489	-2.414325	-1.825447
H	5.695732	-3.426384	0.962567
H	6.629759	-0.858846	-0.382894
H	7.099629	-1.437531	1.213309
H	3.179913	-2.662571	0.600909
H	2.725071	-2.158461	-1.023929
H	6.316633	0.734823	2.156024
H	4.833631	2.614966	1.963037
H	2.471841	2.034537	1.582668
H	2.514236	-0.259774	1.637588
H	5.093931	2.020064	-0.922880
H	4.605351	2.745291	-3.283123
H	3.427407	3.928778	-2.714328
H	5.142777	4.129576	-2.350056
H	1.870502	3.874203	-1.464315
H	1.007105	2.625832	-0.571399
H	1.740368	3.962617	0.294090
H	6.943901	-4.572466	-0.860086
H	7.457764	-3.007219	-1.501198
H	8.025336	-3.541820	0.085730
H	-1.883547	0.713373	2.907848
H	-2.757780	-1.399710	1.029571
H	-3.654704	-1.842310	-0.872096
H	-3.388069	-0.508140	-2.760776
H	-3.450379	1.008823	-1.897321
H	-5.171330	-0.102976	1.278311
H	-7.359357	1.093008	-0.156923
H	-7.292487	0.772862	1.582965
H	-7.409958	-1.154415	-1.254350
H	-1.957149	2.654046	0.777281
H	-3.270183	2.075434	-0.250785
H	-3.458276	2.040091	1.502053

ωB97X Energy = -1950.62648774 a.u.

(3*R*,4*S*,5*R*,8*R*,10*S*,19*S*,24*S*)-**1**, Conf I

C	-5.070966	-2.537515	0.853194
C	-6.252328	-2.356715	-0.100727
C	-6.383335	-0.890129	-0.511884
C	-5.072206	-0.351223	-1.082796
C	-3.942204	-0.477746	-0.053870
C	-3.768639	-1.955144	0.304376
C	-5.198648	1.048923	-1.610096
C	-4.244334	1.963368	-1.500549

C	-2.912696	1.730470	-0.836779
C	-2.690954	0.196778	-0.620739
C	-2.742174	2.596227	0.413786
C	-3.760377	2.816128	1.244458
C	-3.771555	3.625399	2.505938
C	-1.371367	3.196292	0.583058
C	-1.443791	-0.025077	0.202702
C	-7.545314	-2.886575	0.505577
C	-0.129949	-0.011656	-0.462440
O	-1.481765	-0.181721	1.413973
C	0.166140	0.068965	-1.919052
N	1.511397	-0.154159	-2.054053
C	2.217361	-0.095807	-0.790319
C	1.048160	-0.153765	0.186833
O	-0.596657	0.268782	-2.847871
N	3.077786	-1.265784	-0.656776
C	3.677100	-1.518334	0.656381
C	3.120681	-0.703768	1.837357
S	1.312796	-0.516248	1.847183
C	5.202207	-1.334297	0.649403
N	5.755885	-1.141298	-0.556225
C	7.157436	-0.888334	-0.705281
C	7.536473	0.536191	-0.345830
O	8.860728	0.704549	-0.420943
O	5.845093	-1.393357	1.690268
O	6.770997	1.411870	-0.039463
C	3.004184	1.212970	-0.705026
H	-4.242747	0.057023	0.855451
H	-4.808667	-0.997870	-1.934665
H	-4.940187	-3.600388	1.079514
H	-5.308645	-2.041987	1.803733
H	-6.036874	-2.933241	-1.010597
H	-6.673731	-0.289952	0.361228
H	-7.183807	-0.781313	-1.251854
H	-3.465927	-2.508585	-0.594186
H	-2.973931	-2.079594	1.041268
H	-6.125458	1.301143	-2.121504
H	-4.404001	2.962489	-1.897404
H	-2.142095	2.051384	-1.546639
H	-2.497044	-0.213343	-1.616075
H	-4.716730	2.364229	0.990444
H	-2.811599	4.087604	2.731694
H	-4.521195	4.419104	2.443518
H	-4.050568	2.998427	3.357431
H	-1.147358	3.855828	-0.261203
H	-1.270961	3.775797	1.498782
H	-0.594199	2.427296	0.591413
H	-7.454681	-3.944084	0.766797
H	-7.796537	-2.336749	1.418119
H	-8.381779	-2.781496	-0.190193
H	1.973344	0.124575	-2.906553
H	2.554860	-2.074885	-0.963642
H	3.521241	-2.575456	0.884088
H	3.350845	-1.223369	2.764819
H	3.583096	0.277775	1.906600
H	5.120205	-1.056003	-1.336095
H	7.458888	-1.073068	-1.736575
H	7.734926	-1.559453	-0.067269
H	9.069814	1.621814	-0.195408
H	2.317886	2.057440	-0.778063
H	3.577081	1.311795	0.213622
H	3.705729	1.251018	-1.539900

ωB97X Energy = -1950.62622165 a.u.

(3*R*,4*S*,5*R*,8*R*,10*S*,19*S*,24*S*)-**1**, Conf J

C	-5.176286	2.767092	-0.748689
C	-6.424595	2.479262	0.086100
C	-6.571400	0.976060	0.320929
C	-5.304250	0.372732	0.926257
C	-4.100663	0.618621	0.008430
C	-3.915834	2.127140	-0.168798
C	-5.462535	-1.082668	1.259678
C	-4.476049	-1.964247	1.170730
C	-3.067963	-1.637216	0.744388
C	-2.873275	-0.094054	0.581308
C	-2.655561	-2.478505	-0.460705
C	-1.603540	-3.290153	-0.345995
C	-1.008120	-4.192768	-1.383111
C	-3.477201	-2.336604	-1.713377
C	-1.602453	0.132768	-0.209769
C	-7.672927	3.068599	-0.557830
C	-0.300924	-0.082745	0.443826
O	-1.615096	0.430702	-1.394512
C	-0.012578	-0.269349	1.891308
N	1.352405	-0.266553	2.018438
C	2.030805	-0.381567	0.743588
C	0.879974	-0.089418	-0.213078
O	-0.791542	-0.378169	2.821963
N	3.073799	0.633622	0.651965
C	3.684486	0.864858	-0.659912
C	3.002282	0.195096	-1.864641
S	1.188483	0.310765	-1.858956
C	5.169392	0.478370	-0.693561
N	5.691862	0.060874	0.467908
C	7.084543	-0.267131	0.580912
C	8.037740	0.912015	0.525014
O	7.453857	2.091954	0.736691
O	5.817806	0.568866	-1.729274
O	9.221043	0.782129	0.336036
C	2.587675	-1.797555	0.586262
H	-4.335098	0.189530	-0.972226
H	-5.113629	0.908902	1.868899
H	-5.038616	3.848543	-0.845754
H	-5.336705	2.380097	-1.763661
H	-6.285720	2.952953	1.067481
H	-6.789168	0.477520	-0.633412
H	-7.425050	0.783823	0.980033
H	-3.689825	2.575424	0.807514
H	-3.066283	2.333325	-0.821526
H	-6.441576	-1.408155	1.605655
H	-4.656469	-3.007124	1.420999

H	-2.409209	-1.937707	1.564263
H	-2.697128	0.288342	1.591865
H	-1.090014	-3.305775	0.614969
H	-0.998423	-5.228687	-1.033154
H	-1.537314	-4.160880	-2.334573
H	0.033481	-3.915922	-1.571907
H	-4.543751	-2.347348	-1.476359
H	-3.265220	-1.383571	-2.206127
H	-3.282650	-3.133753	-2.429114
H	-7.573273	4.147892	-0.698795
H	-7.847095	2.617566	-1.539869
H	-8.559741	2.888170	0.055146
H	1.769613	-0.651874	2.852289
H	2.699063	1.498210	1.018402
H	3.674786	1.942867	-0.837990
H	3.312000	0.707198	-2.772990
H	3.297853	-0.845375	-1.975132
H	5.086451	0.083916	1.275437
H	7.385370	-0.948277	-0.215776
H	7.256004	-0.774082	1.530947
H	8.135869	2.777263	0.696324
H	1.773053	-2.520891	0.644019
H	3.113238	-1.948615	-0.353222
H	3.289960	-1.986989	1.399662

ωB97X Energy = -1950.62620755 a.u.

(3*R*,4*S*,5*R*,8*R*,10*S*,19*S*,24*S*)-**1**, Conf K

C	-4.688902	-2.867168	1.025736
C	-5.880478	-2.943883	0.070180
C	-6.228662	-1.553646	-0.461854
C	-5.009652	-0.869532	-1.080395
C	-3.887659	-0.734898	-0.043841
C	-3.488454	-2.135224	0.426485
C	-5.344297	0.445341	-1.723067
C	-4.550154	1.506802	-1.680988
C	-3.213680	1.545034	-0.987561
C	-2.750794	0.088408	-0.653127
C	-3.214485	2.515478	0.195909
C	-4.275729	2.629870	0.993526
C	-4.443532	3.512000	2.193509
C	-1.960515	3.337408	0.337655
C	-1.501983	0.131871	0.196938
C	-7.082414	-3.605674	0.731815
C	-0.191250	0.306228	-0.450996
O	-1.540570	0.056968	1.416011
C	0.120959	0.361865	-1.905200
N	1.487073	0.333504	-2.009871
C	2.150259	0.544434	-0.739666
C	0.980437	0.365990	0.221934
O	-0.643408	0.405470	-2.852841
N	3.163716	-0.486271	-0.543553
C	3.741550	-0.632847	0.795449
C	3.073050	0.169008	1.924114
S	1.255661	0.131212	1.903699
C	5.242683	-0.310659	0.827054
N	5.796119	-0.005712	-0.355515
C	7.201935	0.236236	-0.484430
C	8.023043	-1.038983	-0.485098
O	9.329978	-0.757980	-0.515459
O	5.866267	-0.341787	1.880819
O	7.583533	-2.158214	-0.469374
C	2.742835	1.953511	-0.698178
H	-4.283089	-0.188980	0.821496
H	-4.640042	-1.536968	-1.874603
H	-4.398833	-3.877101	1.332282
H	-5.004359	-2.342765	1.937393
H	-5.579000	-3.557095	-0.789949
H	-6.614383	-0.933293	0.358531
H	-7.029861	-1.629922	-1.205070
H	-3.096705	-2.702345	-0.427962
H	-2.690216	-2.078650	1.167804
H	-6.288453	0.504745	-2.260838
H	-4.858753	2.432672	-2.159297
H	-2.489102	1.933720	-1.711746
H	-2.473297	-0.357414	-1.612782
H	-5.143859	2.017969	0.758383
H	-4.703718	2.913051	3.070504
H	-3.550536	4.086523	2.435735
H	-5.266212	4.216366	2.040585
H	-1.075480	2.706812	0.459247
H	-1.800408	3.926995	-0.570257
H	-1.994867	4.023928	1.181585
H	-6.838223	-4.611791	1.082372
H	-7.414919	-3.021818	1.595907
H	-7.923103	-3.685065	0.037703
H	1.922523	0.638963	-2.867026
H	2.776390	-1.366945	-0.854292
H	3.678015	-1.689846	1.065153
H	3.350094	-0.272708	2.878736
H	3.412515	1.201903	1.946351
H	5.197556	-0.074423	-1.165898
H	7.554690	0.861991	0.337177
H	7.400347	0.770644	-1.413661
H	9.824866	-1.589222	-0.527123
H	1.950015	2.690014	-0.834385
H	3.256963	2.171437	0.234465
H	3.463450	2.051095	-1.511827

ωB97X Energy = -1950.62568869 a.u.

(3*R*,4*S*,5*R*,8*R*,10*S*,19*R*,24*R*)-**3**, Conf A

C	-4.649611	-0.155236	-2.738697
C	-6.049010	-0.062739	-2.129975
C	-6.059738	-0.682096	-0.732409
C	-4.988722	-0.062399	0.164720
C	-3.596178	-0.246401	-0.450074
C	-3.569511	0.422686	-1.825634
C	-5.044574	-0.575867	1.575029
C	-3.971320	-0.733235	2.338319
C	-2.561065	-0.410265	1.914654

C	-2.545678	0.297674	0.521263
C	-1.658343	-1.634729	2.035504
C	-0.580965	-1.562591	2.818321
C	0.455251	-2.615665	3.070057
C	-2.045376	-2.856081	1.246254
C	-1.129306	0.261212	-0.013283
C	-7.094945	-0.702034	-3.034059
C	-0.154187	1.213393	0.494871
O	-0.778312	-0.583806	-0.838552
C	-0.363951	2.367449	1.385564
N	0.824165	3.070106	1.412880
C	1.908519	2.320646	0.830571
C	1.162349	1.229328	0.102430
O	-1.364584	2.714154	1.992284
S	2.790274	3.316031	-0.434451
C	3.761835	1.932527	-1.082329
C	2.909137	0.774112	-1.610710
N	1.751955	0.424513	-0.786081
C	2.832352	1.765849	1.913606
H	-3.431461	-1.320253	-0.593294
H	-5.190398	1.019532	0.199742
C	3.856011	-0.408728	-1.816099
N	3.718573	-1.449916	-0.976863
C	4.673867	-2.520566	-0.975480
C	5.975593	-2.127589	-0.302027
O	4.706299	-0.356743	-2.691934
O	6.877900	-3.106585	-0.407302
O	6.183513	-1.085370	0.261572
H	-4.634632	0.353797	-3.707509
H	-4.423128	-1.211345	-2.936433
H	-6.293653	1.002245	-2.018006
H	-5.886406	-1.763955	-0.811811
H	-7.046736	-0.552672	-0.274943
H	-3.731306	1.501247	-1.699527
H	-2.590453	0.297949	-2.291063
H	-6.027596	-0.808667	1.979533
H	-4.079382	-1.114572	3.351223
H	-2.173414	0.325340	2.625838
H	-2.785906	1.345798	0.724411
H	-0.403275	-0.619497	3.334140
H	0.229272	-3.564744	2.585424
H	0.567457	-2.799225	4.141881
H	1.431360	-2.280664	2.705859
H	-1.519283	-3.749351	1.579729
H	-3.118304	-3.043873	1.330084
H	-1.823688	-2.710007	0.185465
H	-7.104527	-0.232939	-4.021242
H	-6.885799	-1.767587	-3.171738
H	-8.096974	-0.610058	-2.607081
H	1.000903	3.694189	2.185056
H	4.349697	2.322213	-1.911085
H	4.463962	1.589431	-0.322400
H	2.536993	1.043522	-2.602636
H	1.078026	-0.206236	-1.218249
H	3.618094	1.130649	1.508442
H	3.297048	2.593292	2.450832
H	2.242639	1.171344	2.614778
H	3.041876	-1.387530	-0.233079
H	4.898152	-2.828788	-1.997321
H	4.260062	-3.383221	-0.453649
H	7.690416	-2.829939	0.039435

ωB97X Energy = -1950.64243173 a.u.

(3*R*,4*S*,5*R*,8*R*,10*S*,19*R*,24*R*)-**3**, Conf B

C	4.221425	1.746993	-2.337599
C	5.669671	1.466540	-1.935226
C	5.758917	1.186691	-0.434886
C	4.801586	0.073619	-0.009361
C	3.356119	0.444892	-0.362315
C	3.256584	0.657204	-1.873944
C	4.943873	-0.288942	1.440985
C	3.928581	-0.680164	2.199407
C	2.506318	-0.827716	1.722350
C	2.410314	-0.631680	0.174342
C	1.561699	0.038140	2.551523
C	0.558596	-0.550958	3.203836
C	-0.499060	0.088433	4.051430
C	1.823259	1.519927	2.572167
C	0.955024	-0.409969	-0.181751
C	6.591981	2.606941	-2.346510
C	0.048421	-1.548260	-0.205791
O	0.513617	0.719367	-0.398717
C	0.358038	-2.983683	-0.095018
N	-0.820370	-3.664611	-0.329914
C	-1.962635	-2.785732	-0.307030
C	-1.302569	-1.433133	-0.421821
O	1.422363	-3.537704	0.128019
S	-3.005266	-3.024354	-1.797701
C	-4.028627	-1.557183	-1.514475
C	-3.226472	-0.254633	-1.465716
N	-1.990631	-0.317328	-0.684111
C	-2.737634	-2.930209	1.002069
H	3.126815	1.397586	0.128394
H	5.065317	-0.817835	-0.599678
C	-4.168913	0.842261	-0.967152
N	-3.989889	1.268505	0.294986
C	-4.814780	2.304362	0.848040
C	-4.428510	3.685491	0.354135
O	-5.063145	1.248235	-1.694020
O	-5.288769	4.603381	0.805260
O	-3.480519	3.943056	-0.338962
H	4.157333	1.869727	-3.423324
H	3.914717	2.704483	-1.896141
H	5.991012	0.556047	-2.458931
H	5.518595	2.103168	0.121175
H	6.786315	0.915741	-0.167966
H	3.492571	-0.286966	-2.382086
H	2.236844	0.925958	-2.154135
H	5.942791	-0.237030	1.869563
H	4.097401	-0.923816	3.245873
H	2.212157	-1.864395	1.910067

H	2.705566	-1.591537	-0.261062
H	0.466619	-1.632083	3.104404
H	-0.348241	1.158266	4.190664
H	-0.539443	-0.380818	5.037891
H	-1.484702	-0.052415	3.596791
H	1.505297	1.976242	1.630569
H	1.296995	2.022029	3.382791
H	2.890925	1.721169	2.685795
H	6.541353	2.787369	-3.423391
H	6.307274	3.533699	-1.838371
H	7.632077	2.390343	-2.089478
H	-0.907452	-4.611420	0.006292
H	-4.725100	-1.493409	-2.348455
H	-4.618318	-1.693522	-0.608401
H	-2.959333	0.022161	-2.489070
H	-1.369108	0.484527	-0.780341
H	-3.555204	-2.216093	1.082897
H	-3.146593	-3.938609	1.072620
H	-2.056617	-2.761777	1.839347
H	-3.197182	0.930828	0.816647
H	-4.734597	2.294299	1.934926
H	-5.861216	2.132659	0.592133
H	-5.010005	5.473037	0.485719

ωB97X Energy = -1950.64171875 a.u.

(3*R*,4*S*,5*R*,8*R*,10*S*,19*R*,24*R*)-**3**, Conf C

C	-4.520197	0.192393	-2.683216
C	-5.910209	0.153511	-2.046971
C	-5.871721	-0.626201	-0.732682
C	-4.803787	-0.080789	0.215142
C	-3.419917	-0.143314	-0.441925
C	-3.443089	0.693348	-1.722293
C	-4.815064	-0.761165	1.554270
C	-3.721286	-0.984819	2.270733
C	-2.327824	-0.584018	1.857710
C	-2.364190	0.302816	0.572209
C	-1.405720	-1.798584	1.791004
C	-0.307166	-1.814803	2.547347
C	0.743404	-2.880944	2.629788
C	-1.794625	-2.910506	0.854585
C	-0.959821	0.382217	0.013031
C	-6.946255	-0.418763	-3.005822
C	-0.003790	1.291753	0.624478
O	-0.601651	-0.336724	-0.921591
C	-0.230076	2.314010	1.660086
N	0.934290	3.050640	1.753612
C	2.028895	2.421284	1.058673
C	1.302164	1.405075	0.212156
O	-1.226494	2.543668	2.326519
S	2.840095	3.601335	-0.089291
C	3.844241	2.348005	-0.927294
C	3.020537	1.233302	-1.580637
N	1.894612	0.744042	-0.784939
C	2.997814	1.769577	2.043833
H	-3.228690	-1.184975	-0.722325
H	-5.038794	0.981767	0.383544
C	3.991522	0.112681	-1.960019
N	3.932705	-1.007899	-1.229811
C	4.838559	-2.103487	-1.454430
C	4.618461	-3.157225	-0.399369
O	4.794869	0.287724	-2.866089
O	5.445883	-4.186787	-0.559464
O	3.796211	-3.082316	0.478920
H	-4.543743	0.817696	-3.581324
H	-4.258351	-0.821117	-3.014913
H	-6.195125	1.187005	-1.807435
H	-5.664277	-1.684524	-0.941792
H	-6.853421	-0.586452	-0.248116
H	-3.639471	1.741328	-1.460506
H	-2.469663	0.663054	-2.214496
H	-5.783485	-1.063301	1.948150
H	-3.798185	-1.486883	3.232468
H	-1.930250	0.057270	2.650127
H	-2.630615	1.306240	0.918408
H	-0.122514	-0.944169	3.175848
H	0.458463	-3.800376	2.118920
H	0.960507	-3.125509	3.672646
H	1.680794	-2.534847	2.182606
H	-1.570378	-2.631781	-0.178989
H	-1.267883	-3.838694	1.072020
H	-2.867549	-3.107286	0.912296
H	-6.987854	0.156508	-3.934320
H	-6.700686	-1.453776	-3.263905
H	-7.944373	-0.413365	-2.560270
H	1.109269	3.575635	2.596689
H	4.397641	2.861491	-1.711290
H	4.574669	1.943283	-0.227357
H	2.615542	1.613015	-2.522400
H	1.231481	0.148274	-1.278994
H	3.796222	1.221839	1.546488
H	3.444190	2.539303	2.674290
H	2.446823	1.068200	2.674122
H	3.255397	-1.101596	-0.485943
H	5.878703	-1.771304	-1.410855
H	4.687179	-2.556912	-2.437670
H	5.269954	-4.840523	0.132213

ωB97X Energy = -1950.64168129 a.u.

(3*R*,4*S*,5*R*,8*R*,10*S*,19*R*,24*R*)-**3**, Conf D

C	-4.417871	-1.035566	-2.711045
C	-5.859632	-0.790934	-2.264174
C	-5.977591	-0.943881	-0.747783
C	-4.975229	-0.054434	-0.012888
C	-3.541520	-0.395593	-0.436368
C	-3.407976	-0.182421	-1.945327
C	-5.139951	-0.105911	1.479096
C	-4.128155	-0.002620	2.330390
C	-2.687201	0.193617	1.933145
C	-2.561271	0.436350	0.394811
C	-1.810304	-0.920961	2.496615

C	-0.806481	-0.596941	3.312893
C	0.183627	-1.508674	3.972101
C	-2.142015	-2.331026	2.089226
C	-1.111645	0.252243	-0.002122
C	-6.832357	-1.705559	-2.997160
C	-0.162310	1.331045	0.233850
O	-0.714263	-0.803722	-0.496386
C	-0.418634	2.697227	0.725629
N	0.756014	3.402730	0.566679
C	1.868185	2.543869	0.250181
C	1.162959	1.260122	-0.112711
O	-1.443364	3.184335	1.173871
S	2.732934	3.129402	-1.260219
C	3.753490	1.646934	-1.450273
C	2.937610	0.357734	-1.598744
N	1.794446	0.232097	-0.695305
C	2.795507	2.380022	1.452247
H	-3.374606	-1.458541	-0.228996
H	-5.173379	0.980765	-0.331477
C	3.908157	-0.808234	-1.454474
N	3.826557	-1.555359	-0.352283
C	4.617398	-2.759106	-0.160809
C	6.087031	-2.494154	0.160428
O	4.760181	-0.995446	-2.326920
O	6.758786	-1.769187	-0.729079
O	6.628899	-2.937339	1.140304
H	-4.328032	-0.850010	-3.785962
H	-4.179183	-2.096082	-2.555824
H	-6.112546	0.249620	-2.509230
H	-5.801647	-1.992757	-0.472405
H	-6.996323	-0.699736	-0.427241
H	-3.574793	0.879061	-2.170677
H	-2.396837	-0.424939	-2.276420
H	-6.152055	-0.218861	1.862601
H	-4.314040	-0.053347	3.400813
H	-2.346200	1.118215	2.408074
H	-2.805187	1.492775	0.245609
H	-0.662686	0.460993	3.529850
H	1.196861	-1.283984	3.625917
H	-0.007368	-2.562940	3.775607
H	0.182670	-1.359538	5.055184
H	-1.833390	-2.510213	1.055668
H	-1.651901	-3.071111	2.720042
H	-3.219739	-2.502653	2.140013
H	-6.761631	-1.570684	-4.079547
H	-6.614920	-2.755347	-2.776197
H	-7.865372	-1.507972	-2.699512
H	0.900834	4.241065	1.108232
H	4.337420	1.781922	-2.358709
H	4.456443	1.578372	-0.620269
H	2.561453	0.306283	-2.624327
H	1.138565	-0.514952	-0.922763
H	3.597199	1.667358	1.267151
H	3.238348	3.344728	1.701410
H	2.214856	2.022767	2.305255
H	3.124468	-1.323560	0.333956
H	4.571630	-3.369958	-1.066959
H	4.190794	-3.324070	0.662101
H	6.169379	-1.507396	-1.475806

ωB97X Energy = -1950.64151997 a.u.

(3*R*,4*S*,5*R*,8*R*,10*S*,19*R*,24*R*)-**3**, Conf E

C	4.256354	1.823749	-2.329871
C	5.707141	1.553740	-1.929384
C	5.798346	1.256774	-0.432457
C	4.851948	0.129153	-0.020818
C	3.403289	0.491360	-0.370013
C	3.302472	0.718869	-1.879386
C	4.997577	-0.249360	1.425064
C	3.985811	-0.657509	2.179190
C	2.564429	-0.810224	1.701315
C	2.466112	-0.598142	0.155388
C	1.614121	0.040647	2.539565
C	0.615922	-0.562304	3.186745
C	-0.445462	0.060595	4.041863
C	1.864965	1.524058	2.574551
C	1.009099	-0.381730	-0.194940
C	6.617602	2.708684	-2.326299
C	0.106532	-1.525030	-0.221704
O	0.560523	0.745894	-0.403512
C	0.423906	-2.961118	-0.126339
N	-0.753716	-3.643848	-0.356008
C	-1.899097	-2.770198	-0.319241
C	-1.245077	-1.414346	-0.422682
O	1.492872	-3.511094	0.081425
S	-2.942090	-2.994475	-1.812596
C	-3.973570	-1.538452	-1.508361
C	-3.175834	-0.231955	-1.444023
N	-1.940654	-0.294991	-0.664335
C	-2.671152	-2.933384	0.989130
H	3.165744	1.436866	0.130585
H	5.124445	-0.752522	-0.621711
C	-4.122138	0.842333	-0.919392
N	-3.930900	1.288345	0.324016
C	-4.888537	2.142223	1.006563
C	-4.897584	3.589496	0.518535
O	-5.042821	1.240323	-1.636950
O	-5.132325	3.769326	-0.777676
O	-4.739467	4.525730	1.259280
H	4.191919	1.957848	-3.414223
H	3.939255	2.772991	-1.878075
H	6.038520	0.653039	-2.463661
H	5.548514	2.163937	0.134589
H	6.828282	0.992933	-0.168279
H	3.548823	-0.217132	-2.397559
H	2.280287	0.980167	-2.157738
H	5.996221	-0.194605	1.853873
H	4.156855	-0.912133	3.222647
H	2.277675	-1.850780	1.878522
H	2.767039	-1.551229	-0.290880
H	0.532222	-1.643137	3.077127

H	-1.431053	-0.087817	3.589503
H	-0.305360	1.131022	4.187348
H	-0.477621	-0.415278	5.025445
H	2.931171	1.731771	2.690123
H	1.543842	1.987389	1.637423
H	1.335307	2.014520	3.390028
H	6.566619	2.900466	-3.401199
H	6.322012	3.626570	-1.808389
H	7.659595	2.500460	-2.070079
H	-0.834635	-4.595728	-0.032929
H	-4.672440	-1.467256	-2.339677
H	-4.559771	-1.689950	-0.602456
H	-2.917017	0.063264	-2.464356
H	-1.317465	0.505443	-0.765430
H	-3.491079	-2.223197	1.080625
H	-3.076502	-3.943928	1.048082
H	-1.988966	-2.772850	1.826947
H	-3.145536	0.931545	0.846677
H	-4.647952	2.148730	2.065304
H	-5.895016	1.733843	0.877746
H	-5.256904	2.900780	-1.228670

ωB97X Energy = -1950.64138838 a.u.

(3*R*,4*S*,5*R*,8*R*,10*S*,19*R*,24*R*)-**3**, Conf F

C	-4.633310	-0.780982	-2.660134
C	-6.038346	-0.571558	-2.094045
C	-6.049528	-0.838424	-0.588999
C	-4.990778	-0.010084	0.138133
C	-3.593427	-0.321556	-0.411363
C	-3.566302	0.009923	-1.904655
C	-5.052391	-0.172734	1.629624
C	-3.983230	-0.139347	2.413728
C	-2.571125	0.073669	1.931254
C	-2.548655	0.436412	0.411363
C	-1.675959	-1.091695	2.343104
C	-0.617642	-0.844989	3.116559
C	0.399824	-1.815720	3.634596
C	-2.053392	-2.460233	1.844304
C	-1.128985	0.275824	-0.090542
C	-7.065865	-1.429582	-2.820899
C	-0.148241	1.307919	0.210447
O	-0.778253	-0.731396	-0.707384
C	-0.353806	2.629194	0.828138
N	0.847092	3.303848	0.733629
C	1.927984	2.431755	0.349072
C	1.174495	1.222167	-0.148399
O	-1.360349	3.112664	1.320584
S	2.854432	3.125067	-1.074576
C	3.806815	1.619188	-1.397757
C	2.937399	0.393446	-1.695250
N	1.766608	0.237232	-0.831010
C	2.817990	2.100851	1.546324
H	-3.422695	-1.398255	-0.300727
H	-5.202749	1.046963	-0.086428
C	3.859944	-0.824126	-1.644783
N	3.700518	-1.667495	-0.611191
C	4.614347	-2.759010	-0.410926
C	6.002691	-2.357316	0.052517
O	4.721675	-0.968947	-2.499326
O	6.077910	-1.108063	0.515211
O	6.939639	-3.113855	0.027663
H	-4.619784	-0.513097	-3.721321
H	-4.389986	-1.850334	-2.604736
H	-6.303002	0.484116	-2.242598
H	-5.862185	-1.905475	-0.406906
H	-7.040831	-0.617286	-0.178587
H	-3.743589	1.085653	-2.033807
H	-2.582882	-0.205229	-2.325695
H	-6.037109	-0.306226	2.073127
H	-4.095909	-0.267922	3.487787
H	-2.183610	0.954125	2.452039
H	-2.788627	1.503209	0.363104
H	-0.444097	0.190798	3.406677
H	0.160874	-2.852844	3.402323
H	0.500975	-1.724947	4.719206
H	1.384284	-1.598496	3.208820
H	-1.535975	-3.254594	2.380359
H	-3.128308	-2.625169	1.948806
H	-1.812095	-2.558183	0.782149
H	-7.074623	-1.215385	-3.892726
H	-6.837800	-2.492608	-2.693909
H	-8.073074	-1.255624	-2.433513
H	1.020378	4.076965	1.357515
H	4.419585	1.817033	-2.275131
H	4.485441	1.428430	-0.566286
H	2.580655	0.465369	-2.726180
H	1.088728	-0.462019	-1.131225
H	3.598701	1.383339	1.299494
H	3.287803	3.014480	1.912055
H	2.202692	1.673632	2.341203
H	3.014303	-1.453015	0.094239
H	4.742174	-3.324671	-1.333878
H	4.204765	-3.436287	0.338777
H	6.989129	-0.943790	0.796756

ωB97X Energy = -1950.64067352 a.u.

(3*R*,4*S*,5*R*,8*R*,10*S*,19*R*,24*R*)-**3**, Conf G

C	5.067728	-1.759975	-1.742029
C	6.381198	-1.315912	-1.096928
C	6.246102	0.096095	-0.526712
C	5.050931	0.213237	0.418954
C	3.754267	-0.156428	-0.311647
C	3.865878	-1.597674	-0.812455
C	4.960306	1.564840	1.066848
C	3.812259	2.161824	1.356977
C	2.449286	1.572611	1.100544
C	2.561427	0.090103	0.614701
C	1.614440	2.488254	0.209244
C	0.448623	2.943873	0.669764
C	-0.545225	3.820390	-0.029636

C	2.165481	2.813918	-1.152855
C	1.225148	-0.304744	0.022758
C	7.543456	-1.411547	-2.076953
C	0.108184	-0.588041	0.916121
O	1.045660	-0.329611	-1.194672
C	0.104622	-0.735533	2.384071
N	-1.171557	-1.104824	2.737250
C	-2.110483	-0.942284	1.650710
C	-1.166924	-0.817315	0.473808
O	1.020096	-0.602118	3.181074
S	-3.276683	-2.361602	1.465649
C	-2.926340	-2.877190	-0.241768
C	-2.724339	-1.708284	-1.194802
N	-1.584784	-0.888985	-0.799164
C	-2.915716	0.349066	1.843622
H	3.666194	0.497772	-1.186292
H	5.200817	-0.528678	1.218596
C	-4.012759	-0.922897	-1.439398
N	-3.903074	0.401936	-1.574431
C	-4.995607	1.232602	-2.052460
C	-6.094350	1.466818	-1.017432
O	-5.087008	-1.514621	-1.557036
O	-6.668871	0.377215	-0.515978
O	-6.452413	2.568019	-0.687061
H	5.153158	-2.801105	-2.068800
H	4.900797	-1.161506	-2.647383
H	6.583077	-1.991672	-0.254776
H	6.125654	0.812849	-1.350465
H	7.165783	0.372829	0.000284
H	3.972164	-2.267944	0.050476
H	2.954822	-1.891166	-1.336263
H	5.897871	2.057571	1.316556
H	3.815599	3.144921	1.822065
H	1.933190	1.532682	2.064066
H	2.702644	-0.505011	1.522471
H	0.154627	2.635798	1.672826
H	-0.208568	4.150025	-1.011879
H	-0.768795	4.707410	0.568970
H	-1.491565	3.286698	-0.164304
H	2.042186	1.963103	-1.828978
H	1.673807	3.674910	-1.603424
H	3.235014	3.028126	-1.093315
H	7.651843	-2.427157	-2.466326
H	7.383473	-0.742158	-2.928086
H	8.486968	-1.129737	-1.602486
H	-1.471515	-0.989167	3.692803
H	-2.045244	-3.516577	-0.271634
H	-3.793536	-3.455413	-0.554227
H	-2.477255	-2.128079	-2.173873
H	-0.819701	-0.791089	-1.461743
H	-3.595818	0.526818	1.012973
H	-3.509714	0.266753	2.755115
H	-2.233277	1.196572	1.941394
H	-2.993329	0.821744	-1.458565
H	-5.448867	0.767505	-2.932612
H	-4.592874	2.199849	-2.336979
H	-6.272078	-0.434647	-0.911043

ωB97X Energy = -1950.64066083 a.u.

(3*R*,4*S*,5*R*,8*R*,10*S*,19*R*,24*R*)-**3**, Conf H

C	-4.442719	0.129012	-2.759513
C	-5.857669	0.311050	-2.208029
C	-5.971443	-0.313360	-0.817052
C	-4.893815	0.222895	0.125083
C	-3.497119	-0.071348	-0.435760
C	-3.361182	0.615715	-1.795624
C	-5.050433	-0.271690	1.533804
C	-4.029062	-0.580323	2.321569
C	-2.581481	-0.487571	1.916368
C	-2.451668	0.347190	0.599796
C	-1.913856	-1.864165	1.883840
C	-2.568827	-2.938126	1.444986
C	-2.073865	-4.348913	1.339241
C	-0.510618	-1.896804	2.429457
C	-1.028150	0.282487	0.093745
C	-6.902508	-0.252945	-3.162349
C	-0.043105	1.216352	0.617661
O	-0.680494	-0.566184	-0.729414
C	-0.241177	2.359108	1.526197
N	0.949433	3.057679	1.551860
C	2.027457	2.310453	0.954485
C	1.272785	1.229375	0.220107
O	-1.234273	2.697878	2.149076
S	2.904154	3.312686	-0.308776
C	3.869726	1.931106	-0.970200
C	3.012760	0.776487	-1.500746
N	1.852997	0.430043	-0.678127
C	2.956931	1.743600	2.026373
H	-3.411622	-1.153400	-0.593571
H	-5.010886	1.317799	0.149647
C	3.953949	-0.410357	-1.710563
N	3.806539	-1.457643	-0.880791
C	4.751140	-2.537770	-0.888231
C	6.053150	-2.167567	-0.202678
O	4.807606	-0.356537	-2.582936
O	6.943148	-3.157559	-0.310339
O	6.271119	-1.132974	0.371013
H	-4.352247	0.646865	-3.719524
H	-4.281566	-0.937881	-2.963203
H	-6.036639	1.389193	-2.098054
H	-5.872820	-1.404488	-0.897556
H	-6.964591	-0.114799	-0.399592
H	-3.449074	1.701370	-1.658314
H	-2.376310	0.425286	-2.224882
H	-6.065478	-0.357347	1.916758
H	-4.213406	-0.941853	3.329841
H	-2.066829	0.089743	2.693114
H	-2.645319	1.383647	0.889859
H	-3.598112	-2.796170	1.122934
H	-2.677675	-5.013877	1.963393
H	-1.032337	-4.466020	1.635759

H	-2.172840	-4.710231	0.311942
H	0.127748	-1.157027	1.939064
H	-0.521434	-1.640930	3.493716
H	-0.034631	-2.870013	2.323393
H	-6.838205	0.222147	-4.144626
H	-6.755631	-1.328749	-3.300764
H	-7.914429	-0.099635	-2.778526
H	1.134446	3.670290	2.331345
H	4.453547	2.324729	-1.799912
H	4.575733	1.583193	-0.216178
H	2.640907	1.049563	-2.491719
H	1.174611	-0.194688	-1.112501
H	3.736823	1.107756	1.611293
H	3.429317	2.565377	2.565595
H	2.370099	1.147234	2.728044
H	3.127779	-1.397909	-0.138733
H	4.977925	-2.834496	-1.912925
H	4.325593	-3.403036	-0.380349
H	7.756246	-2.895501	0.144052

ωB97X Energy = -1950.64058248 a.u.

(3*R*,4*S*,5*R*,8*R*,10*S*,19*R*,24*R*)-**3**, Conf I

C	4.855940	-0.518726	-2.630826
C	6.225272	-0.412950	-1.958673
C	6.157035	0.525587	-0.754020
C	5.051975	0.114434	0.218927
C	3.690664	0.111030	-0.486767
C	3.740666	-0.879795	-1.650985
C	5.032457	0.956271	1.462161
C	3.921051	1.272926	2.112877
C	2.536650	0.828407	1.716632
C	2.591947	-0.193617	0.533780
C	1.615668	2.026154	1.499921
C	0.502815	2.118938	2.229041
C	-0.559786	3.173887	2.171563
C	2.028110	3.031065	0.458724
C	1.204205	-0.290261	-0.063677
C	7.298231	0.024586	-2.947462
C	0.181258	-1.054961	0.637795
O	0.907686	0.310364	-1.096661
C	0.322056	-1.944389	1.805653
N	-0.909525	-2.520000	2.010356
C	-1.951335	-1.880221	1.241385
C	-1.130672	-1.089858	0.246120
O	1.308217	-2.188164	2.483379
S	-3.078010	-3.083931	0.406047
C	-2.981387	-2.525374	-1.323197
C	-2.870885	-1.014980	-1.477174
N	-1.672155	-0.501004	-0.829104
C	-2.773261	-0.952939	2.144026
H	3.525949	1.112191	-0.901464
H	5.265254	-0.921723	0.524495
C	-4.160883	-0.286653	-1.096495
N	-4.036102	0.922102	-0.540719
C	-5.171749	1.677692	-0.039923
C	-6.038255	2.304984	-1.130479
O	-5.255731	-0.781210	-1.369919
O	-6.542681	1.466766	-2.030670
O	-6.287536	3.482479	-1.165627
H	4.898957	-1.255151	-3.439371
H	4.620625	0.446274	-3.098900
H	6.489221	-1.410366	-1.581672
H	5.971365	1.551458	-1.100339
H	7.122586	0.536747	-0.236690
H	3.910800	-1.888235	-1.251784
H	2.783841	-0.900247	-2.175264
H	5.991809	1.301276	1.842664
H	3.974407	1.890069	3.006905
H	2.122764	0.277974	2.566578
H	2.813296	-1.161575	0.994300
H	0.314557	1.326538	2.952967
H	-0.351631	3.953017	1.439325
H	-0.687719	3.649264	3.147854
H	-1.523741	2.723264	1.914556
H	3.092861	3.258960	0.547447
H	1.865542	2.629313	-0.545217
H	1.474917	3.965399	0.541925
H	7.368277	-0.670303	-3.788337
H	7.068670	1.015860	-3.350959
H	8.280288	0.076998	-2.470412
H	-1.116891	-2.938364	2.903908
H	-2.129651	-2.988982	-1.818345
H	-3.898179	-2.873464	-1.794310
H	-2.752521	-0.811619	-2.544797
H	-0.978692	-0.032799	-1.406774
H	-3.542957	-0.424154	1.586025
H	-3.264341	-1.548867	2.914523
H	-2.112759	-0.227166	2.624528
H	-3.108682	1.283622	-0.378041
H	-4.800389	2.476965	0.594534
H	-5.807188	1.020886	0.562174
H	-6.254374	0.542843	-1.840085

ωB97X Energy = -1950.64052420 a.u.

(3*R*,4*S*,5*R*,8*R*,10*S*,19*R*,24*R*)-**3**, Conf J

C	4.631038	2.091930	-2.052975
C	6.062695	1.694835	-1.690787
C	6.129961	1.204231	-0.244292
C	5.120659	0.086927	0.019257
C	3.695884	0.570200	-0.276265
C	3.614385	0.993924	-1.743870
C	5.240658	-0.486420	1.402162
C	4.204598	-0.925970	2.104086
C	2.777367	-0.926625	1.619086
C	2.699752	-0.525155	0.110971
C	1.880710	-0.123161	2.556952
C	0.848213	-0.735295	3.138262
C	-0.174871	-0.151626	4.064837
C	2.220184	1.328591	2.762196
C	1.256734	-0.196304	-0.214562

C	7.037377	2.839095	-1.938028
C	0.308638	-1.276882	-0.396877
O	0.865501	0.972711	-0.272428
C	0.551589	-2.720111	-0.503312
N	-0.656798	-3.309502	-0.837124
C	-1.759231	-2.396020	-0.666027
C	-1.043770	-1.071376	-0.583304
O	1.587505	-3.353100	-0.367548
S	-2.807145	-2.343587	-2.170719
C	-3.808051	-0.940976	-1.610697
C	-2.997307	0.319959	-1.260658
N	-1.673270	0.085633	-0.701238
C	-2.539677	-2.700486	0.612288
H	3.508507	1.456352	0.340371
H	5.347296	-0.721153	-0.693649
C	-3.835978	1.130239	-0.264361
N	-4.582299	2.109460	-0.807637
C	-5.535460	2.833219	-0.014036
C	-6.766601	2.008028	0.310055
O	-3.845718	0.865303	0.926242
O	-7.570004	2.669414	1.147521
O	-7.009431	0.913120	-0.123892
H	4.581570	2.362679	-3.112429
H	4.363947	2.994467	-1.487289
H	6.348062	0.852768	-2.335943
H	5.927340	2.042880	0.435820
H	7.142346	0.851633	-0.018498
H	3.809542	0.119623	-2.378620
H	2.609330	1.345697	-1.982027
H	6.240727	-0.552061	1.826256
H	4.359142	-1.327403	3.103111
H	2.421880	-1.959945	1.670973
H	2.958149	-1.431772	-0.445455
H	0.700435	-1.790044	2.908649
H	-1.171318	-0.222906	3.618212
H	0.008148	0.896270	4.299363
H	-0.210622	-0.709906	5.004253
H	1.929626	1.914907	1.885978
H	1.719656	1.752206	3.631700
H	3.296761	1.457324	2.896027
H	7.005357	3.168241	-2.979927
H	6.787601	3.698747	-1.308046
H	8.064543	2.543580	-1.709097
H	-0.784605	-4.287071	-0.623919
H	-4.487172	-0.699468	-2.426615
H	-4.416076	-1.256797	-0.764721
H	-2.846208	0.900618	-2.174409
H	-1.046860	0.885230	-0.644719
H	-3.281863	-1.935852	0.836741
H	-3.037685	-3.665945	0.515453
H	-1.838934	-2.742606	1.449107
H	-4.561823	2.262272	-1.802092
H	-5.851820	3.727834	-0.549585
H	-5.083418	3.154395	0.925322
H	-8.350451	2.125251	1.323433

ωB97X Energy = -1950.64035236 a.u.

(3*R*,4*S*,5*R*,8*R*,10*S*,19*R*,24*R*)-**3**, Conf K

C	4.233643	1.867880	-2.240748
C	5.680594	1.574206	-1.842641
C	5.760307	1.205277	-0.361037
C	4.807059	0.062491	-0.011186
C	3.362511	0.448373	-0.351529
C	3.272811	0.746587	-1.849265
C	4.942557	-0.388376	1.414713
C	3.923823	-0.823961	2.143655
C	2.503235	-0.939816	1.653268
C	2.414171	-0.656683	0.118169
C	1.557944	-0.117915	2.525528
C	0.553699	-0.739902	3.144739
C	-0.504897	-0.146252	4.023844
C	1.821463	1.360356	2.625589
C	0.960126	-0.410646	-0.227147
C	6.597588	2.743368	-2.178119
C	0.045431	-1.540353	-0.298475
O	0.524797	0.730347	-0.388870
C	0.344490	-2.981657	-0.265520
N	-0.845374	-3.640935	-0.506672
C	-1.978405	-2.754965	-0.408262
C	-1.308777	-1.404003	-0.477293
O	1.408785	-3.554093	-0.095306
S	-3.069477	-2.913426	-1.874675
C	-4.066818	-1.450999	-1.490888
C	-3.247890	-0.160861	-1.411729
N	-1.991982	-0.270922	-0.668746
C	-2.714756	-2.953777	0.916484
H	3.127547	1.371738	0.190006
H	5.080147	-0.789226	-0.653490
C	-4.159979	0.924794	-0.839833
N	-3.937163	1.302116	0.429788
C	-4.756934	2.309536	1.047762
C	-4.576672	3.711665	0.496578
O	-5.067917	1.379005	-1.520647
O	-3.425495	3.892253	-0.151181
O	-5.389577	4.584900	0.664237
H	4.178239	2.051821	-3.318273
H	3.916354	2.796563	-1.748203
H	6.012943	0.698742	-2.416845
H	5.509479	2.084668	0.247840
H	6.787545	0.925145	-0.103144
H	3.519761	-0.164882	-2.409221
H	2.253431	1.024433	-2.121702
H	5.939848	-0.364837	1.849515
H	4.088238	-1.132427	3.173589
H	2.204957	-1.984149	1.782387
H	2.708609	-1.591223	-0.369915
H	0.462876	-1.814689	2.990435
H	-1.492233	-0.283504	3.571598
H	-0.366584	0.919654	4.200869
H	-0.531242	-0.651749	4.992783
H	1.513600	1.865853	1.706015

H	1.289317	1.820822	3.456726
H	2.888541	1.552424	2.759193
H	6.557039	2.983797	-3.243673
H	6.299474	3.637723	-1.621853
H	7.636716	2.520794	-1.922253
H	-0.932388	-4.602581	-0.215295
H	-4.790318	-1.342820	-2.296873
H	-4.626488	-1.622293	-0.571810
H	-3.006553	0.155573	-2.430062
H	-1.364919	0.528949	-0.743371
H	-3.518656	-2.233499	1.058472
H	-3.136173	-3.958911	0.950345
H	-2.005520	-2.836195	1.738926
H	-3.142954	0.927595	0.922733
H	-4.527608	2.347836	2.113023
H	-5.814137	2.065262	0.942554
H	-3.388314	4.811110	-0.452594

ωB97X Energy = -1950.64024237 a.u.

(3*R*,4*S*,5*R*,8*R*,10*S*,19*R*,24*R*)-**3**, Conf L

C	3.908073	1.312690	-2.688944
C	5.380010	0.975114	-2.447114
C	5.654398	0.827871	-0.950244
C	4.696922	-0.169764	-0.299241
C	3.242477	0.274167	-0.496665
C	2.951660	0.341559	-1.997101
C	5.017978	-0.423184	1.145536
C	4.096379	-0.594284	2.083888
C	2.611365	-0.559584	1.835253
C	2.331462	-0.662173	0.300011
C	1.943674	0.622212	2.542198
C	2.541239	1.811162	2.613062
C	2.032986	3.070526	3.247325
C	0.611132	0.310559	3.170875
C	0.856375	-0.463966	0.034387
C	6.299990	2.009399	-3.083003
C	-0.050018	-1.595180	0.172047
O	0.401970	0.640394	-0.268137
C	0.266259	-3.004880	0.463090
N	-0.901925	-3.718770	0.288113
C	-2.050896	-2.856437	0.174106
C	-1.399224	-1.518256	-0.075177
O	1.328024	-3.515768	0.780201
S	-3.035075	-3.273651	-1.317356
C	-4.086974	-1.801229	-1.235243
C	-3.304343	-0.486392	-1.293959
N	-2.090540	-0.446987	-0.475870
C	-2.872262	-2.862868	1.462686
H	3.134484	1.286254	-0.087714
H	4.821102	-1.124825	-0.833397
C	-4.277678	0.639187	-0.940593
N	-4.129828	1.217595	0.263425
C	-4.988842	2.292498	0.671850
C	-4.649190	3.603325	-0.011634
O	-5.167997	0.935470	-1.723294
O	-5.552925	4.539813	0.292258
O	-3.699537	3.798315	-0.722588
H	3.708422	1.335962	-3.764879
H	3.716196	2.326577	-2.313509
H	5.579507	0.001411	-2.914772
H	5.545509	1.805432	-0.460937
H	6.690331	0.508725	-0.791765
H	3.063446	-0.660648	-2.431246
H	1.921871	0.655063	-2.173527
H	6.071319	-0.481621	1.412622
H	4.397772	-0.762856	3.114465
H	2.190709	-1.468568	2.279735
H	2.570220	-1.695071	0.029968
H	3.525504	1.901675	2.158946
H	1.054774	2.956653	3.712399
H	1.958960	3.867278	2.501860
H	2.730815	3.422161	4.012339
H	-0.095938	-0.084725	2.436477
H	0.732808	-0.464449	3.934084
H	0.150548	1.176973	3.641935
H	6.121011	2.092130	-4.158136
H	6.132501	2.995952	-2.639331
H	7.351608	1.750768	-2.934622
H	-0.990760	-4.621590	0.728466
H	-4.748288	-1.837684	-2.098864
H	-4.712811	-1.851308	-0.344723
H	-3.008118	-0.310627	-2.331473
H	-1.474394	0.347419	-0.643698
H	-3.692544	-2.147559	1.437612
H	-3.282883	-3.859670	1.626326
H	-2.222374	-2.602869	2.300851
H	-3.341058	0.964763	0.836412
H	-4.905905	2.436629	1.748921
H	-6.029720	2.053515	0.449219
H	-5.301355	5.366046	-0.143937

ωB97X Energy = -1950.64009173 a.u.

(3*R*,4*S*,5*R*,8*R*,10*S*,19*R*,24*R*)-**3**, Conf M

C	-4.123407	-0.674133	-2.889125
C	-5.586020	-0.370868	-2.561357
C	-5.850854	-0.575047	-1.069296
C	-4.868688	0.222569	-0.211128
C	-3.426808	-0.198751	-0.518793
C	-3.142737	0.083956	-1.995504
C	-5.178539	0.143240	1.255507
C	-4.249283	0.078794	2.199486
C	-2.766043	0.070572	1.938871
C	-2.482378	0.500244	0.462005
C	-2.122810	-1.249708	2.368228
C	-2.745723	-2.413165	2.185821
C	-2.266243	-3.788253	2.540468
C	-0.784105	-1.105520	3.042668
C	-1.018522	0.302637	0.136897
C	-6.529981	-1.206380	-3.416477
C	-0.059674	1.344256	0.481982

O	-0.617242	-0.729534	-0.401698
C	-0.305225	2.660345	1.101170
N	0.879450	3.361951	1.023618
C	1.984636	2.523321	0.635801
C	1.268656	1.289286	0.144652
O	-1.328842	3.111447	1.587378
S	2.874116	3.242564	-0.799927
C	3.874622	1.769825	-1.128805
C	3.038947	0.517954	-1.414002
N	1.897831	0.311716	-0.521743
C	2.895868	2.225886	1.824883
H	-3.344415	-1.280973	-0.360172
H	-4.971347	1.277865	-0.509064
C	3.987100	-0.674887	-1.405355
N	3.899875	-1.534744	-0.388770
C	4.665812	-2.768361	-0.337430
C	6.144571	-2.571535	-0.009484
O	4.826755	-0.784494	-2.302450
O	6.823384	-1.770287	-0.825052
O	6.686576	-3.129572	0.909736
H	-3.927918	-0.446620	-3.941762
H	-3.953922	-1.752004	-2.765744
H	-5.765000	0.689820	-2.783599
H	-5.760286	-1.642310	-0.825301
H	-6.879079	-0.281794	-0.830872
H	-3.233344	1.162757	-2.177235
H	-2.121311	-0.200188	-2.252173
H	-6.229150	0.158994	1.538781
H	-4.542791	0.013726	3.243986
H	-2.326002	0.851635	2.568878
H	-2.678340	1.575963	0.427431
H	-3.732125	-2.383313	1.728193
H	-2.963605	-4.269402	3.231983
H	-1.279489	-3.796645	3.001619
H	-2.225733	-4.419358	1.648252
H	-0.093729	-0.511962	2.437550
H	-0.902649	-0.572051	3.991098
H	-0.306621	-2.061117	3.251719
H	-6.357988	-1.031715	-4.481708
H	-6.378891	-2.273312	-3.224167
H	-7.575677	-0.972268	-3.200847
H	1.028538	4.142859	1.644015
H	4.470056	1.985598	-2.013982
H	4.567119	1.606000	-0.303525
H	2.657269	0.586846	-2.436632
H	1.240623	-0.410798	-0.814609
H	3.691470	1.526551	1.573670
H	3.345939	3.154049	2.178210
H	2.301941	1.788634	2.630043
H	3.212202	-1.363120	0.328808
H	4.595993	-3.276963	-1.303143
H	4.235945	-3.409688	0.425882
H	6.232284	-1.417335	-1.531893

ωB97X Energy = -1950.63988092 a.u.

(3*R*,4*S*,5*R*,8*R*,10*S*,19*R*,24*R*)-**3**, Conf N

C	-4.321879	0.283778	-2.695039
C	-5.724586	0.396700	-2.096675
C	-5.777750	-0.289256	-0.731131
C	-4.687924	0.236231	0.203161
C	-3.300785	0.017634	-0.412164
C	-3.229105	0.768283	-1.742848
C	-4.783362	-0.335828	1.588397
C	-3.727602	-0.655459	2.325270
C	-2.297314	-0.492884	1.881174
C	-2.240688	0.422498	0.613882
C	-1.588148	-1.842386	1.742708
C	-2.226074	-2.910766	1.266199
C	-1.690369	-4.294764	1.056404
C	-0.163406	-1.859699	2.230804
C	-0.831813	0.448060	0.067337
C	-6.779649	-0.161412	-3.043221
C	0.130196	1.388094	0.620654
O	-0.472785	-0.339979	-0.809762
C	-0.086515	2.484507	1.579505
N	1.090040	3.207688	1.630414
C	2.181503	2.502703	1.005105
C	1.443821	1.447939	0.217763
O	-1.083085	2.775935	2.220597
S	3.057289	3.571754	-0.202131
C	4.041190	2.230326	-0.920260
C	3.197219	1.098577	-1.515258
N	2.032083	0.707927	-0.722008
C	3.108090	1.893656	2.056903
H	-3.183531	-1.052337	-0.623027
H	-4.838869	1.323866	0.287752
C	4.133295	-0.082301	-1.786945
N	3.992592	-1.153964	-0.997328
C	4.829056	-2.316510	-1.139529
C	4.398363	-3.361559	-0.142187
O	4.976245	0.002449	-2.669558
O	5.128966	-4.468310	-0.249660
O	3.506659	-3.216681	0.655680
H	-4.277186	0.841670	-3.635665
H	-4.132469	-0.768315	-2.945986
H	-5.933486	1.463099	-1.936424
H	-5.650953	-1.372569	-0.862116
H	-6.763070	-0.138475	-0.276626
H	-3.350288	1.843471	-1.556830
H	-2.251636	0.630860	-2.207616
H	-5.782189	-0.473496	1.997839
H	-3.868275	-1.077617	3.316911
H	-1.774996	0.053239	2.675478
H	-2.467241	1.431005	0.970903
H	-3.271760	-2.785354	0.993958
H	-2.246831	-5.016542	1.661128
H	-0.634412	-4.393064	1.304817
H	-1.818722	-4.596479	0.013253
H	-0.138645	-1.664108	3.307562
H	0.337400	-2.809440	2.051047
H	0.433002	-1.074196	1.759355

H	-6.756432	0.350695	-4.008585
H	-6.607185	-1.226890	-3.225273
H	-7.784196	-0.051315	-2.626548
H	1.265462	3.783433	2.439816
H	4.631052	2.668464	-1.723013
H	4.740300	1.854580	-0.173782
H	2.831088	1.421772	-2.493329
H	1.363358	0.094381	-1.186369
H	3.890076	1.275450	1.620001
H	3.576329	2.693004	2.632229
H	2.518806	1.267722	2.730217
H	3.281081	-1.173672	-0.280161
H	5.881747	-2.078737	-0.965202
H	4.756684	-2.738128	-2.145159
H	4.823764	-5.109880	0.407569

ωB97X Energy = -1950.63974050 a.u.

(3*R*,4*S*,5*R*,8*R*,10*S*,19*R*,24*R*)-**3**, Conf O

C	3.916401	1.294806	-2.733452
C	5.392744	0.972598	-2.497109
C	5.681334	0.858163	-0.999946
C	4.736598	-0.132562	-0.320307
C	3.277940	0.299259	-0.512921
C	2.972788	0.333132	-2.011637
C	5.072096	-0.357372	1.125841
C	4.160021	-0.517134	2.075420
C	2.672543	-0.494765	1.840386
C	2.378714	-0.625744	0.309899
C	2.005250	0.695947	2.532462
C	2.597283	1.888956	2.576169
C	2.088440	3.156929	3.192470
C	0.680294	0.388279	3.178825
C	0.901231	-0.435088	0.054927
C	6.299436	2.000091	-3.162415
C	-0.002444	-1.568000	0.211103
O	0.441075	0.663750	-0.257103
C	0.320369	-2.974915	0.514896
N	-0.844702	-3.693946	0.345911
C	-1.996936	-2.838403	0.219341
C	-1.350298	-1.498996	-0.034027
O	1.384300	-3.476956	0.836911
S	-2.964228	-3.267099	-1.280906
C	-4.024958	-1.800987	-1.217812
C	-3.245035	-0.483027	-1.276333
N	-2.046731	-0.428586	-0.438995
C	-2.829723	-2.844717	1.500263
H	3.167663	1.318885	-0.123822
H	4.861585	-1.096844	-0.837479
C	-4.225709	0.632989	-0.934279
N	-4.096774	1.234703	0.249898
C	-5.090535	2.158866	0.770147
C	-5.080026	3.530833	0.098599
O	-5.116170	0.922268	-1.737008
O	-5.260416	3.539175	-1.218624
O	-4.953293	4.556471	0.716818
H	3.707425	1.292001	-3.807859
H	3.720688	2.315658	-2.379578
H	5.595277	-0.009060	-2.946437
H	5.569969	1.844669	-0.529529
H	6.720872	0.549512	-0.844422
H	3.086830	-0.677514	-2.425088
H	1.939452	0.636422	-2.185521
H	6.128093	-0.405013	1.384384
H	4.471833	-0.665952	3.105916
H	2.260917	-1.397971	2.304690
H	2.617924	-1.662445	0.055660
H	3.577080	1.975895	2.111825
H	2.787327	3.521030	3.950697
H	1.111378	3.048638	3.661230
H	2.011766	3.942320	2.435361
H	-0.031510	-0.023330	2.457988
H	0.813256	-0.372909	3.953938
H	0.219269	1.260117	3.639310
H	6.110379	2.059213	-4.237386
H	6.128654	2.994345	-2.737622
H	7.354185	1.752102	-3.018201
H	-0.930586	-4.596063	0.788242
H	-4.677292	-1.845692	-2.087894
H	-4.658925	-1.848530	-0.332964
H	-2.938922	-0.310987	-2.311561
H	-1.426984	0.361840	-0.615318
H	-3.654585	-2.135088	1.465686
H	-3.235430	-3.843423	1.664307
H	-2.188955	-2.577332	2.343038
H	-3.330632	0.962687	0.847071
H	-4.903777	2.304140	1.829605
H	-6.087356	1.726156	0.645396
H	-5.362646	2.618428	-1.557865

ωB97X Energy = -1950.63970272 a.u.

(3*R*,4*S*,5*R*,8*R*,10*S*,19*R*,24*R*)-**3**, Conf P

C	-4.772290	-0.216032	-2.718712
C	-6.155799	-0.117373	-2.075452
C	-6.124165	-0.696214	-0.660970
C	-5.033007	-0.046194	0.189127
C	-3.656914	-0.241230	-0.458065
C	-3.671867	0.392441	-1.850642
C	-5.049134	-0.523171	1.613095
C	-3.955069	-0.659900	2.350198
C	-2.558280	-0.341372	1.882132
C	-2.579194	0.326987	0.468214
C	-1.642224	-1.554803	2.012942
C	-0.542362	-1.450359	2.760021
C	0.517387	-2.481926	3.001618
C	-2.039100	-2.800183	1.267434
C	-1.177369	0.262820	-0.101773
C	-7.219844	-0.791814	-2.931636
C	-0.169974	1.191890	0.388326
O	-0.861354	-0.592635	-0.929729
C	-0.329711	2.349075	1.286462

N	0.896186	2.968865	1.350796
C	1.951253	2.164940	0.778472
C	1.148086	1.137826	0.011762
O	-1.325120	2.751384	1.869525
S	3.070907	3.132663	-0.328858
C	3.024680	2.134714	-1.851372
C	2.920335	0.636884	-1.606063
N	1.705058	0.304064	-0.872417
C	2.771361	1.505164	1.892714
H	-3.493277	-1.318105	-0.579092
H	-5.239537	1.035297	0.200475
C	4.208335	0.037593	-1.028355
N	4.057358	-1.005904	-0.193962
C	5.204850	-1.708053	0.314716
C	5.981864	-2.504791	-0.716447
O	5.302610	0.454640	-1.374832
O	5.285284	-2.784561	-1.818323
O	7.112825	-2.876850	-0.532237
H	-4.787321	0.267938	-3.700306
H	-4.543290	-1.275279	-2.895660
H	-6.407136	0.948310	-1.987614
H	-5.946290	-1.778972	-0.713959
H	-7.099207	-0.559249	-0.180650
H	-3.837665	1.472813	-1.747600
H	-2.704862	0.261611	-2.338788
H	-6.020174	-0.747887	2.049800
H	-4.034466	-1.015633	3.375006
H	-2.159127	0.415728	2.563376
H	-2.806124	1.382691	0.647120
H	-0.361826	-0.493260	3.248496
H	0.297245	-3.441147	2.534649
H	0.659512	-2.648787	4.072616
H	1.477903	-2.133257	2.608467
H	-3.106687	-2.997071	1.390110
H	-1.852586	-2.679304	0.196693
H	-1.492540	-3.678654	1.607379
H	-7.261832	-0.351096	-3.930999
H	-7.003783	-1.858843	-3.045077
H	-8.210542	-0.697213	-2.479558
H	1.090630	3.598983	2.113302
H	2.189858	2.449133	-2.475869
H	3.957374	2.349458	-2.368894
H	2.821165	0.160720	-2.585219
H	1.026586	-0.310495	-1.314187
H	3.544087	0.852204	1.492251
H	3.255444	2.281907	2.486504
H	2.109862	0.924239	2.540071
H	3.130839	-1.339421	0.016975
H	4.878857	-2.407062	1.085510
H	5.910791	-1.014343	0.772380
H	5.848352	-3.305078	-2.408610

ωB97X Energy = -1950.63964812 a.u.

(3*R*,4*S*,5*R*,8*R*,10*S*,19*R*,24*R*)-**3**, Conf Q

C	4.528267	1.313991	-2.530629
C	5.939559	1.140444	-1.967913
C	5.913487	1.193562	-0.440262
C	4.919887	0.187617	0.139659
C	3.507248	0.461649	-0.389920
C	3.521279	0.343249	-1.915144
C	4.954332	0.137225	1.640259
C	3.883604	-0.090340	2.389231
C	2.497816	-0.338759	1.851141
C	2.520172	-0.485572	0.295065
C	1.507377	0.685833	2.398009
C	0.450279	0.253526	3.086674
C	-0.659710	1.062120	3.686746
C	1.788350	2.136378	2.112513
C	1.094372	-0.371043	-0.202468
C	6.901584	2.169965	-2.546674
C	0.205365	-1.507289	-0.054329
O	0.661204	0.684690	-0.670768
C	0.520672	-2.893630	0.313320
N	-0.642762	-3.626975	0.151055
C	-1.793540	-2.772960	-0.005191
C	-1.145477	-1.449507	-0.323268
O	1.577750	-3.388304	0.672904
S	-2.762717	-3.247139	-1.490378
C	-3.868489	-1.815531	-1.416251
C	-3.150791	-0.449508	-1.414313
N	-1.824488	-0.424933	-0.816024
C	-2.622387	-2.715337	1.277216
H	3.248590	1.495429	-0.134573
H	5.221080	-0.805239	-0.229517
C	-4.062154	0.529182	-0.673439
N	-4.802232	1.344702	-1.430366
C	-5.884170	2.149894	-0.887162
C	-5.423526	3.368344	-0.088437
O	-4.130791	0.523835	0.556271
O	-4.619215	3.124608	0.941513
O	-5.789468	4.486944	-0.343013
H	4.549092	1.194971	-3.618541
H	4.197305	2.342452	-2.334627
H	6.291893	0.140222	-2.254514
H	5.637522	2.205779	-0.114533
H	6.915694	0.996738	-0.044037
H	3.784674	-0.686781	-2.189296
H	2.527282	0.538298	-2.321280
H	5.921525	0.280098	2.118157
H	3.975990	-0.110872	3.472780
H	2.173465	-1.309808	2.237885
H	2.847161	-1.512386	0.103911
H	0.350849	-0.822742	3.223448
H	-0.511834	2.136295	3.582273
H	-0.773115	0.836128	4.750328
H	-1.611471	0.810428	3.208445
H	1.558428	2.369994	1.069377
H	1.202339	2.803117	2.743302
H	2.845588	2.361972	2.270210
H	6.946320	2.101289	-3.636680
H	6.581311	3.184221	-2.288021

H	7.913538	2.029348	-2.158086
H	-0.733863	-4.496237	0.654534
H	-4.504770	-1.867423	-2.298120
H	-4.510394	-1.915106	-0.543014
H	-3.026253	-0.121783	-2.448698
H	-1.238095	0.381109	-1.019875
H	-3.401625	-1.955942	1.233773
H	-3.077847	-3.688688	1.463220
H	-1.960690	-2.466108	2.109763
H	-4.668396	1.351355	-2.428690
H	-6.502115	1.528257	-0.233094
H	-6.496644	2.503076	-1.711015
H	-4.438957	2.158026	1.016333

ωB97X Energy = -1950.63959250 a.u.

(3*R*,4*S*,5*R*,8*R*,10*S*,19*R*,24*R*)-**3**, Conf R

C	4.120521	2.720791	-1.785406
C	5.610759	2.477768	-1.543537
C	5.824492	1.783358	-0.198585
C	4.993156	0.505264	-0.086443
C	3.501699	0.821811	-0.248804
C	3.280647	1.454544	-1.623890
C	5.267856	-0.255961	1.178368
C	4.343435	-0.949961	1.828685
C	2.905816	-1.083402	1.396042
C	2.680935	-0.448321	-0.014196
C	1.959476	-0.596837	2.490055
C	1.049891	-1.443560	2.974052
C	0.004453	-1.187492	4.016791
C	2.110199	0.828347	2.948927
C	1.190167	-0.262062	-0.215748
C	6.408563	3.772164	-1.636110
C	0.369456	-1.409655	-0.545137
O	0.657930	0.839251	-0.051992
C	0.775402	-2.773282	-0.905143
N	-0.372381	-3.448639	-1.285429
C	-1.563560	-2.723075	-0.919656
C	-1.006573	-1.350825	-0.637532
O	1.885710	-3.282594	-0.919835
S	-2.693317	-2.551579	-2.354760
C	-3.821015	-1.402868	-1.523527
C	-3.152040	-0.127695	-0.984450
N	-1.776220	-0.280716	-0.524819
C	-2.228292	-3.329908	0.315455
H	3.232214	1.563738	0.511366
H	5.285611	-0.140460	-0.929177
C	-4.014295	0.386860	0.177197
N	-4.750447	1.473673	-0.091482
C	-5.653520	2.030460	0.883281
C	-6.360285	3.219269	0.284881
O	-4.039554	-0.192400	1.253003
O	-7.216890	3.761149	1.146555
O	-6.174575	3.630929	-0.832332
H	3.973977	3.146053	-2.783298
H	3.768308	3.475190	-1.069439
H	5.968470	1.794358	-2.325568
H	5.546180	2.468621	0.613669
H	6.886553	1.550096	-0.065099
H	3.556856	0.727026	-2.398295
H	2.225554	1.692107	-1.768758
H	6.288681	-0.239464	1.555208
H	4.606767	-1.477591	2.742712
H	2.706092	-2.152194	1.274718
H	3.012399	-1.204821	-0.732519
H	1.032781	-2.448532	2.553624
H	0.090325	-0.203752	4.476922
H	0.056174	-1.939522	4.808429
H	-0.994643	-1.262746	3.576151
H	1.705133	1.514157	2.199577
H	1.595442	1.016871	3.890018
H	3.164703	1.080455	3.083192
H	6.278171	4.249261	-2.610919
H	6.079682	4.481617	-0.870161
H	7.476591	3.592151	-1.488708
H	-0.371075	-4.456257	-1.240645
H	-4.568586	-1.113026	-2.260408
H	-4.334973	-1.932243	-0.723800
H	-3.125839	0.616198	-1.784204
H	-1.246395	0.572159	-0.359379
H	-3.037484	-2.708176	0.696187
H	-2.617910	-4.319528	0.073815
H	-1.479083	-3.424913	1.104742
H	-4.691762	1.942784	-0.983923
H	-5.121413	2.353432	1.781797
H	-6.400003	1.297042	1.197772
H	-7.646114	4.519884	0.726041

ωB97X Energy = -1950.63954789 a.u.

(3*R*,4*S*,5*R*,8*R*,10*S*,19*R*,24*S*)-**3**, Conf A

C	3.960356	2.643998	-0.962950
C	5.362105	2.067276	-1.165801
C	5.538189	0.792345	-0.340923
C	4.432401	-0.222658	-0.628548
C	3.056981	0.381316	-0.319335
C	2.855176	1.615792	-1.200465
C	4.644626	-1.526941	0.083841
C	3.653874	-2.275163	0.549682
C	2.189568	-1.938467	0.425267
C	1.981071	-0.694411	-0.495942
C	1.539865	-1.872911	1.804352
C	0.567150	-2.738614	2.094517
C	-0.190188	-2.894427	3.377902
C	2.071842	-0.849274	2.770180
C	0.566299	-0.185832	-0.311690
C	6.439345	3.094703	-0.842703
C	-0.530159	-0.866854	-0.974150
O	0.322890	0.779308	0.417970
C	-0.490790	-2.014558	-1.897199
N	-1.780738	-2.220455	-2.326647

C	-2.745306	-1.419496	-1.618666
C	-1.844749	-0.479591	-0.853997
O	0.460009	-2.688354	-2.263110
S	-3.783755	-0.446205	-2.807911
C	-3.973125	1.149946	-1.936312
C	-3.707272	1.033668	-0.443260
N	-2.345071	0.564924	-0.198764
C	-3.600738	-2.294020	-0.701602
H	3.063722	0.710856	0.725675
H	4.463619	-0.429346	-1.709726
C	-3.915005	2.392027	0.245075
N	-3.916382	2.407300	1.596339
C	-3.637598	1.319994	2.500638
C	-2.167086	1.052228	2.777660
O	-4.097433	3.404501	-0.408242
O	-1.360943	2.003466	2.307092
O	-1.784264	0.071806	3.362060
H	3.818919	3.506535	-1.621718
H	3.881372	3.020381	0.065582
H	5.457688	1.788677	-2.224009
H	5.528638	1.044510	0.728325
H	6.516128	0.345493	-0.550824
H	2.858565	1.306064	-2.253764
H	1.883348	2.071425	-1.004111
H	5.671950	-1.867152	0.198356
H	3.874685	-3.209854	1.060117
H	1.713215	-2.773968	-0.095156
H	2.052816	-1.077710	-1.518844
H	0.269625	-3.430919	1.307420
H	-1.247011	-2.657784	3.228353
H	0.177261	-2.246908	4.172693
H	-0.137500	-3.929269	3.727107
H	3.164429	-0.866845	2.788017
H	1.773211	0.157429	2.464684
H	1.712241	-1.010622	3.785155
H	6.328520	3.992022	-1.456879
H	6.377392	3.398326	0.207157
H	7.439323	2.688400	-1.015052
H	-2.038485	-3.102664	-2.739767
H	-3.297285	1.884667	-2.369565
H	-4.997638	1.477152	-2.101499
H	-4.422842	0.334138	-0.003310
H	-1.646144	1.194353	0.187493
H	-4.319556	-1.713309	-0.127099
H	-4.158715	-3.008211	-1.308327
H	-2.953636	-2.841224	-0.012495
H	-4.008158	3.329220	1.996156
H	-4.068876	0.383183	2.146787
H	-4.115829	1.530725	3.457833
H	-0.442633	1.692861	2.355713

ωB97X Energy = -1950.63430809 a.u.

(3*R*,4*S*,5*R*,8*R*,10*S*,19*R*,24*S*)-**3**, Conf B

C	-4.133578	-0.447061	2.701233
C	-5.531907	-0.636136	2.112276
C	-5.619947	0.015716	0.732401
C	-4.514367	-0.482328	-0.198344
C	-3.134698	-0.189232	0.403577
C	-3.025152	-0.901640	1.752951
C	-4.643060	0.063269	-1.591100
C	-3.603105	0.346282	-2.363686
C	-2.160973	0.156215	-1.967703
C	-2.047445	-0.586764	-0.597954
C	-1.401821	1.476072	-2.075759
C	-0.387807	1.561710	-2.938150
C	0.480756	2.746238	-3.236447
C	-1.865325	2.612023	-1.204828
C	-0.635393	-0.408325	-0.078169
C	-6.607343	-0.103856	3.050763
C	0.440909	-1.197148	-0.655035
O	-0.372597	0.419906	0.793860
C	0.368892	-2.314652	-1.611204
N	1.643469	-2.815646	-1.727067
C	2.630849	-2.006757	-1.061231
C	1.757116	-1.040240	-0.298969
O	-0.595770	-2.769411	-2.207786
S	3.617535	-3.020734	0.141056
C	3.746337	-1.886894	1.568642
C	3.605193	-0.430074	1.155456
N	2.283223	-0.187075	0.580476
C	3.531392	-1.305600	-2.077520
H	-3.071709	0.889609	0.585792
H	-4.619525	-1.576615	-0.262351
C	3.802169	0.496826	2.362675
N	3.967617	1.812358	2.107655
C	3.931190	2.492602	0.841324
C	2.579154	3.112085	0.540850
O	3.824115	0.054574	3.498390
O	2.615568	3.782086	-0.613508
O	1.604325	3.027212	1.238634
H	-4.058989	-0.982607	3.652869
H	-3.990901	0.617492	2.929390
H	-5.694163	-1.714049	1.976858
H	-5.538469	1.106067	0.839144
H	-6.600134	-0.185975	0.286690
H	-3.098767	-1.984973	1.590769
H	-2.051758	-0.710377	2.207103
H	-5.650615	0.209464	-1.975443
H	-3.763282	0.742932	-3.363673
H	-1.712168	-0.513411	-2.707058
H	-2.173094	-1.648936	-0.831788
H	-0.139211	0.664684	-3.504129
H	1.506879	2.566135	-2.900935
H	0.127122	3.663265	-2.764504
H	0.530207	2.923438	-4.313747
H	-2.954285	2.698886	-1.229640
H	-1.576827	2.434383	-0.165048
H	-1.442828	3.568278	-1.510355
H	-6.560718	-0.595741	4.025733
H	-6.478195	0.971095	3.211728
H	-7.607450	-0.263108	2.639336

H	1.887907	-3.409697	-2.503115
H	2.988084	-2.134282	2.309047
H	4.729435	-2.053086	2.005203
H	4.382120	-0.194802	0.423619
H	1.591072	0.360154	1.090068
H	4.267301	-0.662673	-1.598427
H	4.070449	-2.054626	-2.658826
H	2.918470	-0.704127	-2.752622
H	4.055270	2.385153	2.934274
H	4.191645	1.826147	0.019440
H	4.676175	3.289578	0.834480
H	1.742889	4.167688	-0.777255

ωB97X Energy = -1950.63411206 a.u.

(3*R*,4*S*,5*R*,8*R*,10*S*,19*R*,24*S*)-**3**, Conf C

C	4.416090	0.453137	-2.631953
C	5.802987	0.304587	-2.005831
C	5.760870	0.687202	-0.526417
C	4.687404	-0.096543	0.228016
C	3.305609	0.144602	-0.391504
C	3.333438	-0.295640	-1.856149
C	4.691413	0.190888	1.702429
C	3.594347	0.207179	2.447980
C	2.205682	-0.077959	1.936042
C	2.249029	-0.571193	0.452232
C	1.264085	1.093875	2.202585
C	0.162577	0.882903	2.924292
C	-0.918909	1.861226	3.270823
C	1.637294	2.427724	1.613390
C	0.842342	-0.490961	-0.099447
C	6.847395	1.115671	-2.761914
C	-0.122222	-1.503914	0.288980
O	0.480480	0.450075	-0.808764
C	0.080826	-2.775726	0.998451
N	-1.145747	-3.403861	1.042673
C	-2.225233	-2.548898	0.616318
C	-1.463562	-1.393647	0.017747
O	1.102299	-3.254663	1.467013
S	-3.233870	-3.363804	-0.711242
C	-3.617911	-1.959422	-1.823164
C	-3.454857	-0.612929	-1.136518
N	-2.085651	-0.439674	-0.665499
C	-3.085227	-2.134159	1.810720
H	3.112276	1.222964	-0.367552
H	4.921988	-1.164551	0.098295
C	-3.794892	0.544585	-2.083483
N	-3.800294	1.774865	-1.533686
C	-3.667157	2.116683	-0.135921
C	-3.961989	3.586533	0.042172
O	-4.029335	0.354168	-3.264866
O	-3.851104	3.946524	1.317928
O	-4.254439	4.337682	-0.851894
H	4.442248	0.107160	-3.670087
H	4.158911	1.520229	-2.664497
H	6.081983	-0.756346	-2.063108
H	5.556669	1.762674	-0.433489
H	6.740002	0.511248	-0.067624
H	3.527164	-1.375393	-1.900717
H	2.361994	-0.125152	-2.322973
H	5.657098	0.378538	2.167663
H	3.665780	0.428097	3.510539
H	1.816938	-0.918495	2.519011
H	2.515153	-1.631729	0.504289
H	-0.001432	-0.125886	3.301800
H	-0.754732	2.850282	2.844245
H	-1.017038	1.969242	4.354542
H	-1.884641	1.497260	2.905245
H	2.705554	2.617436	1.740285
H	1.429891	2.444056	0.539850
H	1.092654	3.250264	2.074827
H	6.894879	0.819971	-3.813132
H	6.606226	2.182769	-2.724970
H	7.842076	0.982822	-2.328555
H	-1.304246	-4.134200	1.718722
H	-2.972534	-2.003889	-2.698471
H	-4.650659	-2.086328	-2.142020
H	-4.147697	-0.553514	-0.292612
H	-1.458801	0.228254	-1.108186
H	-3.892246	-1.461326	1.527133
H	-3.532722	-3.024207	2.255108
H	-2.457680	-1.640459	2.556697
H	-4.022340	2.541261	-2.156709
H	-2.660451	1.931492	0.248574
H	-4.366255	1.559613	0.493625
H	-4.042048	4.892271	1.395112

ωB97X Energy = -1950.63312820 a.u.

(3*R*,4*S*,5*R*,8*R*,10*S*,19*R*,24*S*)-**3**, Conf D

C	3.724205	-0.284933	-2.849343
C	5.121523	-0.756651	-2.445410
C	5.407284	-0.390854	-0.988693
C	4.311828	-0.903074	-0.053618
C	2.949341	-0.326430	-0.454758
C	2.635316	-0.759045	-1.888048
C	4.619873	-0.649646	1.393769
C	3.700611	-0.325338	2.293153
C	2.231786	-0.172460	1.998779
C	1.905576	-0.750076	0.581585
C	1.745065	1.257075	2.241659
C	2.485765	2.309211	1.896925
C	2.151437	3.762844	2.047241
C	0.408605	1.353289	2.928577
C	0.489879	-0.387956	0.190927
C	6.187759	-0.200423	-3.380420
C	-0.613085	-1.228490	0.617946
O	0.250352	0.627811	-0.466555
C	-0.584069	-2.476811	1.398888
N	-1.876709	-2.945243	1.441249
C	-2.834203	-2.003773	0.919518
C	-1.925544	-0.972154	0.296388

O	0.361114	-3.042252	1.926547
S	-3.872651	-2.783946	-0.404136
C	-4.034039	-1.420968	-1.612668
C	-3.774161	-0.056239	-0.992232
N	-2.416831	0.010067	-0.454758
C	-3.692100	-1.431226	2.048295
H	3.024462	0.767837	-0.440993
H	4.258264	-1.994139	-0.193191
C	-3.969577	1.052791	-2.038875
N	-3.948584	2.331276	-1.601682
C	-3.667665	2.821136	-0.275888
C	-2.199038	3.041638	0.051057
O	-4.162374	0.779965	-3.210949
O	-1.391442	2.844501	-0.989808
O	-1.820293	3.355064	1.150065
H	3.502251	-0.623324	-3.866315
H	3.719856	0.812823	-2.877480
H	5.136993	-1.852640	-2.517868
H	5.481997	0.701105	-0.893912
H	6.376667	-0.801460	-0.685601
H	2.560115	-1.853730	-1.924104
H	1.670527	-0.360833	-2.205768
H	5.657375	-0.757349	1.704048
H	3.993077	-0.144752	3.324328
H	1.695449	-0.808891	2.711756
H	1.944447	-1.837772	0.691004
H	3.458715	2.111318	1.452030
H	2.869414	4.257545	2.707774
H	1.153001	3.934091	2.447972
H	2.216607	4.269350	1.080171
H	-0.335851	0.721426	2.436837
H	0.496029	0.987233	3.956840
H	0.010584	2.365586	2.955774
H	5.991459	-0.482749	-4.417984
H	6.210221	0.892810	-3.329908
H	7.181830	-0.568148	-3.113138
H	-2.142582	-3.623382	2.137653
H	-3.343456	-1.581924	-2.438308
H	-5.051530	-1.462591	-1.995896
H	-4.497908	0.119690	-0.191754
H	-1.711252	0.589726	-0.904046
H	-4.422951	-0.709384	1.689440
H	-4.237429	-2.244881	2.528089
H	-3.048721	-0.950016	2.788263
H	-4.040785	3.016898	-2.336581
H	-4.063281	2.152019	0.488600
H	-4.177914	3.774679	-0.136936
H	-0.471491	2.824924	-0.681220

ωB97X Energy = -1950.63272302 a.u.

(3*R*,4*S*,5*R*,8*R*,10*S*,19*R*,24*S*)-**3**, Conf E

C	-4.663485	-0.743146	2.452439
C	-6.018972	-0.600795	1.759160
C	-5.924444	0.395294	0.603330
C	-4.801383	0.027012	-0.366322
C	-3.454647	-0.015893	0.365241
C	-3.530435	-1.062026	1.478367
C	-4.754186	0.924851	-1.569227
C	-3.629025	1.270800	-2.180563
C	-2.253019	0.812201	-1.770357
C	-2.337054	-0.272819	-0.646904
C	-1.348100	2.002308	-1.459863
C	-0.213126	2.143148	-2.146060
C	0.836717	3.202297	-1.995430
C	-1.797939	2.946645	-0.377755
C	-0.959766	-0.404568	-0.033599
C	-7.110789	-0.209013	2.746518
C	0.067219	-1.129139	-0.759791
O	-0.671659	0.151563	1.028062
C	-0.046543	-1.991234	-1.945299
N	1.212074	-2.501004	-2.184937
C	2.231964	-1.863300	-1.390478
C	1.392211	-1.091793	-0.404001
O	-1.026323	-2.263151	-2.622119
S	3.234654	-3.121858	-0.467402
C	3.556487	-2.269673	1.123206
C	3.316542	-0.769600	1.043484
N	1.943514	-0.487898	0.643138
C	3.106173	-0.963214	-2.264493
H	-3.296063	0.962620	0.832319
H	-5.011607	-0.992660	-0.724977
C	3.587712	-0.093012	2.393228
N	3.514887	1.257839	2.415520
C	3.438686	2.155359	1.288555
C	4.739445	2.425489	0.552588
O	3.832501	-0.744480	3.392329
O	5.737161	1.616220	0.910619
O	4.837561	3.275463	-0.294260
H	-4.724560	-1.515886	3.225125
H	-4.434442	0.198329	2.969108
H	-6.277096	-1.577789	1.328604
H	-5.742531	1.402089	1.003483
H	-6.879787	0.435722	0.068713
H	-3.696275	-2.049352	1.027837
H	-2.583931	-1.112188	2.019309
H	-5.705188	1.286496	-1.955301
H	-3.663656	1.928691	-3.045994
H	-1.809788	0.312001	-2.636950
H	-2.552714	-1.213974	-1.162712
H	0.006506	1.393772	-2.905776
H	0.601337	3.933008	-1.222646
H	0.985596	3.737580	-2.937173
H	1.799890	2.746904	-1.742115
H	-1.638014	2.500720	0.607715
H	-1.264699	3.895700	-0.409155
H	-2.866099	3.155668	-0.470160
H	-7.198423	-0.943328	3.551461
H	-6.887956	0.761191	3.201756
H	-8.083259	-0.131287	2.253499
H	1.428516	-2.877679	-3.094387
H	2.921772	-2.700073	1.895341

H	4.597306	-2.459322	1.377964
H	4.013318	-0.334688	0.322194
H	1.273210	-0.092271	1.298109
H	3.881238	-0.453511	-1.695322
H	3.599356	-1.572986	-3.022515
H	2.480010	-0.217415	-2.760136
H	3.723691	1.667123	3.314120
H	3.060070	3.117201	1.631818
H	2.723367	1.798512	0.544295
H	6.517738	1.836266	0.382032

ωB97X Energy = -1950.63235423 a.u.

(3*R*,4*S*,5*R*,8*R*,10*S*,19*R*,24*S*)-**3**, Conf F

C	3.900296	-0.561504	-2.765920
C	5.293492	-0.921726	-2.247261
C	5.500999	-0.355899	-0.842299
C	4.381763	-0.788376	0.104086
C	3.020855	-0.312873	-0.419007
C	2.782563	-0.939647	-1.794308
C	4.621504	-0.361018	1.523224
C	3.658854	0.044063	2.340665
C	2.203096	0.133593	1.964368
C	1.952967	-0.636805	0.627146
C	1.699176	1.579133	1.991159
C	2.470053	2.582699	1.573730
C	2.136710	4.042373	1.505999
C	0.308427	1.763929	2.541641
C	0.534212	-0.396072	0.163589
C	6.381097	-0.453085	-3.205344
C	-0.542075	-1.187379	0.738391
O	0.265635	0.474625	-0.664306
C	-0.465897	-2.330354	1.664803
N	-1.738601	-2.839289	1.766761
C	-2.730005	-2.013082	1.128342
C	-1.861237	-1.018718	0.396633
O	0.499506	-2.794055	2.252659
S	-3.705785	-2.991503	-0.111935
C	-3.861511	-1.801109	-1.490684
C	-3.718630	-0.361135	-1.022683
N	-2.391035	-0.138081	-0.452420
C	-3.636164	-1.358164	2.170043
H	3.063677	0.776038	-0.546338
H	4.366485	-1.889613	0.093890
C	-3.934261	0.611500	-2.190209
N	-4.090570	1.916827	-1.882916
C	-4.016187	2.551664	-0.594705
C	-2.656993	3.168005	-0.320273
O	-3.981777	0.211999	-3.340840
O	-2.655558	3.802304	0.854386
O	-1.706988	3.108348	-1.054090
H	3.733760	-1.041494	-3.735483
H	3.863145	0.521469	-2.943543
H	5.349307	-2.016042	-2.170250
H	5.532937	0.741126	-0.890414
H	6.469316	-0.683936	-0.448563
H	2.737765	-2.031362	-1.686613
H	1.822240	-0.617438	-2.199245
H	5.645983	-0.411106	1.886965
H	3.902608	0.348046	3.355330
H	1.640859	-0.408527	2.732584
H	2.030308	-1.697650	0.883769
H	3.470724	2.332348	1.228409
H	2.140517	4.384886	0.467143
H	2.890308	4.633191	2.033597
H	1.162850	4.283175	1.930465
H	-0.449592	1.360076	1.863689
H	0.203135	1.228179	3.489140
H	0.066013	2.810044	2.721932
H	6.250008	-0.891648	-4.197945
H	6.354052	0.635755	-3.314365
H	7.374961	-0.728606	-2.843105
H	-1.980625	-3.449246	2.531262
H	-3.113092	-2.016503	-2.250872
H	-4.850149	-1.953045	-1.919593
H	-4.487234	-0.155870	-0.273209
H	-1.701853	0.424256	-0.950022
H	-4.382564	-0.708193	1.717871
H	-4.163686	-2.134863	2.725141
H	-3.029114	-0.774187	2.865240
H	-4.192633	2.519865	-2.686112
H	-4.244728	1.854400	0.210798
H	-4.764539	3.343204	-0.534369
H	-1.782310	4.195268	0.994560

ωB97X Energy = -1950.63192968 a.u.

(3*R*,4*S*,5*R*,8*R*,10*S*,19*S*,24*R*)-**3**, Conf A

C	4.298433	2.284951	-0.535621
C	5.647993	1.571905	-0.629140
C	5.610863	0.258808	0.152476
C	4.442675	-0.625885	-0.282413
C	3.111483	0.108659	-0.084894
C	3.126015	1.386340	-0.926580
C	4.446741	-1.962706	0.401461
C	3.342012	-2.616964	0.733572
C	1.941086	-2.134416	0.458004
C	1.954051	-0.836721	-0.415518
C	1.125377	-2.044943	1.744846
C	-0.007703	-2.742599	1.835573
C	-0.982111	-2.787075	2.972984
C	1.656156	-1.153696	2.834935
C	0.588318	-0.190772	-0.316737
C	6.785986	2.468381	-0.158235
C	-0.522852	-0.754969	-1.057871
O	0.389912	0.773123	0.427991
C	-0.531165	-1.884438	-2.002657
N	-1.850937	-2.160039	-2.275209
C	-2.759377	-1.190343	-1.722471
C	-1.827969	-0.368912	-0.864861
O	0.403949	-2.504137	-2.485362

S	-3.974604	-2.029491	-0.595034
C	-4.264264	-0.754574	0.689788
C	-3.726028	0.612351	0.293903
N	-2.303559	0.543231	-0.020793
C	-3.445589	-0.404054	-2.837546
H	3.043088	0.398322	0.970009
H	4.562272	-0.805676	-1.362086
C	-3.954138	1.635322	1.417492
N	-3.684161	2.928978	1.134170
C	-3.125333	3.477166	-0.077215
C	-1.610208	3.416802	-0.190667
O	-4.382432	1.290342	2.504953
O	-1.007112	3.144027	0.965396
O	-1.027417	3.598634	-1.228619
H	4.311303	3.181152	-1.163916
H	4.154432	2.629451	0.497034
H	5.819907	1.321607	-1.684778
H	5.518905	0.475452	1.225523
H	6.555272	-0.279937	0.018635
H	3.207704	1.113263	-1.986702
H	2.188885	1.933750	-0.809482
H	5.416706	-2.408184	0.613097
H	3.413550	-3.580688	1.232627
H	1.457228	-2.901089	-0.154064
H	2.075627	-1.178146	-1.448271
H	-0.289148	-3.351429	0.976863
H	-0.685552	-2.166185	3.817418
H	-1.113734	-3.811339	3.332114
H	-1.966565	-2.445403	2.637138
H	2.729590	-1.307037	2.968185
H	1.509150	-0.101784	2.574669
H	1.168273	-1.333860	3.791703
H	6.823924	3.396635	-0.734158
H	6.654156	2.733432	0.895557
H	7.752933	1.968831	-0.259247
H	-2.096684	-2.697867	-3.091059
H	-5.338953	-0.690712	0.847469
H	-3.793596	-1.074970	1.617853
H	-4.272489	0.974753	-0.579887
H	-1.612555	0.927256	0.618811
H	-4.004737	-1.097379	-3.467047
H	-4.147945	0.335380	-2.459588
H	-2.691883	0.099803	-3.446164
H	-3.803392	3.556082	1.915540
H	-3.416491	4.525675	-0.150238
H	-3.528860	2.986257	-0.962930
H	-0.073976	2.939620	0.792230

ωB97X Energy = -1950.63467826 a.u.

(3*R*,4*S*,5*R*,8*R*,10*S*,19*S*,24*R*)-**3**, Conf B

C	-4.262370	-2.436122	-0.343772
C	-5.634500	-1.778752	-0.491724
C	-5.643413	-0.413407	0.195369
C	-4.506363	0.478660	-0.302760
C	-3.149547	-0.192297	-0.061062
C	-3.120563	-1.530607	-0.802360
C	-4.554708	1.857242	0.290403
C	-3.471367	2.572110	0.562676
C	-2.056031	2.125652	0.298544
C	-2.026481	0.764010	-0.471051
C	-1.218078	2.175720	1.572862
C	-0.131902	2.949355	1.594592
C	0.845417	3.146075	2.714134
C	-1.683315	1.343765	2.737064
C	-0.639407	0.166933	-0.335720
C	-6.743872	-2.677854	0.038639
C	0.453253	0.716447	-1.119693
O	-0.406529	-0.732408	0.472113
C	0.428929	1.807522	-2.108341
N	1.738212	2.141594	-2.355689
C	2.680838	1.255107	-1.727438
C	1.767893	0.399754	-0.883637
O	-0.524315	2.356468	-2.640393
S	3.799206	2.205215	-0.584032
C	3.978345	1.050743	0.823496
C	3.646806	-0.383045	0.440305
N	2.264097	-0.487587	-0.020019
C	3.467190	0.471779	-2.775625
H	-3.065271	-0.398053	1.012302
H	-4.633636	0.581312	-1.391654
C	3.848560	-1.323429	1.636688
N	3.841737	-2.649554	1.382758
C	3.612686	-3.318378	0.130375
C	2.165194	-3.728056	-0.068332
O	4.023042	-0.884296	2.760306
O	2.000548	-4.320567	-1.252700
O	1.281030	-3.558970	0.728332
H	-4.242932	-3.378302	-0.900584
H	-4.109619	-2.693891	0.712564
H	-5.809666	-1.609028	-1.562777
H	-5.544549	-0.551868	1.280679
H	-6.605506	0.082609	0.026470
H	-3.209474	-1.342640	-1.880480
H	-2.165950	-2.034028	-0.641484
H	-5.538197	2.279934	0.485838
H	-3.573331	3.563608	0.998194
H	-1.615913	2.861397	-0.380660
H	-2.164868	1.019198	-1.526671
H	0.096528	3.513627	0.690887
H	0.703200	2.444073	3.535038
H	0.778353	4.160324	3.118517
H	1.867593	3.026463	2.343095
H	-2.755300	1.480455	2.899482
H	-1.519100	0.280902	2.540483
H	-1.167562	1.600393	3.661207
H	-6.751681	-3.642541	-0.475232
H	-6.604827	-2.868963	1.107396
H	-7.726012	-2.216725	-0.094001
H	1.985012	2.683044	-3.168459
H	5.014168	1.114636	1.151264
H	3.332546	1.366889	1.641244

H	4.328377	-0.703330	-0.351246
H	1.557417	-0.934040	0.563544
H	4.029119	1.169132	-3.398245
H	4.178874	-0.218709	-2.328268
H	2.775212	-0.089678	-3.406566
H	3.936074	-3.228574	2.204131
H	4.223814	-4.221152	0.086547
H	3.918917	-2.704813	-0.716305
H	1.073199	-4.581660	-1.344167

ωB97X Energy = -1950.63403819 a.u.

(3*R*,4*S*,5*R*,8*R*,10*S*,19*S*,24*R*)-**3**, Conf C

C	4.240187	2.985107	-0.288635
C	5.677671	2.495477	-0.468812
C	5.850032	1.114529	0.163422
C	4.818599	0.118667	-0.366706
C	3.396930	0.618748	-0.083530
C	3.205392	1.971005	-0.773402
C	5.034523	-1.270764	0.159170
C	4.046090	-2.118720	0.408405
C	2.583967	-1.824278	0.190157
C	2.383017	-0.443129	-0.516146
C	1.793919	-2.024982	1.480486
C	0.807563	-2.922696	1.494531
C	-0.099544	-3.291810	2.628666
C	2.193055	-1.190327	2.667065
C	0.939444	-0.020585	-0.329841
C	6.681246	3.495881	0.090025
C	-0.099075	-0.667672	-1.110489
O	0.622277	0.816682	0.517686
C	0.019816	-1.694734	-2.158023
N	-1.252262	-2.161287	-2.397679
C	-2.270703	-1.413589	-1.708622
C	-1.434743	-0.519426	-0.828507
O	1.013127	-2.105063	-2.738926
S	-3.266343	-2.535207	-0.610386
C	-3.589371	-1.471611	0.844143
C	-3.364714	0.004466	0.554818
N	-1.997782	0.244841	0.103528
C	-3.149985	-0.652222	-2.697942
H	3.306882	0.774204	0.997777
H	4.943065	0.081250	-1.460180
C	-3.620317	0.850089	1.810117
N	-3.604648	2.192025	1.646720
C	-3.619050	2.927699	0.405748
C	-4.979968	3.095594	-0.248376
O	-3.795694	0.332897	2.898715
O	-5.883328	2.208980	0.174382
O	-5.200571	3.925259	-1.091655
H	4.107012	3.938095	-0.810309
H	4.069643	3.185647	0.777406
H	5.860625	2.387675	-1.546551
H	5.745317	1.197803	1.253792
H	6.861227	0.739918	-0.029646
H	3.304008	1.832855	-1.858064
H	2.200862	2.354618	-0.588701
H	6.063602	-1.584870	0.322016
H	4.268171	-3.111579	0.792999
H	2.211640	-2.575683	-0.512075
H	2.525785	-0.635491	-1.584477
H	0.615298	-3.465198	0.569298
H	-1.144832	-3.145520	2.339152
H	0.080948	-2.710542	3.532010
H	0.007550	-4.351026	2.878519
H	3.277746	-1.204747	2.798782
H	1.899753	-0.147730	2.516962
H	1.736046	-1.539385	3.591679
H	6.573323	4.473049	-0.387766
H	6.530647	3.631145	1.165713
H	7.708705	3.156368	-0.064345
H	-1.456852	-2.666679	-3.244876
H	-4.626735	-1.633361	1.130713
H	-2.947463	-1.781676	1.667071
H	-4.074972	0.331347	-0.208452
H	-1.329238	0.729972	0.698690
H	-3.661575	-1.365772	-3.345027
H	-3.909115	-0.052109	-2.199913
H	-2.526851	0.001120	-3.312022
H	-3.792887	2.713288	2.490490
H	-2.964095	2.461665	-0.333978
H	-3.218777	3.924838	0.579786
H	-6.710506	2.356410	-0.306232

ωB97X Energy = -1950.63278527 a.u.

(3*R*,4*S*,5*R*,8*R*,10*S*,19*S*,24*R*)-**3**, Conf D

C	3.978203	2.325046	-0.942688
C	5.342623	1.675882	-1.179041
C	5.473718	0.392829	-0.357661
C	4.305459	-0.558731	-0.614383
C	2.975509	0.119109	-0.263371
C	2.813577	1.357193	-1.148103
C	4.466551	-1.879386	0.081098
C	3.461765	-2.546273	0.633011
C	2.030357	-2.077706	0.642285
C	1.850138	-0.910423	-0.383663
C	1.537523	-1.783921	2.061442
C	2.336747	-1.219302	2.965852
C	2.022273	-0.858205	4.386172
C	0.126969	-2.229499	2.343960
C	0.464442	-0.323843	-0.247916
C	6.478741	2.646743	-0.883788
C	-0.645016	-0.948398	-0.943289
O	0.251051	0.654730	0.473347
C	-0.642995	-2.125685	-1.830218
N	-1.958679	-2.406439	-2.113401
C	-2.872001	-1.406325	-1.626581
C	-1.953576	-0.554223	-0.784938
O	0.296719	-2.777546	-2.258230
S	-4.134470	-2.181718	-0.506774
C	-4.409005	-0.870181	0.742988

C	-3.866366	0.480790	0.301699
N	-2.439016	0.394653	0.010270
C	-3.516061	-0.656289	-2.791168
H	3.026720	0.453849	0.780070
H	4.292139	-0.759687	-1.697134
C	-4.106612	1.544522	1.384362
N	-3.832830	2.827273	1.058382
C	-3.255858	3.333010	-0.162824
C	-1.738929	3.271646	-0.249428
O	-4.548505	1.239015	2.478112
O	-1.155760	3.032323	0.924127
O	-1.138846	3.424118	-1.282204
H	3.866161	3.192968	-1.600036
H	3.945216	2.706373	0.086510
H	5.397569	1.392816	-2.238961
H	5.508228	0.644101	0.711214
H	6.419862	-0.105485	-0.595549
H	2.772849	1.044044	-2.199514
H	1.873017	1.867158	-0.931821
H	5.468362	-2.303443	0.108334
H	3.649709	-3.494384	1.130039
H	1.420478	-2.907638	0.267345
H	1.906732	-1.377211	-1.370990
H	3.354010	-0.988950	2.656917
H	2.677157	-1.401431	5.073343
H	0.991589	-1.069299	4.667966
H	2.204512	0.206439	4.556281
H	-0.208186	-1.973547	3.347403
H	-0.582462	-1.794848	1.634439
H	0.051408	-3.315342	2.229683
H	6.398774	3.548652	-1.496084
H	6.458686	2.952993	0.166941
H	7.452143	2.190033	-1.080386
H	-2.194148	-2.990511	-2.899916
H	-5.483045	-0.797568	0.901156
H	-3.935678	-1.164478	1.677792
H	-4.400919	0.809133	-0.592844
H	-1.755936	0.813598	0.636555
H	-4.070938	-1.366311	-3.405718
H	-4.214580	0.109239	-2.460540
H	-2.738556	-0.190621	-3.400211
H	-3.961294	3.481305	1.815916
H	-3.546631	4.377827	-0.277405
H	-3.645184	2.811544	-1.037443
H	-0.218905	2.826347	0.774199

ωB97X Energy = -1950.63272284 a.u.

(3*R*,4*S*,5*R*,8*R*,10*S*,19*S*,24*S*)-**3**, Conf A

C	4.330211	3.104815	-0.576256
C	5.785937	2.644470	-0.661112
C	5.981840	1.350691	0.129266
C	4.991886	0.268007	-0.299940
C	3.550250	0.755424	-0.112772
C	3.338652	2.008789	-0.963801
C	5.232060	-1.039220	0.398484
C	4.261266	-1.875839	0.739228
C	2.797433	-1.651073	0.460772
C	2.581762	-0.385080	-0.433728
C	1.977936	-1.681005	1.748518
C	0.987320	-2.567210	1.858236
C	0.032789	-2.755202	2.997897
C	2.338582	-0.687319	2.819709
C	1.119925	0.001292	-0.342761
C	6.742764	3.733091	-0.192269
C	0.128991	-0.800491	-1.045589
O	0.746808	0.928717	0.375954
C	0.345139	-1.922548	-1.976337
N	-0.871679	-2.551078	-2.129004
C	-1.955833	-1.793642	-1.558991
C	-1.209430	-0.761626	-0.748791
O	1.370718	-2.291489	-2.525776
S	-2.910899	-2.821018	-0.372202
C	-3.864002	-1.441864	0.312181
C	-2.991120	-0.348461	0.932125
N	-1.822055	0.034260	0.137807
C	-2.824196	-1.168115	-2.648045
H	3.427938	1.038548	0.938785
H	5.146343	0.101598	-1.377309
C	-3.907009	0.840451	1.222968
N	-3.756379	1.926141	0.445130
C	-4.672664	3.026426	0.536519
C	-6.000153	2.729594	-0.135325
O	-4.745789	0.756308	2.107503
O	-6.859989	3.734693	0.050867
O	-6.260579	1.735486	-0.760396
H	4.185295	3.985246	-1.210123
H	4.123236	3.423778	0.453877
H	6.003779	2.422760	-1.714691
H	5.850228	1.554731	1.200662
H	7.007359	0.987598	0.000347
H	3.470242	1.748509	-2.022166
H	2.319075	2.379799	-0.848597
H	6.265792	-1.301637	0.614594
H	4.501566	-2.805835	1.249394
H	2.455269	-2.502628	-0.134957
H	2.765783	-0.713730	-1.461489
H	0.821796	-3.238590	1.016113
H	-0.997705	-2.677112	2.636961
H	0.165020	-2.022400	3.793069
H	0.136055	-3.753240	3.433011
H	1.999487	0.314965	2.543143
H	1.895597	-0.938892	3.782208
H	3.422258	-0.637593	2.949039
H	6.612011	4.651872	-0.769792
H	6.564998	3.971891	0.861046
H	7.784274	3.416915	-0.292618
H	-1.027425	-3.137673	-2.934145
H	-4.523013	-1.034425	-0.454587
H	-4.495865	-1.854119	1.096670
H	-2.633704	-0.702740	1.903578

H	-1.151516	0.634574	0.616041
H	-3.303046	-1.957423	-3.228498
H	-3.597010	-0.517861	-2.241454
H	-2.192958	-0.571778	-3.309606
H	-3.091875	1.893268	-0.311485
H	-4.867899	3.267926	1.582121
H	-4.236969	3.909065	0.068719
H	-7.690611	3.519792	-0.396502

ωB97X Energy = -1950.64304348 a.u.

(3*R*,4*S*,5*R*,8*R*,10*S*,19*S*,24*S*)-**3**, Conf B

C	4.094535	2.859219	-1.132625
C	5.562235	2.439739	-1.047453
C	5.749189	1.385042	0.042913
C	4.808392	0.196451	-0.152981
C	3.346295	0.658625	-0.157444
C	3.147933	1.670116	-1.287676
C	5.039394	-0.893770	0.853923
C	4.071924	-1.667199	1.328117
C	2.624839	-1.582789	0.915206
C	2.433743	-0.565848	-0.258132
C	1.716775	-1.357596	2.121328
C	0.745643	-2.238216	2.366752
C	-0.282936	-2.206487	3.455656
C	1.971947	-0.130833	2.954580
C	0.959068	-0.234673	-0.353409
C	6.473474	3.639800	-0.824480
C	0.052006	-1.205067	-0.947944
O	0.503744	0.806391	0.121317
C	0.371384	-2.494771	-1.585986
N	-0.819875	-3.174251	-1.726413
C	-1.960884	-2.337250	-1.458798
C	-1.311479	-1.139435	-0.809278
O	1.451015	-2.947200	-1.931505
S	-3.049051	-3.110472	-0.196821
C	-4.076919	-1.641406	0.054632
C	-3.284022	-0.418020	0.520142
N	-2.019798	-0.191270	-0.183367
C	-2.693930	-1.975425	-2.748539
H	3.155328	1.170596	0.792704
H	5.023333	-0.220592	-1.149123
C	-4.218838	0.789400	0.433819
N	-3.984601	1.669776	-0.553794
C	-4.807818	2.834346	-0.712944
C	-4.509530	3.908387	0.315758
O	-5.155386	0.886329	1.212802
O	-5.389692	4.909327	0.217654
O	-3.607510	3.888276	1.110358
H	3.958856	3.559529	-1.962824
H	3.833143	3.406012	-0.216887
H	5.831988	1.974062	-2.005062
H	5.559634	1.839709	1.024897
H	6.788247	1.037908	0.051126
H	3.335222	1.171935	-2.247987
H	2.115463	2.022758	-1.305833
H	6.062634	-1.050321	1.189505
H	4.304027	-2.435625	2.061965
H	2.353789	-2.562566	0.510614
H	2.699189	-1.112741	-1.168333
H	0.657379	-3.085686	1.687536
H	-1.286197	-2.140521	3.021859
H	-0.160193	-1.367039	4.139008
H	-0.256372	-3.129722	4.040670
H	1.613381	0.762846	2.436168
H	1.477192	-0.178815	3.923558
H	3.042428	0.002267	3.126701
H	6.356541	4.380052	-1.620244
H	6.237966	4.130142	0.125421
H	7.524407	3.340634	-0.794185
H	-0.883880	-3.919997	-2.402189
H	-4.637287	-1.428934	-0.855505
H	-4.798843	-1.890469	0.830009
H	-3.055019	-0.544057	1.582180
H	-1.407931	0.508692	0.234729
H	-3.120931	-2.876640	-3.189755
H	-3.493115	-1.254830	-2.584329
H	-1.982813	-1.537183	-3.451428
H	-3.164240	1.555341	-1.126722
H	-4.652059	3.258553	-1.704738
H	-5.862571	2.568654	-0.628381
H	-5.165826	5.584788	0.873219

ωB97X Energy = -1950.64240150 a.u.

(3*R*,4*S*,5*R*,8*R*,10*S*,19*S*,24*S*)-**3**, Conf C

C	4.286306	3.106989	-0.636257
C	5.749320	2.663591	-0.669029
C	5.943085	1.405849	0.177814
C	4.978181	0.293232	-0.231623
C	3.525858	0.766131	-0.097674
C	3.317996	1.982869	-1.001426
C	5.219491	-0.983111	0.521189
C	4.252358	-1.821303	0.868383
C	2.792975	-1.629690	0.544997
C	2.582399	-0.400332	-0.400263
C	1.940821	-1.627356	1.811360
C	0.964617	-2.528400	1.929209
C	-0.015009	-2.693732	3.050839
C	2.254454	-0.587108	2.852811
C	1.113622	-0.032770	-0.364012
C	6.680989	3.783887	-0.224863
C	0.152753	-0.876879	-1.061884
O	0.706758	0.917157	0.304574
C	0.410017	-2.037786	-1.934653
N	-0.795367	-2.684956	-2.096774
C	-1.903888	-1.915940	-1.594134
C	-1.193464	-0.837588	-0.813770
O	1.455883	-2.418134	-2.434605
S	-2.875391	-2.898295	-0.381499
C	-3.869500	-1.503221	0.204220
C	-3.028329	-0.364826	0.788756

N	-1.843916	-0.001436	0.010868
C	-2.752415	-1.356038	-2.732735
H	3.373545	1.086839	0.939141
H	5.160267	0.088738	-1.298080
C	-3.952565	0.828417	1.000071
N	-3.801795	1.882778	0.196593
C	-4.523997	3.128329	0.391408
C	-5.996047	3.069955	-0.011969
O	-4.827241	0.769041	1.868102
O	-6.730000	2.135905	0.584941
O	-6.485851	3.841181	-0.796549
H	4.145153	3.958130	-1.309763
H	4.051272	3.465768	0.374565
H	5.994041	2.401693	-1.707376
H	5.783388	1.650422	1.236800
H	6.976010	1.052223	0.087447
H	3.476535	1.683215	-2.045613
H	2.291454	2.344249	-0.923446
H	6.251096	-1.221879	0.771993
H	4.492935	-2.728544	1.417876
H	2.479804	-2.507448	-0.028167
H	2.798352	-0.763735	-1.409998
H	0.834289	-3.234294	1.109403
H	0.095472	-1.941693	3.831242
H	0.083299	-3.680683	3.511440
H	-1.037318	-2.629737	2.664695
H	1.899055	0.396301	2.532420
H	1.795882	-0.813738	3.814203
H	3.333555	-0.507141	3.003402
H	6.553043	4.675031	-0.844728
H	6.475188	4.065397	0.812676
H	7.728455	3.477611	-0.286850
H	-0.920088	-3.312267	-2.876161
H	-4.513140	-1.150347	-0.601199
H	-4.515563	-1.885359	0.992506
H	-2.698563	-0.662967	1.788816
H	-1.189349	0.619443	0.486325
H	-3.210390	-2.178292	-3.283516
H	-3.539640	-0.694266	-2.375614
H	-2.112247	-0.786036	-3.408638
H	-3.085382	1.845766	-0.512799
H	-4.472147	3.414813	1.445732
H	-4.044530	3.900313	-0.202401
H	-6.176496	1.610277	1.210561

ωB97X Energy = -1950.64234477 a.u.

(3*R*,4*S*,5*R*,8*R*,10*S*,19*S*,24*S*)-**3**, Conf D

C	4.096931	2.963976	-1.012352
C	5.570962	2.563711	-0.943592
C	5.773262	1.467212	0.101907
C	4.851140	0.273791	-0.146229
C	3.382425	0.713869	-0.127623
C	3.167441	1.768570	-1.214843
C	5.102052	-0.856943	0.809543
C	4.147948	-1.666233	1.249233
C	2.698518	-1.586264	0.843701
C	2.484902	-0.516748	-0.277994
C	1.792317	-1.440483	2.063175
C	0.844036	-2.355173	2.270222
C	-0.174246	-2.406968	3.367974
C	2.027468	-0.251837	2.955288
C	1.006284	-0.196487	-0.345799
C	6.462809	3.767676	-0.669107
C	0.100028	-1.148819	-0.972472
O	0.546453	0.819927	0.175172
C	0.422096	-2.415292	-1.656208
N	-0.766923	-3.094609	-1.811411
C	-1.910242	-2.273495	-1.508157
C	-1.261751	-1.092404	-0.828922
O	1.501625	-2.849264	-2.023329
S	-2.975058	-3.088732	-0.251974
C	-4.015835	-1.638399	0.046865
C	-3.228103	-0.416457	0.531476
N	-1.970482	-0.162915	-0.171039
C	-2.661274	-1.883719	-2.778816
H	3.186726	1.182425	0.843892
H	5.070675	-0.094086	-1.160544
C	-4.170677	0.779871	0.458695
N	-3.934796	1.704727	-0.473798
C	-4.878689	2.765064	-0.785776
C	-4.954443	3.872715	0.263515
O	-5.128526	0.841251	1.233588
O	-5.258766	3.490632	1.499531
O	-4.787904	5.034484	-0.006937
H	3.950460	3.694732	-1.814003
H	3.828045	3.469939	-0.075633
H	5.848690	2.142636	-1.919373
H	5.575707	1.876799	1.101929
H	6.817576	1.136168	0.096899
H	3.359993	1.312292	-2.194730
H	2.130024	2.106895	-1.217266
H	6.128545	-1.012806	1.135259
H	4.393575	-2.463965	1.946529
H	2.444492	-2.549517	0.391773
H	2.747669	-1.016630	-1.215645
H	0.771203	-3.169475	1.549787
H	-0.069626	-1.598680	4.090918
H	-0.113365	-3.356343	3.906716
H	-1.183211	-2.350893	2.946521
H	3.095552	-0.114550	3.139291
H	1.661517	0.661430	2.477745
H	1.528459	-0.351579	3.918084
H	6.337182	4.536773	-1.435645
H	6.216315	4.216258	0.298405
H	7.518143	3.483685	-0.646559
H	-0.832489	-3.821442	-2.507292
H	-4.589528	-1.411040	-0.851250
H	-4.725624	-1.913227	0.824649
H	-2.998763	-0.556612	1.591476
H	-1.353527	0.517658	0.272064
H	-3.083895	-2.776908	-3.239983

H	-3.465868	-1.176324	-2.586922
H	-1.962310	-1.418980	-3.476731
H	-3.122322	1.596012	-1.061524
H	-4.584137	3.218930	-1.726849
H	-5.879135	2.337870	-0.900784
H	-5.375615	2.511200	1.537685

ωB97X Energy = -1950.64203401 a.u.

(3*R*,4*S*,5*R*,8*R*,10*S*,19*S*,24*S*)-**3**, Conf E

C	4.069927	3.104537	-0.310371
C	5.533636	2.689770	-0.464871
C	5.774014	1.336826	0.204531
C	4.802426	0.273273	-0.306272
C	3.353119	0.703204	-0.050498
C	3.096026	2.024158	-0.778438
C	5.087891	-1.086838	0.262324
C	4.144335	-1.977470	0.535709
C	2.670449	-1.766040	0.302373
C	2.406825	-0.426777	-0.462389
C	1.880393	-1.944080	1.596645
C	0.916409	-2.864834	1.638868
C	0.000723	-3.200217	2.776354
C	2.240346	-1.049645	2.752140
C	0.937921	-0.087250	-0.313119
C	6.476438	3.756029	0.077581
C	-0.046255	-0.846541	-1.069970
O	0.556818	0.761812	0.493253
C	0.179360	-1.882631	-2.093225
N	-1.023681	-2.530747	-2.273668
C	-2.115384	-1.851774	-1.624738
C	-1.380333	-0.865976	-0.749508
O	1.201658	-2.177027	-2.691659
S	-3.011278	-2.995239	-0.499327
C	-3.992481	-1.702261	0.305099
C	-3.143140	-0.626465	0.986339
N	-1.994274	-0.159900	0.208033
C	-3.024666	-1.172194	-2.645319
H	3.242715	0.883477	1.024956
H	4.940162	0.211240	-1.396944
C	-4.081571	0.518074	1.374436
N	-3.993409	1.641761	0.652104
C	-4.871959	2.757504	0.886739
C	-4.630562	3.814052	-0.160539
O	-4.888569	0.359054	2.280360
O	-5.441149	4.856695	0.004326
O	-3.806970	3.731592	-1.036257
H	3.891079	4.035779	-0.857044
H	3.877881	3.322091	0.748682
H	5.733184	2.564931	-1.537818
H	5.655416	1.442509	1.291542
H	6.805599	1.012667	0.028845
H	3.213217	1.863809	-1.858156
H	2.070274	2.356823	-0.612567
H	6.131326	-1.340412	0.438668
H	4.416237	-2.944177	0.953355
H	2.339180	-2.565459	-0.366790
H	2.580091	-0.650386	-1.519820
H	0.744296	-3.448572	0.734965
H	0.189622	-2.607074	3.670306
H	0.088111	-4.256858	3.043615
H	-1.040938	-3.038806	2.480170
H	1.867866	-0.035833	2.580279
H	1.825374	-1.404918	3.694196
H	3.324866	-0.980961	2.863940
H	6.319639	4.715123	-0.422786
H	6.310162	3.906839	1.148895
H	7.522191	3.470233	-0.062015
H	-1.181048	-3.051884	-3.122380
H	-4.681154	-1.265887	-0.417964
H	-4.592579	-2.192697	1.069265
H	-2.762545	-1.035106	1.927039
H	-1.328798	0.416797	0.721253
H	-3.490282	-1.929368	-3.277130
H	-3.808538	-0.580737	-2.175739
H	-2.425658	-0.506611	-3.269800
H	-3.316365	1.721123	-0.093746
H	-5.919861	2.450545	0.844603
H	-4.706947	3.200140	1.872727
H	-5.251409	5.512045	-0.682123

ωB97X Energy = -1950.64189255 a.u.

(3*R*,4*S*,5*R*,8*R*,10*S*,19*S*,24*S*)-**3**, Conf F

C	4.367431	3.070475	-0.617134
C	5.818637	2.593327	-0.685572
C	5.996771	1.312703	0.130020
C	4.997832	0.232730	-0.284741
C	3.560264	0.737861	-0.113761
C	3.365804	1.977901	-0.988159
C	5.221306	-1.064547	0.437300
C	4.240382	-1.885696	0.786549
C	2.780459	-1.651513	0.495467
C	2.582416	-0.398419	-0.420890
C	1.953010	-1.653709	1.778345
C	0.956351	-2.531912	1.897266
C	-0.005928	-2.695310	3.034150
C	2.314252	-0.644147	2.834362
C	1.123798	0.003091	-0.346026
C	6.784563	3.680560	-0.232437
C	0.129912	-0.801904	-1.041483
O	0.754523	0.946221	0.353804
C	0.342006	-1.941039	-1.952381
N	-0.879272	-2.561446	-2.102333
C	-1.960143	-1.785711	-1.551211
C	-1.209677	-0.746662	-0.753758
O	1.367561	-2.327581	-2.489474
S	-2.930837	-2.785707	-0.353611
C	-3.876110	-1.387581	0.302720
C	-2.997212	-0.291737	0.909744
N	-1.821467	0.069523	0.115188
C	-2.817164	-1.171212	-2.655436

H	3.435232	1.040718	0.931985
H	5.155753	0.046184	-1.358313
C	-3.903646	0.909841	1.174849
N	-3.752805	1.973330	0.368456
C	-4.648309	3.094173	0.458325
C	-6.056826	2.834429	-0.043074
O	-4.746112	0.853699	2.058638
O	-6.175219	1.724568	-0.773859
O	-6.973549	3.583020	0.181247
H	4.235090	3.939438	-1.269339
H	4.158904	3.412438	0.405269
H	6.039533	2.349469	-1.733605
H	5.861793	1.537922	1.196725
H	7.019011	0.936444	0.013340
H	3.498548	1.696797	-2.041057
H	2.349836	2.361897	-0.883830
H	6.251170	-1.333365	0.663756
H	4.468626	-2.809361	1.313481
H	2.434004	-2.509333	-0.088677
H	2.769419	-0.746158	-1.441829
H	0.792108	-3.217023	1.066024
H	0.129197	-1.953649	3.820563
H	0.085275	-3.688586	3.482581
H	-1.033675	-2.611861	2.666620
H	1.985754	0.355866	2.537605
H	1.862454	-0.874724	3.798022
H	3.397436	-0.601251	2.970397
H	6.667239	4.589101	-0.828717
H	6.603549	3.941936	0.814938
H	7.823033	3.350956	-0.320455
H	-1.035211	-3.159954	-2.898607
H	-4.527385	-0.987396	-0.474498
H	-4.515721	-1.781804	1.090279
H	-2.648170	-0.633085	1.888812
H	-1.149198	0.672685	0.587343
H	-3.297216	-1.966250	-3.227009
H	-3.588552	-0.510004	-2.263971
H	-2.177929	-0.588903	-3.321749
H	-3.079320	1.928564	-0.379346
H	-4.739805	3.430332	1.491317
H	-4.243940	3.922352	-0.123690
H	-7.097339	1.645708	-1.056837

ωB97X Energy = -1950.64140167 a.u.

(3*R*,4*S*,5*R*,8*R*,10*S*,19*S*,24*S*)-**3**, Conf G

C	4.126147	2.806264	-1.182620
C	5.589273	2.375754	-1.076981
C	5.758877	1.341674	0.035718
C	4.808083	0.158088	-0.142034
C	3.350631	0.634113	-0.167282
C	3.170373	1.622346	-1.320789
C	5.021425	-0.912802	0.889236
C	4.042965	-1.664937	1.375058
C	2.598886	-1.572521	0.953470
C	2.427023	-0.583666	-0.246361
C	1.688283	-1.302571	2.148521
C	0.703463	-2.162489	2.412118
C	-0.331492	-2.084955	3.492705
C	1.955793	-0.057412	2.950330
C	0.956370	-0.241500	-0.361034
C	6.509570	3.571973	-0.871142
C	0.044571	-1.218310	-0.938060
O	0.507089	0.815006	0.084275
C	0.356906	-2.525693	-1.543190
N	-0.839490	-3.197078	-1.677649
C	-1.974556	-2.343519	-1.438760
C	-1.318930	-1.137589	-0.810743
O	1.435166	-2.995824	-1.868564
S	-3.082783	-3.078567	-0.171236
C	-4.094763	-1.592409	0.042493
C	-3.289487	-0.372864	0.494876
N	-2.023537	-0.168481	-0.212436
C	-2.692601	-2.002240	-2.742681
H	3.158069	1.169142	0.769645
H	5.026097	-0.282060	-1.127516
C	-4.207945	0.845000	0.393220
N	-3.975482	1.700952	-0.615212
C	-4.805223	2.861130	-0.797777
C	-4.633878	3.948996	0.245818
O	-5.129559	0.979921	1.184830
O	-3.500984	3.856223	0.942117
O	-5.440099	4.830695	0.401717
H	4.003401	3.490291	-2.028243
H	3.862160	3.374148	-0.280521
H	5.862238	1.888595	-2.022915
H	5.567205	1.817783	1.007084
H	6.794492	0.985059	0.058093
H	3.361439	1.102508	-2.268758
H	2.141327	1.983555	-1.355437
H	6.041137	-1.073545	1.233457
H	4.262750	-2.420051	2.126291
H	2.316122	-2.558862	0.573297
H	2.694621	-1.155361	-1.140545
H	0.607395	-3.027447	1.756457
H	-0.213337	-1.216274	4.139182
H	-0.308315	-2.981639	4.117934
H	-1.331897	-2.038843	3.050063
H	1.608412	0.827004	2.408791
H	1.459103	-0.074994	3.919347
H	3.027365	0.067952	3.121396
H	6.405182	4.297374	-1.682166
H	6.271409	4.083106	0.067097
H	7.557509	3.264202	-0.826857
H	-0.905025	-3.958732	-2.335274
H	-4.641984	-1.388835	-0.877637
H	-4.827934	-1.818346	0.814526
H	-3.062692	-0.489304	1.558576
H	-1.407977	0.536051	0.192155
H	-3.123565	-2.908677	-3.169093
H	-3.487162	-1.271759	-2.600635
H	-1.971460	-1.584747	-3.447975

H	-3.168927	1.561674	-1.202153
H	-4.584375	3.305964	-1.768181
H	-5.859840	2.584761	-0.791973
H	-3.470313	4.592284	1.569678

ωB97X Energy = -1950.64094417 a.u.

(3*R*,4*S*,5*R*,8*R*,10*S*,19*S*,24*S*)-**3**, Conf H

C	-4.281038	3.188795	0.197339
C	-5.731495	2.780438	0.458679
C	-6.006270	1.392301	-0.120344
C	-4.999801	0.361517	0.390288
C	-3.573013	0.778480	0.016107
C	-3.275155	2.134957	0.658217
C	-5.314130	-1.033429	-0.068356
C	-4.386417	-1.936854	-0.354682
C	-2.900115	-1.701998	-0.256866
C	-2.597524	-0.328137	0.422870
C	-2.228107	-1.934236	-1.607600
C	-1.294480	-2.881895	-1.706333
C	-0.515378	-3.290072	-2.920055
C	-2.675765	-1.073928	-2.758726
C	-1.143841	0.012703	0.174644
C	-6.707720	3.815702	-0.084808
C	-0.113476	-0.639608	0.973098
O	-0.812180	0.790064	-0.719981
C	-0.271922	-1.587273	2.094562
N	0.979266	-2.082020	2.367670
C	2.025806	-1.357067	1.686169
C	1.220355	-0.572346	0.673247
O	-1.283784	-1.923683	2.689503
S	3.268162	-2.458598	0.873070
C	3.140400	-1.909818	-0.854357
C	3.002583	-0.400996	-0.998117
N	1.791431	0.086451	-0.347923
C	2.746295	-0.424205	2.665940
H	-3.534744	0.905357	-1.071379
H	-5.066565	0.368419	1.489393
C	4.280238	0.338823	-0.602183
N	4.139187	1.503430	0.035899
C	5.269477	2.263620	0.541171
C	6.068656	2.986361	-0.541621
O	5.379540	-0.107182	-0.936225
O	6.566878	2.221526	-1.508636
O	6.273069	4.172722	-0.517191
H	-4.075027	4.145702	0.687159
H	-4.152327	3.355543	-0.880259
H	-5.868718	2.713646	1.546459
H	-5.954478	1.436961	-1.216733
H	-7.023945	1.077677	0.135585
H	-3.322813	2.029681	1.749992
H	-2.263296	2.460967	0.411248
H	-6.365549	-1.302853	-0.146431
H	-4.681147	-2.930238	-0.685035
H	-2.500377	-2.465409	0.417503
H	-2.708157	-0.502597	1.497617
H	-1.049498	-3.437730	-0.801838
H	-0.641727	-4.358143	-3.116095
H	0.555159	-3.127296	-2.762392
H	-0.802246	-2.744209	-3.817865
H	-2.247982	-0.071230	-2.672278
H	-2.378674	-1.484663	-3.722471
H	-3.762593	-0.964385	-2.758657
H	-6.531845	4.797937	0.361357
H	-6.596881	3.916299	-1.169080
H	-7.743223	3.532724	0.121174
H	1.156575	-2.530485	3.252922
H	4.057881	-2.241383	-1.336031
H	2.291544	-2.389405	-1.341119
H	2.885816	-0.187924	-2.064032
H	1.107095	0.566410	-0.927230
H	3.241844	-1.023234	3.431388
H	3.502795	0.175449	2.163703
H	2.021457	0.235065	3.147526
H	3.206545	1.830084	0.237459
H	4.899771	3.008437	1.239475
H	5.948186	1.591049	1.073780
H	6.320203	1.277758	-1.362904

ωB97X Energy = -1950.64094228 a.u.

(3*R*,4*S*,5*R*,8*R*,10*S*,19*S*,24*S*)-**3**, Conf I

C	4.460653	3.025852	-0.941862
C	5.919305	2.570709	-0.889570
C	6.085481	1.445630	0.131521
C	5.119548	0.292216	-0.138228
C	3.667542	0.783330	-0.103795
C	3.487174	1.869701	-1.165867
C	5.333352	-0.867770	0.791199
C	4.352322	-1.653368	1.214612
C	2.906267	-1.514313	0.813243
C	2.725666	-0.410081	-0.280431
C	2.004569	-1.370690	2.036282
C	1.012382	-2.245227	2.207316
C	-0.017175	-2.283181	3.295141
C	2.288495	-0.222907	2.966766
C	1.257558	-0.035450	-0.333308
C	6.857024	3.734654	-0.596245
C	0.320355	-0.934143	-0.977983
O	0.839302	0.982951	0.223077
C	0.587275	-2.198662	-1.676938
N	-0.628022	-2.839630	-1.827505
C	-1.738200	-1.977516	-1.512010
C	-1.045276	-0.834153	-0.812893
O	1.645359	-2.670425	-2.064173
S	-2.838049	-2.764067	-0.268566
C	-3.839602	-1.281528	0.012624
C	-3.033007	-0.059245	0.473840
N	-1.706777	0.079858	-0.116843
C	-2.470685	-1.526641	-2.773936
H	3.490971	1.236195	0.878948
H	5.322259	-0.060771	-1.161325

C	-3.869820	1.182111	0.142487
N	-4.540722	1.714186	1.181049
C	-5.497358	2.765848	0.979003
C	-6.798255	2.258396	0.385844
O	-3.939394	1.618632	-0.994227
O	-7.629217	3.273133	0.132951
O	-7.066209	1.105990	0.170472
H	4.337527	3.778879	-1.726699
H	4.214737	3.520611	0.007116
H	6.176224	2.159874	-1.875383
H	5.906389	1.840583	1.140918
H	7.116951	1.076751	0.114885
H	3.659321	1.428812	-2.156581
H	2.462640	2.245083	-1.155915
H	6.354548	-1.065975	1.110676
H	4.571814	-2.474809	1.893022
H	2.619714	-2.456250	0.336250
H	2.967940	-0.895247	-1.231183
H	0.904207	-3.028612	1.457544
H	0.115821	-1.499973	4.040762
H	-0.003304	-3.248426	3.808494
H	-1.018333	-2.171215	2.865976
H	1.970656	0.720126	2.513085
H	1.777251	-0.327262	3.922707
H	3.360334	-0.142064	3.162323
H	6.757778	4.522196	-1.347753
H	6.631641	4.173974	0.380656
H	7.900846	3.410245	-0.583976
H	-0.722994	-3.536900	-2.549781
H	-4.401638	-1.054671	-0.891620
H	-4.558912	-1.533925	0.790252
H	-2.885212	-0.129594	1.554955
H	-1.090472	0.770557	0.306031
H	-2.958540	-2.383586	-3.239939
H	-3.216232	-0.760630	-2.566607
H	-1.743758	-1.107619	-3.472461
H	-4.479301	1.281879	2.087855
H	-5.715047	3.251644	1.929784
H	-5.090516	3.522849	0.307273
H	-8.450143	2.917798	-0.235817

ωB97X Energy = -1950.64080483 a.u.

(3*R*,4*S*,5*R*,8*R*,10*S*,19*S*,24*S*)-**3**, Conf J

C	3.837252	3.149049	-0.793780
C	5.293371	2.831050	-1.137428
C	5.739898	1.547393	-0.436742
C	4.787491	0.387822	-0.727810
C	3.365883	0.734472	-0.269444
C	2.894360	1.971123	-1.036518
C	5.263637	-0.913721	-0.150515
C	4.455106	-1.820447	0.381591
C	2.959353	-1.675317	0.482767
C	2.478037	-0.501275	-0.432068
C	2.493542	-1.601520	1.938779
C	3.196788	-0.937500	2.855065
C	2.887781	-0.755627	4.310311
C	1.233977	-2.371589	2.236538
C	1.009114	-0.232085	-0.193476
C	6.210079	3.999534	-0.798212
C	0.012863	-1.018515	-0.905486
O	0.640338	0.615702	0.620966
C	0.216902	-2.109608	-1.876213
N	-1.015116	-2.693876	-2.080468
C	-2.089671	-1.918804	-1.514929
C	-1.332459	-0.956775	-0.631875
O	1.243293	-2.493762	-2.412986
S	-3.132074	-2.956482	-0.415240
C	-4.055415	-1.568698	0.291860
C	-3.166068	-0.540887	0.996615
N	-1.936817	-0.191633	0.282200
C	-2.886304	-1.205865	-2.605884
H	3.403559	0.993255	0.795827
H	4.756306	0.267938	-1.822146
C	-4.036911	0.682190	1.284033
N	-3.797166	1.784120	0.552772
C	-4.656824	2.928973	0.649059
C	-5.977286	2.723779	-0.069241
O	-4.920817	0.610994	2.124497
O	-6.793311	3.760915	0.137817
O	-6.266375	1.771331	-0.744449
H	3.506594	4.020811	-1.367199
H	3.782285	3.432926	0.265553
H	5.349655	2.650591	-2.219425
H	5.780925	1.718296	0.647691
H	6.755293	1.285348	-0.753706
H	2.859884	1.737770	-2.108753
H	1.882135	2.245672	-0.735552
H	6.332967	-1.110560	-0.195292
H	4.867922	-2.737934	0.792849
H	2.521401	-2.588159	0.062690
H	2.575685	-0.869027	-1.457459
H	4.117604	-0.460744	2.526263
H	1.975630	-1.261028	4.624699
H	2.780589	0.306926	4.546094
H	3.709497	-1.131518	4.926141
H	0.421391	-2.087526	1.562492
H	1.406898	-3.440702	2.077872
H	0.881473	-2.235259	3.257327
H	5.906411	4.907600	-1.325445
H	6.180516	4.210376	0.275518
H	7.247051	3.783293	-1.067871
H	-1.166802	-3.243838	-2.911890
H	-4.663608	-1.101054	-0.482299
H	-4.737049	-1.986789	1.030196
H	-2.878742	-0.945311	1.970836
H	-1.260330	0.355992	0.812936
H	-3.370697	-1.946878	-3.242659
H	-3.650129	-0.543818	-2.201406
H	-2.204781	-0.606353	-3.212369
H	-3.094581	1.748718	-0.168260
H	-4.870276	3.153941	1.694851

H	-4.160903	3.798541	0.218274
H	-7.618917	3.607595	-0.342794

ωB97X Energy = -1950.64068814 a.u.

(3*R*,4*S*,5*R*,8*R*,10*S*,19*S*,24*S*)-**3**, Conf K

C	3.625054	2.770505	-1.479238
C	5.106604	2.429220	-1.644860
C	5.536183	1.400211	-0.598589
C	4.633482	0.166695	-0.620846
C	3.176063	0.563966	-0.357740
C	2.728743	1.533036	-1.453549
C	5.098932	-0.911677	0.314520
C	4.276624	-1.677085	1.019649
C	2.774332	-1.572717	0.985734
C	2.330851	-0.706657	-0.238318
C	2.205357	-1.129825	2.335450
C	2.826781	-0.217297	3.081166
C	2.409052	0.334072	4.410883
C	0.944303	-1.841843	2.748315
C	0.842606	-0.442700	-0.176003
C	5.975008	3.679430	-1.589153
C	-0.081524	-1.422800	-0.726049
O	0.396730	0.575049	0.356552
C	0.213513	-2.733349	-1.333017
N	-0.994708	-3.380800	-1.486772
C	-2.115812	-2.502848	-1.268904
C	-1.447555	-1.318852	-0.613183
O	1.286683	-3.226655	-1.639857
S	-3.274724	-3.219055	-0.037112
C	-4.260865	-1.712355	0.150286
C	-3.446744	-0.506018	0.625997
N	-2.137644	-0.346932	-0.009834
C	-2.790761	-2.133508	-2.588365
H	3.133145	1.095635	0.600708
H	4.680440	-0.243898	-1.641731
C	-4.327663	0.733230	0.460261
N	-4.006468	1.580464	-0.532315
C	-4.768437	2.776100	-0.755029
C	-4.456438	3.865344	0.253398
O	-5.300174	0.883541	1.184538
O	-5.268381	4.912620	0.080178
O	-3.597892	3.818474	1.093713
H	3.311464	3.446629	-2.280829
H	3.495831	3.320576	-0.537763
H	5.233308	1.964564	-2.632126
H	5.499185	1.857466	0.399533
H	6.576572	1.104834	-0.773115
H	2.773733	1.022920	-2.424658
H	1.692553	1.836430	-1.297579
H	6.173068	-1.067970	0.392116
H	4.680504	-2.433135	1.687961
H	2.387528	-2.581653	0.801799
H	2.510235	-1.328513	-1.119784
H	3.757881	0.192008	2.695292
H	1.487308	-0.104529	4.790873
H	2.263437	1.415986	4.344852
H	3.191516	0.168882	5.156791
H	1.152670	-2.904874	2.905310
H	0.510260	-1.445182	3.664357
H	0.180564	-1.789247	1.967881
H	5.681488	4.399663	-2.357159
H	5.878322	4.170717	-0.615805
H	7.030461	3.437956	-1.738969
H	-1.063928	-4.138301	-2.148829
H	-4.778547	-1.499166	-0.784410
H	-5.020023	-1.923134	0.901005
H	-3.277808	-0.610400	1.700840
H	-1.515706	0.333110	0.426542
H	-3.228098	-3.027385	-3.034304
H	-3.574429	-1.388660	-2.461677
H	-2.042376	-1.725015	-3.270049
H	-3.159222	1.425079	-1.054443
H	-4.561055	3.162600	-1.752714
H	-5.836353	2.560192	-0.699395
H	-5.038465	5.593674	0.727833

ωB97X Energy = -1950.64025013 a.u.

(3*R*,4*S*,5*R*,8*R*,10*S*,19*S*,24*S*)-**3**, Conf L

C	-4.433239	3.082917	-0.402795
C	-5.821594	2.726797	0.130401
C	-6.092226	1.232683	-0.047112
C	-4.984808	0.382750	0.574752
C	-3.628614	0.720353	-0.056149
C	-3.329721	2.201735	0.180683
C	-5.277666	-1.089071	0.511191
C	-4.341496	-2.015793	0.356334
C	-2.865752	-1.732281	0.233515
C	-2.565982	-0.224533	0.510247
C	-2.314194	-2.286254	-1.077299
C	-1.344770	-3.201406	-1.031220
C	-0.659996	-3.883105	-2.177130
C	-2.913210	-1.762030	-2.354660
C	-1.156648	0.073255	0.044490
C	-6.904798	3.572202	-0.527064
C	-0.035197	-0.322317	0.884807
O	-0.939274	0.595664	-1.049164
C	-0.055040	-0.941454	2.224481
N	1.236515	-1.315126	2.502492
C	2.187775	-0.766948	1.563533
C	1.266585	-0.306272	0.455210
O	-0.996892	-1.133045	2.978694
S	3.422285	-2.005601	0.966585
C	3.131636	-1.948444	-0.825780
C	2.899326	-0.542854	-1.357820
N	1.719110	0.066188	-0.749879
C	2.927755	0.419275	2.189937
H	-3.712834	0.561252	-1.136841
H	-4.928676	0.660965	1.638752
C	4.166450	0.319528	-1.327126
N	4.000713	1.623840	-1.051069

C	5.102004	2.542137	-1.175656
C	6.213477	2.363807	-0.159775
O	5.250632	-0.160047	-1.623209
O	5.824570	1.761518	0.964821
O	7.332992	2.769046	-0.342451
H	-4.220436	4.137190	-0.199834
H	-4.438527	2.969841	-1.495017
H	-5.828246	2.935148	1.208917
H	-6.170425	1.000842	-1.118028
H	-7.056517	0.973988	0.403731
H	-3.245495	2.379985	1.260736
H	-2.370873	2.472717	-0.264315
H	-6.317427	-1.391684	0.618608
H	-4.620469	-3.066162	0.314393
H	-2.366709	-2.288851	1.032928
H	-2.570262	-0.122111	1.599741
H	-0.986487	-3.500471	-0.046701
H	0.408509	-3.645990	-2.182191
H	-1.064704	-3.600357	-3.148028
H	-0.737382	-4.969199	-2.079391
H	-2.535718	-0.758093	-2.567158
H	-2.683478	-2.395485	-3.210143
H	-3.999756	-1.688694	-2.269472
H	-6.722021	4.638897	-0.373694
H	-6.933825	3.389215	-1.605873
H	-7.892200	3.337872	-0.120998
H	1.505815	-1.499432	3.456322
H	4.028919	-2.363468	-1.280319
H	2.279248	-2.575138	-1.088291
H	2.674118	-0.639476	-2.423510
H	0.968392	0.360368	-1.369336
H	3.505615	0.066087	3.045998
H	3.619236	0.879684	1.487029
H	2.204801	1.160931	2.536132
H	3.085923	1.965041	-0.805146
H	5.564870	2.462469	-2.159950
H	4.723884	3.558861	-1.064704
H	6.585646	1.705421	1.559987

ωB97X Energy = -1950.64021177 a.u.

(3*R*,4*S*,5*R*,8*R*,10*S*,19*S*,24*S*)-**3**, Conf M

C	4.365379	2.788269	-1.557210
C	5.823353	2.473043	-1.220119
C	5.907046	1.692361	0.091690
C	5.026937	0.443541	0.061155
C	3.564058	0.822594	-0.198702
C	3.473377	1.548080	-1.542788
C	5.172437	-0.395469	1.298123
C	4.176412	-1.089745	1.831592
C	2.777792	-1.151011	1.273747
C	2.691987	-0.431467	-0.113045
C	1.753319	-0.691078	2.307917
C	0.770707	-1.524029	2.653392
C	-0.357904	-1.281975	3.608891
C	1.912848	0.697563	2.866758
C	1.226978	-0.191788	-0.417972
C	6.671188	3.737573	-1.175292
C	0.404872	-1.306500	-0.845136
O	0.707450	0.910969	-0.229903
C	0.805297	-2.690698	-1.132339
N	-0.352440	-3.443239	-1.177759
C	-1.535448	-2.621819	-1.185749
C	-0.973624	-1.274875	-0.805323
O	1.919453	-3.165110	-1.294092
S	-2.668584	-3.112215	0.173856
C	-3.790270	-1.707075	-0.046183
C	-3.112149	-0.333005	0.064468
N	-1.745404	-0.260466	-0.441934
C	-2.199472	-2.607776	-2.560642
H	3.256163	1.524287	0.584819
H	5.365101	-0.164339	-0.792469
C	-3.985722	0.669526	-0.702076
N	-4.753616	1.460826	0.059619
C	-5.676503	2.400777	-0.523622
C	-6.409222	3.125897	0.575677
O	-3.997670	0.697035	-1.923483
O	-7.282080	4.001708	0.084755
O	-6.228562	2.939456	1.752474
H	4.310958	3.279466	-2.533882
H	3.984498	3.510241	-0.822682
H	6.215522	1.822608	-2.013781
H	5.590105	2.339052	0.921325
H	6.946675	1.409940	0.290713
H	3.784290	0.859757	-2.339590
H	2.441157	1.834204	-1.750945
H	6.158191	-0.434823	1.757442
H	4.345924	-1.673099	2.733814
H	2.559653	-2.205502	1.079271
H	3.061405	-1.156119	-0.845255
H	0.754508	-2.502966	2.175147
H	-0.365264	-2.037023	4.399654
H	-1.316276	-1.367732	3.086168
H	-0.320101	-0.299291	4.077425
H	1.580176	1.441594	2.137486
H	1.341593	0.843645	3.782448
H	2.962360	0.906080	3.086453
H	6.630452	4.274091	-2.126728
H	6.311776	4.414620	-0.393883
H	7.718316	3.507001	-0.962816
H	-0.345264	-4.322918	-1.670702
H	-4.299387	-1.809102	-1.002646
H	-4.542861	-1.783723	0.737235
H	-3.063778	-0.053713	1.120542
H	-1.212479	0.572071	-0.200165
H	-2.602578	-3.596450	-2.782983
H	-3.000001	-1.872094	-2.623483
H	-1.449063	-2.351716	-3.311036
H	-4.703784	1.431279	1.067757
H	-5.157752	3.135609	-1.144601
H	-6.405850	1.896973	-1.162827
H	-7.727601	4.445719	0.820329

ωB97X Energy = -1950.64013098 a.u.

(3*R*,4*S*,5*R*,8*R*,10*S*,19*S*,24*S*)-**3**, Conf N

C	4.068931	3.058469	-0.944556
C	5.551630	2.686818	-0.900180
C	5.785582	1.555238	0.100636
C	4.885113	0.353738	-0.185873
C	3.409056	0.765035	-0.133807
C	3.162312	1.851983	-1.181752
C	5.170082	-0.809651	0.719571
C	4.236439	-1.651024	1.142703
C	2.778750	-1.577284	0.767030
C	2.527576	-0.472832	-0.312417
C	1.892893	-1.487393	2.006767
C	0.946611	-2.409740	2.186913
C	-0.061794	-2.504142	3.291103
C	2.138099	-0.336530	2.944702
C	1.042340	-0.170742	-0.335331
C	6.419379	3.898681	-0.585767
C	0.136257	-1.109138	-0.968724
O	0.585154	0.819734	0.240187
C	0.448022	-2.350611	-1.691115
N	-0.736397	-3.051702	-1.813643
C	-1.880138	-2.258814	-1.443281
C	-1.226307	-1.081903	-0.764801
O	1.517185	-2.760968	-2.115264
S	-2.860135	-3.111357	-0.142582
C	-3.957207	-1.705482	0.172890
C	-3.228059	-0.405965	0.559592
N	-1.912020	-0.210666	-0.036295
C	-2.702961	-1.853206	-2.663522
H	3.218119	1.197886	0.855068
H	5.100260	0.031855	-1.216676
C	-4.132291	0.759619	0.157473
N	-4.734752	1.421139	1.149402
C	-5.774370	2.409632	0.912235
C	-5.259135	3.743556	0.373506
O	-4.305505	1.042534	-1.028825
O	-4.572122	3.690822	-0.763461
O	-5.485484	4.793957	0.916914
H	3.901775	3.813357	-1.719398
H	3.796884	3.526107	0.010958
H	5.831116	2.309515	-1.893217
H	5.588826	1.921292	1.117560
H	6.836501	1.246765	0.073775
H	3.355027	1.434166	-2.178587
H	2.117938	2.167795	-1.162889
H	6.204750	-0.962332	1.020110
H	4.507429	-2.472058	1.802705
H	2.528962	-2.527649	0.286226
H	2.778071	-0.935329	-1.272248
H	0.862933	-3.191977	1.432787
H	0.050449	-1.724382	4.043745
H	0.001849	-3.473665	3.792414
H	-1.073647	-2.429673	2.879082
H	1.760685	0.593851	2.511066
H	1.654958	-0.478661	3.910337
H	3.208398	-0.201954	3.117459
H	6.272982	4.692132	-1.323240
H	6.168499	4.306307	0.398590
H	7.480671	3.636914	-0.578767
H	-0.819219	-3.748241	-2.538104
H	-4.586005	-1.548365	-0.701040
H	-4.605762	-1.994654	0.998315
H	-3.081599	-0.400271	1.642223
H	-1.319868	0.504738	0.381042
H	-3.167919	-2.737009	-3.101924
H	-3.474896	-1.125744	-2.416449
H	-2.037483	-1.398761	-3.400111
H	-4.514334	1.189635	2.104663
H	-6.496294	2.008276	0.195568
H	-6.285336	2.602837	1.850370
H	-4.495037	2.759121	-1.076733

ωB97X Energy = -1950.64005754 a.u.

(3*R*,4*S*,5*R*,8*R*,10*S*,19*S*,24*S*)-**3**, Conf O

C	3.820951	3.105460	-0.920583
C	5.288968	2.780664	-1.202119
C	5.723370	1.545422	-0.412474
C	4.789211	0.362122	-0.663671
C	3.352500	0.720941	-0.266234
C	2.893819	1.906552	-1.117208
C	5.258452	-0.900533	-0.000553
C	4.441984	-1.783691	0.558349
C	2.942922	-1.646854	0.609046
C	2.479383	-0.530281	-0.383574
C	2.434155	-1.495112	2.044248
C	3.104814	-0.772589	2.940402
C	2.751208	-0.509347	4.372894
C	1.172469	-2.258751	2.348904
C	1.002402	-0.262452	-0.203600
C	6.186973	3.975363	-0.907209
C	0.034382	-1.095187	-0.904636
O	0.600533	0.623106	0.552267
C	0.277584	-2.234887	-1.810160
N	-0.944267	-2.834308	-2.027373
C	-2.040541	-2.040576	-1.535647
C	-1.317944	-1.027131	-0.682919
O	1.324051	-2.639934	-2.288564
S	-3.102329	-3.029757	-0.409599
C	-4.062416	-1.617721	0.191015
C	-3.202704	-0.541279	0.861355
N	-1.958417	-0.213069	0.166116
C	-2.812736	-1.392686	-2.682998
H	3.357141	1.040851	0.783118
H	4.790865	0.180316	-1.749883
C	-4.081049	0.687569	1.063485
N	-3.831081	1.763396	0.315149
C	-4.510411	3.032418	0.513582
C	-5.954108	3.055157	0.014658

O	-5.010247	0.638088	1.873396
O	-6.766840	2.138529	0.531301
O	-6.356331	3.872080	-0.773407
H	3.500571	3.936441	-1.556963
H	3.732750	3.454941	0.116624
H	5.378720	2.536300	-2.269194
H	5.728575	1.780400	0.660681
H	6.750245	1.274658	-0.681475
H	2.891049	1.609479	-2.174157
H	1.871607	2.189963	-0.861453
H	6.330091	-1.089757	-0.003382
H	4.849593	-2.673438	1.031075
H	2.523968	-2.585652	0.228491
H	2.609263	-0.954082	-1.383432
H	4.031195	-0.306803	2.611710
H	2.636291	0.564567	4.544882
H	3.553567	-0.847829	5.034288
H	1.830008	-0.998011	4.687373
H	0.796823	-2.078644	3.354499
H	0.373358	-2.010475	1.645161
H	1.354253	-3.332797	2.242566
H	5.891751	4.847836	-1.495848
H	6.125183	4.249071	0.150822
H	7.232716	3.752823	-1.134509
H	-1.066052	-3.432377	-2.829887
H	-4.652413	-1.204141	-0.626339
H	-4.760251	-2.002480	0.932382
H	-2.944200	-0.887664	1.866090
H	-1.298332	0.358238	0.693636
H	-3.281443	-2.169214	-3.288302
H	-3.585344	-0.710024	-2.332909
H	-2.118125	-0.826141	-3.306009
H	-3.076082	1.720417	-0.352351
H	-4.515295	3.276471	1.579785
H	-3.960856	3.803655	-0.017207
H	-6.278718	1.566566	1.170388

ωB97X Energy = -1950.63990725 a.u.

(3*R*,4*S*,5*R*,8*R*,10*S*,19*S*,24*S*)-**3**, Conf P

C	3.660220	2.859644	-1.391225
C	5.144053	2.531809	-1.562713
C	5.578774	1.483067	-0.538487
C	4.685486	0.243723	-0.591848
C	3.224231	0.622944	-0.323584
C	2.772169	1.616074	-1.395850
C	5.156078	-0.853686	0.318377
C	4.337211	-1.644715	0.998724
C	2.834155	-1.553995	0.959359
C	2.388585	-0.657064	-0.241798
C	2.253354	-1.157460	2.318337
C	2.862255	-0.263235	3.095956
C	2.432670	0.241149	4.440479
C	0.997192	-1.894018	2.702558
C	0.898684	-0.405672	-0.178085
C	6.004460	3.785971	-1.479759
C	-0.017713	-1.378892	-0.756396
O	0.442844	0.594477	0.377882
C	0.289444	-2.673264	-1.394169
N	-0.913438	-3.324393	-1.567076
C	-2.041718	-2.462016	-1.327723
C	-1.382895	-1.286963	-0.647816
O	1.367317	-3.150080	-1.708553
S	-3.185681	-3.215272	-0.103461
C	-4.188793	-1.725914	0.118710
C	-3.383319	-0.519216	0.613431
N	-2.081904	-0.328795	-0.026203
C	-2.726035	-2.072750	-2.636228
H	3.173600	1.129099	0.648205
H	4.738179	-0.142007	-1.622134
C	-4.278270	0.705941	0.464997
N	-3.960902	1.604184	-0.469308
C	-4.850387	2.690284	-0.846926
C	-4.937082	3.819449	0.178350
O	-5.274034	0.814419	1.184817
O	-5.318619	3.471999	1.403214
O	-4.711995	4.968894	-0.102021
H	3.342986	3.551949	-2.177387
H	3.526409	3.387129	-0.437536
H	5.274558	2.089054	-2.559514
H	5.535558	1.917629	0.469487
H	6.621802	1.199456	-0.716591
H	2.821631	1.129770	-2.378881
H	1.733755	1.908622	-1.234005
H	6.231146	-1.002333	0.397789
H	4.744573	-2.414337	1.649155
H	2.458841	-2.560978	0.743710
H	2.575401	-1.252486	-1.139864
H	3.791788	0.166228	2.728700
H	3.209659	0.051811	5.186368
H	1.509199	-0.212576	4.797617
H	2.285021	1.324373	4.410939
H	1.216197	-2.958443	2.833477
H	0.552230	-1.526298	3.625384
H	0.238475	-1.829661	1.918089
H	5.708057	4.519501	-2.233928
H	5.902855	4.257387	-0.497102
H	7.061677	3.554021	-1.632113
H	-0.976649	-4.069709	-2.243369
H	-4.714414	-1.500273	-0.808591
H	-4.940847	-1.960179	0.869526
H	-3.211642	-0.639300	1.686219
H	-1.460435	0.337917	0.431980
H	-3.160252	-2.960754	-3.096587
H	-3.513144	-1.334783	-2.492223
H	-1.983422	-1.647113	-3.313646
H	-3.121772	1.460025	-1.010327
H	-4.494140	3.115410	-1.779986
H	-5.858456	2.295705	-1.003772
H	-5.474778	2.498683	1.451661

ωB97X Energy = -1950.63987153 a.u.

HPLC Chromatograms; HRESIMS; NMR Spectra

Aplospojaveedins A (1)

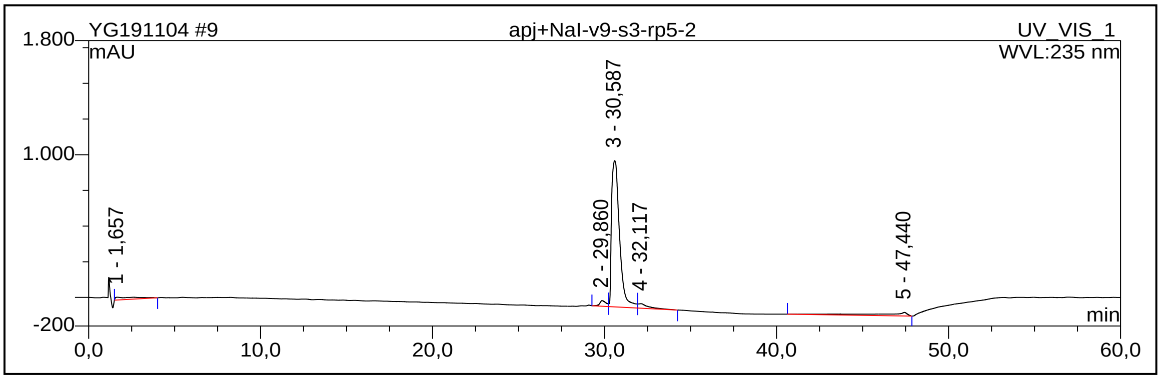

Figure S14. HPLC chromatogram of Aplospojaveedins A (1)

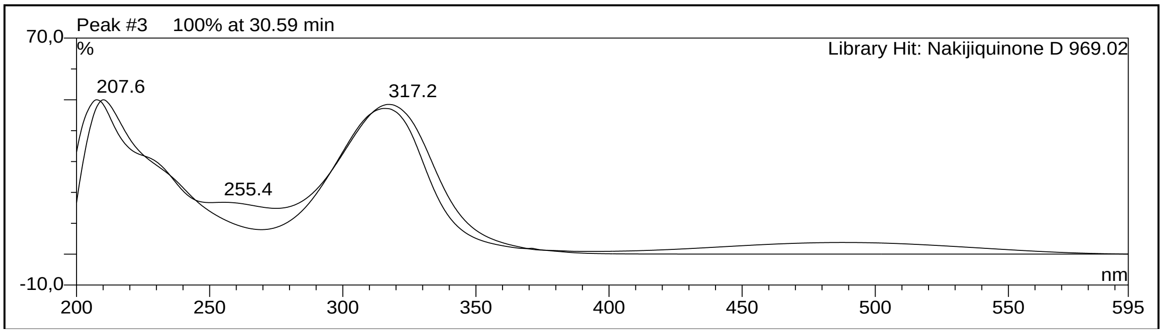

UV absorption of compound **1**

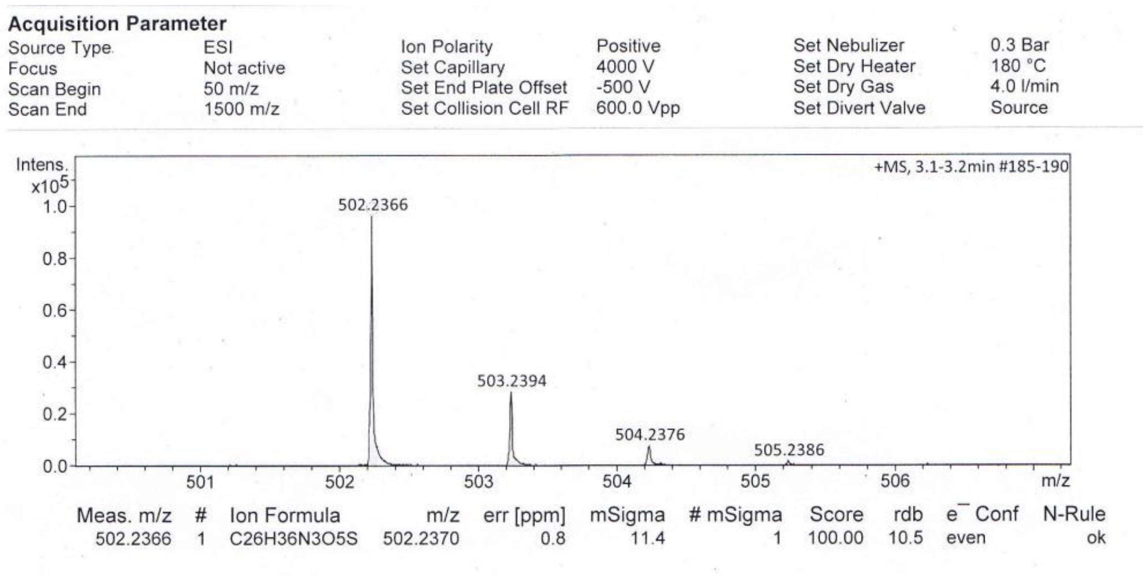

Figure S15. HRESIMS of compound **1**

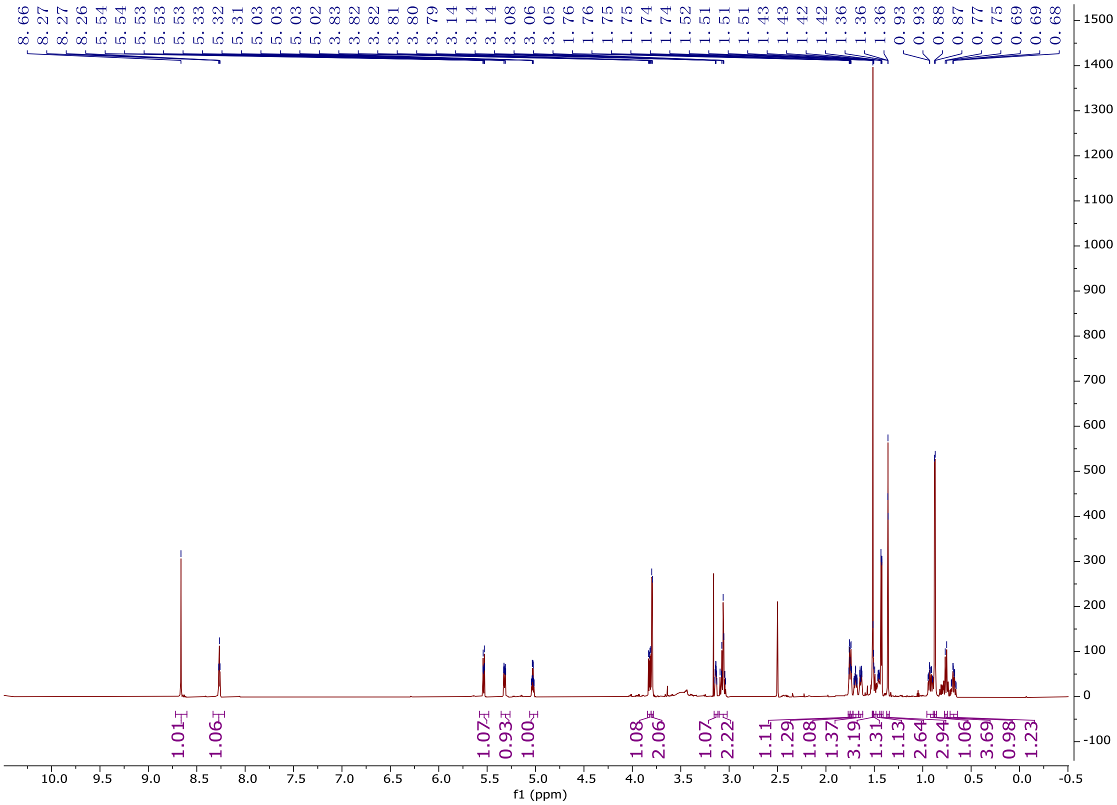

Figure S16. ^{1}H NMR (800M Hz, DMSO-d_4) spectrum of compound **1**

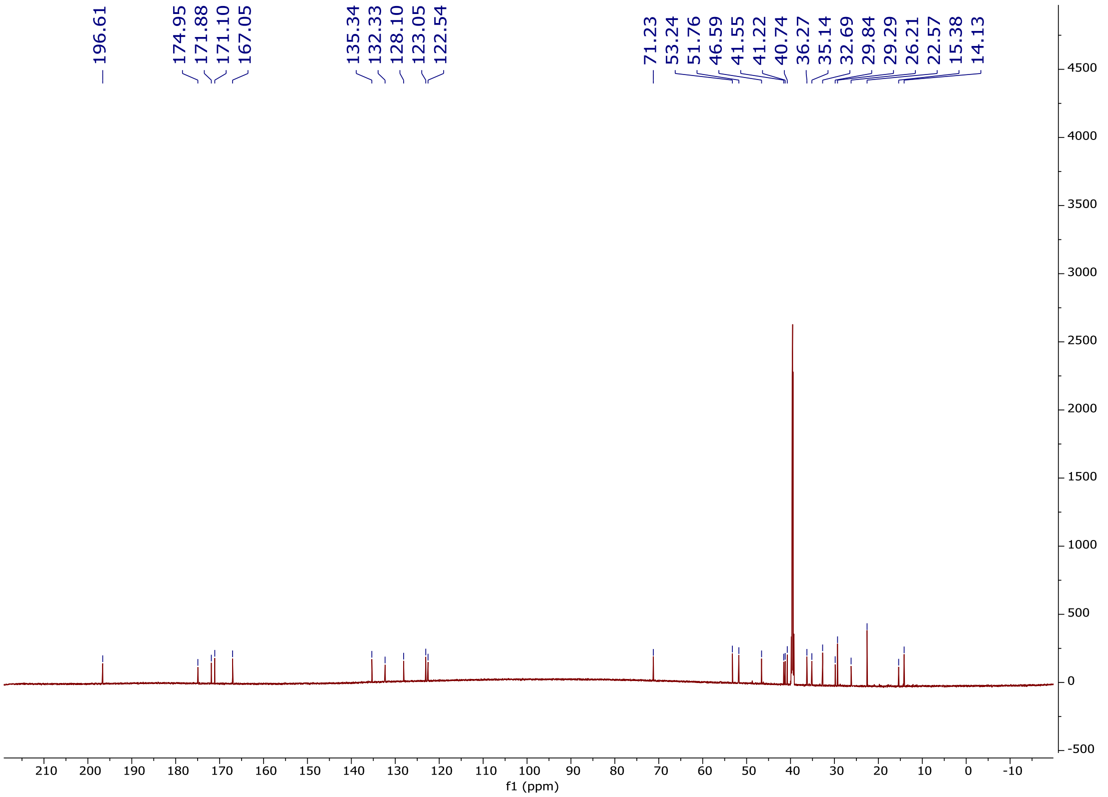

Figure S17. ^{13}C NMR (200M Hz, DMSO-d_6) spectrum of compound **1**

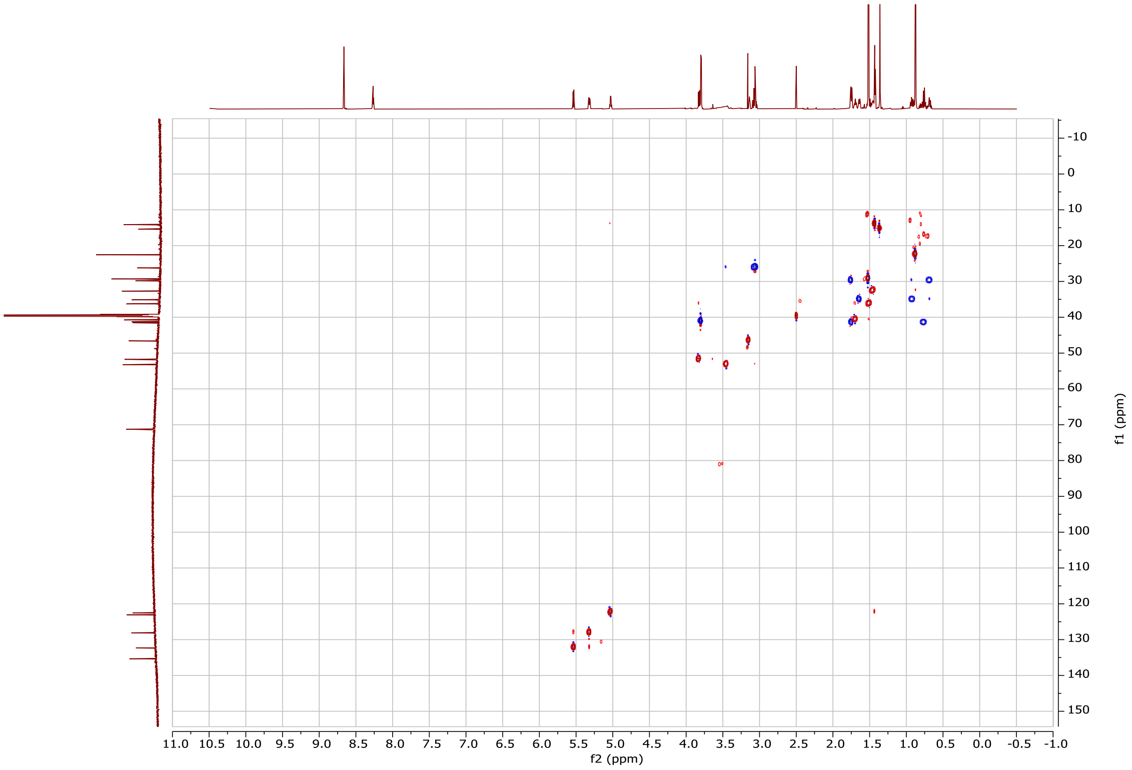

Figure S18. HSQC (DMSO-d_6) spectrum of compound **1**

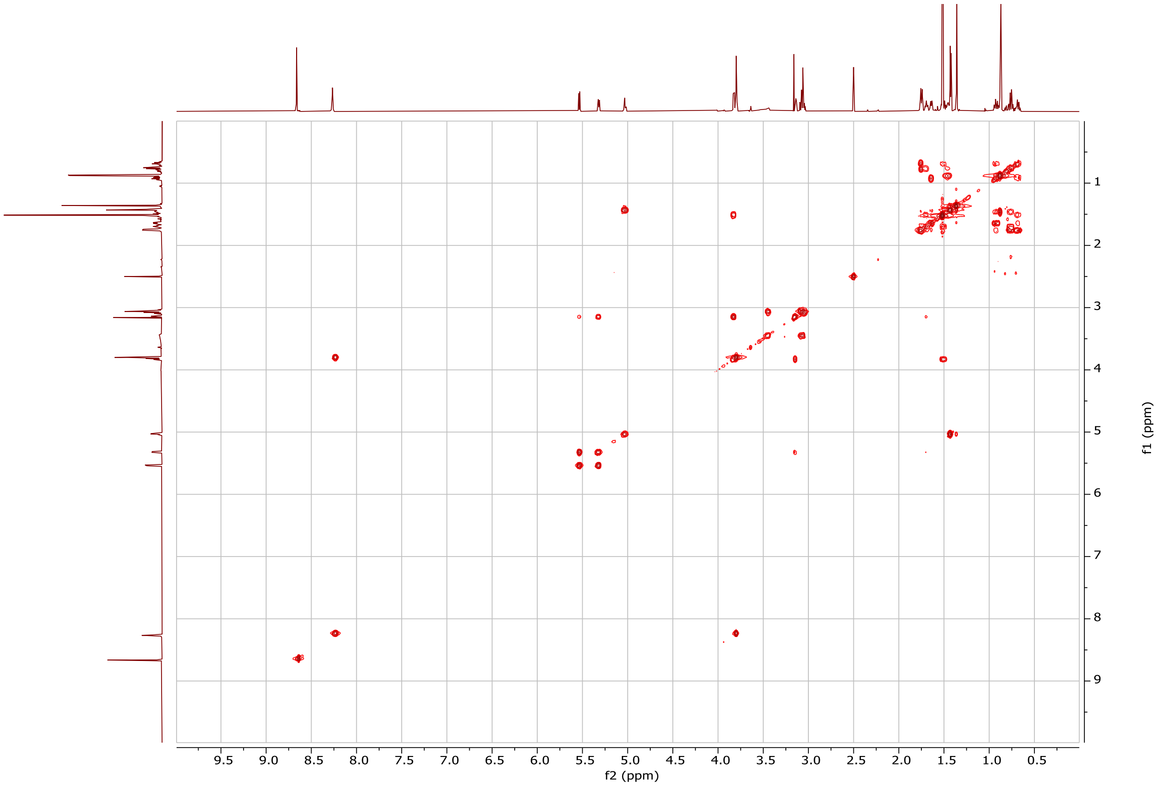

Figure S19. COSY (DMSO-d_6) spectrum of compound **1**

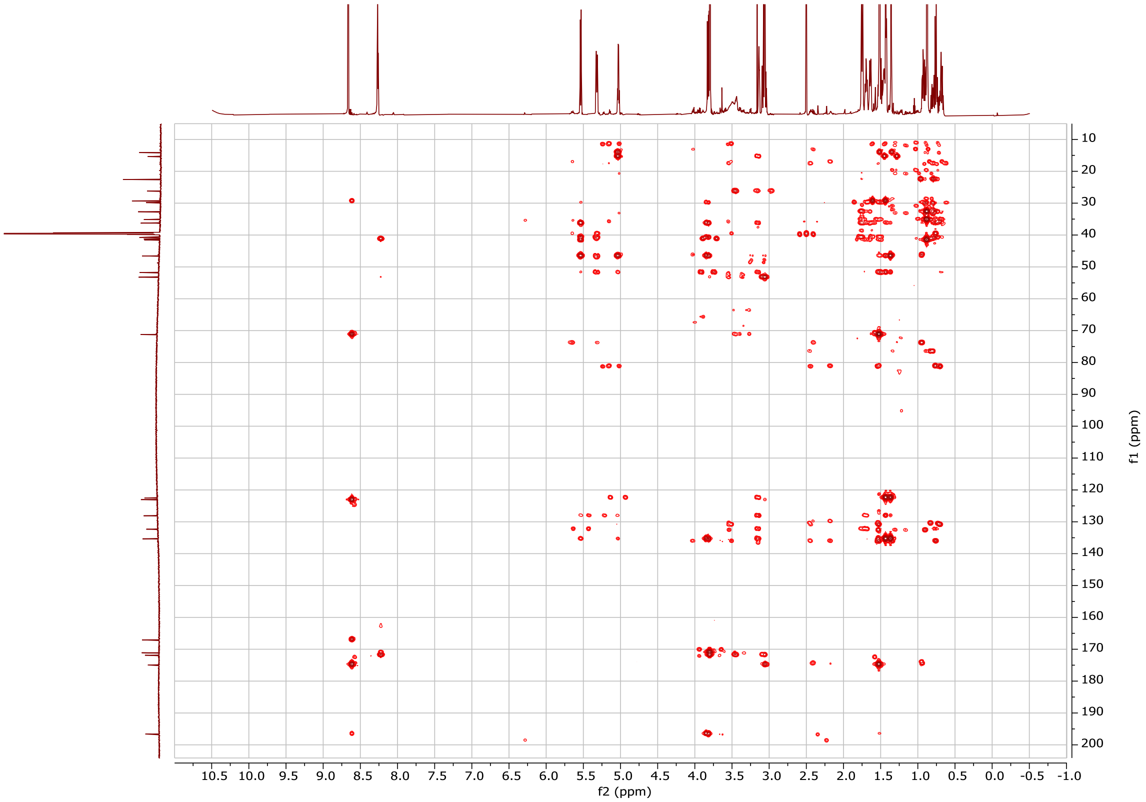

Figure S20. HMBC (DMSO-d_6) spectrum of compound **1**

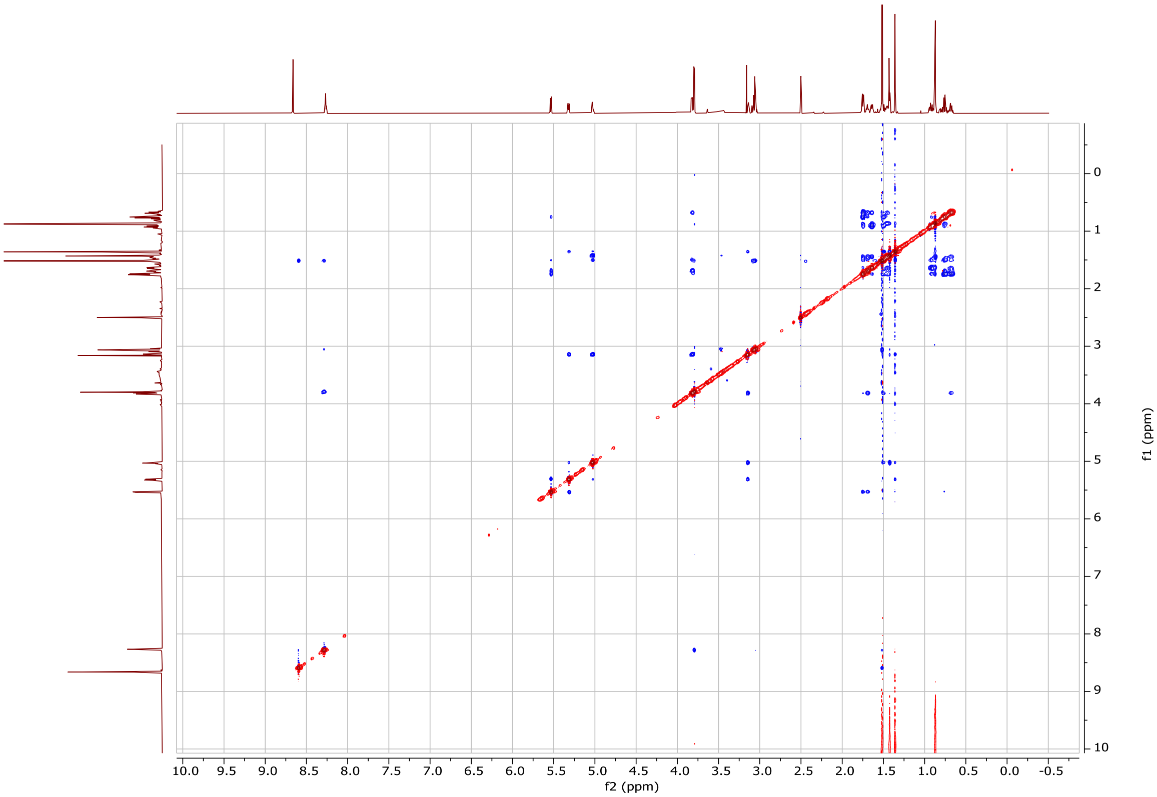

Figure S21. ROESY (DMSO-d_6) spectrum of compound **1**

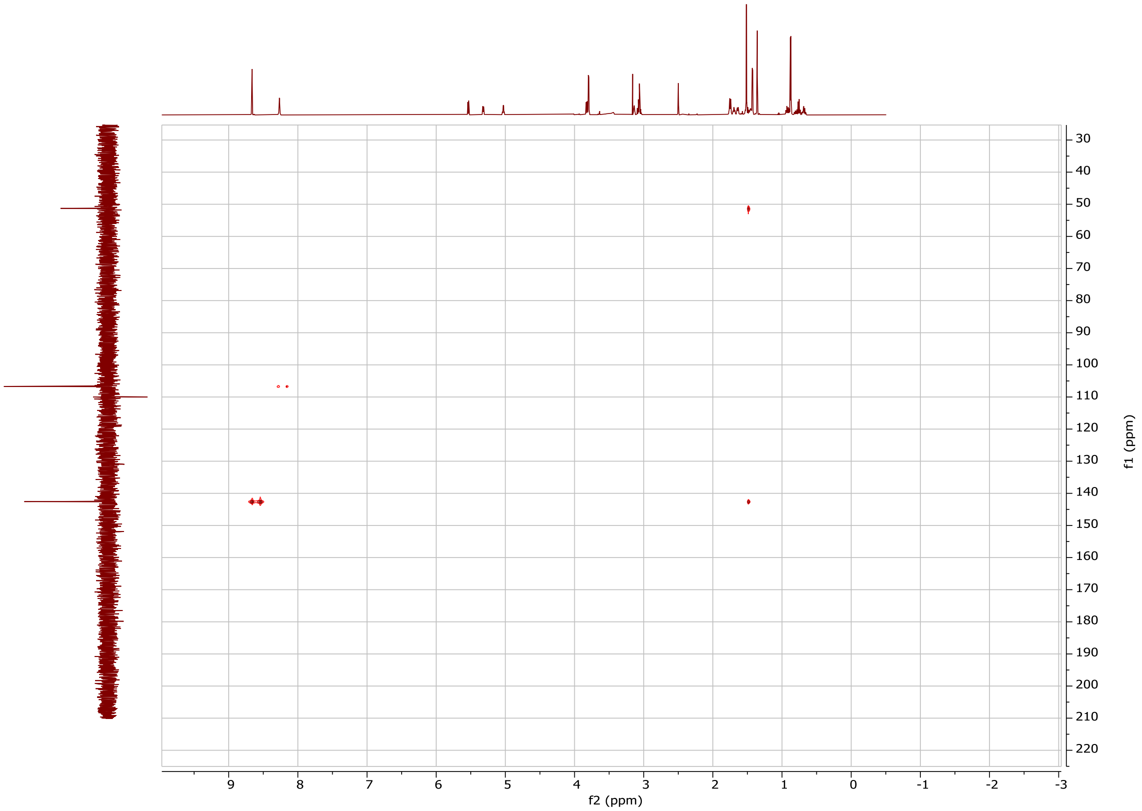

Figure S22. ^{1}H-^{15}N-HMBC (DMSO-d_6) spectrum of compound **1**

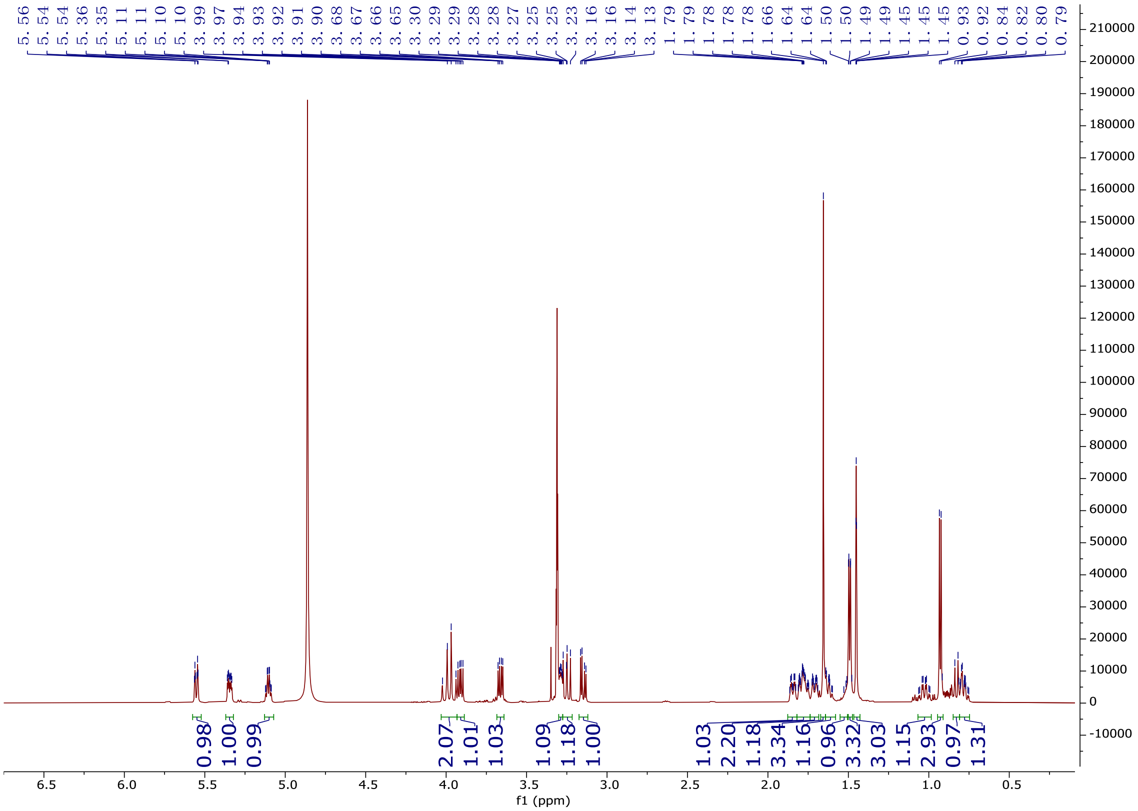

Figure S23. ^{1}H NMR (600M Hz, CD_3OD) spectrum of compound **1**

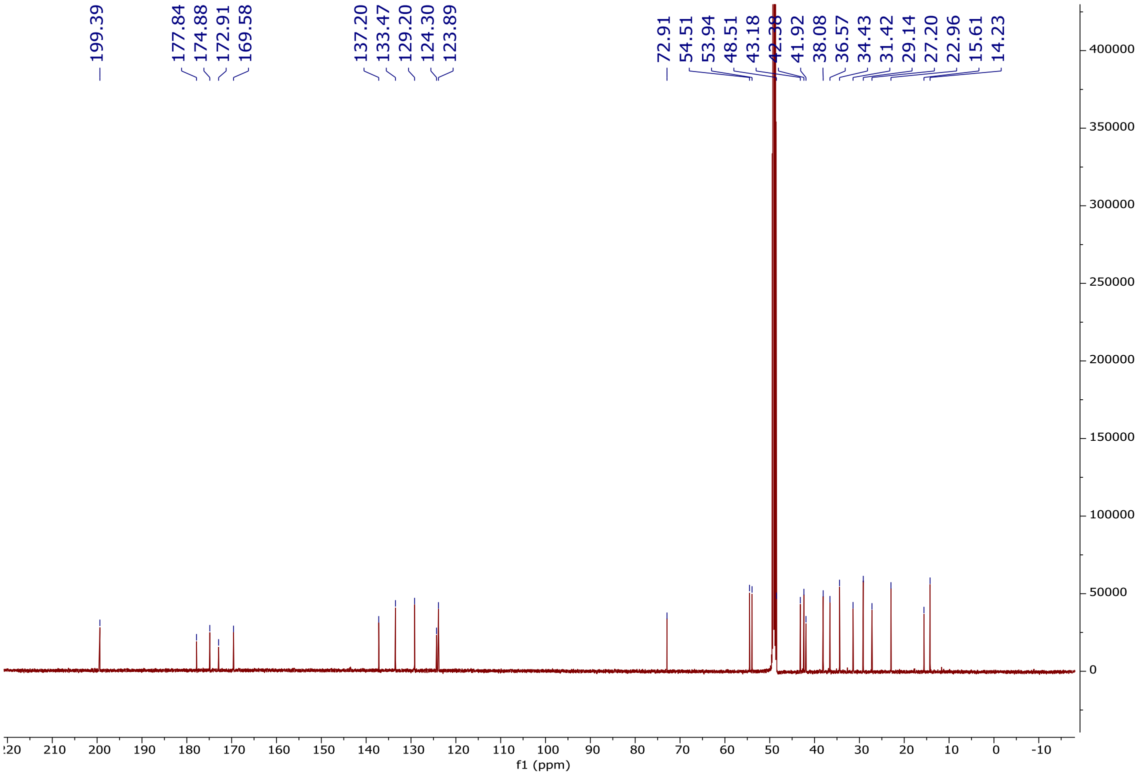

Figure S24. ^{13}C NMR (150M Hz, CD_3OD) spectrum of compound **1**

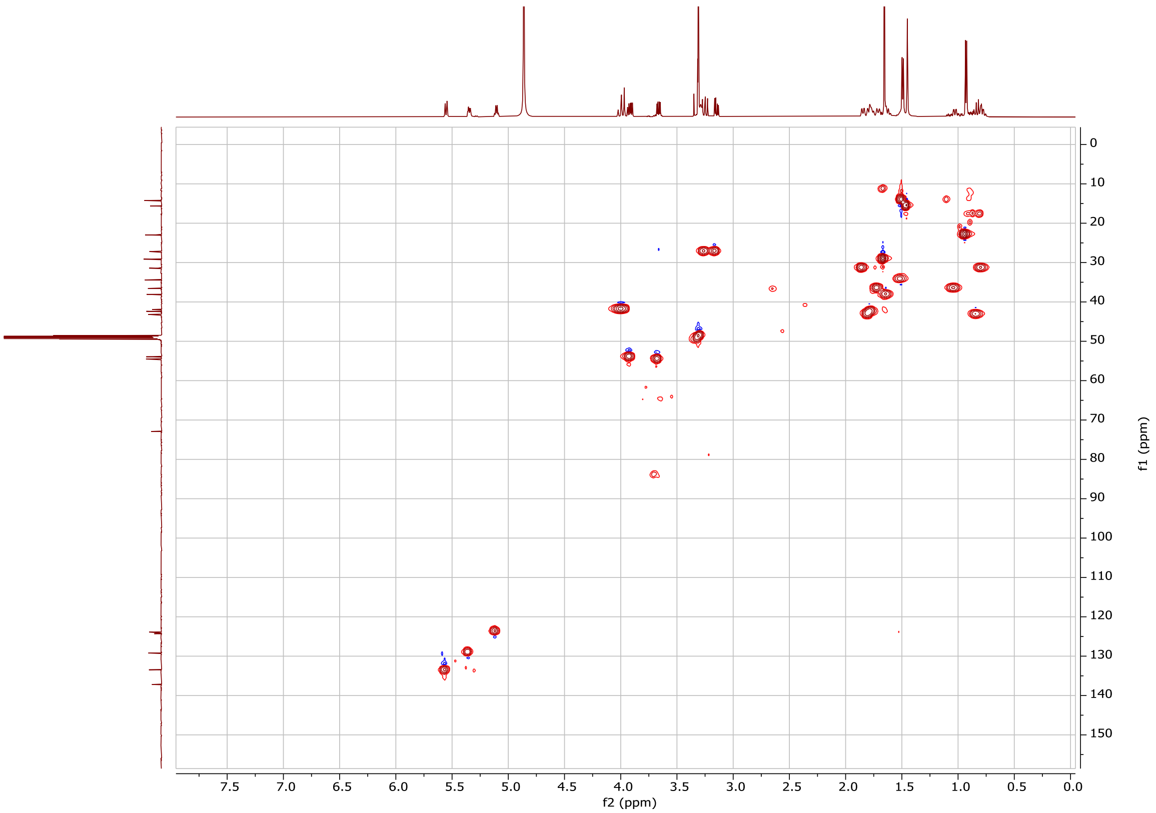

Figure S25. HSQC (CD_3OD) spectrum of compound **1**

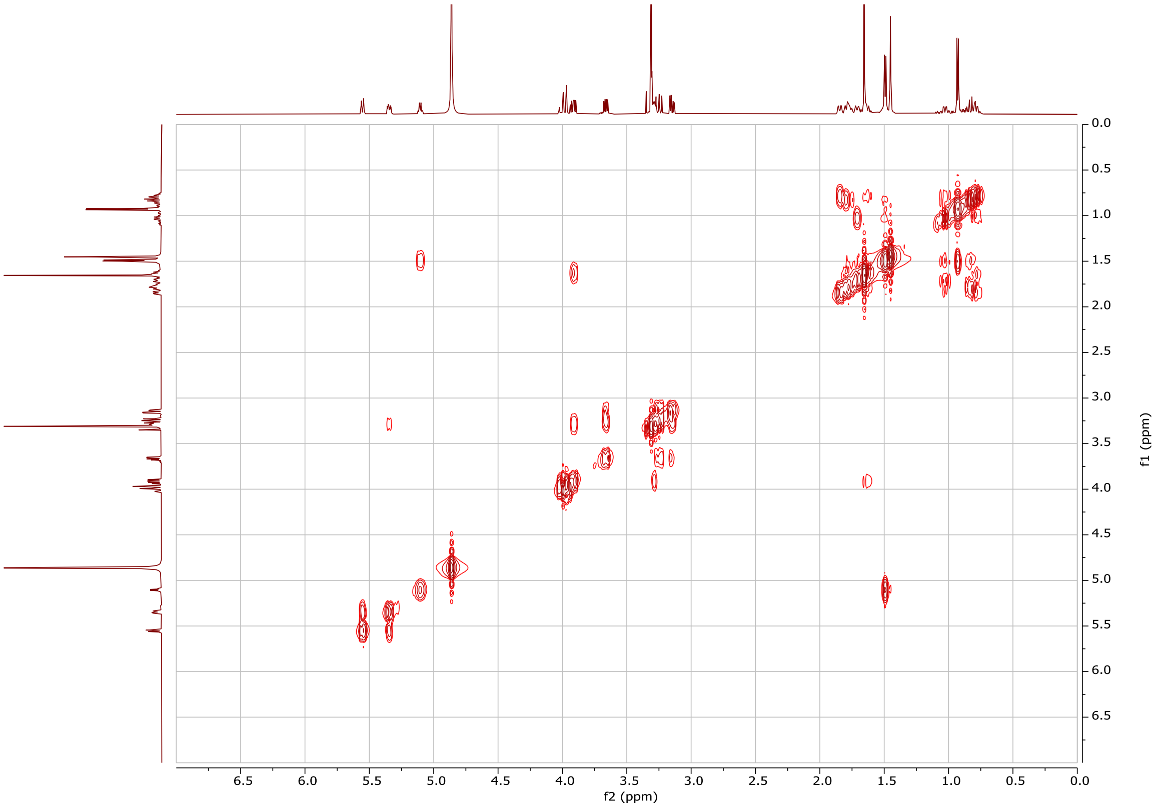

Figure S26. COSY (CD_3OD) spectrum of compound **1**

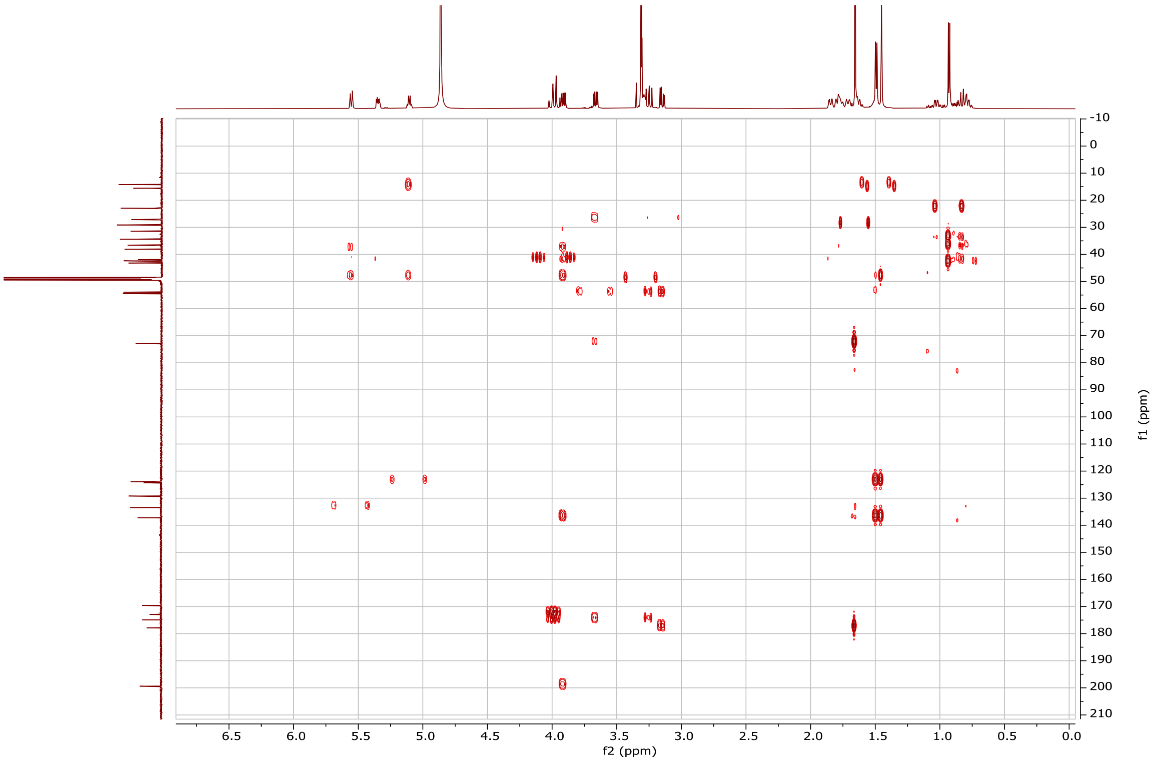

Figure S27. HMBC (CD_3OD) spectrum of compound **1**

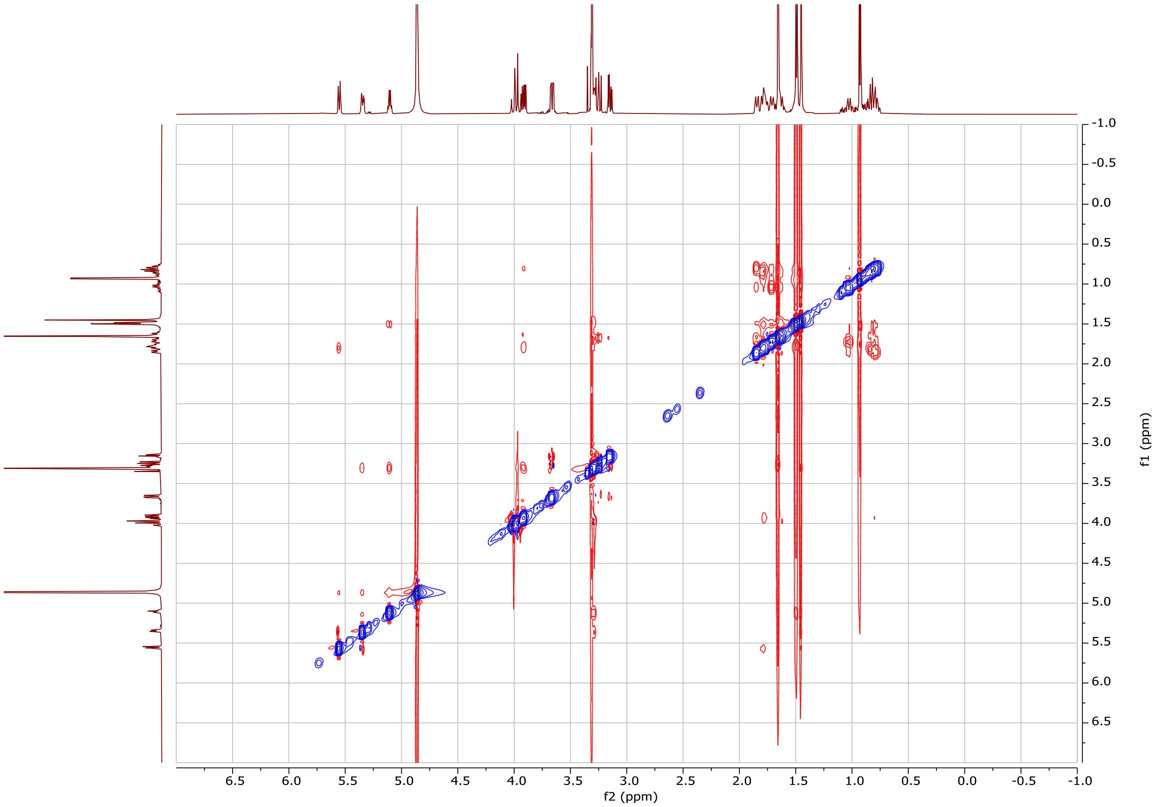

Figure S28. ROESY (CD_3OD) spectrum of compound **1**

Aplospojaveedins B (2)

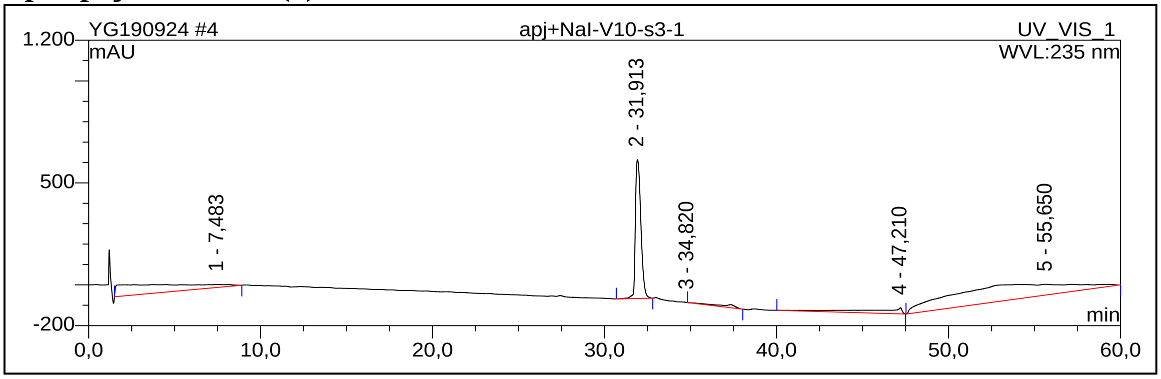

Figure S29. HPLC chromatogram of Aplospojaveedins B (2)

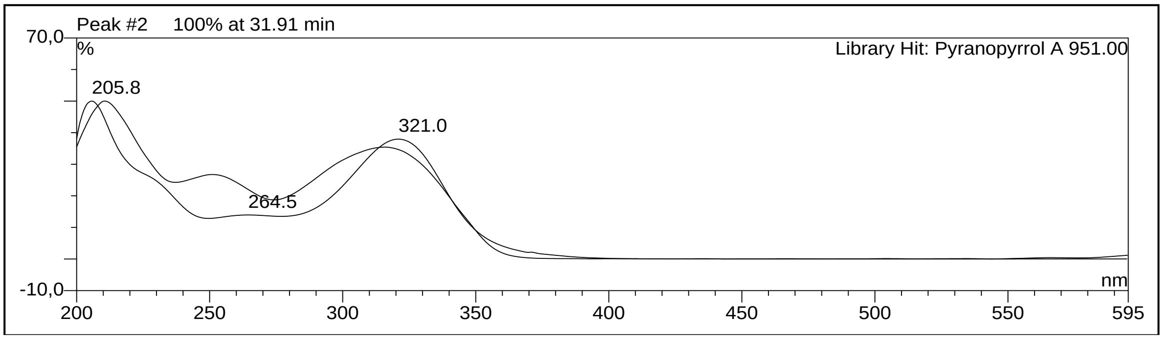

UV absorption of compound **2**

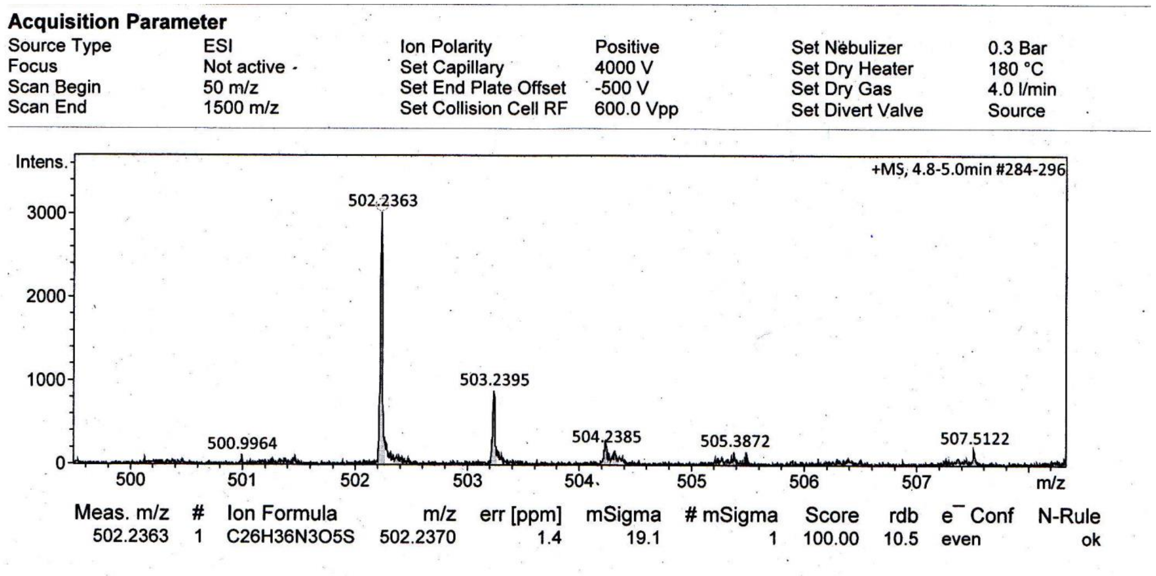

Figure S30. HRESIMS of compound **2**

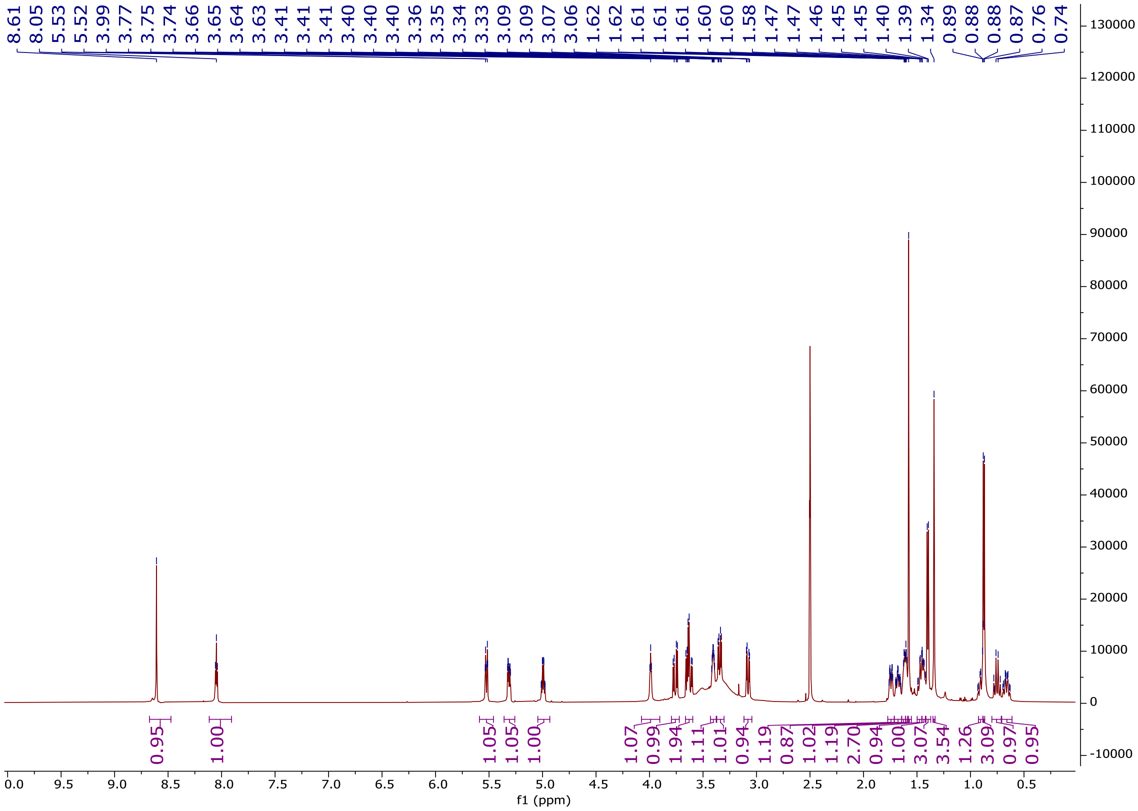

Figure S31. ^{1}H NMR (600M Hz, DMSO-d_4) spectrum of compound **2**

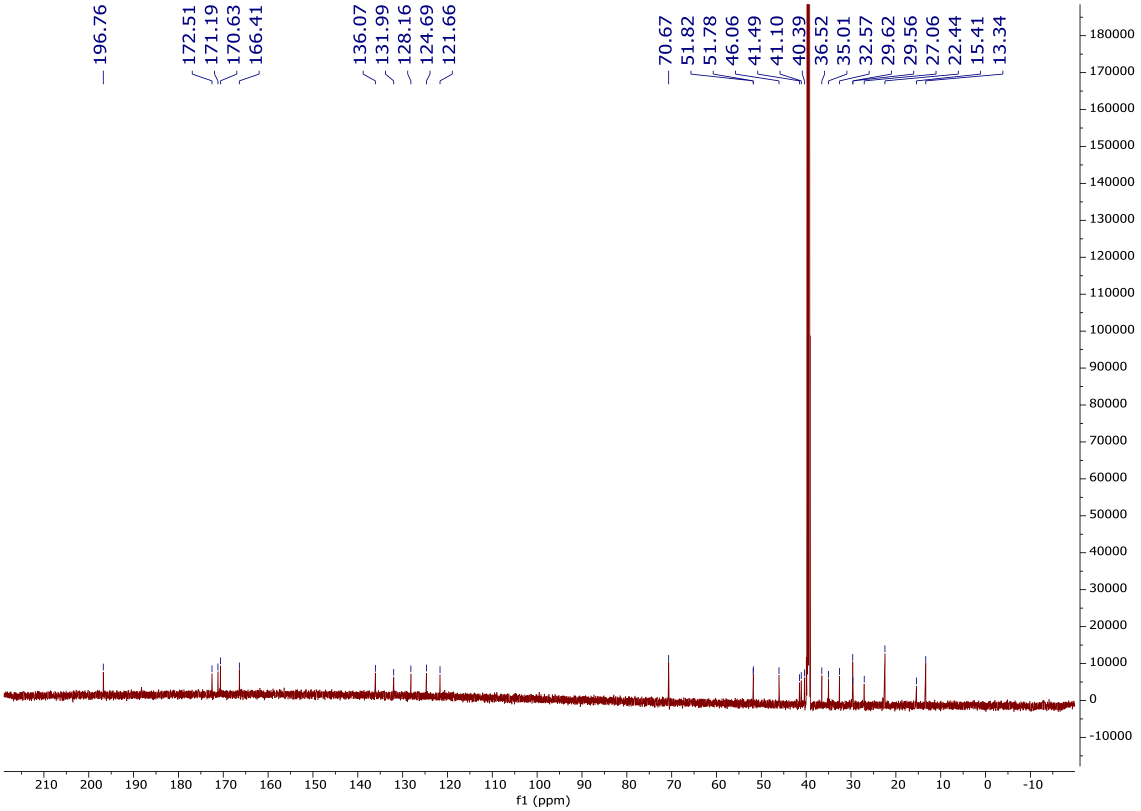

Figure S32. ^{13}C NMR (150M Hz, DMSO-d_6) spectrum of compound **2**

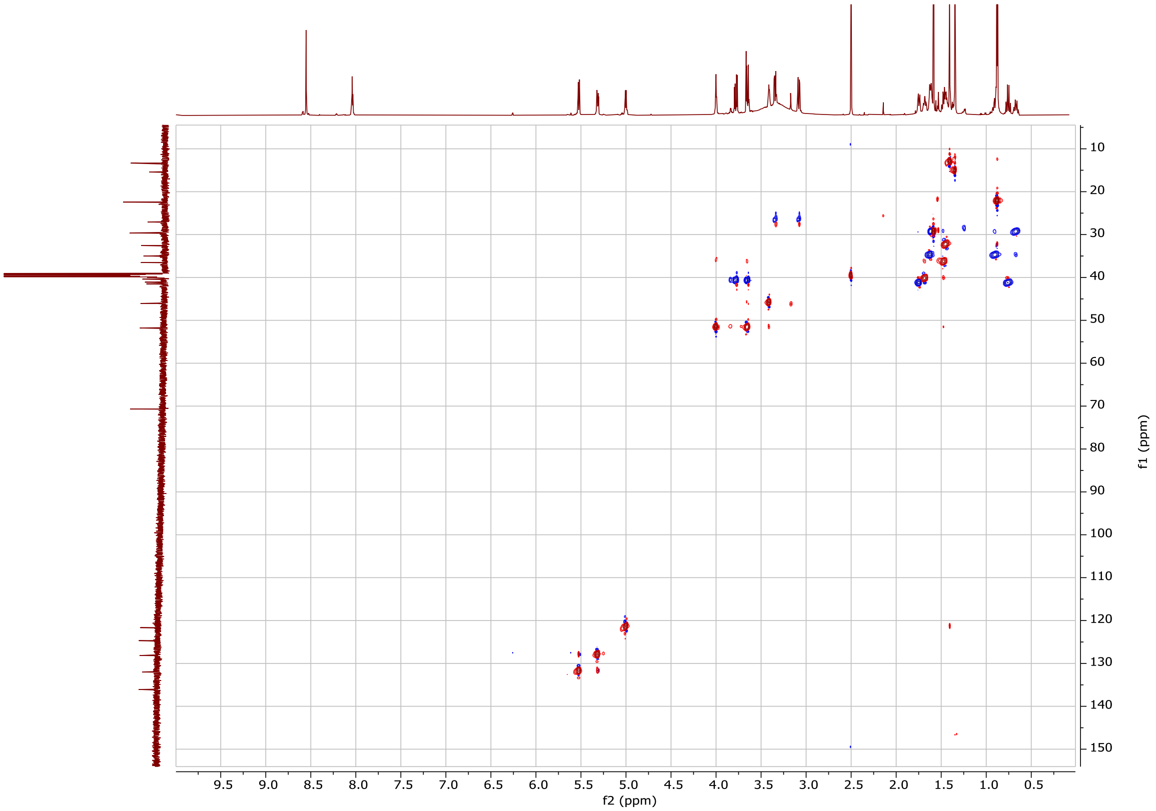

Figure S33. HSQC (DMSO-d_6) spectrum of compound **2**

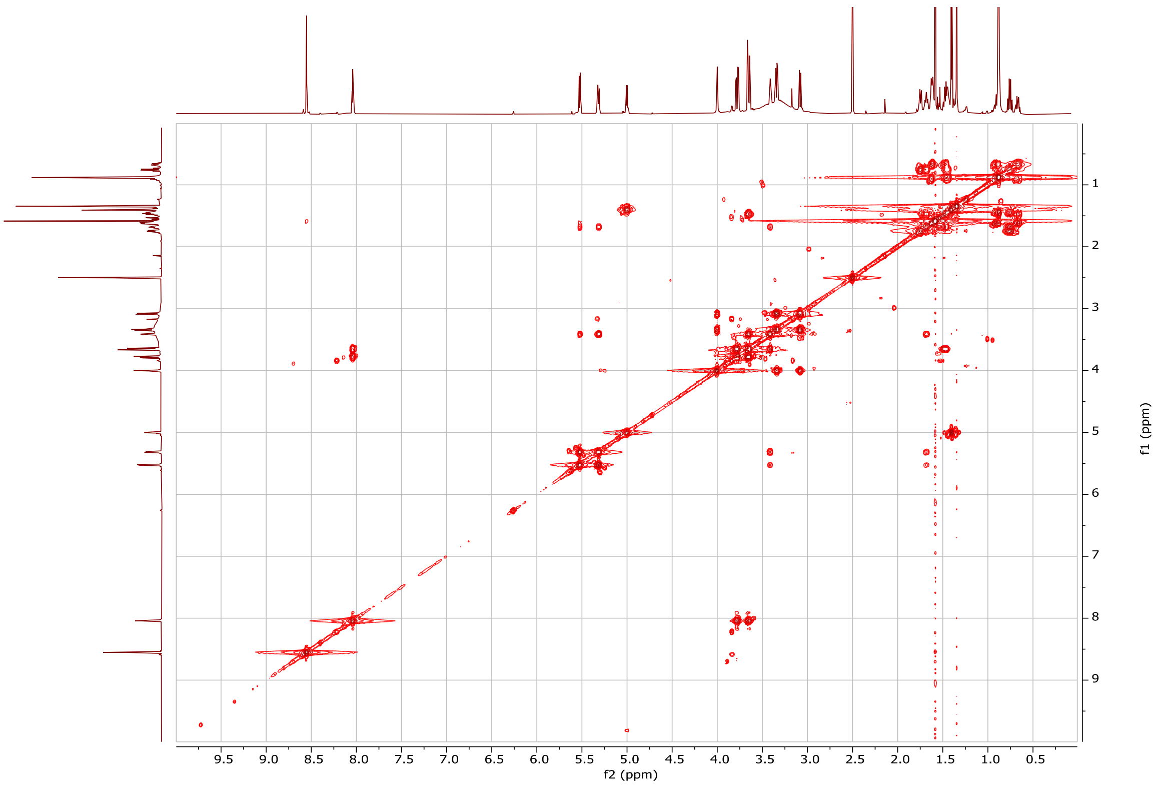

Figure S34. COSY (DMSO-d_6) spectrum of compound **2**

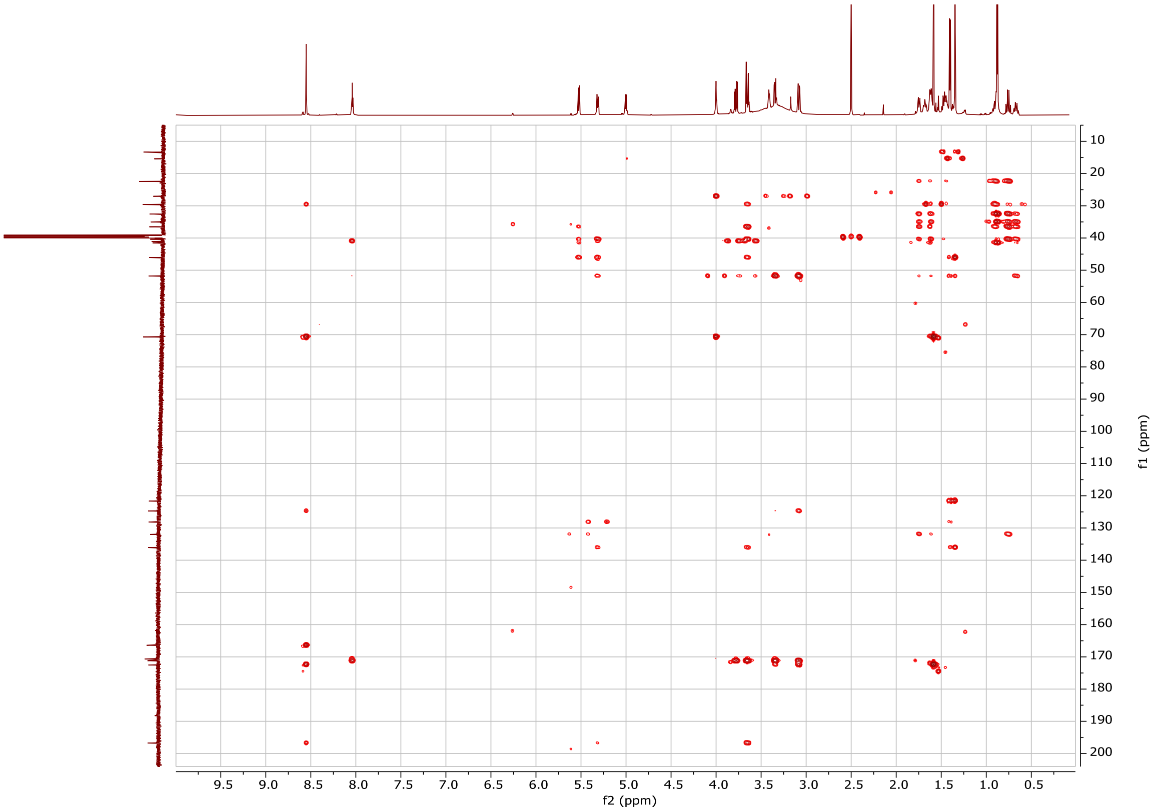

Figure S35. HMBC (DMSO-d_6) spectrum of compound **2**

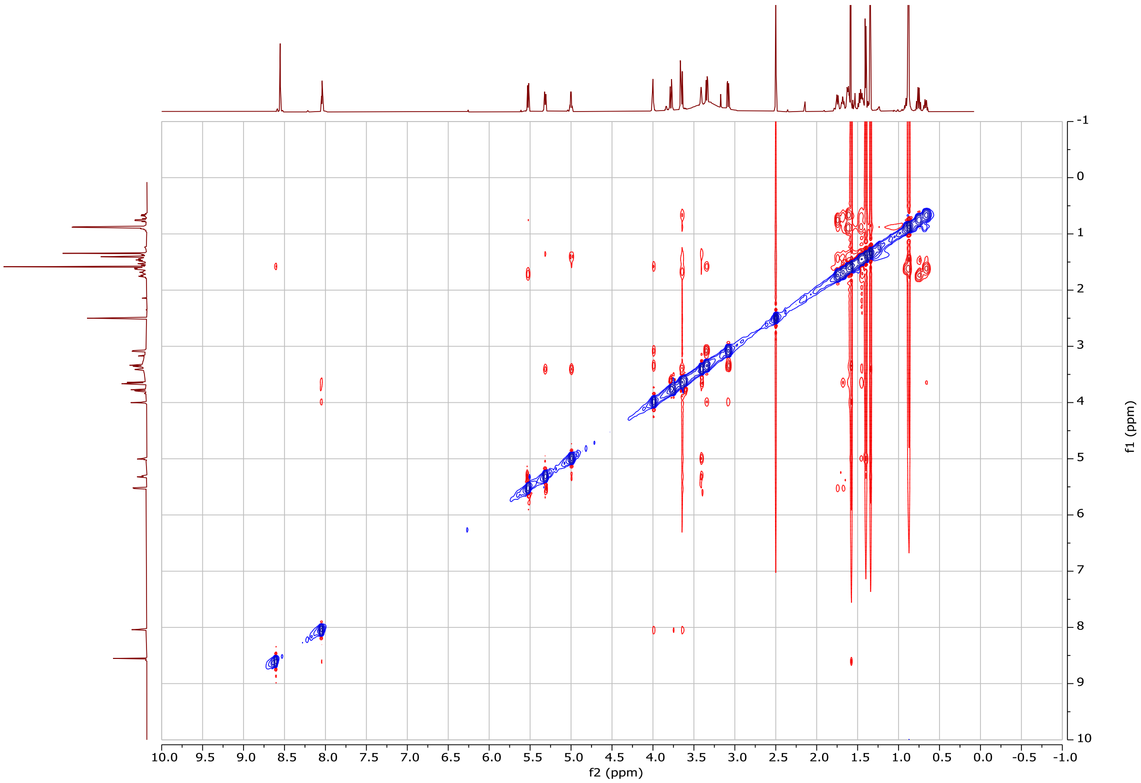

Figure S36. ROESY (DMSO-d_6) spectrum of compound **2**

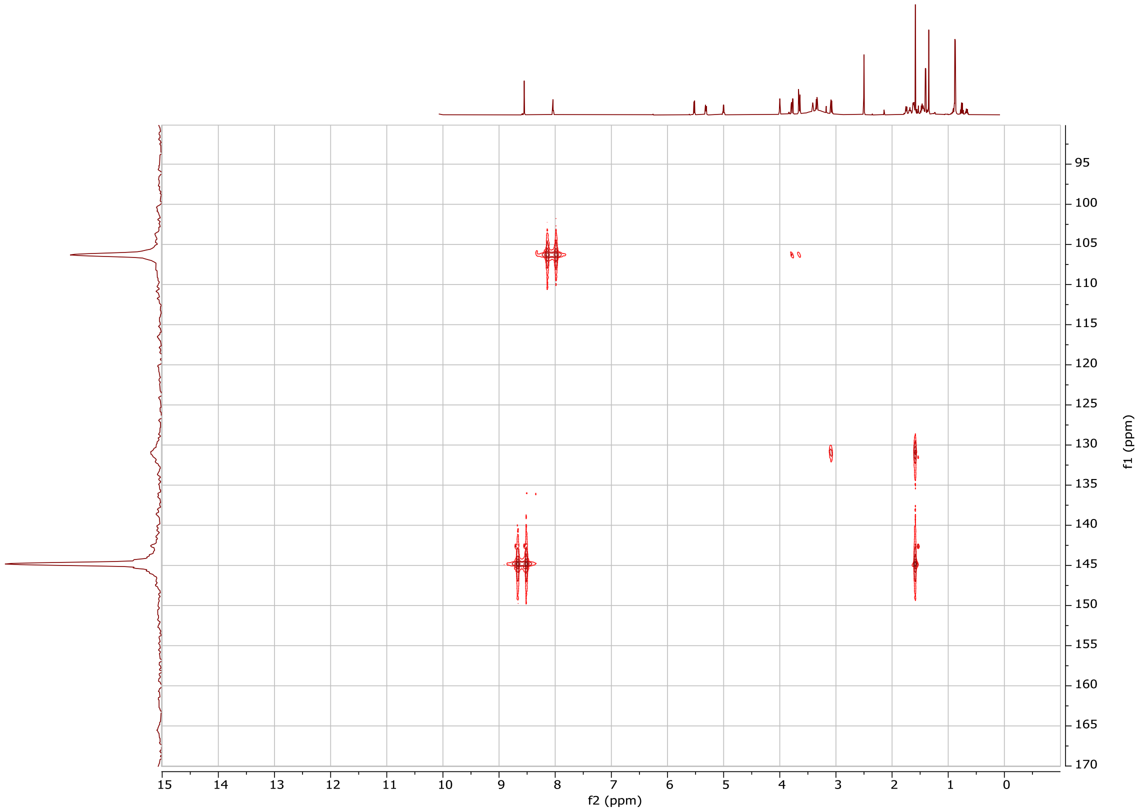

Figure S37. ^{1}H-^{15}N-HMBC (DMSO-d_6) spectrum of compound **2**

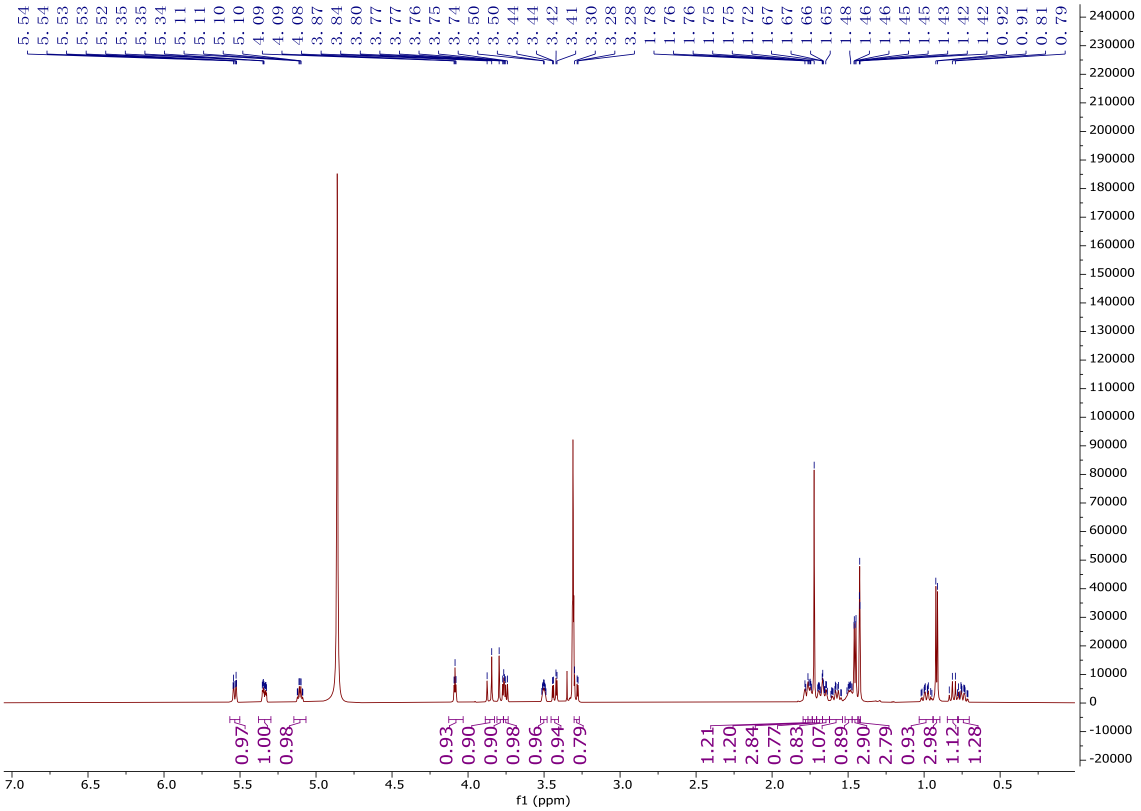

Figure S38. ^{1}H NMR (600M Hz, CD_3OD) spectrum of compound **2**

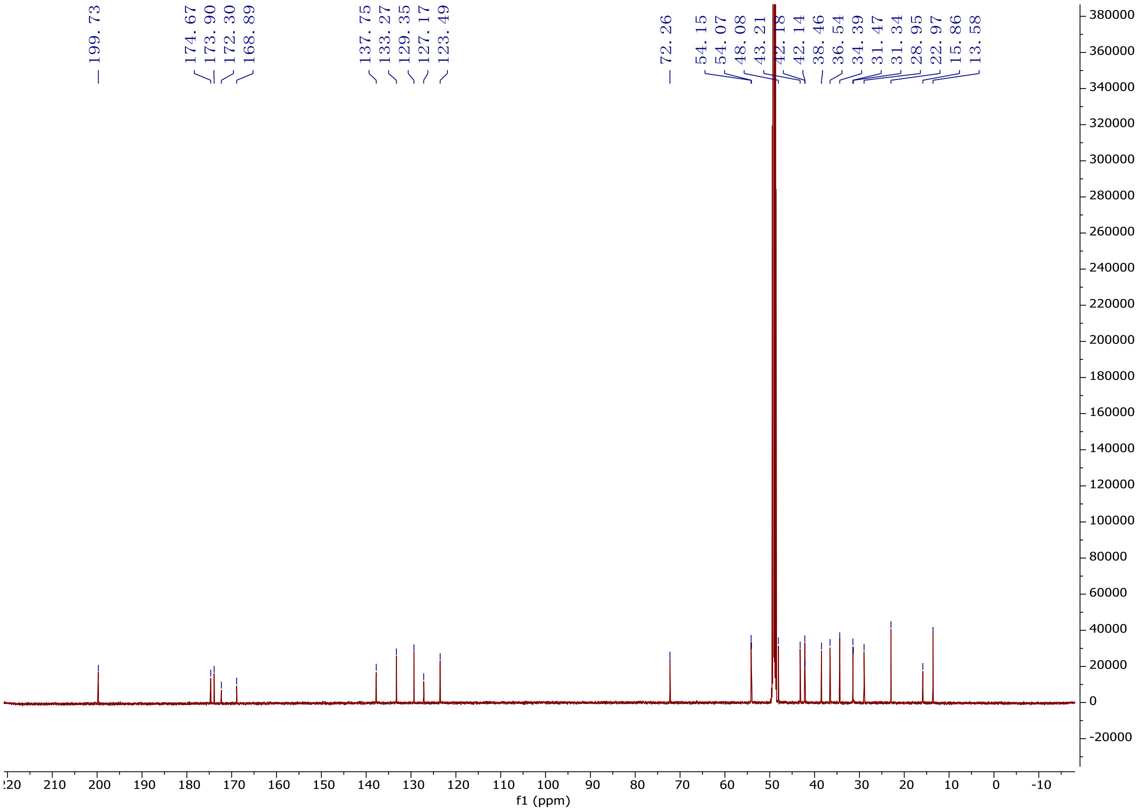

Figure S39. ^{13}C NMR (150M Hz, CD_3OD) spectrum of compound **2**

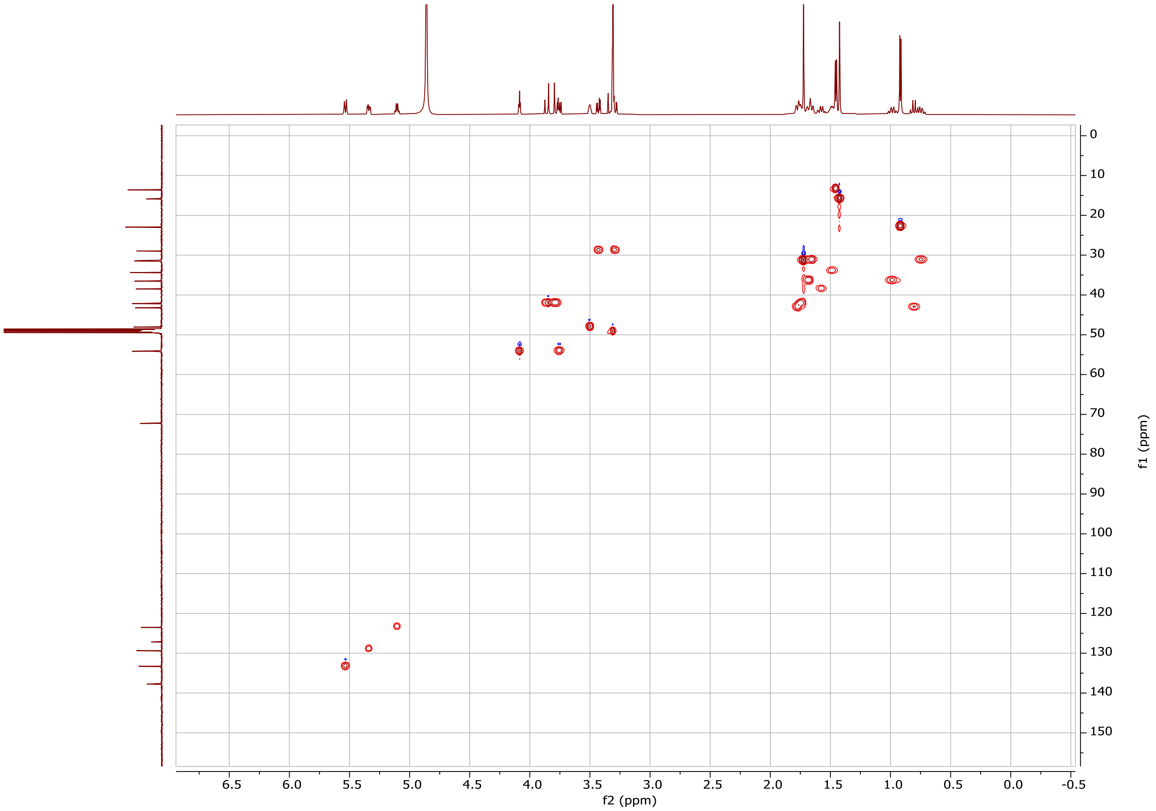

Figure S40. HSQC (CD_3OD) spectrum of compound **2**

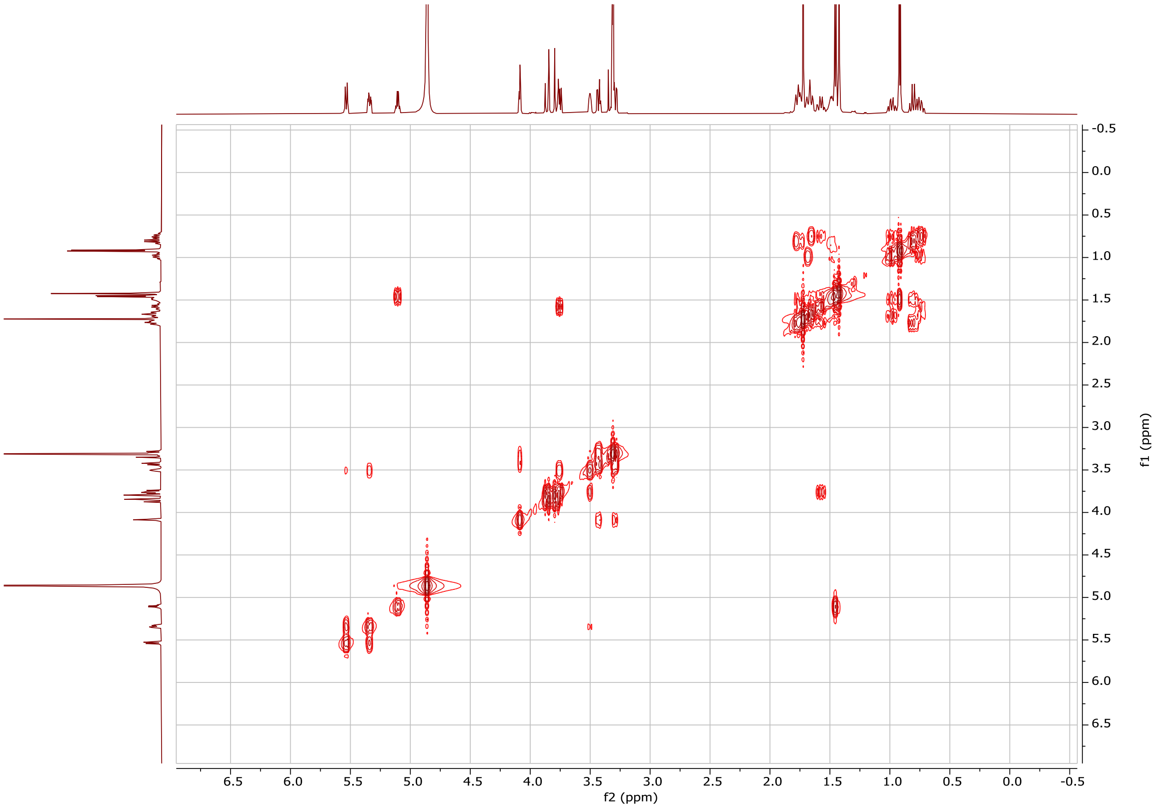

Figure S41. COSY (CD_3OD) spectrum of compound **2**

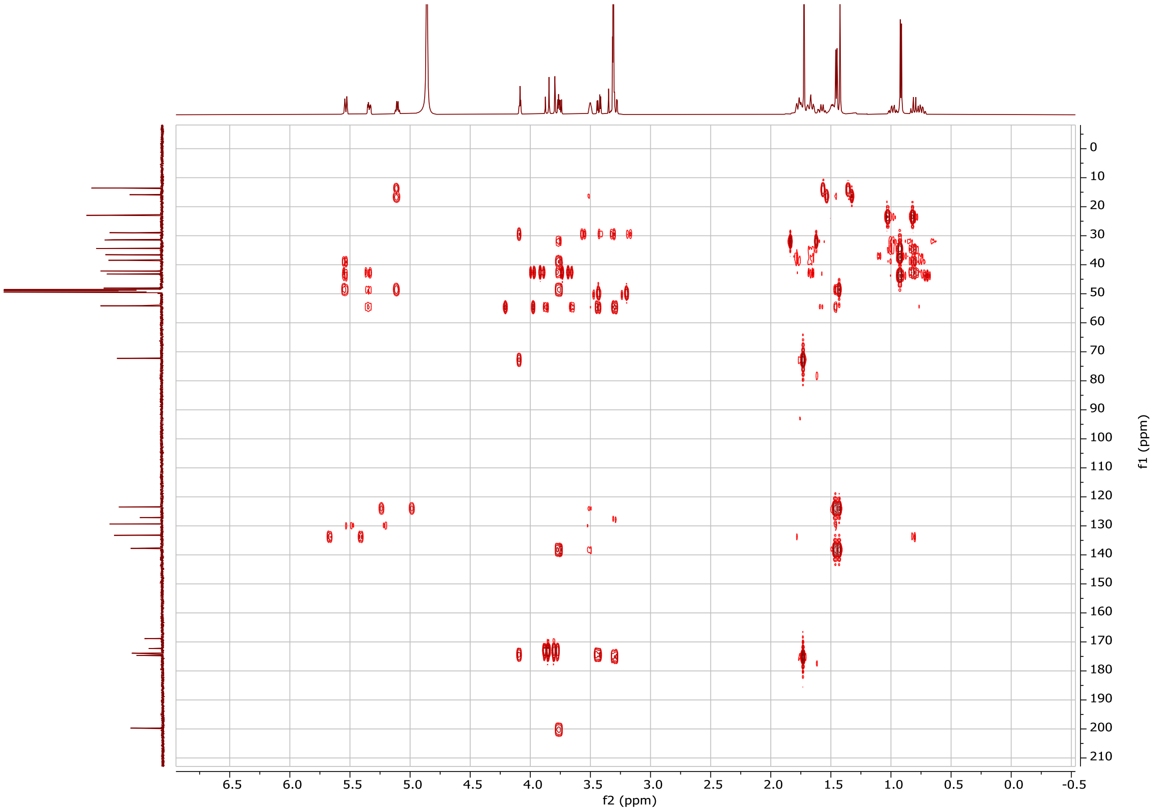

Figure S42. HMBC (CD3OD) spectrum of compound **2**

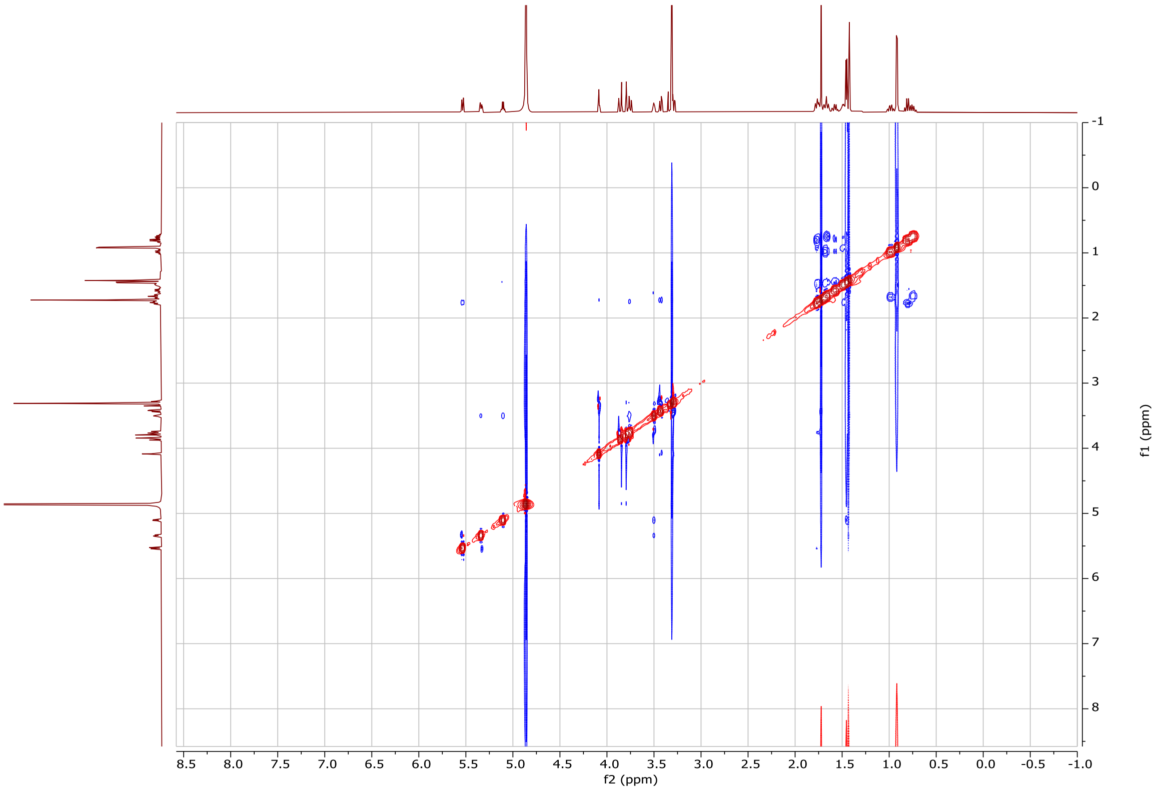

Figure S43. ROESY (CD3OD) spectrum of compound **2**

Aplospojaveedins C (3)

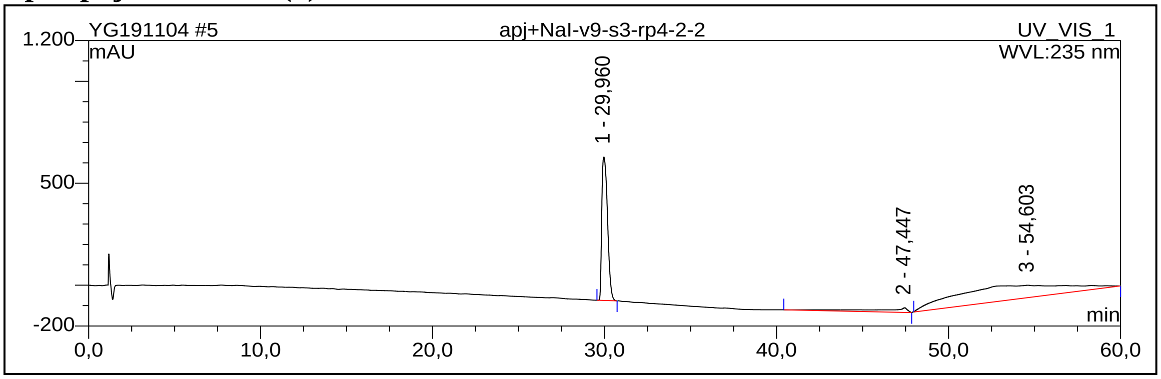

Figure S44. HPLC chromatogram of Aplospojaveedins C (3)

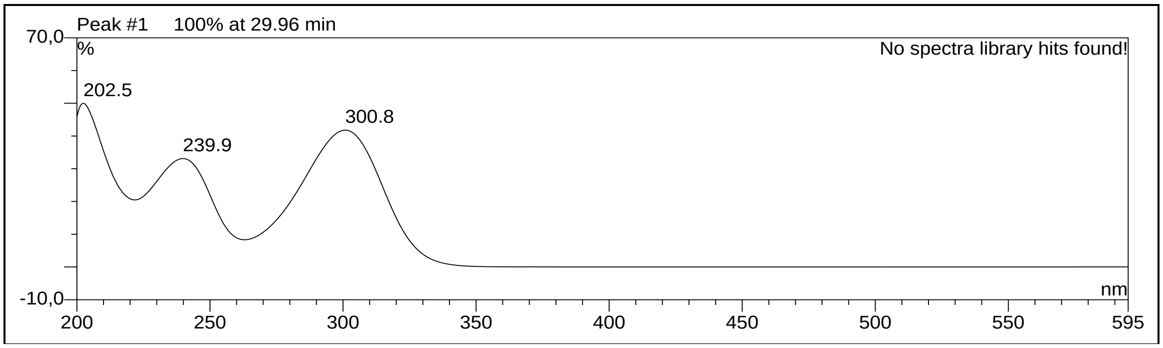

UV absorption of compound **3**

Acquisition Parameter

Source Type	ESI	Ion Polarity	Positive	Set Nebulizer	0.3 Bar
Focus	Not active	Set Capillary	4000 V	Set Dry Heater	180 °C
Scan Begin	50 m/z	Set End Plate Offset	-500 V	Set Dry Gas	4.0 l/min
Scan End	1500 m/z	Set Collision Cell RF	600.0 Vpp	Set Divert Valve	Source

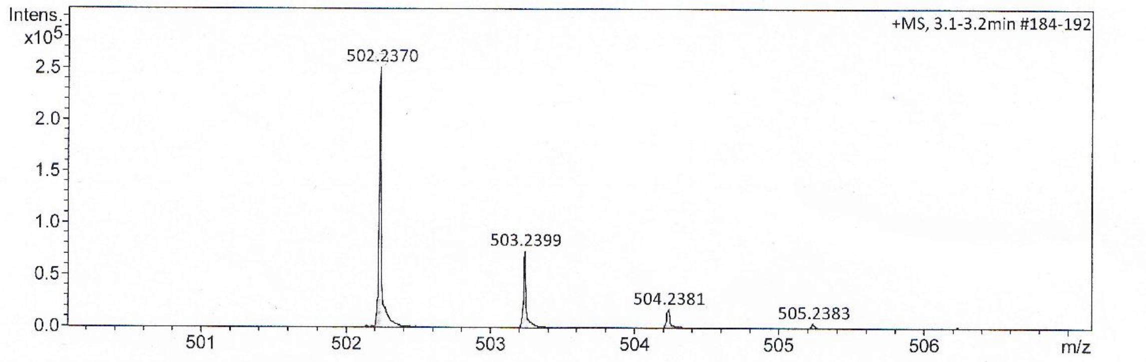

Meas. m/z	#	Ion Formula	m/z	err [ppm]	mSigma	# mSigma	Score	rdb	e⁻ Conf	N-Rule
502.2370	1	C26H36N3O5S	502.2370	0.0	17.0	1	100.00	10.5	even	ok
	2	C27H32N7OS	502.2384	2.7	23.7	2	43.60	15.5	even	ok
	3	C30H28N7O	502.2350	-4.0	30.7	3	22.78	20.5	even	ok
	4	C34H32NO3	502.2377	1.3	41.7	4	41.09	19.5	even	ok

Figure S45. HRESIMS of compound **3**

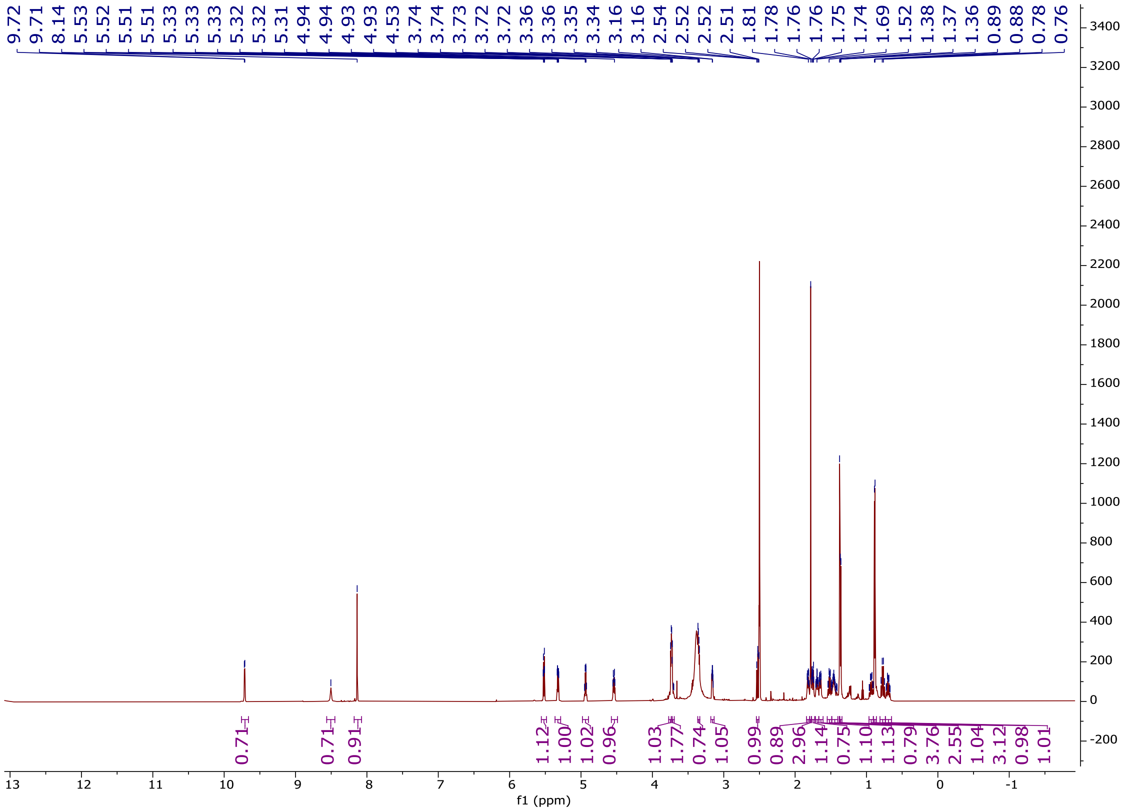

Figure S46. ^{1}H NMR (750M Hz, DMSO-d_4) spectrum of compound **3**

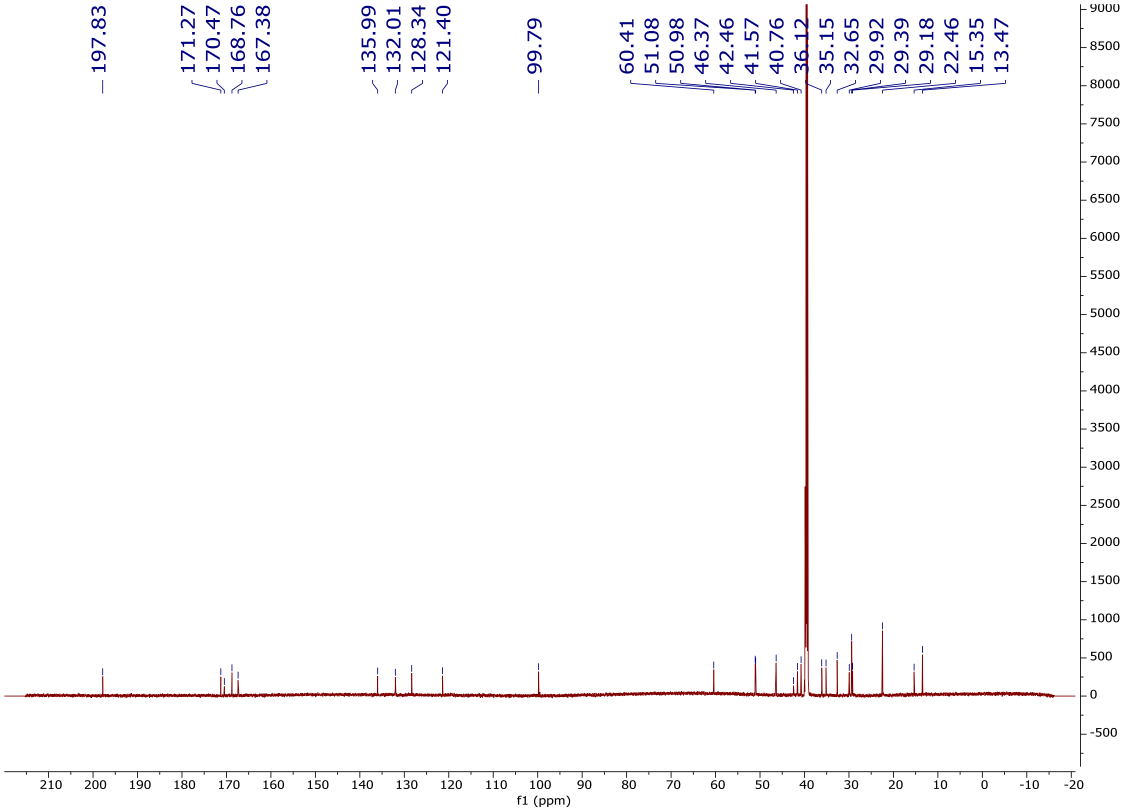

Figure S47. ^{13}C NMR (188M Hz, DMSO-d_4) spectrum of compound **3**

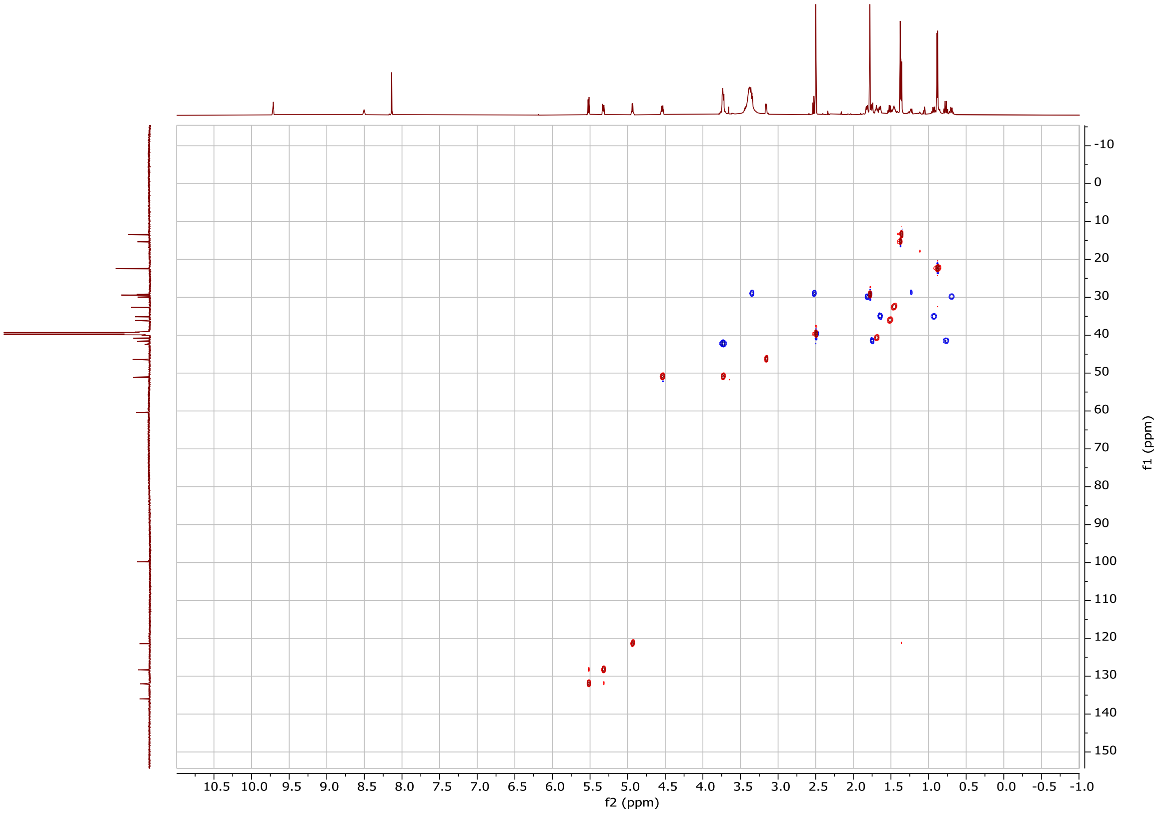

Figure S48. HSQC (DMSO-d_4) spectrum of compound **3**

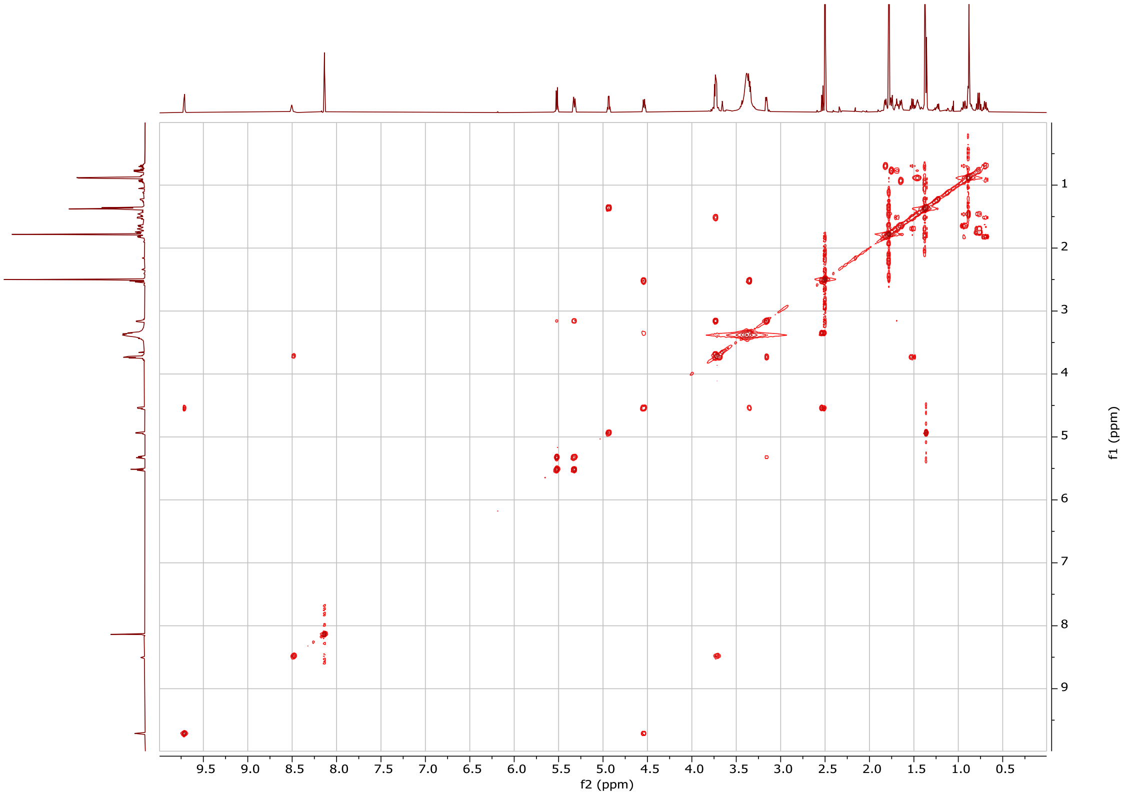

Figure S49.COSY (DMSO-d_4) spectrum of compound **3**

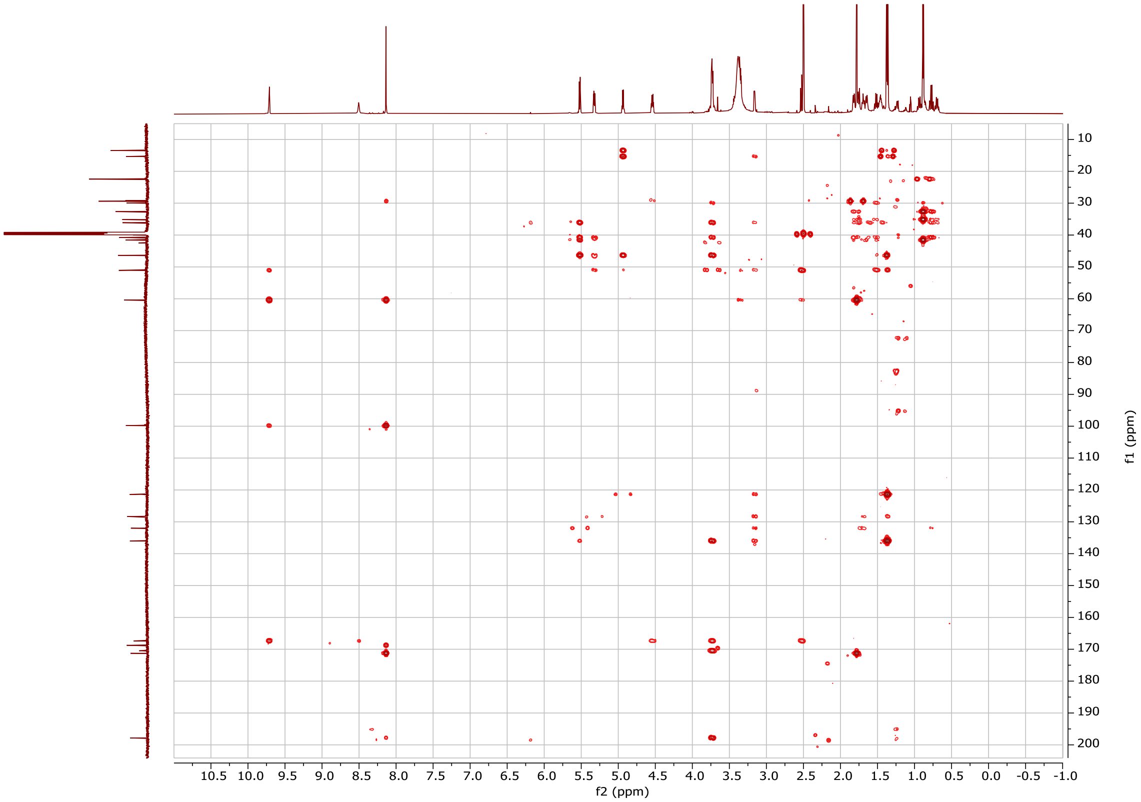

Figure S50.HMBC (DMSO-d_4) spectrum of compound **3**

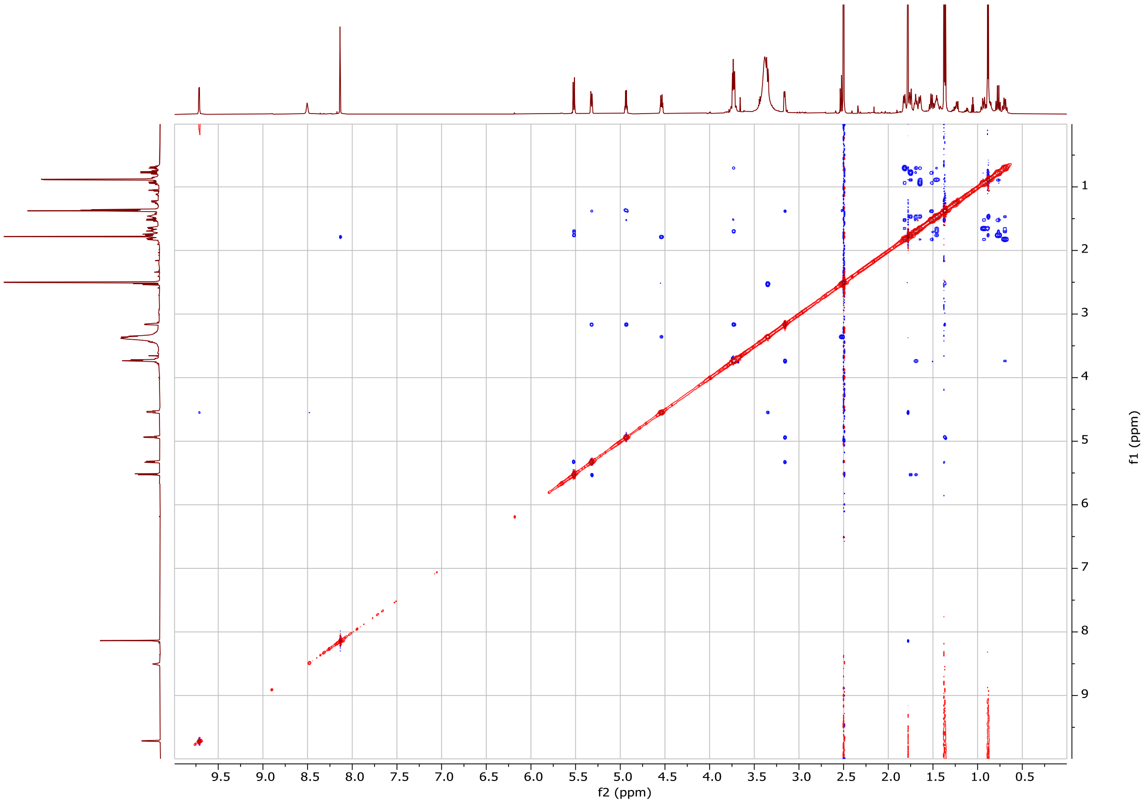

Figure S51. ROESY (DMSO-d_4) spectrum of compound **3**

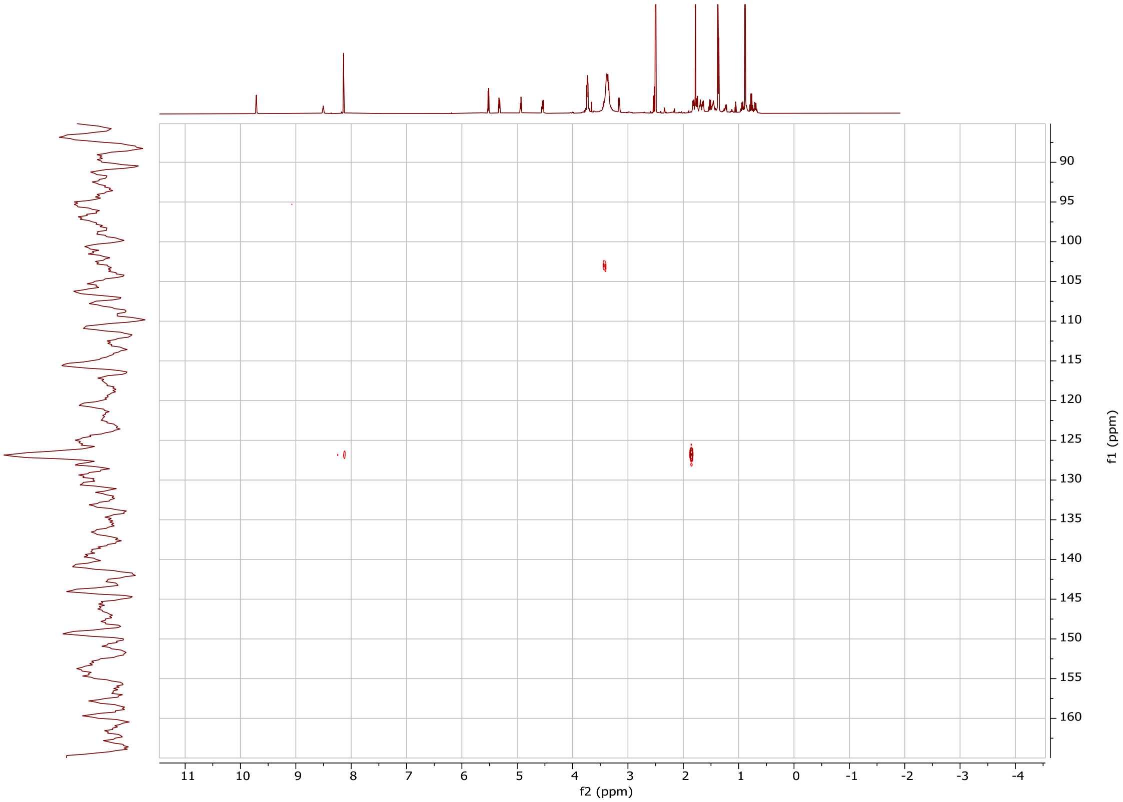

Figure S52. ^{1}H-^{15}N-HMBC (DMSO-d_6) spectrum of compound **3**

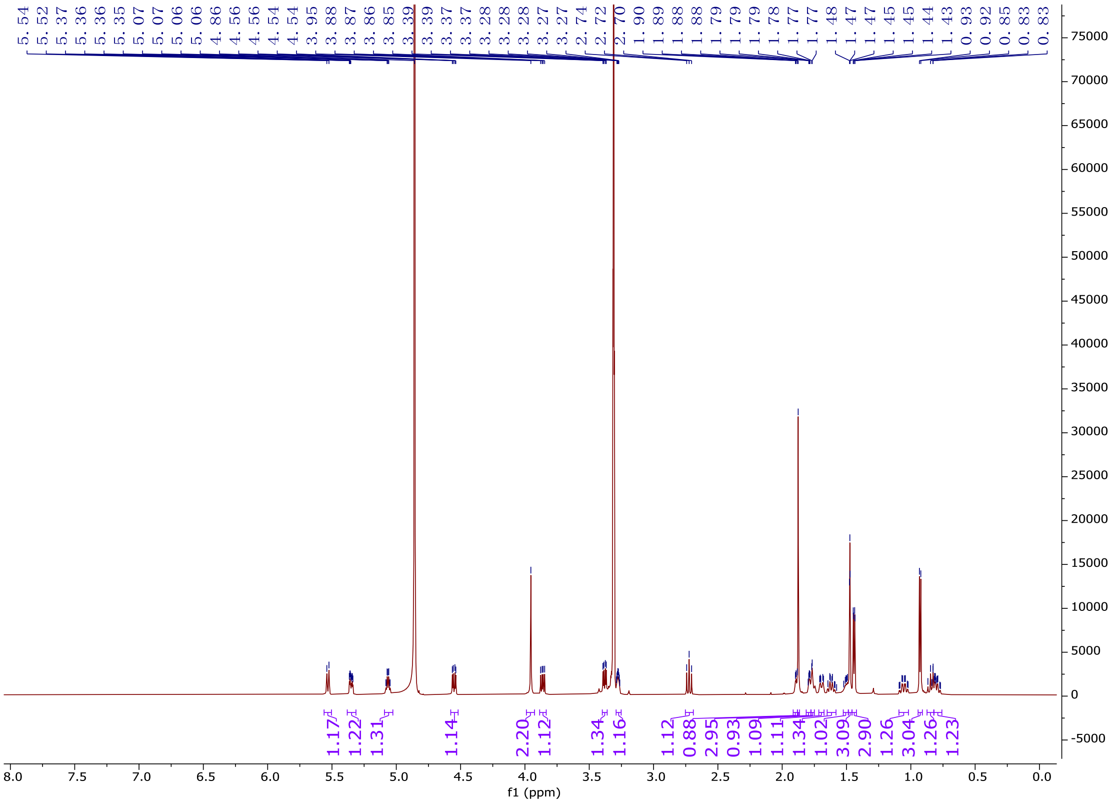

Figure S53. ^{1}H NMR (600M Hz, CD_3OD) spectrum of compound **3**

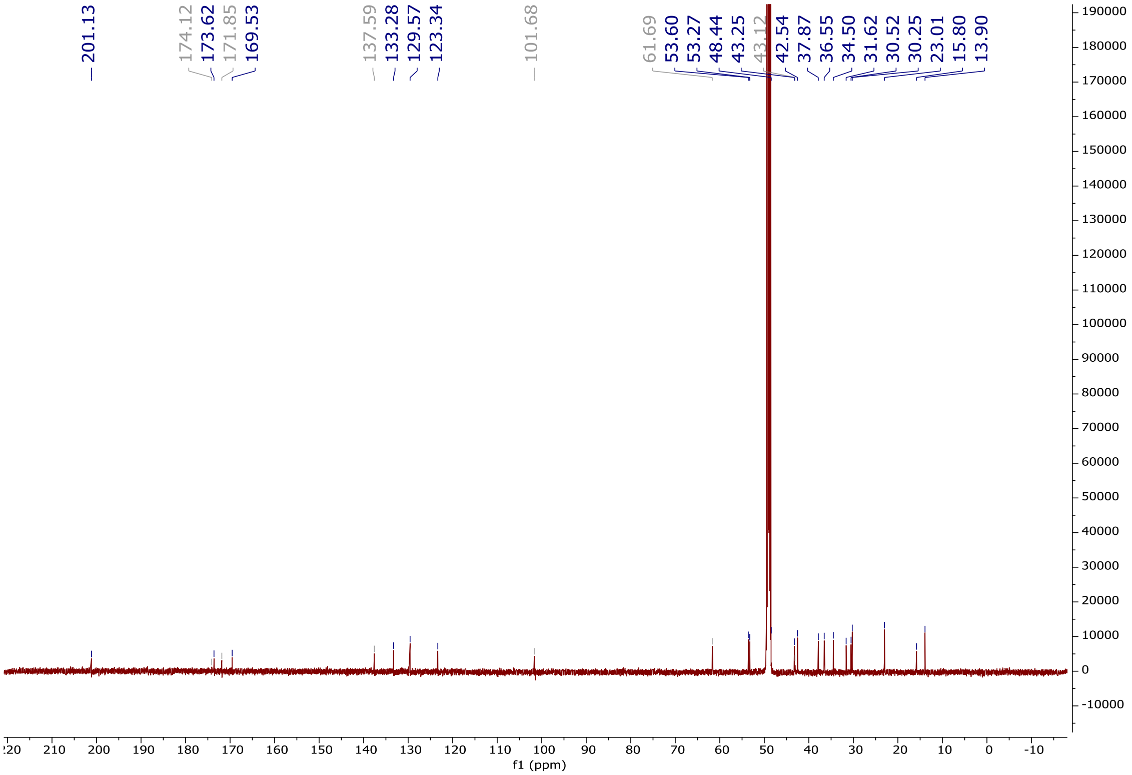

Figure S54. ^{13}C NMR (150M Hz, CD_3OD) spectrum of compound **3**

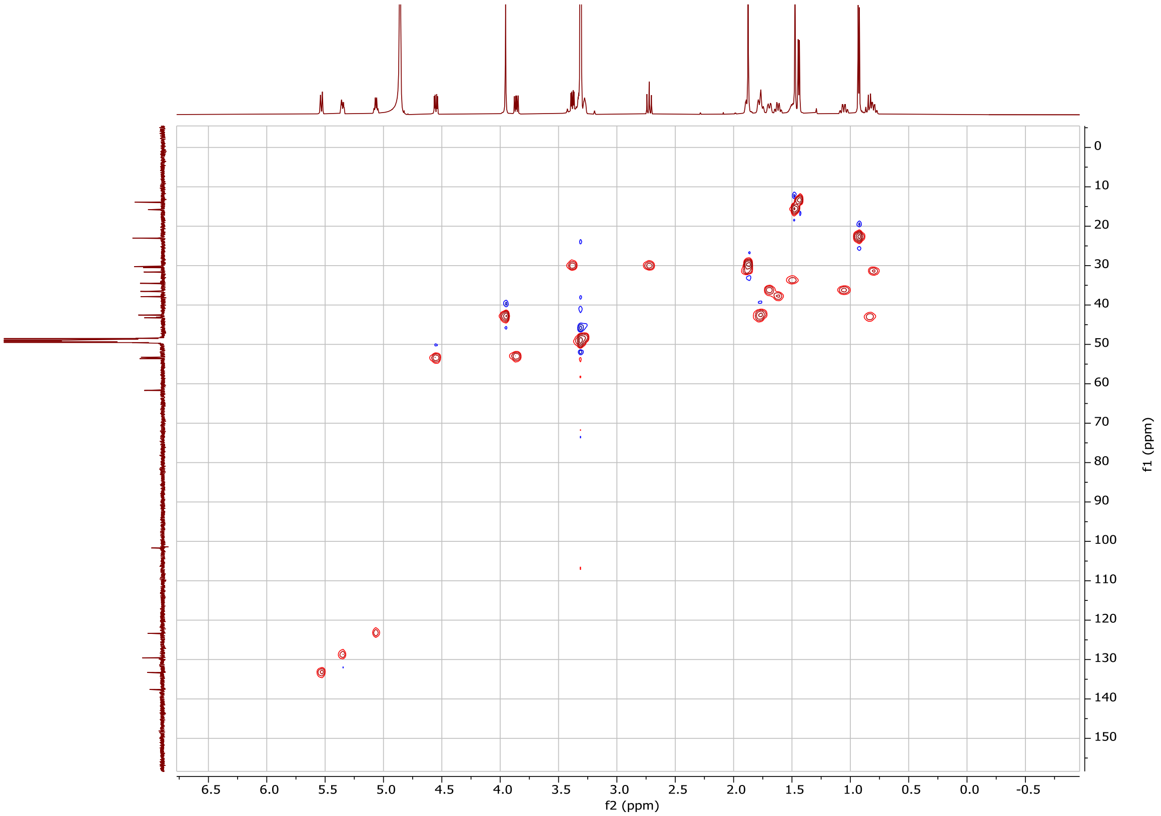

Figure S55. HSQC (CD_3OD) spectrum of compound **3**

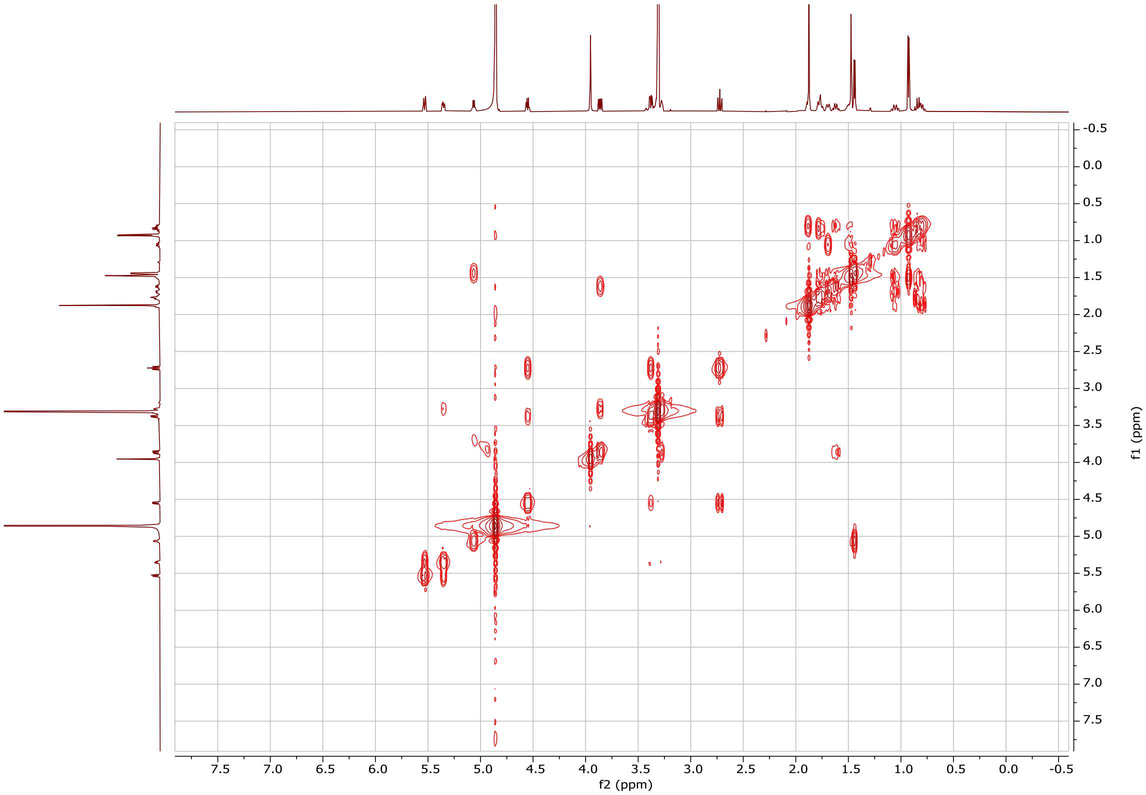

Figure S56. COSY (CD_3OD) spectrum of compound **3**

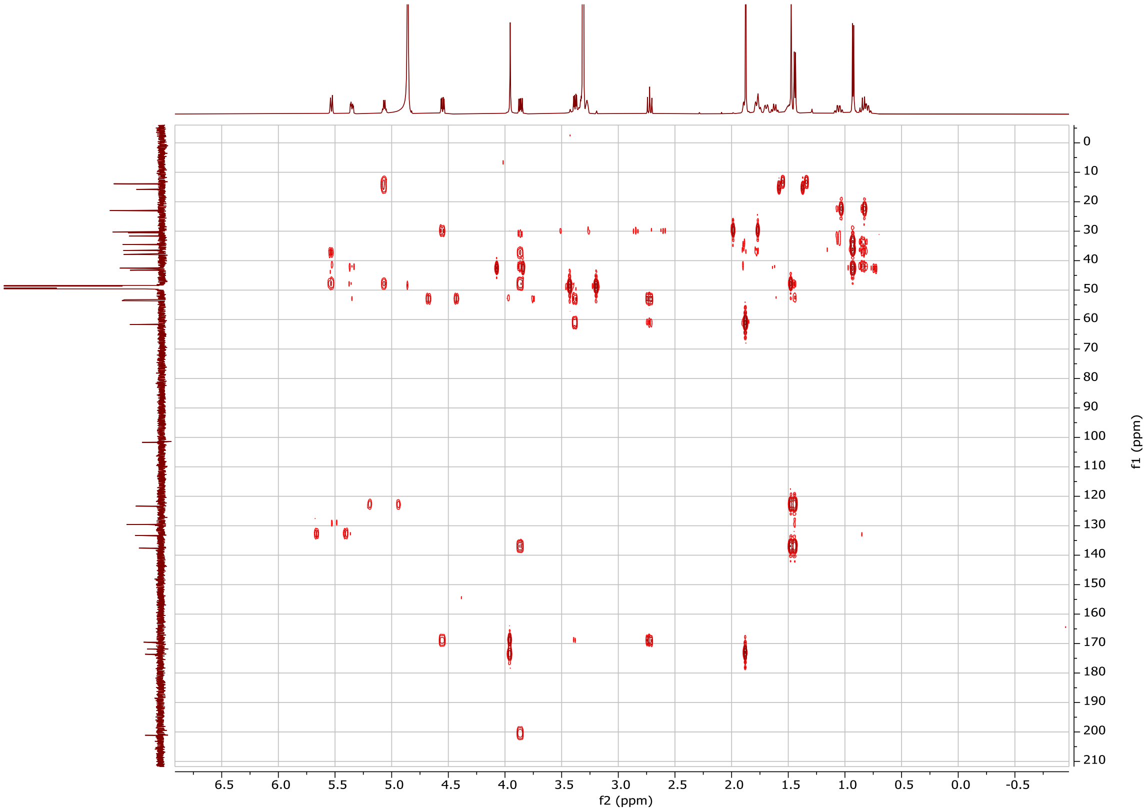

Figure S57. HMBC (CD_3OD) spectrum of compound **3**

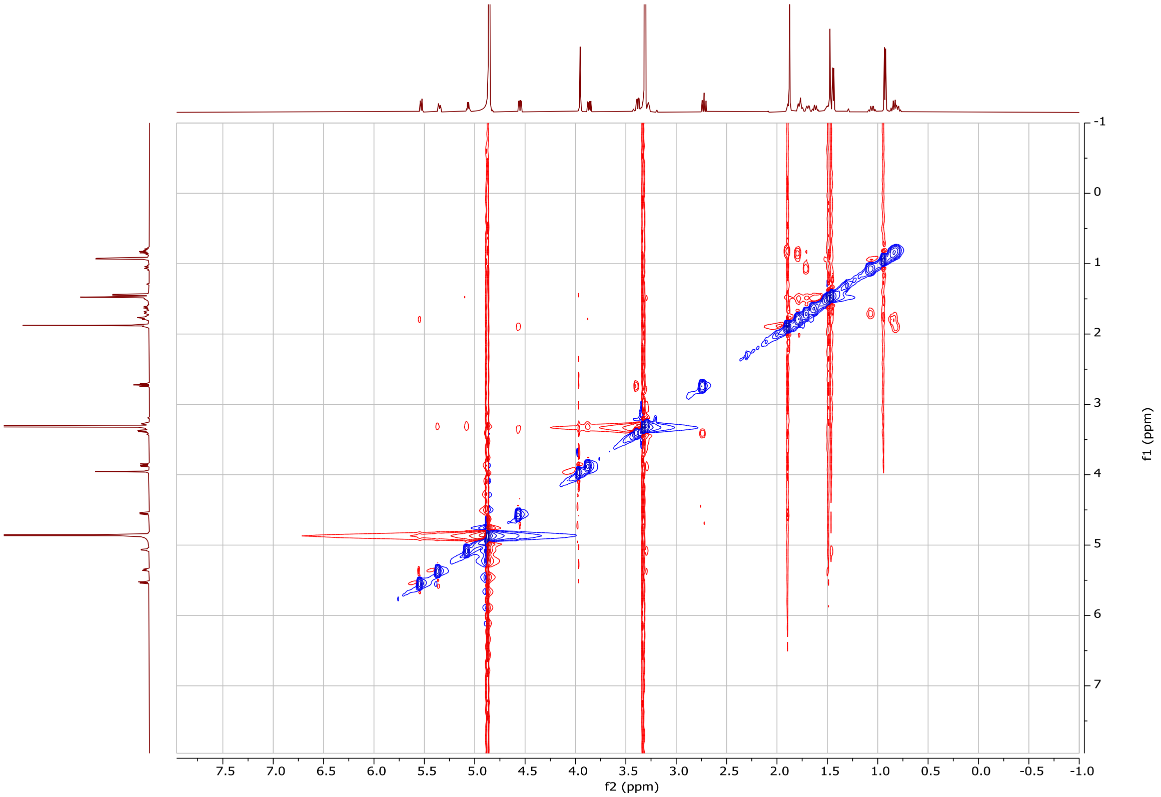

Figure S58. ROESY (CD_3OD) spectrum of compound **3**

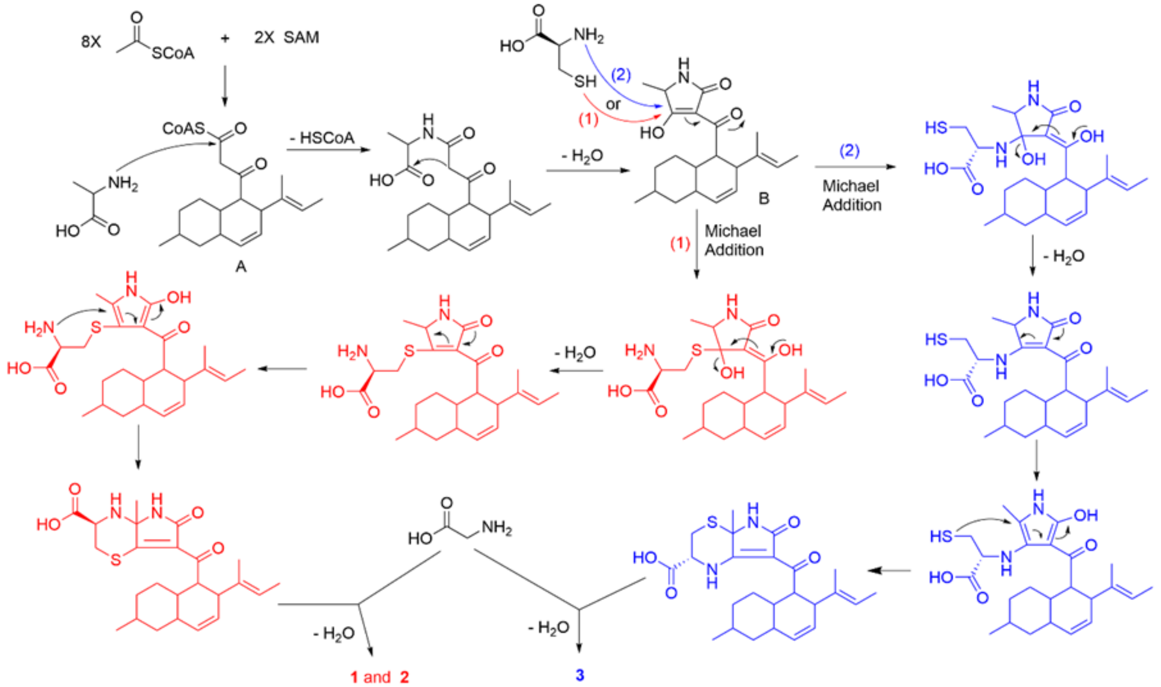

Figure S59. Proposed biosynthetic pathway

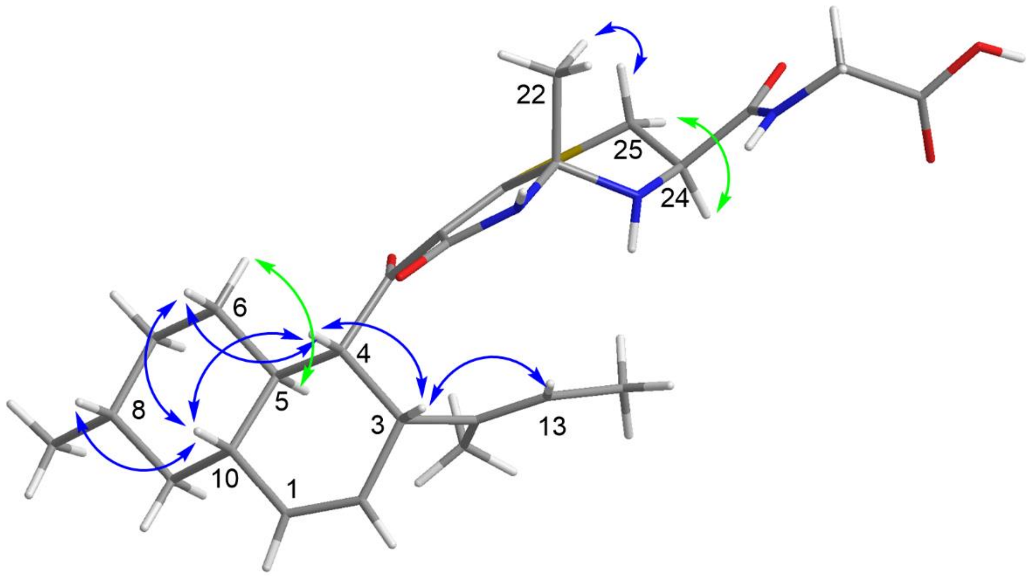

Figure S60. Key ROESY correlations for compound **1**

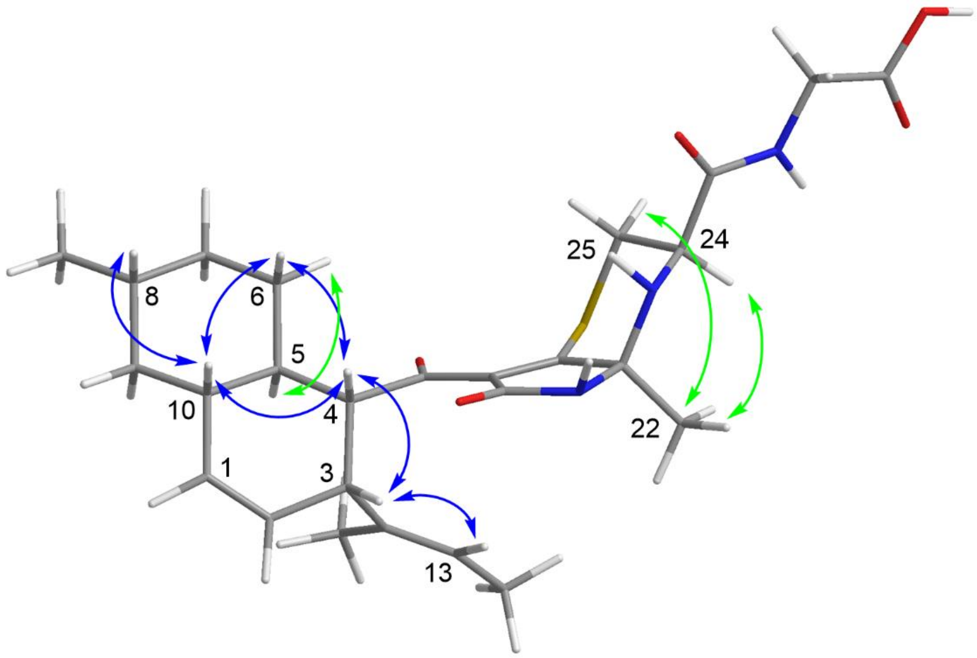

Figure S61. Key ROESY correlations for compound **2**

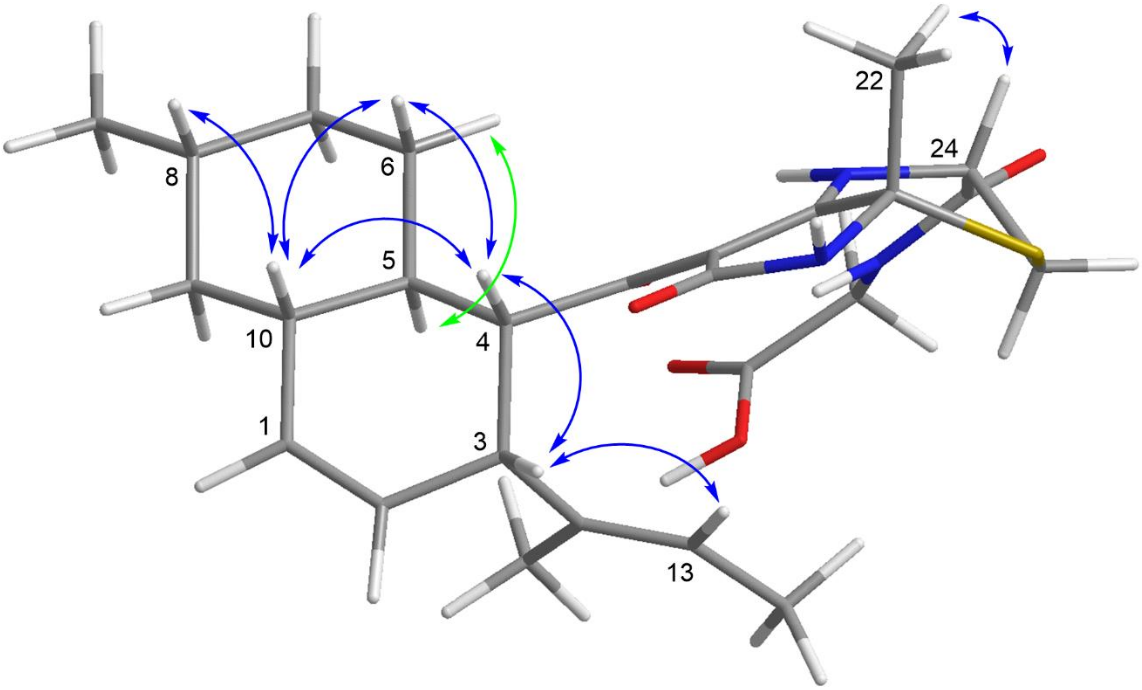

Figure S62. Key ROESY correlations for compound **3**
